# Classification of *GBA1* variants and their impact on Parkinson’s disease: an in silico score analysis

**DOI:** 10.1038/s41531-025-01060-6

**Published:** 2025-08-02

**Authors:** Aymeric Lanore, Christelle Tesson, Aymeric Basset, François-Xavier Lejeune, Guillaume Cogan, Graziella Mangone, Sara Sambin, Nathalie Bertille, Mathieu Anheim, Isabelle Arnulf, Solène Ansquer, Jean-Philippe Brandel, Christine Brefel-Courbon, Luc Defebvre, Sophie Drapier, Alexandre Eusebsio, Margherita Fabbri, Caroline Giordana, Elodie Hainque, Stephane Lehericy, Ana Marques, Caroline Moreau, Elena Moro, Fabienne Ory, Anne-Sophie Rolland, Stéphane Thobois, Marie Vidailhet, David Devos, Louise-Laure Mariani, Suzanne Lesage, Alexis Brice, Jean-Christophe Corvol, the Iceberg study group, Poornima Menon, Jonas Ihle, Caroline Weill, Louise Laure Mariani, Bertrand Degos, Richard Levy, Julie Socha, Marie-Alexandrine Glachant, Sophie Rivaud-Pechoux, Smaranda Leu Semenescu, Pauline Dodet, Samir Bekadar, Fanny Mochel, Farid Ichou, Vincent Perlbarg, Benoit Colsch, Arthur Tenenhaus, Rahul Gaurav, Nadya Pyatigorskaya, Lydia Yahia-Cherif, Romain Valabregue, Cécile Galléa, Marie-Odile Habert, Dijana Petrovska, Laetitia Jeancolas, Alizé Chalançon, Carole Dongmo-Kenfack, Christelle Laganot, Valentine Maheo, Manon Gomes, Mickaël Lé, the NSPark study group, Helene Esperou, Florence Tubach, Yann De Rycke, Stephanie Carvalho, Yajiththa Rajasegaram, Layidé Roufai, Dalila Ouazib, Sandra Zarrad, Fatma Khelif, Nacim Miri, Vanessa Rousseau, Samuel Tessier, David Tavel, Dounya Metdaoui, Avigaelle Abitbol, Mathias Antunes, Fanny Casse, Mélanie Ferrien, Lisa Welment, Silvia Di Legge, Melissa Tir, Thierry Moulin, Claire Thiriez, Philippe Remy, Gwendoline Dupont, Christian Geny, Giovanni Castelnovo, Anne Doe De Maindreville, Mickael Aubignat, Marie Mongin, Arnaud Lapostolle, Matthieu Bereau, Alexandra Foubert-Samier, Brice Laurens, Sylvain Vergnet, Thomas Boraud, David Bendetowicz, Jade Sarrabere, Edouard Courtin, Paul-Alexandre Pfeiffer, Gilles Defer, Bérengère Debilly, Hayet Salhi, Alice Dormeuil, Aimée Petit, Alban Gravier, Vincent Schneider, Lucie Garnier, Philippe Couratier, Chloé Laurencin, Stephane Prange, Paul Jaulent, Bruno Plus, Hélène Gervais-Bernard, Frédérique Fluchere, Valentin Mira, Mahmoud Charif, Ophélie Forster, Alix Durand, Pauline Prin, Armory Jardel, Salome Puisieux, Guillemette Clement, Arthur Lionnet, Adrien De Guilhem De Lataillade, Cosmin Alecu, Charlotte Heraud, Marie De Verdal, Thomas Courtin, Fouad Khoury, Marie Vidhaillet, Aurélie Meneret, Cendrine Foucard, Florian Von Raison, Alexis Elbaz, Andreas Hartmann, Vincent Leclercq, Théodore Soulier, Daniel Torres, Giulia Coarelli, Giorgia Querin, Fabien Hauw, Margaux Dunoyer, Frederique Leh, Marion Leclercq, Simon Lamy, Guillaume Carey, Guillaume Costentin, Clémence Hardy, Ouhaid Legha Boukbiza, Thomas Wirth, Thomas Bogdan, Fabienne Ory-Magne, Clemence Leung, Hélène Catala, Gabrielle Sill, Raquel Pinheiro Barbosa, Lucy Famer, Lucie Braccagni, Hiba Sifaoui, Juliette Palisson, Kenza Benrahmoune Idrissi, Astrid Causel, Lydie Romeo, Constance Bissessur, Audace Cure-Martin, Charline Compagne, Sandrine Dupouy, Sandrine Villars, Wei-Ho Lai, Rachida Bari, Damien Chevanne, Stephane Bernard, Corinne Garsault, Nathalie Meunier, Alexia Cresson, Marine Sgard, Marie Dreano, Justine Montillot, Renaud Massart, Pascale Grebent, Pierre Pelissier, Valérie Santraine, Thomas Gaudin, Pierre Boutet, Cécile Thuilier, Coralie Chalot, Céline Prevost, Hélène Videaud, Justine Picut, Christian Tarrade, Catherine Caire, Hélène Merle, Mathilde Millot, Chloé Bernardi, Emilie Favre, Adelaide Jaulent, Laura Mundler, Blandine Dufresne, Valérie Driss, Alexia Arifi, Maura Rodrigues, Lili Le Monnier, Nathalie Dumont, Virginie Bablon, Régis Frenais, Caroline Herve, Christelle Guimber, Elodie David, Christina Faroul, Leslie Fra, Elsa Foucaran, Fatima-Ezzahra Ennaji, Jérémy Bonetto, Ryad Ladghem-Chikouche, Mickael Lé, Sophie Liot, Sonia Messar, Hamza Salah, Amelie Bernardo, Naoual Serari, Carole David, Zoé Fournier, Margaux Bonnaire-Verdier, Elise Chevaillier, Françoise Kestens, Rozenn Gourhan, Sandra Lopez-Alfaro, Jean-François Houvenaghel, Mélanie Alexandre, Christine Bourdonnais, Ahmed Boumediene, Sandrine Bendele, Hugo Rummel, Céline Julie, Clélie Phillipps, Anne Claire Andries-Ros, Stéphanie Bras, Claudia Gillet, Yoan Herades, Eva Camgrand

**Affiliations:** 1 Sorbonne Université, Institut du Cerveau – Paris Brain Institute – ICM, Inserm, CNRS, Assistance Publique Hôpitaux de Paris, NS-Park/FCRIN network, Department of Neurology, CIC Neurosciences, Hôpital Pitié-Salpêtrière, Paris, France; 2 Assistance Publique Hôpitaux de Paris, Department of Neurology, CIC Neurosciences, Hôpital Pitié-Salpêtrière, Paris, France; 3 AP-HP, Hôpital Pitié Salpêtrière, Centre de Pharmacoépidémiologie (Cephepi), CIC-1901, F75013 Paris, France; 4 Department of Neurology, Neurogenetic Reference Centre, Parkinson’s Expert Centre, University Hospitals of Strasbourg, Strasbourg, France; 5 Assistance Publique Hôpitaux de Paris, Sleep disorders unit, Hôpital Pitié-Salpêtrière, Paris, France; 6 Neurology Department, Centre Expert Parkinson, CIC-INSERM 1402, CHU Poitiers, 86000 Poitiers, NS-PARK/FCRIN Network France; 7 Hôpital Fondation Ophtalmologique A de Rothschild, Unité James Parkisnon, NS-PARK/FCRIN Network, F-75019 Paris, France; 8 Clinical Investigation Center CIC1436, Parkinson Expert Center, Department of Clinical, Pharmacology and Neuroscience, NS-Park/FCRIN network, NeuroToul COEN Center; Toulouse, University Hospital, INSERM and University of Toulouse 3, Toulouse, France; 9 Parkinson’s Disease Center of Excellence, Department of Neurology, University of Lille, CHU Lille, INSERM U1172- Degenerative & Vascular Cognitive Disorders, Lille, France; 10 Pontchaillou University Hospital, Department of Neurology, CIC INSERM 1414, Rennes, France; 11 Aix Marseille Université, AP-HM, Hôpital de La Timone, Service de Neurologie et Pathologie du Mouvement, Rennes, France; 12 Neurology Department, NS-Park/FCRIN network, Centre Hospitalier Universitaire de Nice, Université Côte d’Azur, Nice, France; 13 Assistance Publique Hôpitaux de Paris, Department of Neuroradiology, Hôpital Pitié-Salpêtrière, Paris, France; 14 Université Clermont Auvergne, IGCNC, Department of Neurology, Parkinson expert center, CHU Clermont-Ferrand, Clermont-Ferrand, France; 15 Department of Medical Pharmacology, CHU Lille, University of Lille, Lille Neuroscience & Cognition, INSERM, UMR-S1172, LICEND, NS-Park/F-CRIN network, Lille, France; 16 Grenoble Alpes University, Movement Disorders Unit, Division of Neurology, CHU de Grenoble, Grenoble Institute of Neurosciences, Grenoble, France; 17 Hospices Civils de Lyon, Hôpital Neurologique Pierre Wertheimer, Department of Neurology C, NS-Park/FCRIN network, Univ Lyon, Université Claude Bernard Lyon 1, Faculté de Médecine Lyon Sud Charles Mérieux, Lyon, France; INSERM, Centre de Recherche en Neurosciences de Lyon, PATH-PARK, Bron, France; 18 Department of Neuroradiology, University of Lille, Lille Neuroscience & Cognition, INSERM, UMR-S1172 CHU de Lille Lille, France; 19 Univ Lille, CNRS, INSERM, CHU Lille, Institut Pasteur de Lille, US 41-UAR 2014- PLBS, F-59000 Lille, France; 20 Neurosurgery Department, University of Lillle, CHU Lille, Lille, France; 21 Clinical Studies Pharmacovigilance Department, CHU Lille, Lille, France; 22 CHU de Nantes, CIC1413, Nantes & Université de Nantes, UFR Médecine, Nantes, France; 23 CHU Bordeaux, Service de Neurologie des Maladies Neurodégénératives, IMNc, F-33000 Bordeaux, NS-Park/FCRIN network France; 24 Department of Neurology, Rouen University Hospital and University of Rouen, France; INSERM U1239, Laboratory of Neuronal and Neuroendocrine Differentiation and Communication, NS-Park/FCRIN network, Mont-Saint-Aignan, France; 25 Amiens University Hospital, Department of Neurology, NS-Park/FCRIN network, Amiens, France; 26 Department of Neurology, NS-Park/FCRIN network; University Hospital of Nancy, Nancy, France; 27 Neuroscience Pole, NS-PARK/F-CRIN, Hôpital Foch, Suresnes, University of Versailles Paris-Saclay, INSERM-CEA NeuroSpin, Saclay, France; 28 Univ. Paris-Saclay, CEA, CNRS, Baobab, Neurospin, CATI multicenter neuroimaging platform, Gif-sur-Yvette, France; 29 Centres de Ressources Biologiques- Centre d’investigation clinique, 1403 CHU Lille Lille, France; 30 Clinical Studies Pharmacovigilance Department, CHU Lille, Lille, France ; University of Lille, INSERM, CHU Lille, CIC 1403- Centre d’Investigation Clinique, Lille, France; 31 Department of Biostatistics, University of Lille, CHU de Lille, Lille, France; 32 Neurology Department, Avicenne Hospital, APHP, Hôpitaux Universitaires de Paris-Seine Saint Denis (HUPSSD), Sorbonne Paris Nord, Réseau NS-PARK/FCRIN, Bobigny, France; 33 Foundation for Innovation in Cardiometabolism and Nutrition (IHU ICAN), ICAN I/O Data Science (MPo), ICAN Omics (FI and ML), Paris, France; 34 BrainTale, Strasbourg, France; 35 Université Paris Saclay, CEA, INRAE, Médicaments et Technologie pour la Santé (MTS), Gif-sur-Yvette, France; 36 Université Paris-Saclay, Centrale Supélec, Laboratoire des Signaux et Systèmes, Gif-sur-Yvette, France; 37 AP-HP Department of Nuclear Medicine, Sorbonne University, Paris, France; CNRS, INSERM, Laboratoire d’Imagerie Biomédicale, Sorbonne University, Paris, France; Institut du Cerveau, Sorbonne University, Paris, France; 38 Paris Brain Institute - ICM, Centre de NeuroImagerie de Recherche - CENIR, Paris, France; Laboratoire SAMOVAR, Télécom SudParis, Institut Polytechnique de, Paris, France; 39 Inserm, pôle de recherche clinique, Institut de santé publique, Paris, France; 40 Neurology Department, Pays D’Aix Hospital, Aix-en-Provence, France; 41 Department of Neurology, Hôpital Jean Minjoz, Besançon, France; 42 Department of Neurology, NS-Park/FCRIN network, CHU Caen, Caen, France; 43 Centre Expert Parkinson and NS-Park/FCRIN network, CHU Henri Mondor ; AP-HP et Equipe Neuropsychologie Interventionnelle, INSERM-IMRB, Faculté de Santé, Université Paris-Est Créteil et Ecole Normale Supérieure PSL Université, Créteil, France; 44 François Mitterrand University Hospital, Department of Neurology, University de Bourgogne, Dijon, France; 45 Centre Expert Maladie de Parkinson, Service de Neurologie, Hôpital Gui de Chauliac, Montpellier, France; 46 Department of Neurology, NS-Park/FCRIN network; CHU de Nîmes, Nîmes, France; 47 Department of Neurology, NS-park/FCRIN Network, CHU Reims, Reims, France; 48 Department of Neurology, NS-Park/FCRIN network, Limoges University Hospital, Limoges, France

**Keywords:** Parkinson's disease, Parkinson's disease, Clinical genetics

## Abstract

Bi-allelic pathogenic *GBA1* variants cause Gaucher disease (GD), whereas certain heterozygous missense variants increase the risk of Parkinson’s disease (PD), although the underlying mechanisms are unclear. Here, we classified *GBA1* missense variants using predictive and structural scores, and analysed their associations with enzyme activity, Saposin C (SapC) interaction and PD progression in 639 patients with heterozygous *GBA1* variants from five cohorts. Principal component analysis (PCA) identified two components: PC1, associated with reduced β-glucocerebosidase activity, the GD clinical severity classification, younger age at PD diagnosis, and faster cognitive and motor decline; and PC2, associated with surface-exposed, flexible regions involved in SapC interactions, younger age at PD diagnosis, and slightly with motor decline. These findings highlight that impaired SapC interactions, in addition to reduced activity, may contribute to PD severity in *GBA1* variant carriers. This is relevant for therapeutic approaches aimed at stabilizing β-glucocerebosidase or enhancing its enzymatic activity in PD.

## Introduction

Parkinson’s disease (PD) is a neurodegenerative disorder mainly characterized by the pathological aggregation and spreading of alpha-synuclein (enriched in the so-called Lewy bodies), and the loss of dopaminergic and non-dopaminergic neurons^1^. This leads to the onset and progression of a constellation of motor (akinesia, resting tremor, and rigidity) and non-motor signs and symptoms (cognitive impairment, dysautonomia, sleep issues)^2^.

β-Glucocerebrosidase (GCase), a lysosomal enzyme encoded by *GBA1*, is involved in the catabolism of glycosphingolipids such as glucosylceramide (GlcCer) and glucosylsphingosine, and appears to play a role in the regulation of alpha-synuclein aggregation^3^. The activity and stability of GCase are modulated by Saposin C (SapC), a lysosomal cofactor that facilitates substrate access and protects GCase from proteolytic degradation^4^. Biallelic *GBA1* pathogenic variants cause Gaucher disease (GD), an autosomal recessive lysosomal disorder with a variable phenotype, ranging from asymptomatic cases to severe systemic involvement^5^. Most of these pathogenic variants are missense variants, such as N370S and L444P. Heterozygous *GBA1* variants have been identified as genetic risk factors for developing PD and earlier cognitive impairment^6,7^. More recently, non-GD-causing *GBA1* missense variants, such as E326K and T369M have also been identified as risk factor for PD^8,9^.

*GBA1* variants were originally classified according to the phenotype of patients with GD, with “mild” variants causing non-neuronopathic GD (type 1 GD) and “severe” variants causing neuronopathic GD (types 2 and 3 GD)^10,11^. In GD, the phenotype is linked to the loss of function of GCase, and severe pathogenic variants are indeed associated with a lower GCase activity than mild pathogenic variants. Several studies have demonstrated that the classification of *GBA1* variants into ‘mild’ or ‘severe’ based on GD correlates with PD risk, age at onset, and disease progression. Patients carrying severe variants, such as L444P, exhibit a higher PD risk and earlier onset than those with mild variants or risk factors such as E326K^7,12,13^. However, the mechanisms by which heterozygous *GBA1* variants cause PD and the relationship with GCase activity remain unclear. Therefore, GD clinical classification applied to heterozygous pathogenic variants raises questions about their relevance in terms of association with PD progression, the severity of non-GD-causative missense variants, and the effect of numerous unclassified *GBA1* variants^14^. Standard in silico prediction scores used in clinical laboratories have been developed to assess the pathogenicity of missense variants, such as differences in enzymatic activity^8^. Other structural scores, such as solvent accessibility, provide information on the effect of missense variants on protein surface exposure, which has been shown to be an important feature for pathogenicity^15,16^. In the context of *GBA1*, these in silico scores may allow for the inference of the mechanisms associated with PD progression and gain a better understanding of the PD pathophysiology in patients with heterozygous *GBA1* variants.

In this work, we first aimed to disentangle the effects of *GBA1* missense variants using in silico tools and then determine whether these components were associated with age at PD diagnosis, motor, and cognitive progression in a large cohort of *GBA1* PD carriers.

## Results

### Population

**Fig. 1:**
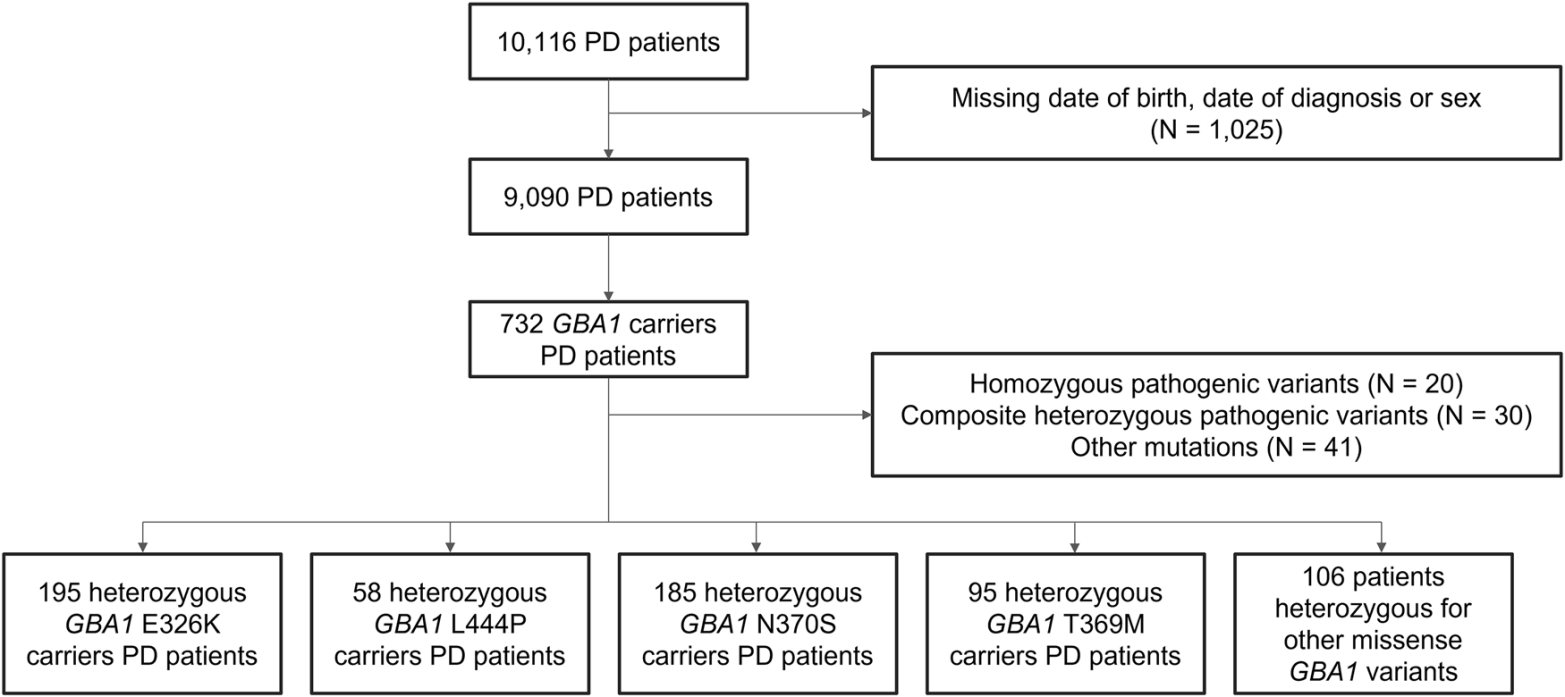
Flow chart.

Of the 732 patients with identified *GBA1* variants, we excluded 93 patients, including 20 patients with homozygous variants, 30 with compound heterozygous variants and 43 with other variants (recombination, nonsense *GBA1* variants and variants in other PD genes) (Fig. 1). After exclusion, the study population included a total of 639 PD patients with heterozygous *GBA1* variants, distributed across several cohorts. Specifically, 280 patients were from the AMPPD study (6.9% of the cohort), 204 patients from the NGC cohort (4.8%), 16 patients from the ICEBERG study (9.5%), 61 patients from the NSPARK cohort (7%), and 78 patients from the PREDISTIM study (9.4%). Patients carried 46 different heterozygous missense *GBA1* variants, with 33% (15/46) of *GBA1* variants classified in the unknown GD category (Table S1). The patients included 195 E326K carriers, 58 L444P carriers, 185 N370S carriers, 95 T369M carriers and 106 patients with other variants (Table 1). The sex ratio between variant types was balanced between groups. Patients with severe and unknown variant categories had a lower median age at diagnosis compared to the other categories (respectively 51.0 y IQR: 11.9 y and 53.0 y IQR: 21.1 y), whereas patients with mild variant had a later median age at diagnosis (59.0 y IQR: 14.9 y) (Table 1).

**Table 1:** Demographic characteristics of the population

GD classification	Risk variant	Mild	Severe	Unknown	Total	*P* value
*N* (%)	293 (45.9)	195 (30.5)	122 (19.1)	29 (4.5)	639	
	E326K: 195/293 (66.6)	N370S: 185/195 (96.9)	L444P: 58/122 (47.5)	Other: 29/29 (100)		
	T369M: 95/293 (32.4)	Other: 10/195 (5.1)	Other: 64/122 (52.5)			
	Other: 3/293 (1.0)					
Male - *N* (%)	164/293 (56.0)	113/195 (58.0)	59/122 (48.4)	12/29 (41.4)	291/639 (54.5)	0.168
Age at diagnosis in years –median (IQR)	56.0 (16.0)	59.0 (14.9)	51.0 (11.9)	53.0 (21.1)	55.0 (15.3)	**<0.001**
Patients included in the longitudinal cohort *N* (%)	188 (49.0)	125 (32.6)	57 (14.8)	14 (3.7)	384	
	E326K: 122/188 (64.9)	N370S: 121/125 (96.8)	L444P: 23/57 (40.4)	Other: 14/14 (100)		
	T369M: 64/188 (34.0)	Other: 4/125 (3.2)	Other: 34/57 (59.7)			
	Other: 2/188 (1.1)					
Male–*N* (%)	108/188 (57.5)	69/125 (55.2)	28/57 (49.01)	5/14 (35.7)	210/384 (54.7)	0.343
Age at diagnosis in years –median (IQR)	56.0 (14.0)	59.9 (13.5)	50.9 (10.8)	55.0 (18.1)	56.0 (14.0)	**<0.001**
Duration between diagnosis and baseline in years–median (IQR)	4.4 (8.0)	3.0 (4.8)	5.2 (6.1)	1.1 (6.0)	3.8 (6.7)	**0.033**
Education below 12 years–*N* (%)	52/183 (28.4)	22/124 (17.7)	21/57 (36.8)	5/14 (35.7)	100/378 (26.5)	**0.030**

Statistical tests used were the Kruskal–Wallis test for numerical variables and the Chi-square test for categorical variables.

*P* values in bold correspond to group differences below the level of statistical significance of 5%.

### Variant analysis

**Fig. 2:**
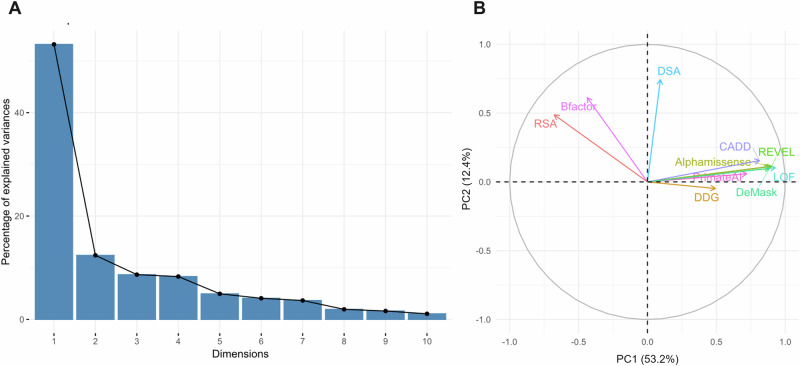
Principal component analysis of *GBA1* missense variants. **A** Percentage of explained variance by the principal component analysis. **B** Variable plot of the first two principal components. DDG free energy difference, RSA relative solvent accessibility, DSA delta of solvent accessibility, LOF Loss of function.

We retained 2906 *GBA1* variants from 495 residues with available VEP and structural scores. Following standardization, we performed a PCA on these variants. The first principal component (PC1) accounted for 53% of the variance, while the second principal component (PC2) explained 12% (Fig. 2A, B). The contributions of the various scores to PC1 and PC2 are detailed in Table S2A, and scores are available in Table S1.

**Fig. 3:**
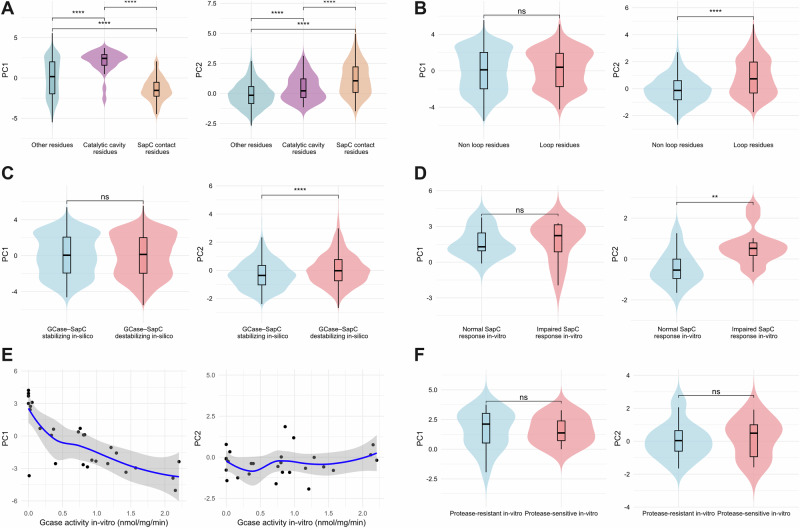
Principal component classification of *GBA1* missense variants by structural features and in vitro functional assays. **A** Distributions of PC1 and PC2 scores for *GBA1* missense variants stratified by structural spatial context: non-catalytic cavity/non–Saposin C contact residues (other residues, blue, *n* = 2659), catalytic cavity residues (purple, *n* = 96), and Saposin C contact residues (orange, *n* = 151)^61^. **B** Comparison of PC1 and PC2 distributions between loop residues (red, *n* = 220) and non-loop residues (blue, *n* = 2686)^61^. Loop regions are typically associated with increased flexibility and surface exposure. **C** in silico analysis of the GCase–Saposin C interface using AlphaFold3 predictions. Variants were classified as stabilizing (blue, *n* = 511) or destabilizing (red, *n* = 2395) based on predicted effects on protein–protein interactions. **D** In vitro Saposin C activation response among variants for which recombinant β-glucocerebosidase activity was measured in the presence or absence of Saposin C^53^. Variants were grouped as responsive (blue, *n* = 11) or non-responsive (red, *n* = 14). **E** Relationship between PC scores and recombinant β-glucocerebosidase activity (*n* = 28)^54^. PC1 and PC2 scores were plotted against in vitro activity values. **F** Protease resistance analysis based on Cathepsin D digestion assays^53^. Variants were classified as protease-resistant (blue, *n* = 18) or protease-sensitive (red, *n* = 9), reflecting differences in protein folding stability. Violin plots display the distribution density, with overlaid boxplots showing the median and interquartile range. Statistical significance was assessed using Wilcoxon rank-sum; ns = not significant, *P < 0.05, **P < 0.01, ***P < 0.001, ****P < 0.0001. Spearman’s correlation coefficient (*R*) is shown, with non-linear loess smoothing used to visualize trends. Shaded regions represent 95% confidence intervals.

**Fig. 4:**
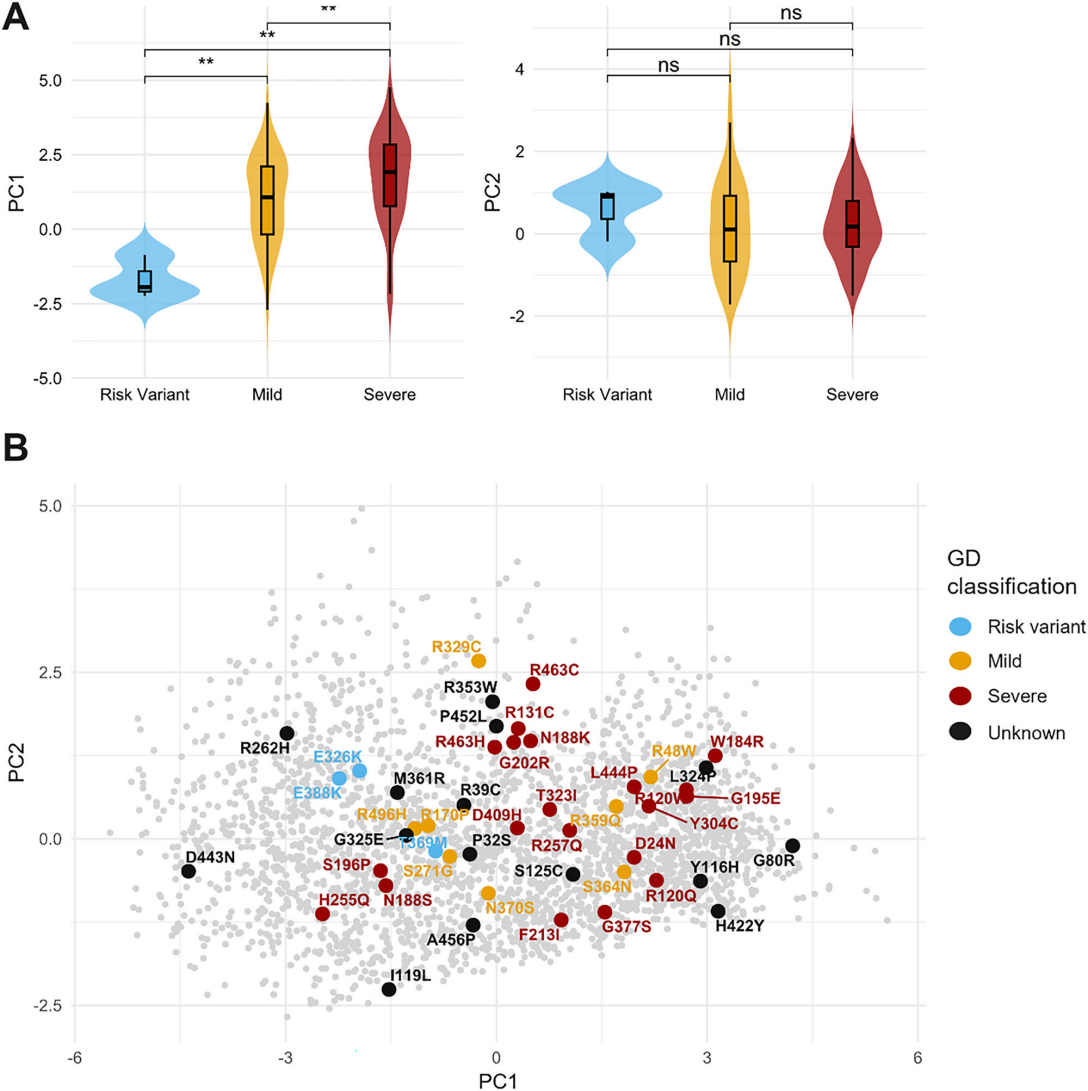
Comparison of Gaucher disease and principal component classification of *GBA1* missense variants. **A** Gaucher disease classification according to PC1 and PC2, respectively. Variants are classified as risk variants (*N* = 3), mild (*N* = 55) or severe (*N* = 74). Statistical significance was assessed using Wilcoxon rank-sum; ns not significant, *P < 0.05, **P < 0.01, ***P < 0.001, ****P < 0.0001. **B** Plot of *GBA1* missense variants according to PC1 and PC. Color key: Blue = Risk variant, Orange = Mild, Red = Severe.

Variants located within the catalytic cavity exhibited significantly higher PC1 scores compared to other residues, while those located in the SapC contact region showed elevated PC2 scores (Fig. 3A). Similarly, variants positioned in flexible loop regions displayed significantly higher PC2 scores compared to non-loop residues, while no significant difference in PC1 was observed (Fig. 3B). in silico modeling of the GCase–SapC interface predicted that variants destabilizing the protein–protein interaction had significantly higher PC2 scores, with no association observed for PC1 (Fig. 3C). In in vitro SapC activation assays, variants classified as non-responsive to SapC-mediated activation exhibited significantly higher PC2 values compared to responsive variants (Fig. 3D). PC1 scores were strongly associated with reduced recombinant GCase enzymatic activity (*P* < 0.001), whereas PC2 showed no significant relationship with enzymatic activity (Fig. 3E). Protease resistance assays based on Cathepsin D digestion revealed no significant differences in either PC1 or PC2 scores between protease-resistant and protease-sensitive variants (Fig. 3F). While PC1 was significantly different across GD classifications, no significant difference was observed for PC2 (Fig. 4).

In the Partial Least Squares (PLS) regression model using enzymatic activity as the response variable, the lowest prediction error was achieved with a single latent component—indicating that PLS Component 1 (PLS1) captures the most relevant signal predictive of GCase activity (Fig. S1A, B). When applied across the full set of *GBA1* variants, inverted PLS1 scores were strongly and positively correlated with PC1 (Pearson’s *R* = 0.99, *P* < 0.001) with similar feature loadings observed between the two components (Table S2B), whereas a weaker inverse correlation was observed with PC2 (Pearson’s *R* = –0.10, *P* < 0.001) (Fig. S1C, D).

### *GBA1* variant and enzymatic activity ex vivo

**Fig. 5:**
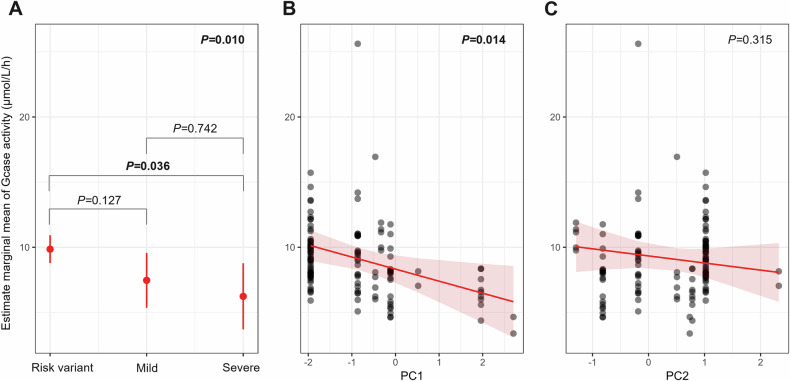
Estimated marginal means of β-glucocerebosidase activity according to Gaucher disease and principal component classification. These results are derived from linear mixed-effects models, where β-glucocerebrosidase activity is the dependent variable. **A** Estimated marginal means of β-glucocerebosidase activity (μmol/L/h) across Gaucher disease classification (risk variant, mild or severe). Pairwise comparisons of estimated means are shown with associated *p* values. **B** Relationship between β-glucocerebosidase activity and principal component 1 (PC1). Each dot represents an individual sample; the red line indicates the model-predicted slope with a 95% confidence interval. **C** Relationship between β-glucocerebosidase activity and principal component 2 (PC2). No significant association was observed. Gcase β-glucocerebosidase.

Blood GCase activity was available for 127 visits from 43 patients with *GBA1* variants, 17 patients with E326K, 10 patients with T369M, 7 with N370S, 3 with L444P and 6 with other variants. GD classification was significantly associated with GCase enzymatic activity (*P* = 0.021). In the post hoc analysis, the only significant association was a lower GCase activity in severe *GBA1* carriers compared to risk variant carriers (*P* = 0.036) (Fig. 5A). Patients with higher PC1 had a significantly lower blood GCase activity (*P* = 0.014) while PC2 was not significantly associated with lower GCase activity (Fig. 5B, C). These results were similar when excluding L444P carriers (Table S3).

### *GBA1* classification and age at diagnosis

**Fig. 6:**
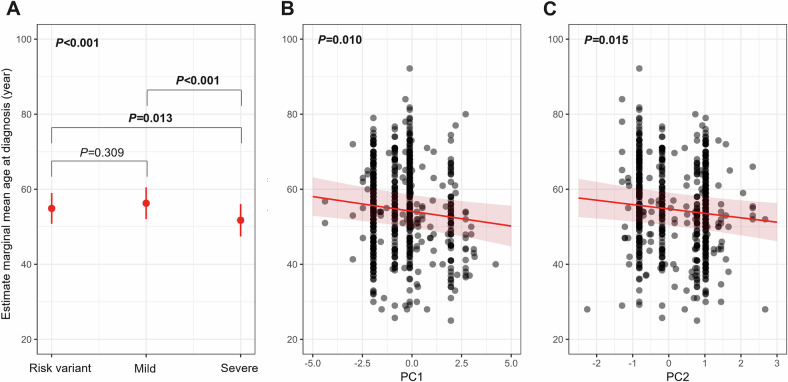
Estimated marginal means of age at diagnosis according to Gaucher disease and principal component classification. These results are derived from linear mixed-effects models. **A** Estimated marginal means of age at diagnosis (in years) across Gaucher disease classification (risk variant, mild or severe). Pairwise comparisons of estimated means are shown with corresponding *p* values. **B** Relationship between age at diagnosis and principal component 1 (PC1). Each dot represents an individual; the red line shows the fitted slope with 95% confidence interval. **C** Relationship between age at diagnosis and principal component 2 (PC2), also showing model-estimated slope and confidence interval.

Among the 639 patients, GD classification was significantly associated with age at diagnosis (*P* < 0.001). Patients with severe variants had lower age at diagnosis compared to patients with risk variants (*P* = 0.013) and patients with mild variants (*P* < 0.001) (Fig. 6A). Higher PC1 and PC2 values were significantly associated with younger age at diagnosis (*P* = 0.010 and *P* = 0.015, respectively) (Fig. 6B, C). These associations remained significant in the sensitivity analysis, excluding L444P carriers (Table S4).

### *GBA1* classification and disease progression

In the longitudinal analysis, 384 patients were included, corresponding to 1558 visits, with a median of two visits per patient (IQR 5), and a median follow-up of one year (IQR 3.6 years). Sex ratios, variant proportions and age at diagnosis were similar to the whole cohort (Table 1). The median duration between the diagnosis and baseline visit was 3.8 years (IQR: 6.7 years), with no significant differences between GD classification categories. Education was significantly different between GD categories, with more patients with education below twelve years in the severe and unknown categories (Table 1).

**Fig. 7:**
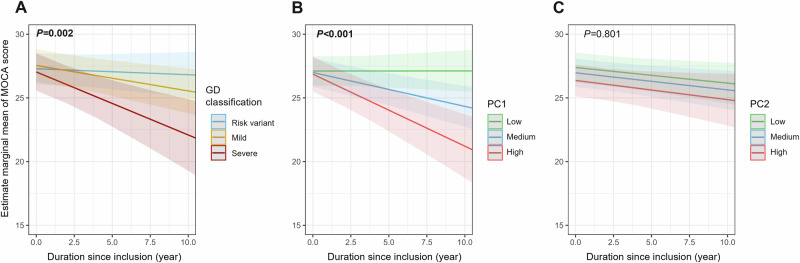
Estimated marginal means of MOCA score during follow-up according to Gaucher disease and principal component classification. These results are derived from linear mixed-effects models. The estimated marginal means represent predicted slopes of cognitive decline (MoCA score). **A** Estimated marginal mean trajectories of MoCA scores over time by Gaucher disease classification (risk variant, mild or severe). A significant difference in cognitive decline slopes is observed between groups. (Color key: Blue = Risk variant, Orange = Mild, Red = Severe). **B** Predicted MoCA score change by PC1. Individuals with higher PC1 scores show significantly faster cognitive decline. (Color key: Green = Low tercile PC score, Blue = Medium tercile, Red = High tercile). **C** Predicted MoCA score change by PC2. No significant differences were detected. (Color key: Green = Low tercile PC score, Blue = Medium tercile, Red = High tercile). Shaded areas represent 95% confidence intervals. The estimated slopes reflect adjusted marginal means derived from the fitted LMMs.

In the cognitive analysis using linear mixed-effects model (LMM), GD classification was significantly associated with cognitive decline during follow-up (*P* = 0.002) (Table S5). In post hoc analysis at ten years, severe variant carriers had a higher cognitive decline compared to risk variant (*P* = 0.002) and mild variants (*P* = 0.040) carriers (Table S5A, Fig. 7A). Higher PC1 values were associated with higher cognitive decline (*P* < 0.001). The predicted decrease of MoCA over 10-year follow-up was 0.1 ± 1.1 point for patients with low PC1 tercile, 2.8 ± 1.1 points for medium PC1 tercile and 5.7 ± 1.5 points for high PC1 tercile. PC2 was not significantly associated with cognitive decline during follow-up. In the sensitivity analysis using generalized estimating equations (GEE), the results were consistent for the PC-based classification, which remained significantly associated with cognitive decline. However, the association with GD classification was no longer statistically significant. The results remain similar in the sensitivity analysis excluding L444P carriers (Table S5).

**Fig. 8:**
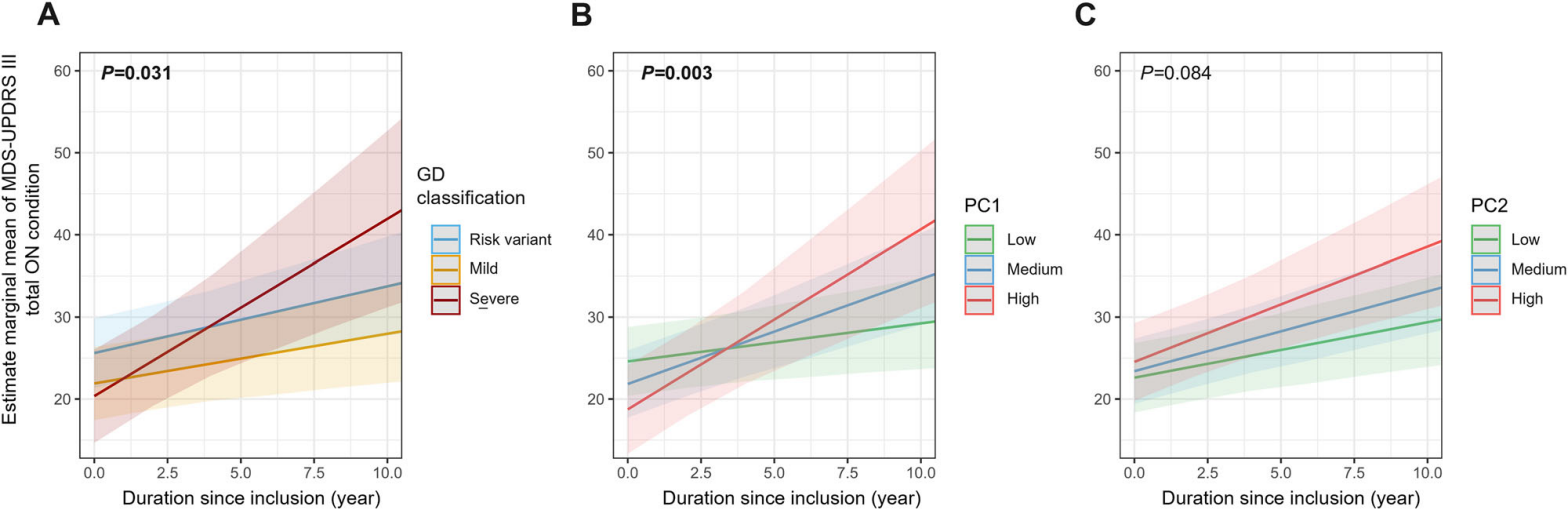
Estimated marginal means of MDS-UPDRS III ON condition score during follow-up according to Gaucher disease and principal component classification. These results are derived from linear mixed-effects models. The estimated marginal means represent predicted slopes of motor symptom progression (MDS-UPDRS III ON condition). **A** Predicted trajectories of MDS-UPDRS III scores over time by Gaucher disease classification (risk variant, mild or severe). A significant difference in motor progression slopes is observed across groups. (Color key: Blue = Risk variant, Orange = Mild, Red = Severe). **B** Predicted MDS-UPDRS III score change by PC1. Individuals with higher PC1 scores show significantly faster motor progression. (Color key: Green = Low tercile PC score, Blue = Medium tercile, Red = High tercile). **C** Predicted MDS-UPDRS III score change by PC2. Individuals with higher PC2 scores show a trend toward faster motor progression. (Color key: Green = Low tercile PC score, Blue = Medium tercile, Red = High tercile). Shaded areas represent 95% confidence intervals. The estimated slopes reflect adjusted marginal means derived from the fitted LMMs.

For the motor analysis using LMM, GD classification was significantly associated with motor progression (*P* = 0.031). In the post hoc analysis at 10 years, only severe variants showed significantly higher motor progression compared with mild variants (*P* = 0.034) (Fig. 8A, Table S6A). The association was no longer significant in the sensitivity analysis after excluding L444P carriers. Higher PC1 values were significantly associated with faster motor progression (*P* = 0.003) (Fig. 8B, Table S6B). The predicted increase of MDS-UPDRS III ON over 10-year follow-up was 4.6 ± 3.5 points for patients with low PC1 tercile, 12.8 ± 3.5 points for medium PC1 tercile, and 21.9 ± 5.5 points for high PC1 tercile. There was a trend toward higher motor progression for higher PC2 (*P* = 0.084) (Fig. 8C, Table S6B). This association was significant after excluding L444P carriers. In contrast, the sensitivity analysis using GEE yielded non-significant results for both GD classification and PC-based classifications (Table S6).

## Discussion

Our analysis identified distinct disease progression profiles in PD patients with *GBA1* variants based on two components of in silico scores. Higher PC1 values were linked to lower GCase activity and faster motor and cognitive decline, while higher PC2 values were associated with younger age at diagnosis, and a trend toward faster motor progression, but not with GCase activity or cognitive decline. Additionally, in this large dataset, we confirmed that severe *GBA1* variants, according to the classic clinical GD classification, were associated with lower GCase activity, earlier onset, and faster progression of both cognitive and motor symptoms. We demonstrate that VEP scores, which are designed to estimate the impact of variants on protein function, were mostly represented in PC1, and reflect GCase enzyme activity, as further confirmed by PLS regression. In PD, the enzymatic activity was shown to be reduced for mild and severe variants compared to risk variant carriers^17^, and this GD classification has been shown to influence age at onset and disease progression^7,12,13^. We consistently show that PC1 was associated with reduced enzymatic activity of GCase and cognitive progression in PD *GBA1* patients. The main representative of the mild form, the N370S variant in the glycosyl hydrolase TIM domain, results in a stable GCase with reduced enzyme activity^18^. Experimental evidence suggests that reduced GCase activity is associated with the propagation of the synucleinopathy^19^. In cell culture models, GCase deficiency has been demonstrated to exacerbate existing alpha-synuclein pathology and facilitate the spread of alpha-synuclein fibrils^20–22^. Consistently, in vivo studies have shown that reduced GCase activity significantly increases the dissemination of alpha-synuclein pathology in mice^21,23^. The reduction in GCase enzymatic activity leading to increased propagation of synuclein may provide a mechanistic explanation for its association with accelerated cognitive decline.

Structural scores were mostly represented in PC2, which was associated with residue surface exposure, local flexibility, and proximity to the SapC interaction interface. These features suggest that PC2 reflects a distinct axis of structural perturbation—less related to catalytic impairment. Consistently, in silico modeling indicated that variants predicted to destabilize the GCase–SapC interface were also enriched for higher PC2 values, and in vitro data showed that variants with altered response to SapC activation exhibited higher PC2 scores. Notably, the risk variant E326K—which is associated with PD but not GD—has been reported to impair GCase–SapC binding^24,25^. This observation supports the hypothesis that disruption of cofactor interactions, as captured by PC2, may contribute to PD pathogenesis. Clinically, higher PC2 scores were associated with earlier onset of motor symptoms and showed a trend toward faster motor progression, while no significant association was observed with cognitive decline. This pattern aligns with growing evidence that GCase dysfunction may promote synucleinopathy through mechanisms beyond enzymatic loss. SapC normally binds to GCase, enhancing its enzymatic activity and protecting it from proteolysis^4^. Impaired binding to SapC may destabilize GCase, reducing its functional activation and increasing its vulnerability to degradation. Furthermore, impaired GCase–SapC interaction may destabilize GCase and compromise chaperone-mediated autophagy, promoting α-synuclein accumulation—particularly in dopaminergic neurons^26,27^. This may explain why higher PC2 scores are selectively associated with motor progression but not cognitive decline. Although PC2 correlates with structural metrics such as B-factor and solvent accessibility—features known to influence protein folding and stability^28,29^—we did not observe significant differences in protease susceptibility. This suggests that PC2 may reflect a structural flexibility that, while not inherently destabilizing, could predispose to misfolding under stress or during biogenesis. Clinical laboratories commonly use tools such as CADD and REVEL for variant classification, while structural prediction tools are less commonly used, despite their potential relevance. Such tools offer complementary insights by assessing the impact of variants on protein interaction interfaces, which could be particularly valuable in understanding the variant pathogenicity beyond what is captured by traditional scores.

This study presents a well-characterized cohort of PD patients with *GBA1* variants, with a particular focus on heterozygous carriers and detailed clinical profiling. However, this study has several limitations. We used data from different cohorts, which may have introduced heterogeneity in the studied population, and differences in genetic screening methods across cohorts could have introduced bias. To address these issues, we accounted for the study effect in the LMM and excluded post-DBS visits in the PREDISTIM cohort to avoid potential bias in cognitive and motor progression assessments^30^. Although our models adjusted for key clinical covariates and cohort-level effects, we acknowledge that other potential confounders—such as polygenic risk scores, environmental exposures, and comorbidities—were not systematically accounted for and may also influence disease trajectories. The frequency of *GBA1* missense variants was similar to previous reports, with E326K, T369M, N370S, and L444P accounting for more than 80% of PD cases^7,31,32^. Due to this overrepresentation, the weight of frequent variants was particularly high, potentially driving the observed differences, especially for severe variants. The robustness of our results was tested by excluding carriers of the severe L444P variant in sensitivity analyses, which yielded similar findings. To further evaluate the robustness of our findings, we used GEE to assess population-averaged effects of *GBA1* variants on longitudinal changes in cognitive and motor scores. The results from the GEE analysis were consistent with our main findings for cognitive progression, thereby reinforcing the robustness of the association. However, for motor progression, GEE results were less stable and non-significant, likely reflecting the greater inter-individual variability in motor symptoms, which are influenced by heterogeneous disease trajectories and treatment effects. Unlike GEE, LMMs incorporate random effects, allowing for the modeling of subject-specific slopes and intercepts; hence, they are better suited for capturing individual-level variability in motor progression^33^. Studies with larger sample sizes are needed to confirm our findings, particularly for rare variants. Given that our study population primarily includes individuals of European ancestry, caution is warranted when generalizing these findings to non-European populations. However, the proposed framework may help to prioritize and functionally contextualize rare or population-specific variants. Establishing the link between heterozygous variants and biological mechanisms is challenging, and our data on GCase activity are limited. Although a significant difference in ex vivo GCase activity was observed between severe and risk variant carriers, other comparisons (e.g., risk vs. mild, mild vs. severe) did not reach statistical significance, likely reflecting the limited sample size in this subset (*n* = 43). Similarly, the lack of a statistically significant association between PC2 and ex vivo GCase activity should be interpreted with caution, as the observed trend suggests a potential biological signal that may become significant with larger cohorts. Future studies incorporating high-throughput functional assays, such as Multiplex Assays for Variant Effects (MAVEs), will be essential to systematically characterize the effects of *GBA1* missense variants on enzymatic function and cofactor interactions, thereby refining our understanding of their contribution to disease risk and progression^34^. Integration of multimodal biomarkers—such as neuroimaging, fluid-based measures, and transcriptomic profiles—may further enhance the predictive power of variant classification by capturing complementary dimensions of disease biology and individual heterogeneity.

In conclusion, we show that the PC1, predominantly represented by VEP scores, aligns with GD classification and is associated with reduced enzymatic activity, as well as motor and cognitive decline. In contrast, PC2, predominantly represented by structural scores, was associated with age at diagnosis and progression of motor symptoms. These results suggest that, beyond diminished GCase enzymatic activity, pathogenicity in PD among *GBA1* variant carriers may also be driven by impaired SapC interaction. Our PCA-based classification provides a scalable and biologically informed tool for prognostic assessment and future stratification of PD patients with heterozygous *GBA1* variants, particularly in the context of clinical trials evaluating targeted therapies, such as chaperone molecules aimed at stabilizing GCase.

## Methods

### Study design

Data from five cohorts were analyzed: Accelerating Medicine Partnership – Parkinson’s Disease (AMPPD) cohort, from eight North American cohorts (*N* = 4038)^35^; ICEBERG a French monocentric cohort, recruiting patients between 2014 and 2022 (*N* = 168) (NCT02305147); the “Noyaux Gris Centraux” (NGC) cohort, a French multicentric cohort recruiting patients between 1990 and 2021, enriched for patients with familial and early-onset PD (*N* = 4210); NSPARK, a French real-life multicentric prospective cohort, recruiting patients between 2021 and 2024 (*N* = 867)^36^; and PREDISTIM, a multicentric French cohort, recruiting patients between 2013 and 2019 if eligible and planning to undergo deep brain stimulation (DBS) (*N* = 833)^37^.

All clinically diagnosed PD patients^38^ with complete demographic data (sex, date of birth, date of diagnosis, date of visit) and carrying a heterozygous pathogenic missense variant of *GBA1* were included. Pathogenic missense variants of *GBA1* were selected if they were defined as pathogenic or likely pathogenic by the American College of Medical Genetics (ACMG)^39^ or were identified in the literature as a risk factor for PD. For consistency, patients with nonsense variants, homozygous and compound heterozygous missense variants, and structural variants of *GBA1* were excluded. Patients with mutations identified in other PD genes (*LRRK2, PRKN, VPS35, PARK7, SNCA* and *PINK1*) were also excluded. For the longitudinal analysis, only patients with available motor and cognitive data were retained. Patients with deep brain stimulation (DBS) were not included because of its potential effect on cognitive and motor function.

All participants provided written informed consent. The genetic and clinical studies were approved by local ethics committees: PREDISTIM (RCB: 2013-A00193-42), ICEBERG (RCB: 2014-A00725-42), and NSPARK (CPPIDF1-2020-ND58). Studies involving the NGC were reviewed and approved by the CCPPRB (Comité Consultatif de Protection des Personnes dans la Recherche Biomédicale) of the Groupe Hospitalier Pitié-Salpêtrière, Paris, France. AMPPD data were accessed under a data use agreement; all contributing cohorts obtained informed consent and ethics approval at their respective sites.

### Data collection

Sex and age at PD diagnosis were collected in all five cohorts. The baseline visit was defined as the first visit reported in each cohort. In the AMPPD, NSPARK, ICEBERG and PREDISTIM cohorts, level of education (above and below twelve years), Montreal Cognitive Assessment (MoCA), Movement Disorder Society-Unified Parkinson’s Disease Rating Scale (MDS-UPDRS) part III ON state were collected at baseline and follow-up visits.

For the GCase activity, we used previously published data available in the AMPPD cohort using dried blood spots prepared from blood^40^. GCase enzyme activity was measured using a multiplex assay. The enzyme activity of each sample was calculated from the ratio of the ion abundance of the product to that of the internal standard measured by mass spectrometry. Activity was expressed as micromoles of product per litre of whole blood per hour (µmol/L/h).

### Genetic analysis

Patients were screened for pathogenic variants in PD-associated genes using a combination of sequencing and targeted molecular techniques. In the NGC, NSPARK, ICEBERG and PREDISTIM cohorts, patients were screened for *GBA1* variants using long-range PCR (LR-PCR) with two overlapping fragments covering all exons and gene-specific primers to avoid *GBA1* pseudogene (*GBA1LP*)^41^, followed by sequencing on MiSeq 2000 (Illumina)^42^ or Sanger sequencing as previously described^43^. In addition, the most common autosomal dominant PD mutations were assessed using the same method: *LRRK2* Ex41, *VPS35* Ex15, and *SNCA* Ex3. Patients were also screened for the *LRRK2* Gly2019Ser variant using the TaqMan allelic discrimination Assay-By-Design method. *PRKN* or *SNCA* rearrangements were evaluated using Multiplex Ligation-dependent Probe Amplification (MLPA) with MRC-Holland Salsa MLPA P051/P052 Parkinson kits, following the manufacturer’s protocol. Furthermore, PD patients with age at onset below 45 years old or with an autosomal recessive form underwent WES to look for rare variants in genes linked to PD or Parkinsonian syndromes.

Genetic data from AMPPD subjects for the 7 PD genes (*GBA1, LRRK2, VPS35, SNCA, PRKN, PARK7* and *PINK1*) were obtained by analyzing DNA microarray data, WES, whole genome sequencing (WGS), Sanger sequencing of variants in the *GBA1* gene and RNA sequencing. For the E326K and T369M *GBA1* variants, rs2230288 and rs75548401 were extracted from WGS data.

### Variant analysis

*GBA1* variants were classified according to GD–based severity as “risk factor”, “mild”, or “severe”, following the classifications provided by the *GBA1* Browser^11^. For each variant, multiple variant effect predictors (VEPs) were gathered, including two widely used scores (CADD^44^ and REVEL^45^), two newer ones (PrimateAI^46^ and AlphaMissense^47^), and two scores focused on loss-of-function (LOF) effects (DeMask^48^ and LOF score^49^) (Table S7). We also collected structural scores, including free energy difference calculated by FoldX (version 5.0), relative solvent accessibility (RSA), delta solvent accessibility (DSA), and B-factor available in SIGMA^15^ and Envision^16^ (Table S7). Structural annotations of residues were derived from a previously published reference^50^, categorizing residues into catalytic cavity (*n* = 96) and SapC contact sites (*n* = 151). Loop residues (*n* = 220) were similarly defined. in silico modeling of the GCase–SapC interface was performed using AlphaFold3-predicted structures^51^, and the impact of missense variants on the interaction was assessed using the SAAMBE prediction tool^52^. Variants were classified as “stabilizing” (*n* = 511) or “destabilizing” (*n* = 2395) based on predicted effects on the GCase–SapC binding energy. We collected in vitro data from previously published sources^53,54^. GCase enzymatic activity was measured as the mean of three independent replicates of *GBA1* missense variants expressed in HEK293T cells, reported in nmol/mg/min^54^. SapC activation status was determined based on recombinant GCase activation profiles relative to wild-type (WT) GCase in the presence of SapC^53^. Variants were classified as SapC-responsive (*n* = 11) or non-responsive (*n* = 14) depending on whether their mean enzymatic activity after SapC exposure, plus one standard deviation, was higher than the WT mean activation level. Protease sensitivity was assessed based on susceptibility to Cathepsin D digestion assays reported in the same study^53^. Variants labeled as “highly unstable” or “dead enzyme” were considered protease-sensitive (*n* = 9), while all other variants were classified as protease-resistant.

All missense variants with available scores were included in the variant analysis. We performed a principal component analysis (PCA) on the different scores, retaining the first two principal components (PC1 and PC2) for each variant. For the first two PCs, we performed a Spearman correlation with in vitro GCase activity. Group comparisons were performed using Wilcoxon rank-sum tests. To investigate the relationship between in silico scores of *GBA1* missense variants and GCase enzymatic activity, we also performed a partial least squares (PLS) regression analysis^55^. We selected the 28 variants for which in vitro enzymatic activity measurements were available. Structural and functional annotations for each variant were scaled and used as predictors, while the corresponding normalized enzymatic activity served as the response variable. A PLS model was trained using leave-one-out cross-validation, and model performance was evaluated using the root mean squared error of prediction (RMSEP)^56^. The optimal number of components was selected based on the lowest RMSEP. The trained model was then applied to the full dataset to compute PLS scores for all annotated variants. To facilitate interpretation, the direction of Component 1 scores (PLS1) was inverted, and the resulting scores were used for downstream correlation analyses and visualization. We classified *GBA1* variants according to i) GD classification categories: risk variants (non-GD causing but risk variants for PD), mild (causing type I GD) and severe (causing type II and III GD as previously described^11^) and ii) PC classification (PC1 and PC2).

### Statistical analyses

Quantitative variables were described using median and interquartile range (IQR), and categorical variables were described using counts and percentages (%). Comparisons of demographic characteristics between the variant categories and the GD classification categories were made using the Kruskal–Wallis test for numerical variables and the Chi-2 test for categorical variables.

To model the enzymatic activity in patients, we used the available blood enzymatic activity of GCase in the AMPPD cohort. We analyzed enzymatic activity according to the GD classifications and PCA classification through an LMM, with enzymatic activity as the dependent variable, patient groups with the two covariates sex and age at visit as fixed effects and subject identifiers as random effects. We analyzed age at diagnosis according to the GD classification and PCA classification through an LMM for each classification with age at diagnosis as the dependent variable, including sex as a covariate and a random effect on the cohorts. A sensitivity analysis was used to assess the robustness of the model after excluding the L444P carriers.

We used data from the AMPPD, NSPARK, ICEBERG and PREDISTIM cohorts to model the effect of *GBA1* variant classifications on motor and cognitive progression. For motor decline, we performed LMM with MDS-UPDRS III total score in ON condition as the dependent variable and subject identifiers and cohort as random effects. The fixed effects of the model were sex, age at diagnosis, disease duration between diagnosis and baseline, *GBA1* variant classifications and interaction of each of these variables with duration since baseline.

To model cognitive decline, we performed LMM with MoCA score as the dependent variable, and with subject identifiers and cohort as random effects. The fixed effects were sex, age at diagnosis, education (below 12 years), disease duration between diagnosis and baseline, *GBA1* variant classifications, and interaction of each of these variables with duration since baseline during the study. Sensitivity analyses were performed by excluding the L444P carriers.

Missing data were not imputed: only patients with complete demographic and clinical data were included in each analysis. Analyses were performed with R software version 4.0.3 (version 4.0.3, R Core Team 2020; https://www.R-project.org/). All models were obtained with the restricted maximum likelihood estimation method using the ‘lme4’ R package (v1.1-32)^50^. Model assumptions and fit were checked by a visual inspection of the diagnostic residual plots generated with the ‘ggResidpanel’ R package (v0.3.0)^57^.

Based on the models, the relationships of enzyme activity, age of onset, cognitive and motor progression with the *GBA1* variant classifications were reported in terms of *p* value. *P* values were calculated using Type II Wald Chi-square tests from the ‘car’ R package (v3.1–2)^58^. To examine the effect of the interaction between *GBA1* classification and time to diagnosis on the dependent variable, the estimated marginal means were calculated using the ‘emmeans’ R package (v1.8.9)^59^. The significance level for statistical tests was set at 0.05 (two-tailed). Whenever the GD classification was significant, we conducted a post hoc analysis using Tukey’s method from ‘emmeans’ to identify pairwise differences between categories for GCase activity, age at diagnosis, and at the 10-year mark from inclusion for the longitudinal analysis.

To assess the robustness of our findings on clinical progression, we conducted a sensitivity analysis using GEE, modeling the population-averaged effects of *GBA1* variant classifications on both motor and cognitive scores over time. GEE models were specified with an exchangeable correlation structure to account for within-subject dependencies and included the same fixed effects as in the LMMs: sex, age at diagnosis, education (for MoCA), disease duration between diagnosis and baseline, *GBA1* variant classification, and interactions of these variables with follow-up time. Analyses were performed using the geepack R package (v1.3–2)^60^, with robust sandwich estimators for standard errors. A sensitivity analysis excluding L444P carriers was also conducted within the GEE models.

## Supplementary information

Supplementary merged

## Data Availability

The datasets generated and analyzed during the current study are available from the corresponding author upon request. The genotype and clinical data for the AMP PD cohorts are available through the Accelerating Medicine Partnership® (AMP®) Parkinson’s Disease (AMP PD) Knowledge Platform. For up-to-date information on the study, visit https://www.amp-pd.org. Clinical longitudinal data and genotyping data for the other cohorts included are accessible through appropriate data-sharing agreements that protect participant privacy with the institutions that conducted or are conducting study consents and clinical assessments under local institutional review board approvals.
