# Author Correction: Classification of *GBA1* variants and their impact on Parkinson’s disease: an in silico score analysis

**DOI:** 10.1038/s41531-025-01204-8

**Published:** 2025-11-21

**Authors:** Aymeric Lanore, Christelle Tesson, Aymeric Basset, François-Xavier Lejeune, Guillaume Cogan, Graziella Mangone, Sara Sambin, Nathalie Bertille, Mathieu Anheim, Isabelle Arnulf, Solène Ansquer, Jean-Philippe Brandel, Christine Brefel-Courbon, Luc Defebvre, Sophie Drapier, Alexandre Eusebsio, Margherita Fabbri, Caroline Giordana, Elodie Hainque, Stephane Lehericy, Ana Marques, Caroline Moreau, Elena Moro, Fabienne Ory, Anne-Sophie Rolland, Stéphane Thobois, Marie Vidailhet, David Devos, Louise-Laure Mariani, Suzanne Lesage, Alexis Brice, Jean-Christophe Corvol, the Iceberg study group, Poornima Menon, Jonas Ihle, Caroline Weill, Louise Laure Mariani, Bertrand Degos, Richard Levy, Julie Socha, Marie-Alexandrine Glachant, Sophie Rivaud-Pechoux, Smaranda Leu Semenescu, Pauline Dodet, Samir Bekadar, Fanny Mochel, Farid Ichou, Vincent Perlbarg, Benoit Colsch, Arthur Tenenhaus, Rahul Gaurav, Nadya Pyatigorskaya, Lydia Yahia-Cherif, Romain Valabregue, Cécile Galléa, Marie-Odile Habert, Dijana Petrovska, Laetitia Jeancolas, Alizé Chalançon, Carole Dongmo-Kenfack, Christelle Laganot, Valentine Maheo, Manon Gomes, Mickaël Lé, the NSPark study group, Helene Esperou, Florence Tubach, Yann De Rycke, Stephanie Carvalho, Yajiththa Rajasegaram, Layidé Roufai, Dalila Ouazib, Sandra Zarrad, Fatma Khelif, Nacim Miri, Vanessa Rousseau, Samuel Tessier, David Tavel, Dounya Metdaoui, Avigaelle Abitbol, Mathias Antunes, Fanny Casse, Mélanie Ferrien, Lisa Welment, Silvia Di Legge, Melissa Tir, Thierry Moulin, Claire Thiriez, Philippe Remy, Gwendoline Dupont, Christian Geny, Giovanni Castelnovo, Anne Doe De Maindreville, Mickael Aubignat, Marie Mongin, Arnaud Lapostolle, Matthieu Bereau, Alexandra Foubert-Samier, Brice Laurens, Sylvain Vergnet, Thomas Boraud, David Bendetowicz, Jade Sarrabere, Edouard Courtin, Paul-Alexandre Pfeiffer, Gilles Defer, Bérengère Debilly, Hayet Salhi, Alice Dormeuil, Aimée Petit, Alban Gravier, Vincent Schneider, Lucie Garnier, Philippe Couratier, Chloé Laurencin, Stephane Prange, Paul Jaulent, Bruno Plus, Hélène Gervais-Bernard, Frédérique Fluchere, Valentin Mira, Mahmoud Charif, Ophélie Forster, Alix Durand, Pauline Prin, Armory Jardel, Salome Puisieux, Guillemette Clement, Arthur Lionnet, Adrien De Guilhem De Lataillade, Cosmin Alecu, Charlotte Heraud, Marie De Verdal, Thomas Courtin, Fouad Khoury, Marie Vidhaillet, Aurélie Meneret, Cendrine Foucard, Florian Von Raison, Alexis Elbaz, Andreas Hartmann, Vincent Leclercq, Théodore Soulier, Daniel Torres, Giulia Coarelli, Giorgia Querin, Fabien Hauw, Margaux Dunoyer, Frederique Leh, Marion Leclercq, Simon Lamy, Guillaume Carey, Guillaume Costentin, Clémence Hardy, Ouhaid Legha Boukbiza, Thomas Wirth, Thomas Bogdan, Fabienne Ory-Magne, Clemence Leung, Hélène Catala, Gabrielle Sill, Raquel Pinheiro Barbosa, Lucy Famer, Lucie Braccagni, Hiba Sifaoui, Juliette Palisson, Kenza Benrahmoune Idrissi, Astrid Causel, Lydie Romeo, Constance Bissessur, Audace Cure-Martin, Charline Compagne, Sandrine Dupouy, Sandrine Villars, Wei-Ho Lai, Rachida Bari, Damien Chevanne, Stephane Bernard, Corinne Garsault, Nathalie Meunier, Alexia Cresson, Marine Sgard, Marie Dreano, Justine Montillot, Renaud Massart, Pascale Grebent, Pierre Pelissier, Valérie Santraine, Thomas Gaudin, Pierre Boutet, Cécile Thuilier, Coralie Chalot, Céline Prevost, Hélène Videaud, Justine Picut, Christian Tarrade, Catherine Caire, Hélène Merle, Mathilde Millot, Chloé Bernardi, Emilie Favre, Adelaide Jaulent, Laura Mundler, Blandine Dufresne, Valérie Driss, Alexia Arifi, Maura Rodrigues, Lili Le Monnier, Nathalie Dumont, Virginie Bablon, Régis Frenais, Caroline Herve, Christelle Guimber, Elodie David, Christina Faroul, Leslie Fra, Elsa Foucaran, Fatima-Ezzahra Ennaji, Jérémy Bonetto, Ryad Ladghem-Chikouche, Mickael Lé, Sophie Liot, Sonia Messar, Hamza Salah, Amelie Bernardo, Naoual Serari, Carole David, Zoé Fournier, Margaux Bonnaire-Verdier, Elise Chevaillier, Françoise Kestens, Rozenn Gourhan, Sandra Lopez-Alfaro, Jean-François Houvenaghel, Mélanie Alexandre, Christine Bourdonnais, Ahmed Boumediene, Sandrine Bendele, Hugo Rummel, Céline Julie, Clélie Phillipps, Anne Claire Andries-Ros, Stéphanie Bras, Claudia Gillet, Yoan Herades, Eva Camgrand

**Affiliations:** 1 Sorbonne Université, Institut du Cerveau – Paris Brain Institute – ICM, Inserm, CNRS, Assistance Publique Hôpitaux de Paris, NS-Park/FCRIN network, Department of Neurology, CIC Neurosciences, Hôpital Pitié-Salpêtrière, Paris, France; 2 Assistance Publique Hôpitaux de Paris, Department of Neurology, CIC Neurosciences, Hôpital Pitié-Salpêtrière, Paris, France; 3 CAP-HP, Hôpital Pitié Salpêtrière, Centre de Pharmacoépidémiologie (Cephepi), Paris, France; 4 Department of Neurology, Neurogenetic Reference Centre, Parkinson’s Expert Centre, University Hospitals of Strasbourg, Strasbourg, France; 5 Assistance Publique Hôpitaux de Paris, Sleep disorders unit, Hôpital Pitié-Salpêtrière, Paris, France; 6 Neurology Department, Centre Expert Parkinson, CIC-INSERM 1402, CHU Poitiers, Poitiers, NS-PARK/FCRIN Network France; 7 Hôpital Fondation Ophtalmologique A de Rothschild, Unité James Parkisnon, NS-PARK/FCRIN Network, Paris, France; 8 Clinical Investigation Center CIC1436, Parkinson Expert Center, Department of Clinical, Pharmacology and Neuroscience, NS-Park/FCRIN network, NeuroToul COEN Center; Toulouse, University Hospital, INSERM and University of Toulouse 3, Toulouse, France; 9 Parkinson’s Disease Center of Excellence, Department of Neurology, University of Lille, CHU Lille, INSERM U1172- Degenerative & Vascular Cognitive Disorders, Lille, France; 10 Pontchaillou University Hospital, Department of Neurology, CIC INSERM 1414, Rennes, France; 11 Aix Marseille Université, AP-HM, Hôpital de La Timone, Service de Neurologie et Pathologie du Mouvement, Rennes, France; 12 Neurology Department, NS-Park/FCRIN network, Centre Hospitalier Universitaire de Nice, Université Côte d’Azur, Nice, France; 13 Assistance Publique Hôpitaux de Paris, Department of Neuroradiology, Hôpital Pitié-Salpêtrière, Paris, France; 14 Université Clermont Auvergne, IGCNC, Department of Neurology, Parkinson expert center, CHU Clermont-Ferrand, Clermont-Ferrand, France; 15 Department of Medical Pharmacology, CHU Lille, University of Lille, Lille Neuroscience & Cognition, INSERM, UMR-S1172, LICEND, NS-Park/F-CRIN network, Lille, France; 16 Grenoble Alpes University, Movement Disorders Unit, Division of Neurology, CHU de Grenoble, Grenoble Institute of Neurosciences, Grenoble, France; 17 Hospices Civils de Lyon, Hôpital Neurologique Pierre Wertheimer, Department of Neurology C, NS-Park/FCRIN network, Univ Lyon, Université Claude Bernard Lyon 1, Faculté de Médecine Lyon Sud Charles Mérieux, Lyon, France; INSERM, Centre de Recherche en Neurosciences de Lyon, PATH-PARK, Bron, France; 18 Department of Neuroradiology, University of Lille, Lille Neuroscience & Cognition, INSERM, CHU de Lille Lille, France; 19 Univ Lille, CNRS, INSERM, CHU Lille, Institut Pasteur de Lille, US 41-UAR 2014- PLBS, Lille, France; 20 Neurosurgery Department, University of Lillle, CHU Lille, Lille, France; 21 Clinical Studies Pharmacovigilance Department, CHU Lille, Lille, France; 22 CHU de Nantes, CIC1413, Nantes & Université de Nantes, UFR Médecine, Nantes, France; 23 CHU Bordeaux, Service de Neurologie des Maladies Neurodégénératives, IMNc, Bordeaux, NS-Park/FCRIN network France; 24 Department of Neurology, Rouen University Hospital and University of Rouen, France; INSERM U1239, Laboratory of Neuronal and Neuroendocrine Differentiation and Communication, NS-Park/FCRIN network, Mont-Saint-Aignan, France; 25 Amiens University Hospital, Department of Neurology, NS-Park/FCRIN network, Amiens, France; 26 Department of Neurology, NS-Park/FCRIN network; University Hospital of Nancy, Nancy, France; 27 Neuroscience Pole, NS-PARK/F-CRIN, Hôpital Foch, Suresnes, University of Versailles Paris-Saclay, INSERM-CEA NeuroSpin, Saclay, France; 28 Univ. Paris-Saclay, CEA, CNRS, Baobab, Neurospin, CATI multicenter neuroimaging platform, Gif-sur-Yvette, France; 29 Centres de Ressources Biologiques- Centre d’investigation clinique, 1403 CHU Lille Lille, France; 30 Clinical Studies Pharmacovigilance Department, CHU Lille, Lille, France ; University of Lille, INSERM, CHU Lille, CIC 1403- Centre d’Investigation Clinique, Lille, France; 31 Department of Biostatistics, University of Lille, CHU de Lille, Lille, France; 32 Neurology Department, Avicenne Hospital, APHP, Hôpitaux Universitaires de Paris-Seine Saint Denis (HUPSSD), Sorbonne Paris Nord, Réseau NS-PARK/FCRIN, Bobigny, France; 33 Foundation for Innovation in Cardiometabolism and Nutrition (IHU ICAN), ICAN I/O Data Science (MPo), ICAN Omics (FI and ML), Paris, France; 34 BrainTale, Strasbourg, France; 35 Université Paris Saclay, CEA, INRAE, Médicaments et Technologie pour la Santé (MTS), Gif-sur-Yvette, France; 36 Université Paris-Saclay, Centrale Supélec, Laboratoire des Signaux et Systèmes, Gif-sur-Yvette, France; 37 AP-HP Department of Nuclear Medicine, Sorbonne University, Paris, France; CNRS, INSERM, Laboratoire d’Imagerie Biomédicale, Sorbonne University, Paris, France; Institut du Cerveau, Sorbonne University, Paris, France; 38 Paris Brain Institute - ICM, Centre de NeuroImagerie de Recherche - CENIR, Paris, France; Laboratoire SAMOVAR, Télécom SudParis, Institut Polytechnique de, Paris, France; 39 Inserm, pôle de recherche clinique, Institut de santé publique, Paris, France; 40 Neurology Department, Pays D’Aix Hospital, Aix-en-Provence, France; 41 Department of Neurology, Hôpital Jean Minjoz, Besançon, France; 42 Department of Neurology, NS-Park/FCRIN network, CHU Caen, Caen, France; 43 Centre Expert Parkinson and NS-Park/FCRIN network, CHU Henri Mondor ; AP-HP et Equipe Neuropsychologie Interventionnelle, INSERM-IMRB, Faculté de Santé, Université Paris-Est Créteil et Ecole Normale Supérieure PSL Université, Créteil, France; 44 François Mitterrand University Hospital, Department of Neurology, University de Bourgogne, Dijon, France; 45 Centre Expert Maladie de Parkinson, Service de Neurologie, Hôpital Gui de Chauliac, Montpellier, France; 46 Department of Neurology, NS-Park/FCRIN network; CHU de Nîmes, Nîmes, France; 47 Department of Neurology, NS-park/FCRIN Network, CHU Reims, Reims, France; 48 Department of Neurology, NS-Park/FCRIN network, Limoges University Hospital, Limoges, France

**Keywords:** Parkinson's disease, Parkinson's disease, Clinical genetics

Correction to: *npj Parkinson’s Disease* 10.1038/s41531-025-01060-6, published online 02 August 2025.

In this article, the ‘Competing interests’ statement was missing and should have been ‘Elena Moro was the Associate Publisher of npj Parkinson’s Disease at the time of publication and was not involved in the peer review or editorial decision for this article. All other authors declare no competing interests’.

