# Supplementary material for: Classification of GBA1 variants and their impact on Parkinson’s disease: an in silico score analysis
Source: NPJ Parkinsons Dis. 2025 Aug 2;11:226. doi: 10.1038/s41531-025-01060-6 (PMC12318041; doi:10.1038/s41531-025-01060-6)
Supplement: Supplementary file 1 — Supplementary merged [file 41531_2025_1060_MOESM1_ESM.pdf]

Supplementary material

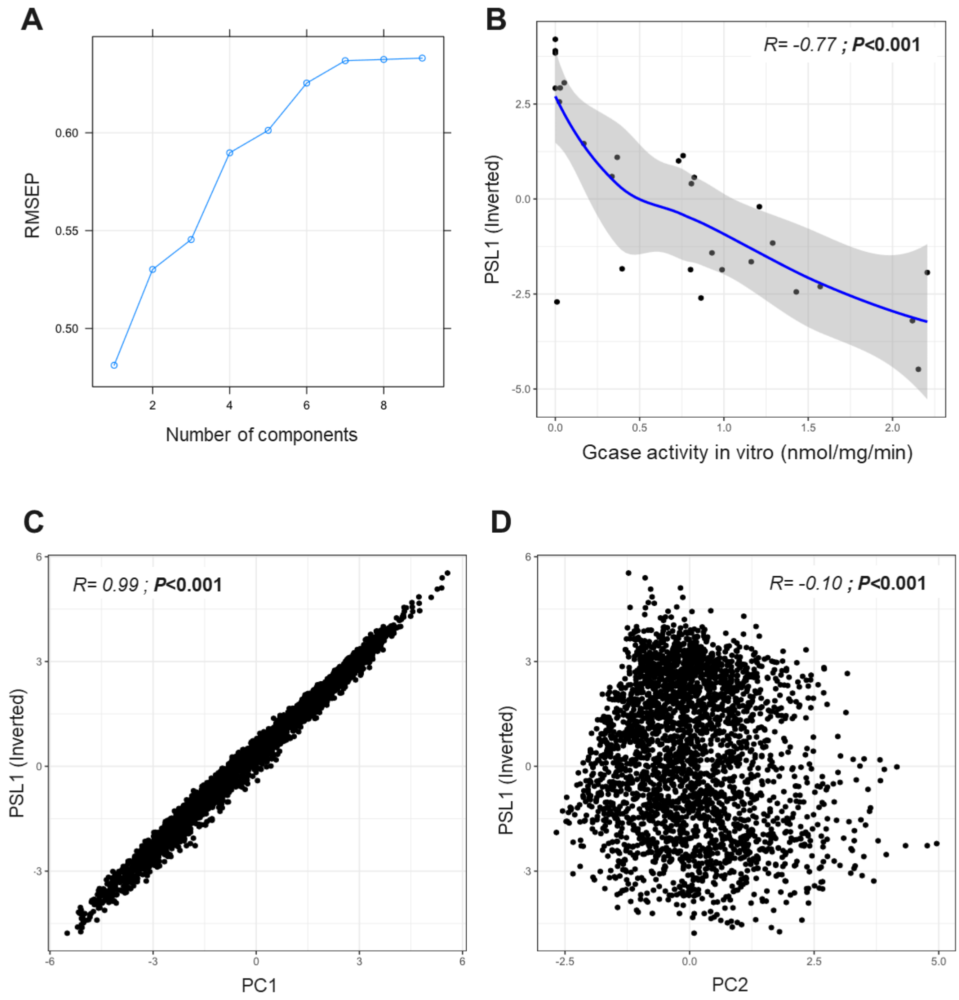

Figure S1: Partial least squares regression modeling of β-glucocerebosidase enzymatic activity and comparison with principal component axes.

A) Root mean squared error of prediction curve for the partial least squares model, showing the cross-validated prediction error across 1 to 10 components. The lowest root mean squared error of prediction was observed with a single component, indicating that partial least squares component 1 provides optimal predictive performance for enzymatic activity.
B) Scatter plot of PLS1 (inverted for interpretability) versus in vitro β-glucocerebosidase enzymatic activity (*n* = 28), with a LOESS curve and 95% confidence interval.
C) PLS1 (inverted) shows a strong linear correlation with the first principal component.
D) Scatter plot showing a weak negative correlation between PLS1 (inverted) and the second principal component.

Abbreviations: PLS – Partial Least Squares; PC1 – Principal Component 1; PC2 – Principal Component 2; PLS1 – First Component from PLS regression; RMSEP – Root Mean Squared Error of Prediction

Table S1 : List of *GBA1* missense variants retained in the analysis

GBA1 mutations	ACMG	GD classification	CADD	REVEL	PrimateAI	Alphamissense	Demask	LOF	DDG	RSA	DSA	Bfactor	PC1	PC2	PSL1 (Inverted)	Observed clinical variants
R2C	VUS		22,60	0,36	0,3296	0,1191	0,0404	0,5638	0,559	0,2263	1,66	1,27	-2,240	1,637	-2,070	
R2G	VUS		22,20	0,357	0,2937	0,1034	0,0699	0,4798	1,72	0,2263	0,91	1,27	-2,439	0,666	-2,091	
R2H	VUS		20,70	0,24	0,2974	0,0765	-0,0059	0,2881	0,929	0,2263	1,03	1,27	-3,280	0,606	-2,779	
R2L	VUS		20,50	0,307	0,3021	0,0935	0,0251	0,5902	-0,85	0,2263	1,75	1,27	-2,631	1,656	-2,449	
R2P	VUS		21,80	0,389	0,3116	0,2049	0,1766	0,6897	0,709	0,2263	0,68	1,27	-1,742	0,565	-1,484	
R2S	VUS		21,80	0,361	0,2901	0,1555	0,0168	0,4014	1,629	0,2263	0,93	1,27	-2,657	0,640	-2,256	
P3A	VUS		23,10	0,712	0,4642	0,0882	0,0292	0,5909	1,356	0,5723	0,52	0,16	-1,542	0,538	-1,233	
P3H	VUS		25,80	0,744	0,5032	0,1835	0,0761	0,7289	1,677	0,5723	0,35	0,16	-0,774	0,535	-0,528	
P3L	VUS		25,40	0,683	0,5165	0,1477	0,078	0,6712	1,244	0,5723	1,07	0,16	-0,972	1,354	-0,844	
P3R	VUS		25,60	0,755	0,5150	0,1363	0,0928	0,5951	0,442	0,5723	0,68	0,16	-0,977	0,903	-0,760	
P3S	VUS		22,70	0,713	0,4609	0,1196	0,0209	0,5571	1,303	0,5723	0,25	0,16	-1,633	0,189	-1,242	
P3T	VUS		22,70	0,727	0,4936	0,123	0,036	0,6957	1,931	0,5723	0,3	0,16	-1,253	0,322	-0,929	
C4F	VUS		25,90	0,922	0,6407	0,9557	0,4787	0,8997	2,101	0,1497	0,12	0,14	2,554	0,025	2,641	
C4G	VUS		25,50	0,876	0,6003	0,9113	0,4772	0,8457	4,112	0,1497	0,75	0,14	2,339	0,696	2,289	
C4R	VUS		25,70	0,928	0,7030	0,983	0,537	0,9004	5,295	0,1497	1,66	0,14	3,116	1,944	2,799	
C4S	VUS		25,30	0,882	0,6215	0,9816	0,4755	0,9054	4,653	0,1497	0,73	0,14	2,570	0,713	2,504	
C4W	VUS		23,70	0,741	0,6813	0,977	0,4819	0,8847	2,926	0,1497	0,01	0,14	2,186	-0,248	2,402	
C4Y	VUS		25,60	0,927	0,7203	0,98	0,488	0,9005	1,569	0,1497	0,25	0,14	2,770	0,227	2,855	
I5F	VUS		20,60	0,332	0,4163	0,0902	-0,0032	0,2052	0,122	0,3909	0,1	-0,1	-2,986	-0,912	-2,173	
I5L	VUS		19,04	0,267	0,3986	0,0713	-0,088	0,1796	-0,226	0,3909	0,07	-0,1	-3,540	-1,090	-2,625	
I5M	VUS		18,49	0,28	0,3902	0,0868	-0,0665	0,2975	-0,377	0,3909	0,07	-0,1	-3,336	-1,044	-2,482	
I5N	VUS		17,96	0,326	0,4473	0,0779	0,0624	0,1876	0,142	0,3909	1,09	-0,1	-2,918	0,261	-2,343	
I5S	VUS		16,65	0,315	0,4041	0,0796	0,0578	0,1640	1,467	0,3909	0,75	-0,1	-3,149	-0,235	-2,491	
I5T	VUS		16,58	0,303	0,4587	0,1249	-0,0051	0,1808	1,255	0,3909	0,7	-0,1	-3,135	-0,296	-2,405	
I5V	VUS		17,17	0,229	0,3661	0,0726	-0,1402	0,1165	0,778	0,3909	0,04	-0,1	-4,000	-1,268	-3,013	
P6A	VUS		20,90	0,361	0,5212	0,0813	-0,0091	0,1833	1,315	0,5157	0,52	-0,13	-2,802	-0,119	-2,099	
P6H	VUS		22,30	0,505	0,5612	0,2505	0,0479	0,4280	1,371	0,5157	0,35	-0,13	-1,657	-0,037	-1,098	
P6L	VUS		22,40	0,377	0,5850	0,1847	0,0539	0,3755	0,908	0,5157	1,07	-0,13	-1,939	0,763	-1,474	
P6R	VUS		23,20	0,515	0,5779	0,1391	0,0713	0,4781	0,893	0,5157	0,68	-0,13	-1,522	0,405	-1,070	
P6S	VUS		21,90	0,334	0,5167	0,1343	-0,0011	0,2898	1,835	0,5157	0,25	-0,13	-2,556	-0,385	-1,837	
P6T	VUS		22,00	0,385	0,5396	0,162	0,0272	0,3824	0,938	0,5157	0,3	-0,13	-2,194	-0,224	-1,546	
K7E	VUS		22,60	0,724	0,4734	0,1081	0,0996	0,5288	0,801	0,3390	1,3	0,11	-1,069	1,010	-0,873	
K7I	VUS		22,30	0,578	0,4581	0,2784	0,1249	0,6925	0,501	0,3390	2,82	0,11	-0,811	2,903	-1,018	
K7N	VUS		24,50	0,575	0,4959	0,4077	0,1726	0,6287	0,675	0,3390	1,73	0,11	-0,484	1,686	-0,441	
K7Q	VUS		20,90	0,346	0,4434	0,0835	0,0645	0,2871	0,545	0,3390	1,7	0,11	-2,432	1,146	-2,073	
K7R	VUS		17,32	0,346	0,4284	0,0666	-0,0192	0,3288	-0,093	0,3390	1,14	0,11	-2,944	0,324	-2,387	
K7T	Likely pathogenic		22,30	0,65	0,4788	0,1505	0,1034	0,6222	0,59	0,3390	2,12	0,11	-0,971	2,018	-0,979	
S8C	Likely pathogenic		22,50	0,658	0,5341	0,3178	0,0339	0,5235	0,166	0,4581	0,73	0,15	-1,231	0,596	-0,860	
S8G	VUS		21,80	0,435	0,4987	0,1238	-0,0752	0,2565	0,504	0,4581	0,02	0,15	-2,701	-0,626	-1,905	
S8I	VUS		17,20	0,511	0,5353	0,1975	-0,015	0,4748	0,472	0,4581	0,75	0,15	-2,190	0,320	-1,670	
S8N	VUS		15,53	0,282	0,5489	0,0949	-0,0937	0,1779	0,492	0,4581	0,34	0,15	-3,485	-0,510	-2,594	
S8R	VUS		20,60	0,324	0,5589	0,4646	-0,0082	0,3556	0,43	0,4581	0,93	0,15	-2,104	0,592	-1,551	
S8T	VUS		15,58	0,397	0,5278	0,1029	-0,108	0,2012	-0,201	0,4581	0,05	0,15	-3,354	-0,807	-2,443	
F9C	Likely pathogenic		27,30	0,849	0,7132	0,7614	0,2068	0,8592	2,077	0,3167	0,12	-0,2	1,597	-0,018	1,891	
F9I	Likely pathogenic		25,40	0,808	0,6850	0,5626	0,165	0,7535	1,876	0,3167	0,1	-0,2	0,831	-0,242	1,227	
F9L	VUS		21,70	0,553	0,7174	0,9134	0,1271	0,6591	1,274	0,3167	0,03	-0,2	0,289	-0,490	0,881	
F9S	Likely pathogenic		27,30	0,837	0,693	0,578	0,218	0,873	2,827	0,317	0,850	-0,200	1,455	0,812	1,563	
F9V	Likely pathogenic		25,80	0,820	0,685	0,457	0,155	0,831	2,731	0,317	0,140	-0,200	0,893	-0,193	1,252	
F9Y	Likely pathogenic		22,50	0,655	0,705	0,284	0,099	0,648	0,798	0,317	0,370	-0,200	-0,265	-0,204	0,262	
G10A	Likely pathogenic		24,10	0,729	0,628	0,269	0,169	0,734	3,875	0,692	0,290	0,070	-0,363	0,710	-0,146	

G10C	Likely pathogenic		26,00	0,666	0,656	0,640	0,310	0,818	2,598	0,692	0,750	0,070	0,572	1,529	0,563
G10D	Likely pathogenic		23,10	0,730	0,701	0,318	0,223	0,737	3,955	0,692	0,460	0,070	-0,052	0,964	0,125
G10R	VUS		25,60	0,618	0,698	0,478	0,259	0,813	3,237	0,692	0,910	0,070	0,281	1,625	0,314
G10S	Likely pathogenic		23,30	0,669	0,616	0,140	0,168	0,700	3,780	0,692	0,020	0,070	-0,779	0,275	-0,449
G10V	Likely pathogenic		24,70	0,812	0,632	0,595	0,337	0,814	5,002	0,692	0,730	0,070	0,808	1,512	0,721
Y11C	Likely pathogenic		23,90	0,673	0,618	0,198	0,017	0,609	0,581	0,285	0,250	0,050	-0,713	-0,340	-0,148
Y11D	VUS		21,70	0,447	0,613	0,168	0,051	0,398	-0,984	0,285	0,960	0,050	-1,589	0,304	-1,026
Y11F	VUS		20,90	0,394	0,558	0,100	-0,131	0,345	0,102	0,285	0,370	0,050	-2,463	-0,634	-1,623
Y11H	VUS		18,96	0,364	0,615	0,146	-0,074	0,201	-0,054	0,285	0,380	0,050	-2,555	-0,671	-1,651
Y11N	VUS		21,40	0,376	0,618	0,125	0,021	0,429	-1,831	0,285	0,820	0,050	-1,851	0,080	-1,208
Y11S	VUS		21,90	0,389	0,564	0,136	0,031	0,422	0,883	0,285	0,480	0,050	-1,806	-0,353	-1,131
S12C	Likely pathogenic		23,50	0,665	0,535	0,295	0,096	0,572	1,319	0,297	0,730	-0,380	-0,575	0,027	-0,175
S12G	VUS		17,07	0,357	0,494	0,078	-0,048	0,188	0,990	0,297	0,020	-0,380	-2,937	-1,460	-1,966
S12I	Likely pathogenic		23,40	0,750	0,543	0,416	0,127	0,490	2,566	0,297	0,750	-0,380	-0,262	0,107	0,099
S12N	VUS		17,14	0,284	0,556	0,128	-0,027	0,245	1,200	0,297	0,340	-0,380	-2,700	-1,034	-1,791
S12R	VUS		20,70	0,464	0,566	0,558	0,060	0,385	1,206	0,297	0,930	-0,380	-1,145	0,100	-0,608
S12T	VUS		19,60	0,258	0,526	0,106	-0,049	0,256	0,772	0,297	0,050	-0,380	-2,746	-1,356	-1,775
S13A	VUS		22,40	0,611	0,624	0,179	0,008	0,293	1,765	0,045	0,270	-0,430	-0,962	-1,282	-0,121
S13L	VUS		22,30	0,631	0,651	0,579	0,099	0,612	9,514	0,045	0,820	-0,430	0,629	-0,380	1,097
S13P	Likely pathogenic		26,00	0,828	0,645	0,875	0,254	0,863	7,650	0,045	0,250	-0,430	2,205	-0,607	2,506
S13T	Likely pathogenic		23,00	0,682	0,627	0,321	0,014	0,596	6,683	0,045	0,050	-0,430	0,033	-1,390	0,745
S13W	VUS		25,20	0,641	0,682	0,822	0,189	0,860	52,861	0,045	0,740	-0,430	3,555	-0,534	3,625
V14A	Likely pathogenic		22,70	0,752	0,701	0,210	0,113	0,370	0,582	0,155	0,440	-0,750	-0,209	-0,821	0,460
V14E	Likely pathogenic		24,00	0,857	0,684	0,299	0,237	0,786	-0,138	0,155	1,480	-0,750	1,103	0,773	1,230
V14G	Likely pathogenic		23,00	0,819	0,587	0,334	0,171	0,593	1,391	0,155	0,730	-0,750	0,333	-0,334	0,723
V14L	VUS		17,07	0,489	0,681	0,200	0,024	0,318	-0,811	0,155	0,110	-0,750	-1,539	-1,578	-0,569
V14M	VUS		21,00	0,615	0,710	0,125	0,028	0,351	-0,247	0,155	0,110	-0,750	-0,952	-1,407	-0,063
V15A	Likely pathogenic		24,60	0,561	0,777	0,382	0,228	0,727	1,848	0,000	0,440	-0,860	1,025	-0,988	1,633
V15E	Likely pathogenic		25,80	0,717	0,764	0,966	0,438	0,915	5,678	0,000	1,480	-0,860	3,038	0,705	3,058
V15G	Likely pathogenic		25,80	0,662	0,685	0,639	0,440	0,868	3,763	0,000	0,730	-0,860	2,186	-0,364	2,424
V15L	Pathogenic	Severe	24,10	0,862	0,763	0,842	0,239	0,924	0,673	0,000	0,110	-0,860	2,285	-1,047	2,750
V15M	Likely pathogenic		24,70	0,882	0,786	0,858	0,324	0,935	2,280	0,000	0,110	-0,860	2,722	-0,969	3,118
C16F	Likely pathogenic		25,30	0,932	0,856	0,986	0,485	0,919	22,981	0,000	0,120	-0,770	4,363	-0,886	4,538
C16G	Likely pathogenic		25,00	0,897	0,832	0,957	0,484	0,929	4,992	0,000	0,750	-0,770	3,508	-0,008	3,635
C16R	Likely pathogenic		25,10	0,936	0,889	0,994	0,544	0,926	17,649	0,000	1,660	-0,770	4,491	1,088	4,297
C16S	Likely pathogenic	Severe	24,40	0,889	0,847	0,993	0,482	0,927	4,112	0,000	0,730	-0,770	3,480	-0,031	3,630
C16W	Likely pathogenic		24,30	0,761	0,877	0,990	0,489	0,892	30,712	0,000	0,010	-0,770	4,300	-1,194	4,549
C16Y	Likely pathogenic		25,00	0,945	0,894	0,992	0,495	0,921	28,619	0,000	0,250	-0,770	4,732	-0,752	4,851
V17A	Likely pathogenic		23,80	0,797	0,762	0,200	0,139	0,574	1,933	0,161	0,440	-0,680	0,487	-0,622	1,051
V17D	Likely pathogenic		25,90	0,898	0,761	0,541	0,390	0,842	2,355	0,161	1,190	-0,680	2,276	0,728	2,323
V17F	Likely pathogenic		24,90	0,871	0,731	0,238	0,301	0,790	0,932	0,161	0,140	-0,680	1,319	-0,752	1,730
V17G	Likely pathogenic		25,80	0,894	0,668	0,371	0,350	0,690	3,019	0,161	0,730	-0,680	1,521	-0,005	1,730
V17I	VUS		21,90	0,636	0,624	0,078	0,077	0,543	0,286	0,161	0,040	-0,680	-0,708	-1,365	0,039
V17L	Likely pathogenic		22,90	0,677	0,750	0,232	0,143	0,652	-0,196	0,161	0,110	-0,680	0,215	-1,059	0,887
C18F	Likely pathogenic		25,30	0,914	0,856	0,986	0,486	0,897	20,211	0,000	0,120	-0,260	4,081	-0,585	4,249
C18G	Likely pathogenic		24,90	0,864	0,837	0,936	0,484	0,893	2,574	0,000	0,750	-0,260	3,171	0,272	3,305
C18R	Likely pathogenic		25,10	0,854	0,884	0,991	0,544	0,912	17,743	0,000	1,660	-0,260	4,201	1,334	4,004
C18S	Likely pathogenic		24,40	0,843	0,849	0,992	0,482	0,896	3,596	0,000	0,730	-0,260	3,228	0,236	3,374
C18W	Likely pathogenic		25,80	0,867	0,874	0,990	0,489	0,869	32,323	0,000	0,010	-0,260	4,530	-0,828	4,689
C18Y	Likely pathogenic		25,00	0,919	0,890	0,991	0,495	0,904	22,967	0,000	0,250	-0,260	4,311	-0,432	4,439
N19D	Likely pathogenic		23,40	0,696	0,764	0,118	0,158	0,517	0,643	0,287	0,140	0,050	-0,253	-0,391	0,348
N19H	Likely pathogenic		24,70	0,789	0,736	0,389	0,249	0,763	1,205	0,287	0,440	0,050	0,880	0,255	1,182
N19I	Likely pathogenic		25,20	0,711	0,730	0,894	0,227	0,876	3,919	0,287	1,090	0,050	1,585	1,172	1,674
N19K	Likely pathogenic		23,80	0,745	0,787	0,747	0,248	0,787	0,926	0,287	1,730	0,050	1,366	1,919	1,369

N19S	VUS		22,70	0,641	0,679	0,093	0,122	0,461	2,622	0,287	0,340	0,050	-0,711	-0,290	-0,134	
N19T	Likely pathogenic		24,60	0,665	0,719	0,325	0,181	0,693	4,311	0,287	0,390	0,050	0,380	0,017	0,791	
N19Y	Likely pathogenic		25,30	0,854	0,763	0,694	0,285	0,884	1,609	0,287	0,820	0,050	1,758	0,925	1,859	
A20D	Likely pathogenic		25,60	0,702	0,772	0,276	0,173	0,828	0,721	0,225	0,750	0,030	0,744	0,443	1,072	
A20G	VUS		23,40	0,479	0,689	0,138	0,079	0,408	0,590	0,225	0,290	0,030	-1,075	-0,559	-0,345	
A20P	Likely pathogenic		25,60	0,820	0,749	0,487	0,216	0,850	-0,548	0,225	0,520	0,030	1,215	0,314	1,501	
A20S	VUS		21,90	0,293	0,668	0,083	0,028	0,184	-0,026	0,225	0,270	0,030	-2,126	-0,840	-1,197	
A20T	VUS		22,60	0,483	0,700	0,096	0,052	0,523	1,475	0,225	0,220	0,030	-1,008	-0,656	-0,279	
A20V	VUS		23,50	0,465	0,720	0,185	0,054	0,595	1,015	0,225	0,440	0,030	-0,722	-0,307	-0,070	
T21A	VUS		20,50	0,598	0,659	0,114	0,122	0,553	0,414	0,657	0,220	0,080	-1,446	0,301	-0,980	
T21I	Likely pathogenic		25,30	0,825	0,755	0,474	0,227	0,799	-0,146	0,657	0,700	0,080	0,581	1,424	0,669	
T21K	Likely pathogenic		23,60	0,740	0,739	0,273	0,209	0,739	-0,549	0,657	2,120	0,080	-0,005	2,979	-0,173	
T21P	Likely pathogenic		23,50	0,811	0,651	0,361	0,366	0,743	1,044	0,657	0,300	0,080	0,324	0,850	0,419	
T21R	Likely pathogenic		24,00	0,799	0,725	0,183	0,209	0,651	-0,748	0,657	0,980	0,080	-0,219	1,566	-0,103	
T21S	VUS		18,76	0,358	0,667	0,089	0,071	0,278	0,048	0,657	0,050	0,080	-2,592	-0,196	-1,840	
Y22C	Likely pathogenic		27,70	0,909	0,785	0,337	0,304	0,884	1,839	0,232	0,250	0,080	1,771	0,141	2,012	
Y22D	Likely pathogenic		24,00	0,884	0,777	0,393	0,280	0,832	2,581	0,232	0,960	0,080	1,453	0,869	1,582	
Y22F	Likely pathogenic		25,40	0,780	0,727	0,116	0,084	0,525	0,630	0,232	0,370	0,080	-0,121	-0,161	0,422	
Y22H	Likely pathogenic		22,80	0,690	0,782	0,187	0,148	0,545	1,986	0,232	0,380	0,080	-0,028	-0,193	0,533	
Y22N	Likely pathogenic		23,90	0,863	0,778	0,295	0,249	0,882	2,259	0,232	0,820	0,080	1,277	0,660	1,459	
Y22S	Likely pathogenic		26,80	0,873	0,740	0,181	0,256	0,603	2,166	0,232	0,480	0,080	0,855	0,191	1,182	
C23F	Likely pathogenic		25,40	0,920	0,753	0,974	0,483	0,911	-0,481	0,012	0,120	0,320	2,877	-0,089	3,063	
C23G	Likely pathogenic		26,70	0,909	0,709	0,894	0,482	0,899	2,154	0,012	0,750	0,320	2,888	0,645	2,891	
C23R	Likely pathogenic		27,00	0,942	0,807	0,975	0,542	0,904	2,578	0,012	1,660	0,320	3,530	1,876	3,293	
C23S	Likely pathogenic		24,90	0,905	0,737	0,979	0,480	0,913	1,685	0,012	0,730	0,320	2,911	0,612	2,940	
C23W	Likely pathogenic	Severe	24,60	0,947	0,788	0,983	0,486	0,891	2,623	0,012	0,010	0,320	3,061	-0,246	3,272	
C23Y	Likely pathogenic		25,20	0,931	0,818	0,986	0,493	0,907	0,193	0,012	0,250	0,320	3,105	0,098	3,276	
D24A	Likely pathogenic		26,30	0,947	0,699	0,878	0,332	0,912	1,580	0,036	0,750	0,300	2,499	0,609	2,571	
D24E	Likely pathogenic		24,00	0,815	0,724	0,885	0,277	0,916	-0,693	0,036	0,290	0,300	1,899	-0,081	2,207	
D24G	Likely pathogenic		26,30	0,884	0,704	0,808	0,266	0,905	3,022	0,036	0,460	0,300	2,182	0,154	2,391	
D24H	Likely pathogenic		25,10	0,939	0,760	0,932	0,342	0,919	7,318	0,036	0,580	0,300	2,859	0,363	2,967	
D24N	Likely pathogenic	Severe	25,20	0,779	0,742	0,759	0,299	0,929	1,278	0,036	0,140	0,300	1,960	-0,278	2,298	Yes
D24V	Likely pathogenic		26,10	0,949	0,696	0,921	0,379	0,918	2,141	0,036	1,190	0,300	2,707	1,176	2,634	
D24Y	Likely pathogenic		25,10	0,942	0,747	0,884	0,377	0,919	6,333	0,036	0,960	0,300	2,855	0,838	2,850	
S25A	VUS		22,40	0,465	0,554	0,095	-0,032	0,341	0,044	0,123	0,270	0,320	-1,848	-0,806	-1,042	
S25C	Likely pathogenic		23,40	0,681	0,576	0,204	0,095	0,628	-0,218	0,123	0,730	0,320	-0,440	0,102	-0,017	
S25F	Likely pathogenic		22,50	0,757	0,590	0,182	0,035	0,571	-2,047	0,123	0,850	0,320	-0,644	0,223	-0,198	
S25P	Likely pathogenic		25,30	0,819	0,567	0,424	0,130	0,689	3,717	0,123	0,250	0,320	0,481	-0,296	0,867	
S25T	VUS		19,84	0,418	0,562	0,076	-0,071	0,199	-0,527	0,123	0,050	0,320	-2,464	-1,236	-1,483	
S25Y	VUS		22,00	0,602	0,610	0,163	0,049	0,507	-0,091	0,123	0,480	0,320	-0,956	-0,347	-0,342	
F26C	VUS		23,60	0,457	0,538	0,207	0,168	0,413	2,756	0,083	0,120	0,430	-0,973	-0,837	-0,309	
F26I	VUS		14,87	0,285	0,490	0,067	0,043	0,205	1,624	0,083	0,100	0,430	-2,841	-1,381	-1,890	
F26L	VUS		12,68	0,290	0,481	0,130	0,002	0,140	0,296	0,083	0,030	0,430	-3,196	-1,544	-2,164	
F26S	VUS		22,40	0,393	0,494	0,151	0,111	0,321	2,865	0,083	0,850	0,430	-1,561	-0,108	-0,988	
F26V	VUS		14,75	0,288	0,459	0,066	0,036	0,164	2,529	0,083	0,140	0,430	-2,957	-1,372	-2,015	
F26Y	VUS		21,80	0,428	0,557	0,172	0,006	0,310	1,502	0,083	0,370	0,430	-1,724	-0,713	-0,941	
D27A	VUS		22,40	0,470	0,497	0,094	0,005	0,519	0,444	0,415	0,750	0,620	-2,030	0,608	-1,591	
D27E	VUS		13,88	0,310	0,516	0,119	-0,064	0,204	-0,520	0,415	0,290	0,620	-3,568	-0,393	-2,719	
D27G	VUS		20,90	0,360	0,501	0,098	0,000	0,369	0,774	0,415	0,460	0,620	-2,572	0,104	-1,950	
D27H	Likely pathogenic		22,90	0,694	0,557	0,127	0,026	0,630	0,677	0,415	0,580	0,620	-1,175	0,607	-0,816	
D27N	VUS		21,50	0,494	0,540	0,095	-0,029	0,511	0,137	0,415	0,140	0,620	-2,093	-0,152	-1,464	
D27V	VUS		22,60	0,627	0,485	0,122	0,057	0,628	0,642	0,415	1,190	0,620	-1,386	1,297	-1,196	
D27Y	Likely pathogenic		23,00	0,783	0,544	0,127	0,067	0,688	0,126	0,415	0,960	0,620	-0,840	1,159	-0,658	
P28A	VUS		16,62	0,470	0,497	0,059	-0,010	0,481	1,133	0,415	0,520	0,640	-2,560	0,142	-2,000	

P28H	Likely pathogenic	23,90	0,717	0,534	0,092	0,031	0,759	1,457	0,415	0,350	0,640	-0,940	0,398	-0,605	
P28L	Likely pathogenic	22,90	0,684	0,562	0,076	0,040	0,724	0,887	0,415	1,070	0,640	-1,028	1,238	-0,824	
P28R	VUS	21,60	0,523	0,555	0,079	0,049	0,496	1,215	0,415	0,680	0,640	-1,770	0,568	-1,327	
P28S	VUS	15,13	0,458	0,489	0,071	-0,024	0,387	1,628	0,415	0,250	0,640	-2,867	-0,279	-2,189	
P28T	VUS	14,75	0,519	0,517	0,068	0,000	0,680	1,629	0,415	0,300	0,640	-2,218	-0,075	-1,681	
P29A	VUS	11,58	0,241	0,476	0,074	-0,026	0,242	1,742	0,214	0,520	1,030	-3,555	-0,397	-2,734	
P29L	VUS	6,41	0,252	0,445	0,055	0,007	0,251	0,892	0,214	1,070	1,030	-3,895	0,147	-3,203	
P29Q	VUS	14,18	0,352	0,456	0,093	0,005	0,323	2,030	0,214	0,120	1,030	-3,012	-0,719	-2,217	
P29R	VUS	14,46	0,344	0,505	0,085	0,048	0,322	3,206	0,214	0,680	1,030	-2,707	0,001	-2,059	
P29S	VUS	13,27	0,248	0,462	0,103	-0,020	0,232	2,312	0,214	0,250	1,030	-3,423	-0,679	-2,564	
P29T	VUS	12,25	0,233	0,508	0,098	-0,010	0,205	2,135	0,214	0,300	1,030	-3,437	-0,638	-2,552	
T30A	VUS	8,44	0,290	0,453	0,058	-0,087	0,100	0,628	0,430	0,220	1,400	-4,553	-0,258	-3,676	
T30I	VUS	16,04	0,318	0,524	0,074	-0,054	0,191	-0,289	0,430	0,700	1,400	-3,565	0,645	-2,894	
T30N	VUS	14,79	0,288	0,492	0,083	-0,029	0,141	-0,039	0,430	0,390	1,400	-3,803	0,200	-3,049	
T30P	VUS	10,98	0,293	0,458	0,069	0,080	0,201	0,204	0,430	0,300	1,400	-3,804	0,048	-3,104	
T30S	VUS	13,19	0,324	0,453	0,074	-0,130	0,126	0,461	0,430	0,050	1,400	-4,220	-0,326	-3,336	
F31C	VUS	20,60	0,389	0,504	0,181	0,155	0,398	1,193	0,154	0,120	1,650	-1,881	-0,125	-1,275	
F31I	VUS	9,20	0,182	0,475	0,064	0,042	0,138	0,967	0,154	0,100	1,650	-3,945	-0,764	-3,003	
F31L	VUS	16,69	0,212	0,472	0,236	-0,014	0,187	-0,536	0,154	0,030	1,650	-3,305	-0,580	-2,405	
F31S	VUS	19,12	0,286	0,479	0,094	0,079	0,219	2,112	0,154	0,850	1,650	-2,695	0,529	-2,113	
F31V	VUS	9,16	0,212	0,445	0,066	0,034	0,116	1,680	0,154	0,140	1,650	-3,981	-0,731	-3,064	
F31Y	VUS	16,88	0,335	0,502	0,109	0,001	0,260	0,956	0,154	0,370	1,650	-2,896	-0,101	-2,149	
P32A	VUS	21,60	0,629	0,648	0,125	0,044	0,632	2,277	0,050	0,520	1,060	-0,660	0,008	-0,125	
P32H	Likely pathogenic	24,00	0,743	0,686	0,262	0,100	0,788	5,132	0,050	0,350	1,060	0,440	0,039	0,852	
P32L	VUS	21,50	0,614	0,698	0,127	0,081	0,548	2,575	0,050	1,070	1,060	-0,556	0,681	-0,121	
P32R	Likely pathogenic	24,00	0,781	0,697	0,231	0,126	0,861	2,947	0,050	0,680	1,060	0,610	0,514	0,903	
P32S	Likely pathogenic	23,10	0,675	0,649	0,176	0,049	0,659	2,213	0,050	0,250	1,060	-0,377	-0,229	0,178	Yes
P32T	VUS	21,90	0,615	0,679	0,160	0,057	0,626	4,571	0,050	0,300	1,060	-0,453	-0,251	0,130	
A33D	VUS	18,54	0,263	0,498	0,087	0,032	0,268	-0,018	0,558	0,750	0,800	-3,260	0,727	-2,700	
A33G	VUS	18,41	0,322	0,437	0,094	-0,052	0,264	0,148	0,558	0,290	0,800	-3,528	0,119	-2,852	
A33P	VUS	13,66	0,214	0,472	0,070	0,064	0,313	-1,704	0,558	0,520	0,800	-3,722	0,317	-3,086	
A33S	VUS	13,03	0,307	0,420	0,073	-0,085	0,242	0,320	0,558	0,270	0,800	-4,106	-0,103	-3,356	
A33T	VUS	14,10	0,232	0,439	0,072	-0,077	0,269	0,566	0,558	0,220	0,800	-4,064	-0,152	-3,289	
A33V	VUS	19,16	0,306	0,455	0,080	-0,072	0,513	0,437	0,558	0,440	0,800	-3,132	0,390	-2,564	
L34F	VUS	7,64	0,224	0,375	0,085	-0,053	0,261	-0,110	0,478	0,030	0,600	-4,516	-0,854	-3,633	
L34H	VUS	5,43	0,277	0,391	0,090	0,056	0,301	0,295	0,478	0,720	0,600	-4,141	0,032	-3,506	
L34I	VUS	7,62	0,184	0,379	0,080	-0,119	0,236	0,097	0,478	0,070	0,600	-4,777	-0,873	-3,837	
L34P	VUS	6,67	0,193	0,407	0,045	0,076	0,235	-0,729	0,478	1,070	0,600	-4,293	0,443	-3,693	
L34R	VUS	5,76	0,245	0,401	0,063	0,082	0,297	0,189	0,478	1,750	0,600	-4,055	1,295	-3,668	
L34V	VUS	6,74	0,204	0,362	0,069	-0,094	0,223	0,416	0,478	0,110	0,600	-4,795	-0,842	-3,884	
G35A	Likely pathogenic	23,60	0,755	0,716	0,232	0,184	0,551	2,685	0,298	0,290	0,210	0,047	-0,035	0,503	
G35C	Likely pathogenic	23,00	0,759	0,744	0,431	0,322	0,879	2,803	0,298	0,750	0,210	1,156	0,768	1,296	
G35D	Likely pathogenic	23,00	0,807	0,785	0,164	0,235	0,858	2,900	0,298	0,460	0,210	0,792	0,321	1,082	
G35R	Likely pathogenic	22,70	0,766	0,776	0,274	0,271	0,890	1,362	0,298	0,910	0,210	0,898	0,914	1,059	
G35S	VUS	22,40	0,583	0,704	0,156	0,183	0,657	2,767	0,298	0,020	0,210	-0,339	-0,476	0,221	
G35V	Likely pathogenic	24,30	0,845	0,719	0,433	0,349	0,843	4,567	0,298	0,730	0,210	1,438	0,801	1,515	
T36A	VUS	18,32	0,273	0,552	0,074	-0,072	0,335	0,271	0,233	0,220	-0,050	-2,705	-1,097	-1,769	
T36I	VUS	18,22	0,263	0,624	0,118	-0,040	0,536	-0,892	0,233	0,700	-0,050	-2,145	-0,380	-1,380	
T36N	VUS	16,28	0,197	0,592	0,074	-0,023	0,186	0,360	0,233	0,390	-0,050	-2,986	-0,992	-2,005	
T36P	VUS	20,50	0,327	0,554	0,212	0,103	0,551	2,752	0,233	0,300	-0,050	-1,443	-0,722	-0,774	
T36S	VUS	16,75	0,197	0,550	0,071	-0,119	0,220	1,062	0,233	0,050	-0,050	-3,233	-1,460	-2,148	
F37C	Likely pathogenic	26,60	0,790	0,724	0,658	0,224	0,839	3,067	0,046	0,120	-0,520	1,832	-0,836	2,283	
F37I	Likely pathogenic	24,00	0,707	0,701	0,404	0,112	0,789	2,459	0,046	0,100	-0,520	0,786	-1,131	1,394	
F37L	Likely pathogenic	22,70	0,536	0,733	0,943	0,072	0,617	2,668	0,046	0,030	-0,520	0,694	-1,251	1,458	

F37S	Likely pathogenic		24,60	0,806	0,703	0,766	0,167	0,793	4,912	0,046	0,850	-0,520	1,717	-0,031	2,025	
F37V	Likely pathogenic	Mild	22,70	0,775	0,692	0,356	0,101	0,730	0,805	0,046	0,140	-0,520	0,571	-1,114	1,190	
F37Y	Likely pathogenic		22,10	0,477	0,720	0,196	0,043	0,379	0,494	0,046	0,370	-0,520	-0,805	-1,181	0,067	
S38C	VUS		23,40	0,633	0,562	0,128	0,050	0,678	-0,848	0,000	0,730	-0,570	-0,361	-0,717	0,205	
S38G	VUS		21,80	0,351	0,521	0,121	-0,076	0,353	1,247	0,000	0,020	-0,570	-1,870	-1,985	-0,842	
S38I	VUS		18,49	0,551	0,569	0,135	-0,012	0,507	-0,787	0,000	0,750	-0,570	-1,237	-0,958	-0,504	
S38N	VUS		20,10	0,476	0,593	0,199	-0,060	0,549	0,909	0,000	0,340	-0,570	-1,151	-1,449	-0,291	
S38R	VUS		22,20	0,570	0,607	0,360	0,010	0,718	3,043	0,000	0,930	-0,570	-0,066	-0,493	0,476	
S38T	VUS		19,42	0,392	0,559	0,097	-0,099	0,340	-0,192	0,000	0,050	-0,570	-2,025	-1,991	-0,958	
R39C	VUS		24,30	0,554	0,598	0,123	0,036	0,595	2,243	0,124	1,660	-0,750	-0,464	0,507	-0,098	Yes
R39G	VUS		22,30	0,503	0,516	0,259	0,063	0,581	4,687	0,124	0,910	-0,750	-0,657	-0,495	-0,143	
R39H	Likely pathogenic		21,80	0,661	0,527	0,092	-0,016	0,838	6,089	0,124	1,030	-0,750	-0,288	-0,294	0,102	
R39L	VUS		20,40	0,203	0,541	0,152	0,005	0,341	-0,348	0,124	1,750	-0,750	-2,063	0,238	-1,453	
R39P	VUS		22,60	0,587	0,555	0,876	0,237	0,717	6,045	0,124	0,680	-0,750	0,957	-0,401	1,301	
R39S	VUS		21,50	0,306	0,513	0,397	-0,003	0,377	4,065	0,124	0,930	-0,750	-1,428	-0,652	-0,729	
Y40C	Likely pathogenic		27,90	0,723	0,746	0,402	0,341	0,899	2,193	0,027	0,250	-0,890	2,012	-0,900	2,420	
Y40D	Likely pathogenic		25,20	0,826	0,736	0,930	0,317	0,872	3,152	0,027	0,960	-0,890	2,570	0,061	2,765	
Y40F	VUS		22,70	0,476	0,677	0,092	0,113	0,472	-0,862	0,027	0,370	-0,890	-0,632	-1,384	0,189	
Y40H	Likely pathogenic		25,00	0,825	0,745	0,549	0,278	0,828	1,544	0,027	0,380	-0,890	1,886	-0,783	2,303	
Y40N	Likely pathogenic		25,10	0,791	0,742	0,788	0,363	0,931	2,112	0,027	0,820	-0,890	2,500	-0,114	2,711	
Y40S	Likely pathogenic		27,10	0,735	0,692	0,719	0,375	0,859	3,567	0,027	0,480	-0,890	2,295	-0,572	2,589	
E41A	Likely pathogenic		22,80	0,449	0,615	0,317	0,004	0,551	1,291	0,013	1,040	-0,980	-0,544	-0,665	0,120	
E41D	Likely pathogenic		21,60	0,314	0,612	0,675	0,049	0,446	0,783	0,013	0,290	-0,980	-0,622	-1,587	0,273	
E41G	Likely pathogenic		24,40	0,558	0,575	0,514	0,041	0,639	2,878	0,013	0,750	-0,980	0,163	-0,843	0,747	
E41K	Likely pathogenic	Severe	22,70	0,590	0,684	0,423	0,021	0,716	2,037	0,013	1,300	-0,980	0,336	-0,162	0,830	
E41Q	Likely pathogenic		22,40	0,277	0,599	0,203	-0,029	0,306	0,703	0,013	0,400	-0,980	-1,578	-1,680	-0,575	
E41V	Likely pathogenic		23,00	0,374	0,596	0,349	0,001	0,508	-1,086	0,013	1,480	-0,980	-0,817	-0,154	-0,213	
S42C	Likely pathogenic		28,10	0,789	0,684	0,850	0,268	0,761	-0,004	0,000	0,730	-1,150	2,150	-0,445	2,493	
S42G	Likely pathogenic		27,20	0,772	0,640	0,570	0,226	0,754	0,959	0,000	0,020	-1,150	1,518	-1,478	2,071	
S42I	Likely pathogenic		25,00	0,791	0,693	0,950	0,298	0,826	2,532	0,000	0,750	-1,150	2,342	-0,455	2,644	
S42N	Likely pathogenic		24,30	0,706	0,707	0,841	0,240	0,821	2,453	0,000	0,340	-1,150	1,864	-1,077	2,356	
S42R	Likely pathogenic		27,90	0,774	0,713	0,996	0,311	0,863	15,001	0,000	0,930	-1,150	3,225	-0,225	3,381	
S42T	VUS		22,70	0,492	0,677	0,102	0,109	0,339	-0,036	0,000	0,050	-1,150	-0,701	-2,024	0,267	
T43A	Likely pathogenic		22,90	0,364	0,656	0,150	0,099	0,325	1,356	0,035	0,220	-0,930	-0,992	-1,691	-0,056	
T43I	Likely pathogenic	Mild	25,70	0,606	0,741	0,744	0,193	0,518	0,292	0,035	0,700	-0,930	1,030	-0,574	1,606	
T43K	Likely pathogenic		25,60	0,508	0,721	0,580	0,250	0,538	0,150	0,035	2,120	-0,930	0,872	1,094	1,103	
T43P	Likely pathogenic		25,10	0,639	0,652	0,726	0,331	0,596	4,759	0,035	0,300	-0,930	1,421	-1,039	1,910	
T43R	Likely pathogenic		25,20	0,399	0,712	0,366	0,175	0,542	0,632	0,035	0,980	-0,930	0,139	-0,474	0,745	
T43S	Likely pathogenic		19,52	0,333	0,654	0,072	0,022	0,175	0,782	0,035	0,050	-0,930	-1,825	-2,121	-0,699	
R44C	VUS		24,70	0,362	0,671	0,179	0,192	0,561	0,692	0,350	1,660	-0,490	-0,668	1,169	-0,383	
R44G	VUS		22,60	0,354	0,604	0,195	0,151	0,391	1,585	0,350	0,910	-0,490	-1,343	0,079	-0,804	
R44H	VUS		22,70	0,362	0,617	0,115	0,153	0,489	1,395	0,350	1,030	-0,490	-1,226	0,251	-0,745	
R44L	VUS		21,80	0,324	0,624	0,177	0,095	0,488	-0,627	0,350	1,750	-0,490	-1,445	1,093	-1,071	
R44P	VUS		22,80	0,368	0,633	0,538	0,257	0,532	3,199	0,350	0,680	-0,490	-0,339	0,014	0,111	
R44S	VUS		21,20	0,389	0,607	0,349	0,103	0,292	1,049	0,350	0,930	-0,490	-1,482	0,071	-0,891	
S45C	VUS		23,40	0,552	0,679	0,187	0,036	0,529	0,308	0,716	0,730	-0,480	-1,380	0,754	-0,959	
S45G	VUS		20,30	0,402	0,645	0,072	-0,023	0,354	0,558	0,716	0,020	-0,480	-2,537	-0,416	-1,769	
S45I	VUS		24,70	0,637	0,685	0,183	0,067	0,592	0,744	0,716	0,750	-0,480	-0,929	0,893	-0,598	
S45N	VUS		21,60	0,442	0,700	0,086	-0,012	0,365	0,573	0,716	0,340	-0,480	-2,156	0,067	-1,479	
S45R	VUS		15,32	0,427	0,707	0,199	0,061	0,356	-0,025	0,716	0,930	-0,480	-2,303	0,684	-1,758	
S45T	VUS		21,60	0,450	0,678	0,093	-0,043	0,355	0,371	0,716	0,050	-0,480	-2,302	-0,308	-1,544	
G46A	Likely pathogenic		24,00	0,784	0,713	0,537	0,254	0,821	2,422	0,212	0,290	-0,530	1,286	-0,394	1,652	
G46E	Pathogenic	Mild	24,30	0,868	0,769	0,706	0,314	0,899	2,171	0,212	0,750	-0,530	2,069	0,351	2,236	
G46R	VUS		23,90	0,837	0,780	0,637	0,334	0,852	2,146	0,212	0,910	-0,530	1,921	0,500	2,079	

G46V	Likely pathogenic		24,30	0,850	0,719	0,857	0,417	0,908	3,616	0,212	0,730	-0,530	2,402	0,389	2,475	
G46W	Likely pathogenic		24,40	0,850	0,768	0,853	0,428	0,890	3,732	0,212	0,760	-0,530	2,526	0,446	2,610	
R47G	VUS		21,60	0,369	0,609	0,157	0,095	0,284	1,002	0,380	0,910	0,510	-1,972	0,625	-1,433	
R47L	VUS		22,20	0,355	0,630	0,154	0,036	0,371	-0,693	0,380	1,750	0,510	-1,927	1,681	-1,569	
R47P	VUS		22,70	0,449	0,644	0,735	0,275	0,595	0,418	0,380	0,680	0,510	-0,166	0,805	0,142	
R47Q	VUS		22,30	0,288	0,562	0,084	0,015	0,379	0,529	0,380	0,560	0,510	-2,364	0,129	-1,712	
R48G	Likely pathogenic		24,20	0,696	0,696	0,896	0,356	0,930	5,110	0,047	0,910	-0,770	2,324	0,019	2,501	
R48L	Likely pathogenic		24,50	0,844	0,720	0,828	0,323	0,924	2,883	0,047	1,750	-0,770	2,490	1,116	2,453	
R48P	Likely pathogenic		24,80	0,847	0,732	0,959	0,463	0,933	3,253	0,047	0,680	-0,770	2,975	-0,062	3,100	
R48Q	Likely pathogenic		24,60	0,789	0,653	0,533	0,363	0,938	3,274	0,047	0,560	-0,770	1,953	-0,452	2,186	
R48W	Pathogenic	Mild	25,10	0,712	0,762	0,607	0,339	0,928	3,398	0,047	1,670	-0,770	2,198	0,929	2,247	Yes
E50A	VUS		23,00	0,554	0,694	0,143	-0,014	0,617	0,142	0,404	1,040	0,200	-1,097	0,875	-0,671	
E50D	VUS		16,82	0,547	0,695	0,221	-0,053	0,554	-0,784	0,404	0,290	0,200	-1,738	-0,230	-1,031	
E50G	Likely pathogenic		26,80	0,646	0,661	0,218	0,024	0,851	0,895	0,404	0,750	0,200	-0,202	0,774	0,097	
E50K	VUS		22,50	0,565	0,760	0,255	0,006	0,723	-0,464	0,404	1,300	0,200	-0,630	1,296	-0,295	
E50Q	VUS		22,00	0,498	0,685	0,123	-0,053	0,630	-0,378	0,404	0,400	0,200	-1,460	0,015	-0,829	
E50V	VUS		23,20	0,489	0,679	0,200	-0,001	0,656	-0,329	0,404	1,480	0,200	-1,077	1,421	-0,765	
L51M	VUS		21,40	0,333	0,532	0,118	-0,061	0,313	-0,233	0,388	0,000	-0,550	-2,529	-1,215	-1,618	
L51P	Likely pathogenic		18,78	0,656	0,612	0,131	0,122	0,385	0,704	0,388	1,070	-0,550	-1,230	0,334	-0,794	
L51Q	VUS		16,99	0,373	0,544	0,081	0,061	0,243	1,096	0,388	1,190	-0,550	-2,445	0,163	-1,853	
L51R	VUS		15,72	0,277	0,588	0,062	0,105	0,224	1,113	0,388	1,750	-0,550	-2,513	0,796	-2,010	
L51V	VUS		20,30	0,343	0,508	0,089	-0,052	0,336	1,482	0,388	0,110	-0,550	-2,541	-1,125	-1,683	
S52C	Likely pathogenic		23,60	0,810	0,706	0,207	0,022	0,814	0,497	0,245	0,730	-0,480	0,254	0,030	0,666	
S52G	VUS		22,00	0,522	0,673	0,113	-0,040	0,462	1,206	0,245	0,020	-0,480	-1,285	-1,226	-0,421	
S52I	Likely pathogenic		22,80	0,764	0,719	0,190	0,046	0,847	0,425	0,245	0,750	-0,480	0,229	0,035	0,640	
S52N	VUS		22,50	0,619	0,727	0,188	-0,016	0,714	0,943	0,245	0,340	-0,480	-0,409	-0,628	0,249	
S52R	Likely pathogenic		23,20	0,732	0,732	0,367	0,047	0,583	0,293	0,245	0,930	-0,480	0,041	0,218	0,508	
S52T	VUS		17,34	0,598	0,699	0,086	-0,063	0,555	-0,001	0,245	0,050	-0,480	-1,400	-1,249	-0,536	
M53I	VUS		0,73	0,199	0,411	0,105	-0,107	0,121	1,063	0,496	0,070	-0,100	-5,114	-1,445	-4,040	
M53K	VUS		7,85	0,308	0,428	0,068	0,042	0,256	0,617	0,496	2,890	-0,100	-3,657	2,383	-3,495	
M53L	VUS		8,45	0,337	0,394	0,074	-0,141	0,111	-0,118	0,496	0,000	-0,100	-4,532	-1,276	-3,529	
M53R	VUS		8,17	0,266	0,423	0,061	0,072	0,253	0,176	0,496	1,750	-0,100	-3,769	0,992	-3,334	
M53T	VUS		5,78	0,227	0,415	0,058	-0,057	0,166	1,375	0,496	0,770	-0,100	-4,497	-0,407	-3,688	
M53V	VUS		8,13	0,309	0,380	0,059	-0,078	0,111	1,243	0,496	0,110	-0,100	-4,446	-1,151	-3,508	
G54A	VUS		17,72	0,547	0,660	0,127	0,082	0,656	1,434	0,115	0,290	-0,100	-0,842	-0,918	-0,152	
G54E	VUS		21,70	0,651	0,707	0,183	0,151	0,870	1,883	0,115	0,750	-0,100	0,347	-0,050	0,747	
G54R	VUS		21,70	0,654	0,716	0,347	0,177	0,877	1,144	0,115	0,910	-0,100	0,613	0,221	0,950	
G54V	VUS		19,24	0,583	0,663	0,204	0,177	0,804	3,705	0,115	0,730	-0,100	0,003	-0,212	0,430	
G54W	VUS		23,00	0,710	0,707	0,398	0,271	0,872	1,917	0,115	0,760	-0,100	1,081	0,156	1,355	
P55A	VUS		0,23	0,261	0,410	0,055	-0,103	0,129	0,641	0,541	0,520	-0,020	-5,132	-0,746	-4,211	
P55H	VUS		6,75	0,244	0,427	0,064	-0,062	0,216	1,311	0,541	0,350	-0,020	-4,410	-0,724	-3,546	
P55L	VUS		6,57	0,310	0,419	0,068	-0,049	0,172	0,583	0,541	1,070	-0,020	-4,325	0,179	-3,652	
P55R	VUS		4,52	0,279	0,433	0,056	-0,034	0,183	0,545	0,541	0,680	-0,020	-4,484	-0,355	-3,695	
P55S	VUS		0,47	0,255	0,406	0,064	-0,112	0,173	0,845	0,541	0,250	-0,020	-5,092	-1,063	-4,119	
P55T	VUS		0,58	0,218	0,420	0,053	-0,096	0,111	1,321	0,541	0,300	-0,020	-5,167	-1,032	-4,176	
I56F	VUS		4,08	0,329	0,517	0,097	0,031	0,221	8,320	0,096	0,100	0,510	-3,187	-1,624	-2,205	
I56L	VUS		6,69	0,309	0,521	0,093	-0,036	0,268	-0,177	0,096	0,070	0,510	-3,476	-1,547	-2,426	
I56M	VUS		20,50	0,322	0,502	0,123	-0,017	0,318	0,134	0,096	0,070	0,510	-2,359	-1,131	-1,464	
I56N	VUS		20,20	0,524	0,558	0,565	0,131	0,684	3,850	0,096	1,090	0,510	-0,243	0,535	0,075	
I56S	VUS		20,60	0,430	0,527	0,494	0,135	0,597	4,826	0,096	0,750	0,510	-0,653	0,015	-0,203	
I56T	VUS		18,29	0,417	0,574	0,503	0,048	0,560	3,296	0,096	0,700	0,510	-1,048	-0,143	-0,468	
I56V	VUS		4,65	0,188	0,478	0,078	-0,078	0,164	1,168	0,096	0,040	0,510	-4,170	-1,792	-3,011	
Q57E	VUS		14,14	0,430	0,478	0,079	-0,084	0,367	0,194	0,258	0,400	1,610	-3,206	0,072	-2,510	
Q57H	VUS		21,90	0,572	0,494	0,116	-0,081	0,683	0,500	0,258	0,470	1,610	-1,814	0,568	-1,365	

Q57K	VUS		14,70	0,453	0,501	0,072	-0,053	0,559	-0,214	0,258	1,700	1,610	-2,634	1,782	-2,344
Q57L	VUS		21,50	0,411	0,473	0,079	-0,067	0,558	-0,325	0,258	1,190	1,610	-2,374	1,310	-2,000
Q57P	VUS		21,30	0,514	0,483	0,114	0,096	0,780	1,104	0,258	0,120	1,610	-1,427	0,216	-1,019
Q57R	VUS		17,69	0,359	0,497	0,056	-0,059	0,289	-0,390	0,258	0,560	1,610	-3,139	0,324	-2,456
A58D	VUS		16,96	0,388	0,556	0,098	-0,012	0,478	0,278	0,628	0,750	1,470	-2,993	1,347	-2,571
A58G	VUS		17,45	0,436	0,496	0,084	-0,097	0,453	0,249	0,628	0,290	1,470	-3,301	0,738	-2,752
A58P	VUS		19,40	0,346	0,535	0,081	0,025	0,384	-0,758	0,628	0,520	1,470	-3,080	1,096	-2,596
A58S	VUS		16,52	0,261	0,488	0,079	-0,138	0,270	0,295	0,628	0,270	1,470	-4,094	0,511	-3,378
A58T	VUS		18,31	0,310	0,503	0,068	-0,125	0,290	0,343	0,628	0,220	1,470	-3,794	0,540	-3,106
A58V	VUS		17,62	0,359	0,519	0,085	-0,130	0,354	0,362	0,628	0,440	1,470	-3,591	0,845	-2,985
N59D	VUS		16,03	0,369	0,554	0,075	-0,027	0,293	-0,054	0,713	0,140	1,720	-3,661	0,808	-3,047
N59H	VUS		21,80	0,483	0,535	0,084	-0,012	0,503	-0,393	0,713	0,440	1,720	-2,716	1,469	-2,358
N59I	VUS		21,20	0,395	0,547	0,147	0,046	0,632	-0,010	0,713	1,090	1,720	-2,436	2,299	-2,281
N59K	VUS		13,27	0,341	0,574	0,116	-0,029	0,248	-0,670	0,713	1,730	1,720	-3,794	2,670	-3,508
N59S	VUS		14,66	0,321	0,503	0,062	-0,065	0,216	-0,119	0,713	0,340	1,720	-4,179	0,919	-3,554
N59T	VUS		16,67	0,329	0,526	0,101	-0,008	0,234	-0,209	0,713	0,390	1,720	-3,759	1,099	-3,199
N59Y	VUS		22,20	0,492	0,558	0,126	0,031	0,636	-0,518	0,713	0,820	1,720	-2,247	2,041	-2,054
H60D	VUS		13,80	0,284	0,360	0,100	-0,100	0,248	2,681	0,196	0,580	2,110	-3,883	0,262	-3,197
H60L	VUS		0,36	0,328	0,352	0,072	-0,102	0,224	-1,292	0,196	0,720	2,110	-4,986	0,094	-4,216
H60N	VUS		12,78	0,267	0,359	0,096	-0,141	0,255	0,751	0,196	0,440	2,110	-4,169	0,049	-3,401
H60P	VUS		1,72	0,358	0,355	0,078	0,015	0,228	6,394	0,196	0,350	2,110	-4,243	-0,301	-3,521
H60Q	VUS		4,68	0,243	0,323	0,075	-0,175	0,204	0,111	0,196	0,470	2,110	-5,074	-0,203	-4,213
H60R	VUS		0,29	0,302	0,360	0,047	-0,116	0,192	-0,707	0,196	1,030	2,110	-5,084	0,430	-4,356
H60Y	VUS		9,11	0,282	0,363	0,075	-0,165	0,264	-1,002	0,196	0,380	2,110	-4,535	-0,120	-3,705
T61A	VUS		10,58	0,445	0,552	0,078	-0,085	0,233	0,684	0,814	0,220	1,620	-4,215	0,881	-3,586
T61K	VUS		13,68	0,555	0,610	0,107	0,001	0,323	-0,183	0,814	2,120	1,620	-3,170	3,462	-3,128
T61M	VUS		21,30	0,545	0,588	0,097	-0,027	0,428	0,581	0,814	0,770	1,620	-2,696	2,021	-2,415
T61P	VUS		12,80	0,447	0,553	0,106	0,078	0,322	1,993	0,814	0,300	1,620	-3,436	1,160	-2,987
T61R	VUS		14,00	0,614	0,600	0,078	0,004	0,352	-0,231	0,814	0,980	1,620	-3,128	2,106	-2,849
T61S	VUS		7,95	0,406	0,553	0,087	-0,130	0,218	0,150	0,814	0,050	1,620	-4,626	0,558	-3,884
G62A	VUS		18,25	0,418	0,508	0,093	-0,028	0,210	2,629	0,510	0,290	1,040	-3,091	0,204	-2,429
G62C	VUS		22,40	0,650	0,529	0,215	0,051	0,634	3,788	0,510	0,750	1,040	-1,271	1,219	-1,052
G62D	VUS		20,60	0,472	0,579	0,115	0,040	0,352	1,623	0,510	0,460	1,040	-2,286	0,633	-1,763
G62R	VUS		16,63	0,394	0,565	0,109	0,063	0,402	3,408	0,510	0,910	1,040	-2,517	1,042	-2,090
G62S	VUS		15,31	0,400	0,491	0,088	-0,018	0,349	3,916	0,510	0,020	1,040	-3,116	-0,183	-2,429
G62V	VUS		21,40	0,487	0,511	0,144	0,077	0,362	6,271	0,510	0,730	1,040	-2,016	0,959	-1,654
T63A	VUS		16,15	0,292	0,449	0,066	-0,089	0,165	0,086	0,808	0,220	0,400	-4,254	0,178	-3,531
T63I	VUS		18,41	0,425	0,531	0,111	-0,047	0,432	-0,041	0,808	0,700	0,400	-3,068	1,055	-2,622
T63K	VUS		16,73	0,403	0,499	0,069	0,005	0,220	-0,665	0,808	2,120	0,400	-3,462	2,670	-3,302
T63P	VUS		17,38	0,413	0,462	0,075	0,076	0,347	0,216	0,808	0,300	0,400	-3,217	0,530	-2,735
T63R	VUS		17,73	0,403	0,489	0,062	0,011	0,364	-0,878	0,808	0,980	0,400	-3,288	1,352	-2,917
T63S	VUS		15,20	0,345	0,457	0,077	-0,123	0,217	-0,012	0,808	0,050	0,400	-4,210	-0,024	-3,454
G64A	VUS		20,90	0,327	0,554	0,131	0,027	0,468	-0,388	0,327	0,290	0,170	-2,114	-0,471	-1,398
G64C	Likely pathogenic		23,10	0,647	0,584	0,294	0,104	0,902	-0,533	0,327	0,750	0,170	-0,238	0,562	0,021
G64D	VUS		21,00	0,427	0,630	0,146	0,080	0,560	0,278	0,327	0,460	0,170	-1,413	-0,120	-0,816
G64R	VUS		21,90	0,756	0,622	0,221	0,116	0,913	-0,706	0,327	0,910	0,170	-0,056	0,785	0,144
G64V	VUS		22,60	0,768	0,556	0,252	0,124	0,596	-0,488	0,327	0,730	0,170	-0,564	0,466	-0,253
L65M	VUS		17,61	0,494	0,577	0,127	-0,031	0,512	1,491	0,030	0,000	0,020	-1,557	-1,552	-0,666
L65P	VUS		25,80	0,753	0,671	0,277	0,163	0,871	2,123	0,030	1,070	0,020	1,009	0,418	1,251
L65Q	VUS		26,40	0,714	0,596	0,166	0,097	0,809	3,845	0,030	1,190	0,020	0,503	0,428	0,755
L65R	VUS		23,10	0,730	0,645	0,174	0,158	0,800	3,587	0,030	1,750	0,020	0,591	1,083	0,715
L65V	VUS		15,72	0,462	0,555	0,095	-0,033	0,412	1,563	0,030	0,110	0,020	-1,981	-1,540	-1,060
L66I	VUS		17,09	0,441	0,564	0,078	-0,102	0,273	0,558	0,169	0,070	-0,150	-2,496	-1,453	-1,504
L66P	Likely pathogenic	Mild	23,60	0,769	0,637	0,393	0,135	0,871	4,376	0,169	1,070	-0,150	0,800	0,537	0,995

L66Q	VUS	22,30	0,503	0,562	0,083	0,054	0,435	1,697	0,169	1,190	-0,150	-1,265	0,224	-0,772
L66R	VUS	22,10	0,384	0,608	0,089	0,100	0,380	1,161	0,169	1,750	-0,150	-1,341	0,876	-0,923
L66V	VUS	15,99	0,411	0,516	0,073	-0,071	0,284	0,332	0,169	0,110	-0,150	-2,666	-1,448	-1,705
L67M	VUS	22,00	0,590	0,658	0,214	0,076	0,685	-0,392	0,025	0,000	-0,390	-0,250	-1,438	0,512
L67P	VUS	28,10	0,878	0,755	0,951	0,330	0,917	5,475	0,025	1,070	-0,390	3,046	0,605	3,104
L67Q	VUS	27,70	0,854	0,686	0,787	0,268	0,891	3,152	0,025	1,190	-0,390	2,353	0,630	2,438
L67R	VUS	27,90	0,869	0,735	0,819	0,259	0,908	3,311	0,025	1,750	-0,390	2,602	1,357	2,560
L67V	VUS	15,54	0,352	0,635	0,164	0,064	0,307	2,362	0,025	0,110	-0,390	-1,740	-1,783	-0,739
T68A	VUS	21,70	0,508	0,585	0,091	-0,001	0,561	-0,214	0,203	0,220	-0,290	-1,350	-0,947	-0,614
T68I	VUS	23,90	0,657	0,683	0,103	0,014	0,665	-2,292	0,203	0,700	-0,290	-0,520	-0,120	0,030
T68N	VUS	22,70	0,560	0,644	0,103	0,044	0,593	0,030	0,203	0,390	-0,290	-0,847	-0,624	-0,197
T68P	VUS	24,10	0,818	0,587	0,256	0,170	0,763	0,795	0,203	0,300	-0,290	0,356	-0,425	0,738
T68S	VUS	20,60	0,437	0,594	0,100	-0,050	0,350	0,490	0,203	0,050	-0,290	-1,945	-1,319	-1,024
L69M	VUS	22,90	0,582	0,534	0,224	0,072	0,595	-0,303	0,005	0,000	-0,260	-0,626	-1,464	0,111
L69P	VUS	27,00	0,873	0,631	0,783	0,331	0,924	5,415	0,005	1,070	-0,260	2,490	0,511	2,512
L69Q	VUS	26,80	0,849	0,545	0,752	0,268	0,881	3,847	0,005	1,190	-0,260	1,923	0,561	1,955
L69R	VUS	27,00	0,837	0,601	0,766	0,327	0,907	3,722	0,005	1,750	-0,260	2,281	1,314	2,157
L69V	VUS	16,15	0,390	0,508	0,099	0,054	0,258	1,419	0,005	0,110	-0,260	-2,131	-1,797	-1,172
Q70E	VUS	19,07	0,316	0,377	0,082	-0,033	0,284	0,497	0,200	0,400	0,400	-2,982	-0,708	-2,217
Q70H	VUS	18,79	0,385	0,388	0,120	-0,025	0,336	-0,587	0,200	0,470	0,400	-2,744	-0,549	-2,036
Q70K	VUS	20,20	0,341	0,400	0,100	-0,002	0,265	-0,555	0,200	1,700	0,400	-2,680	0,960	-2,247
Q70L	VUS	21,60	0,405	0,368	0,079	-0,010	0,371	-0,785	0,200	1,190	0,400	-2,469	0,421	-1,992
Q70P	VUS	21,40	0,536	0,379	0,307	0,226	0,553	4,862	0,200	0,120	0,400	-0,966	-0,612	-0,534
Q70R	VUS	20,80	0,335	0,394	0,076	-0,001	0,302	-1,316	0,200	0,560	0,400	-2,750	-0,411	-2,053
P71A	VUS	17,68	0,447	0,490	0,086	-0,055	0,387	2,967	0,119	0,520	0,670	-2,289	-0,493	-1,571
P71L	VUS	22,50	0,454	0,550	0,115	0,000	0,455	3,663	0,119	1,070	0,670	-1,460	0,397	-0,955
P71Q	VUS	22,20	0,579	0,496	0,160	-0,014	0,396	4,108	0,119	0,120	0,670	-1,495	-0,751	-0,804
P71R	VUS	22,20	0,486	0,548	0,146	0,013	0,376	3,983	0,119	0,680	0,670	-1,493	-0,087	-0,887
P71S	VUS	18,72	0,446	0,490	0,125	-0,063	0,342	4,542	0,119	0,250	0,670	-2,217	-0,817	-1,431
P71T	VUS	16,94	0,436	0,512	0,095	-0,053	0,339	5,045	0,119	0,300	0,670	-2,297	-0,809	-1,501
E72A	VUS	21,60	0,419	0,459	0,095	-0,083	0,551	0,279	0,543	1,040	1,240	-2,716	1,485	-2,389
E72D	VUS	18,93	0,334	0,467	0,085	-0,119	0,376	0,245	0,543	0,290	1,240	-3,464	0,372	-2,810
E72G	VUS	22,40	0,464	0,431	0,070	-0,046	0,520	0,619	0,543	0,750	1,240	-2,635	1,163	-2,285
E72K	VUS	18,50	0,412	0,525	0,083	-0,056	0,633	0,012	0,543	1,300	1,240	-2,610	1,781	-2,334
E72Q	VUS	17,87	0,375	0,442	0,078	-0,105	0,474	0,092	0,543	0,400	1,240	-3,344	0,528	-2,778
E72V	VUS	22,40	0,500	0,447	0,167	-0,054	0,643	0,299	0,543	1,480	1,240	-2,217	2,148	-2,095
Q73E	VUS	15,90	0,418	0,466	0,066	-0,059	0,188	0,559	0,356	0,400	1,600	-3,471	0,250	-2,777
Q73H	VUS	21,70	0,408	0,496	0,224	-0,042	0,405	0,046	0,356	0,470	1,600	-2,488	0,638	-1,937
Q73K	VUS	16,91	0,431	0,507	0,078	-0,034	0,180	-0,569	0,356	1,700	1,600	-3,166	1,915	-2,790
Q73L	VUS	22,60	0,454	0,465	0,113	-0,028	0,361	-0,136	0,356	1,190	1,600	-2,520	1,515	-2,166
Q73P	VUS	22,60	0,616	0,477	0,332	0,206	0,624	-0,785	0,356	0,120	1,600	-1,093	0,580	-0,789
Q73R	VUS	22,20	0,420	0,501	0,084	-0,021	0,357	-0,388	0,356	0,560	1,600	-2,602	0,729	-2,063
K74E	VUS	23,20	0,541	0,534	0,147	0,021	0,522	0,181	0,403	1,300	0,980	-1,672	1,564	-1,419
K74M	VUS	22,00	0,636	0,490	0,129	0,015	0,617	-0,264	0,403	2,890	0,980	-1,474	3,532	-1,682
K74N	VUS	22,80	0,657	0,556	0,315	0,035	0,557	0,749	0,403	1,730	0,980	-1,101	2,209	-1,021
K74Q	VUS	23,10	0,430	0,504	0,094	-0,009	0,504	0,442	0,403	1,700	0,980	-2,078	1,944	-1,865
K74R	VUS	19,79	0,520	0,488	0,071	-0,083	0,287	-0,640	0,403	1,140	0,980	-2,793	1,091	-2,326
K74T	VUS	21,40	0,574	0,546	0,106	0,009	0,383	0,670	0,403	2,120	0,980	-1,910	2,467	-1,791
F75C	VUS	28,00	0,714	0,722	0,484	0,274	0,913	0,174	0,275	0,120	-0,020	1,289	-0,052	1,604
F75I	VUS	25,30	0,835	0,703	0,573	0,250	0,819	0,717	0,275	0,100	-0,020	1,204	-0,123	1,530
F75L	VUS	23,80	0,697	0,728	0,904	0,129	0,690	-0,564	0,275	0,030	-0,020	0,723	-0,313	1,248
F75S	VUS	24,50	0,638	0,703	0,486	0,217	0,896	0,118	0,275	0,850	-0,020	0,744	0,662	0,972
F75V	VUS	25,40	0,802	0,688	0,529	0,244	0,815	0,192	0,275	0,140	-0,020	1,027	-0,107	1,360
F75Y	VUS	21,70	0,354	0,716	0,087	0,088	0,472	0,411	0,275	0,370	-0,020	-1,368	-0,472	-0,625

Q76E	VUS		25,30	0,916	0,590	0,529	0,298	0,935	0,691	0,089	0,400	-0,380	1,680	-0,297	1,881	
Q76H	VUS		23,50	0,896	0,619	0,972	0,226	0,943	2,615	0,089	0,470	-0,380	1,995	-0,184	2,213	
Q76K	VUS		25,90	0,953	0,628	0,892	0,258	0,939	1,260	0,089	1,700	-0,380	2,314	1,423	2,191	
Q76L	VUS		26,30	0,937	0,584	0,840	0,312	0,935	0,564	0,089	1,190	-0,380	2,205	0,803	2,166	
Q76P	VUS		25,90	0,941	0,595	0,667	0,467	0,952	0,200	0,089	0,120	-0,380	2,322	-0,465	2,461	
Q76R	VUS		25,60	0,951	0,625	0,837	0,325	0,943	1,454	0,089	0,560	-0,380	2,309	0,039	2,426	
K77E	VUS		22,40	0,538	0,542	0,096	-0,019	0,269	0,257	0,538	1,300	0,110	-2,253	1,194	-1,857	
K77I	VUS		20,60	0,598	0,526	0,226	0,012	0,624	0,512	0,538	2,820	0,110	-1,435	3,199	-1,585	
K77N	VUS		22,40	0,466	0,572	0,259	-0,016	0,369	0,751	0,538	1,730	0,110	-1,934	1,774	-1,654	
K77Q	VUS		22,40	0,530	0,505	0,087	-0,040	0,330	0,497	0,538	1,700	0,110	-2,288	1,668	-2,012	
K77R	VUS		17,60	0,363	0,482	0,074	-0,114	0,312	0,066	0,538	1,140	0,110	-3,275	0,711	-2,715	
K77T	VUS		18,09	0,549	0,560	0,095	-0,028	0,340	1,121	0,538	2,120	0,110	-2,317	2,100	-2,108	
V78A	VUS		24,30	0,807	0,373	0,640	0,225	0,896	2,627	0,017	0,440	-0,740	1,028	-0,809	1,270	
V78E	VUS		25,50	0,823	0,368	0,892	0,349	0,930	3,451	0,017	1,480	-0,740	1,869	0,648	1,728	
V78G	VUS		25,40	0,796	0,338	0,668	0,351	0,854	3,732	0,017	0,730	-0,740	1,337	-0,390	1,418	
V78L	VUS		13,64	0,355	0,361	0,243	0,139	0,314	-0,810	0,017	0,110	-0,740	-2,291	-2,071	-1,403	
V78M	VUS		17,25	0,373	0,364	0,202	0,142	0,334	-0,859	0,017	0,110	-0,740	-2,005	-1,963	-1,157	
K79E	VUS		21,40	0,561	0,609	0,370	-0,013	0,641	2,059	0,106	1,300	-0,720	-0,420	0,036	0,061	
K79M	VUS		22,20	0,459	0,554	0,335	-0,022	0,526	-0,044	0,106	2,890	-0,720	-0,890	1,892	-0,725	
K79N	Likely pathogenic	Mild	22,70	0,538	0,632	0,810	0,004	0,812	2,152	0,106	1,730	-0,720	0,503	0,791	0,783	
K79Q	VUS		20,70	0,537	0,571	0,224	-0,034	0,421	1,159	0,106	1,700	-0,720	-1,153	0,356	-0,664	
K79R	VUS		21,80	0,451	0,545	0,097	-0,108	0,433	0,802	0,106	1,140	-0,720	-1,655	-0,420	-0,963	
K79T	VUS		23,30	0,560	0,614	0,377	-0,002	0,734	1,650	0,106	2,120	-0,720	-0,060	1,136	0,170	
G80A	Likely pathogenic		24,20	0,922	0,718	0,853	0,383	0,946	7,764	0,000	0,290	-1,100	3,042	-0,920	3,299	
G80E	Likely pathogenic		24,60	0,958	0,767	0,985	0,443	0,949	17,875	0,000	0,750	-1,100	3,998	-0,320	4,044	
G80R	Likely pathogenic		25,10	0,967	0,782	0,985	0,460	0,953	19,657	0,000	0,910	-1,100	4,218	-0,104	4,203	Yes
G80V	Likely pathogenic		24,50	0,962	0,723	0,949	0,470	0,954	15,994	0,000	0,730	-1,100	3,849	-0,341	3,874	
F81C	Likely pathogenic		27,10	0,949	0,730	0,971	0,390	0,944	3,438	0,004	0,120	-1,350	3,325	-1,091	3,625	
F81I	VUS		24,40	0,868	0,705	0,764	0,288	0,949	3,045	0,004	0,100	-1,350	2,434	-1,353	2,858	
F81L	Likely pathogenic		24,70	0,936	0,731	0,992	0,328	0,949	2,022	0,004	0,030	-1,350	2,951	-1,289	3,340	
F81S	Likely pathogenic		27,10	0,939	0,707	0,983	0,401	0,954	4,487	0,004	0,850	-1,350	3,401	-0,209	3,500	
F81V	VUS		24,50	0,925	0,689	0,841	0,360	0,949	3,716	0,004	0,140	-1,350	2,802	-1,223	3,133	
F81Y	VUS		25,60	0,897	0,718	0,735	0,238	0,895	0,937	0,004	0,370	-1,350	2,307	-1,006	2,715	
G82A	Likely pathogenic		24,20	0,951	0,663	0,901	0,381	0,944	7,012	0,000	0,290	-1,350	3,031	-1,054	3,275	
G82E	Likely pathogenic		24,60	0,965	0,719	0,992	0,441	0,949	23,076	0,000	0,750	-1,350	4,163	-0,523	4,176	
G82R	Likely pathogenic		25,20	0,968	0,733	0,976	0,390	0,953	27,115	0,000	0,910	-1,350	4,288	-0,368	4,272	
G82V	Likely pathogenic		24,50	0,975	0,669	0,987	0,468	0,950	19,841	0,000	0,730	-1,350	3,983	-0,525	3,977	
G83A	Likely pathogenic		24,10	0,867	0,640	0,360	0,269	0,791	1,688	0,000	0,290	-1,430	1,511	-1,380	1,977	
G83E	Likely pathogenic		24,60	0,922	0,706	0,978	0,417	0,956	6,503	0,000	0,750	-1,430	3,323	-0,473	3,458	
G83R	Pathogenic		23,90	0,954	0,717	0,975	0,361	0,947	19,273	0,000	0,910	-1,430	3,745	-0,418	3,807	
G83V	Likely pathogenic		24,50	0,942	0,652	0,910	0,444	0,953	8,267	0,000	0,730	-1,430	3,282	-0,533	3,371	
G83W	Likely pathogenic		24,60	0,937	0,701	0,984	0,455	0,936	38,915	0,000	0,760	-1,430	4,740	-0,708	4,667	
A84D	VUS		27,90	0,964	0,576	0,980	0,337	0,931	1,683	0,000	0,750	-1,430	2,904	-0,425	3,026	
A84G	VUS		27,50	0,873	0,460	0,335	0,173	0,737	1,698	0,000	0,290	-1,430	0,995	-1,434	1,434	
A84P	Likely pathogenic		23,20	0,948	0,539	0,987	0,376	0,942	2,480	0,000	0,520	-1,430	2,588	-0,839	2,759	
A84S	VUS		21,40	0,748	0,440	0,135	0,117	0,400	0,639	0,000	0,270	-1,430	-0,620	-1,893	0,089	
A84T	VUS		23,10	0,841	0,467	0,612	0,153	0,587	-0,848	0,000	0,220	-1,430	0,568	-1,616	1,136	
A84V	VUS		27,60	0,904	0,497	0,908	0,223	0,852	-0,488	0,000	0,440	-1,430	1,997	-0,967	2,302	
M85I	VUS		21,30	0,545	0,472	0,334	0,023	0,347	2,665	0,000	0,070	-0,870	-1,065	-1,933	-0,202	
M85K	VUS		23,00	0,849	0,546	0,953	0,260	0,835	3,989	0,000	2,890	-0,870	2,052	2,208	1,753	
M85L	VUS		13,74	0,427	0,467	0,155	-0,015	0,294	1,641	0,000	0,000	-0,870	-2,250	-2,372	-1,207	
M85R	VUS		24,30	0,832	0,524	0,945	0,287	0,888	4,775	0,000	1,750	-0,870	2,143	0,857	2,070	
M85T	VUS		22,60	0,747	0,580	0,607	0,093	0,806	5,684	0,000	0,770	-0,870	0,997	-0,648	1,401	
M85V	VUS		14,27	0,495	0,417	0,072	0,048	0,318	3,704	0,000	0,110	-0,870	-2,013	-2,207	-1,102	

T86A	VUS		24,80	0,890	0,622	0,851	0,286	0,939	2,687	0,000	0,220	-1,120	2,340	-1,069	2,673
T86I	VUS		25,10	0,916	0,718	0,992	0,312	0,957	5,350	0,000	0,700	-1,120	3,027	-0,383	3,225
T86K	VUS		25,50	0,933	0,700	0,976	0,369	0,953	6,404	0,000	2,120	-1,120	3,309	1,382	3,106
T86P	VUS		25,30	0,927	0,615	0,892	0,377	0,943	4,783	0,000	0,300	-1,120	2,791	-0,896	3,012
T86R	Likely pathogenic		25,30	0,944	0,689	0,969	0,372	0,955	7,879	0,000	0,980	-1,120	3,267	-0,028	3,323
T86S	VUS		23,10	0,815	0,628	0,618	0,157	0,886	0,936	0,000	0,050	-1,120	1,362	-1,501	1,906
D87A	VUS		25,30	0,840	0,633	0,970	0,344	0,892	0,134	0,000	0,750	-1,200	2,457	-0,402	2,675
D87E	VUS		17,48	0,607	0,661	0,934	0,289	0,903	-0,021	0,000	0,290	-1,200	1,336	-1,322	1,862
D87G	VUS		25,40	0,791	0,640	0,945	0,355	0,958	1,573	0,000	0,460	-1,200	2,523	-0,766	2,795
D87H	VUS		23,50	0,895	0,707	0,987	0,286	0,954	0,253	0,000	0,580	-1,200	2,574	-0,610	2,869
D87N	VUS		23,50	0,805	0,685	0,920	0,311	0,951	0,083	0,000	0,140	-1,200	2,294	-1,206	2,713
D87V	VUS		25,20	0,900	0,631	0,988	0,391	0,950	-1,495	0,000	1,190	-1,200	2,744	0,226	2,788
D87Y	VUS		23,50	0,911	0,690	0,982	0,389	0,950	1,553	0,000	0,960	-1,200	2,882	-0,101	3,002
A88D	VUS		25,60	0,895	0,667	0,971	0,338	0,922	10,507	0,000	0,750	-1,240	3,130	-0,455	3,277
A88G	VUS		25,40	0,808	0,556	0,483	0,250	0,724	1,547	0,000	0,290	-1,240	1,239	-1,293	1,695
A88P	VUS		24,50	0,898	0,623	0,975	0,378	0,933	8,857	0,000	0,520	-1,240	2,985	-0,742	3,156
A88S	VUS		22,70	0,700	0,533	0,112	0,118	0,394	1,200	0,000	0,270	-1,240	-0,448	-1,746	0,296
A88T	VUS		24,40	0,801	0,565	0,527	0,227	0,777	6,320	0,000	0,220	-1,240	1,442	-1,428	1,894
A88V	VUS		25,40	0,884	0,595	0,835	0,157	0,874	4,519	0,000	0,440	-1,240	1,999	-0,980	2,367
A89D	VUS		25,80	0,899	0,623	0,962	0,333	0,927	3,323	0,000	0,750	-1,220	2,733	-0,398	2,898
A89G	VUS		23,40	0,699	0,499	0,328	0,172	0,609	1,873	0,000	0,290	-1,220	0,233	-1,545	0,820
A89P	VUS		22,50	0,831	0,574	0,953	0,372	0,923	4,753	0,000	0,520	-1,220	2,374	-0,818	2,595
A89S	VUS		21,90	0,619	0,475	0,126	0,131	0,516	-0,285	0,000	0,270	-1,220	-0,631	-1,755	0,079
A89T	VUS		19,97	0,447	0,508	0,099	0,129	0,358	0,041	0,000	0,220	-1,220	-1,278	-2,007	-0,400
A89V	VUS		23,60	0,722	0,541	0,241	0,128	0,699	3,047	0,000	0,440	-1,220	0,386	-1,355	0,938
A90D	Likely pathogenic		24,70	0,830	0,591	0,964	0,287	0,817	4,911	0,000	0,750	-1,110	2,219	-0,487	2,457
A90G	VUS		21,80	0,438	0,479	0,136	0,093	0,322	1,802	0,000	0,290	-1,110	-1,276	-1,859	-0,424
A90P	Likely pathogenic		22,60	0,855	0,548	0,961	0,327	0,926	3,113	0,000	0,520	-1,110	2,181	-0,759	2,408
A90S	VUS		22,20	0,629	0,461	0,096	0,082	0,355	0,421	0,000	0,270	-1,110	-1,005	-1,780	-0,229
A90T	VUS	Mild	17,92	0,754	0,491	0,190	0,088	0,766	0,010	0,000	0,220	-1,110	-0,290	-1,712	0,339
A90V	VUS		24,90	0,813	0,519	0,458	0,173	0,823	1,628	0,000	0,440	-1,110	1,049	-1,078	1,457
L91F	VUS		23,40	0,637	0,580	0,140	0,123	0,424	-0,269	0,264	0,030	-0,560	-0,890	-1,066	-0,206
L91H	VUS		27,00	0,787	0,652	0,244	0,315	0,591	1,439	0,264	0,720	-0,560	0,760	0,159	1,025
L91I	VUS		16,59	0,319	0,592	0,093	0,036	0,233	0,177	0,264	0,070	-0,560	-2,471	-1,490	-1,491
L91P	VUS		27,50	0,826	0,670	0,887	0,288	0,809	5,561	0,264	1,070	-0,560	2,082	0,839	2,120
L91R	VUS		27,30	0,829	0,657	0,189	0,352	0,710	0,832	0,264	1,750	-0,560	1,133	1,500	1,072
L91V	VUS		20,10	0,427	0,540	0,099	0,096	0,284	1,244	0,264	0,110	-0,560	-1,870	-1,268	-1,053
N92D	VUS		24,30	0,818	0,721	0,838	0,227	0,922	3,940	0,021	0,140	-0,640	2,145	-0,909	2,566
N92H	VUS		24,00	0,850	0,694	0,516	0,217	0,932	27,046	0,021	0,440	-0,640	2,726	-0,832	2,949
N92I	VUS		26,00	0,813	0,685	0,723	0,342	0,942	1,252	0,021	1,090	-0,640	2,308	0,342	2,415
N92K	VUS		23,10	0,770	0,742	0,956	0,295	0,933	1,933	0,021	1,730	-0,640	2,362	1,078	2,390
N92S	VUS		24,70	0,751	0,641	0,194	0,184	0,912	1,233	0,021	0,340	-0,640	0,926	-0,910	1,377
N92T	VUS		25,40	0,820	0,678	0,423	0,244	0,911	0,988	0,021	0,390	-0,640	1,584	-0,680	1,955
N92Y	VUS		24,50	0,872	0,718	0,806	0,333	0,947	25,317	0,021	0,820	-0,640	3,438	-0,166	3,479
I93F	VUS		25,10	0,799	0,703	0,246	0,206	0,746	4,247	0,005	0,100	-0,660	1,189	-1,241	1,738
I93L	VUS		23,20	0,619	0,698	0,146	0,108	0,368	-0,106	0,005	0,070	-0,660	-0,390	-1,598	0,478
I93M	VUS		24,00	0,590	0,672	0,177	0,123	0,384	-0,376	0,005	0,070	-0,660	-0,367	-1,575	0,479
I93N	VUS		26,70	0,905	0,761	0,917	0,355	0,948	3,126	0,005	1,090	-0,660	3,068	0,444	3,141
I93S	VUS		26,80	0,907	0,707	0,835	0,358	0,925	4,241	0,005	0,750	-0,660	2,854	-0,029	2,990
I93T	VUS		25,70	0,872	0,772	0,809	0,275	0,860	2,556	0,005	0,700	-0,660	2,467	-0,173	2,753
I93V	VUS		22,90	0,668	0,629	0,130	0,077	0,299	1,324	0,005	0,040	-0,660	-0,619	-1,704	0,247
L94F	VUS		20,10	0,373	0,586	0,135	0,029	0,216	0,791	0,264	0,030	-0,800	-2,062	-1,558	-1,101
L94H	VUS		23,50	0,649	0,659	0,206	0,150	0,590	1,835	0,264	0,720	-0,800	-0,104	-0,248	0,365
L94I	VUS		20,30	0,477	0,597	0,154	0,049	0,329	0,822	0,264	0,070	-0,800	-1,581	-1,393	-0,722

L94P	VUS	28,70	0,787	0,690	0,798	0,259	0,804	4,304	0,264	1,070	-0,800	1,960	0,689	2,062
L94R	VUS	23,40	0,599	0,663	0,129	0,163	0,566	1,067	0,264	1,750	-0,800	-0,241	0,969	0,010
L94V	VUS	19,26	0,360	0,549	0,130	0,008	0,301	1,236	0,264	0,110	-0,800	-2,139	-1,493	-1,222
A95D	VUS	22,90	0,533	0,569	0,204	0,147	0,361	1,036	0,349	0,750	-0,740	-1,090	-0,187	-0,564
A95G	VUS	21,90	0,474	0,471	0,106	0,037	0,267	0,881	0,349	0,290	-0,740	-2,059	-0,966	-1,323
A95P	VUS	22,80	0,717	0,527	0,585	0,254	0,522	1,148	0,349	0,520	-0,740	0,061	-0,173	0,401
A95S	VUS	14,95	0,367	0,452	0,076	-0,011	0,156	0,034	0,349	0,270	-0,740	-3,145	-1,310	-2,240
A95T	VUS	21,50	0,406	0,477	0,096	0,014	0,242	1,286	0,349	0,220	-0,740	-2,294	-1,121	-1,488
A95V	VUS	22,90	0,525	0,503	0,168	0,101	0,337	2,488	0,349	0,440	-0,740	-1,412	-0,653	-0,805
L96M	VUS	22,80	0,642	0,522	0,289	0,279	0,714	0,414	0,030	0,000	-0,810	0,303	-1,541	0,862
L96P	VUS	26,80	0,909	0,625	0,965	0,465	0,955	2,040	0,030	1,070	-0,810	3,038	0,419	2,988
L96Q	VUS	26,50	0,865	0,539	0,873	0,334	0,942	2,074	0,030	1,190	-0,810	2,304	0,398	2,298
L96R	VUS	26,70	0,907	0,597	0,916	0,389	0,943	8,044	0,030	1,750	-0,810	3,002	1,125	2,797
L96V	VUS	22,60	0,696	0,489	0,192	0,207	0,752	2,872	0,030	0,110	-0,810	0,201	-1,473	0,724
S97A	VUS	23,90	0,680	0,420	0,176	0,083	0,438	0,353	0,213	0,270	-0,530	-1,083	-0,901	-0,517
S97L	VUS	27,90	0,807	0,443	0,243	0,160	0,656	-0,127	0,213	0,820	-0,530	0,101	0,095	0,334
S97P	VUS	22,00	0,498	0,436	0,239	0,204	0,252	-0,854	0,213	0,250	-0,530	-1,493	-1,030	-0,834
S97T	VUS	21,20	0,552	0,435	0,105	0,050	0,261	-0,166	0,213	0,050	-0,530	-1,940	-1,397	-1,150
P98A	VUS	19,71	0,395	0,322	0,060	-0,078	0,252	1,664	0,535	0,520	-0,420	-3,336	-0,369	-2,692
P98H	VUS	21,10	0,487	0,349	0,079	-0,009	0,371	2,318	0,535	0,350	-0,420	-2,619	-0,402	-2,059
P98L	VUS	18,68	0,463	0,361	0,065	-0,016	0,337	1,176	0,535	1,070	-0,420	-2,882	0,394	-2,440
P98R	VUS	17,71	0,521	0,362	0,073	0,003	0,348	1,545	0,535	0,680	-0,420	-2,780	-0,068	-2,276
P98S	VUS	19,56	0,394	0,315	0,066	-0,074	0,181	2,129	0,535	0,250	-0,420	-3,455	-0,730	-2,726
P98T	VUS	20,80	0,446	0,327	0,067	-0,056	0,321	1,817	0,535	0,300	-0,420	-2,995	-0,543	-2,364
P99A	VUS	6,87	0,228	0,273	0,044	-0,086	0,083	1,863	0,415	0,520	-0,490	-4,774	-1,181	-3,869
P99H	VUS	20,90	0,307	0,282	0,077	0,051	0,288	2,021	0,415	0,350	-0,490	-2,951	-0,802	-2,304
P99L	VUS	19,58	0,279	0,287	0,075	-0,023	0,278	1,683	0,415	1,070	-0,490	-3,242	-0,013	-2,695
P99R	VUS	18,77	0,297	0,288	0,065	-0,005	0,223	1,989	0,415	0,680	-0,490	-3,326	-0,516	-2,677
P99S	VUS	9,32	0,218	0,263	0,057	-0,082	0,078	2,571	0,415	0,250	-0,490	-4,618	-1,453	-3,672
P99T	VUS	9,10	0,215	0,268	0,060	-0,068	0,087	3,146	0,415	0,300	-0,490	-4,554	-1,392	-3,631
A100D	VUS	23,40	0,493	0,381	0,644	0,232	0,521	7,144	0,000	0,750	-0,640	0,061	-0,745	0,466
A100G	VUS	22,20	0,321	0,329	0,200	0,144	0,293	1,140	0,000	0,290	-0,640	-1,798	-1,652	-1,026
A100P	VUS	21,50	0,611	0,360	0,682	0,272	0,589	2,920	0,000	0,520	-0,640	0,149	-0,939	0,541
A100S	VUS	16,62	0,272	0,317	0,105	0,028	0,293	0,872	0,000	0,270	-0,640	-2,708	-1,950	-1,793
A100T	VUS	13,83	0,196	0,329	0,068	0,019	0,161	-0,107	0,000	0,220	-0,640	-3,323	-2,174	-2,278
A100V	VUS	19,35	0,309	0,348	0,091	0,026	0,236	3,319	0,000	0,440	-0,640	-2,362	-1,680	-1,502
Q101E	VUS	22,50	0,574	0,281	0,227	0,137	0,576	3,330	0,071	0,400	-0,670	-0,968	-1,193	-0,482
Q101H	VUS	21,30	0,624	0,286	0,764	0,206	0,701	5,721	0,071	0,470	-0,670	0,104	-0,898	0,427
Q101K	VUS	22,80	0,604	0,297	0,187	0,156	0,491	0,484	0,071	1,700	-0,670	-0,999	0,419	-0,798
Q101L	VUS	22,70	0,597	0,279	0,569	0,151	0,571	0,214	0,071	1,190	-0,670	-0,580	-0,081	-0,310
Q101P	VUS	23,90	0,654	0,290	0,792	0,379	0,790	6,660	0,071	0,120	-0,670	0,944	-1,111	1,175
Q101R	VUS	21,10	0,381	0,295	0,107	0,138	0,229	0,694	0,071	0,560	-0,670	-2,144	-1,257	-1,456
N102D	VUS	14,16	0,300	0,377	0,058	-0,030	0,111	0,048	0,354	0,140	-0,320	-3,738	-1,332	-2,805
N102H	VUS	16,59	0,313	0,372	0,075	-0,006	0,247	0,555	0,354	0,440	-0,320	-3,236	-0,834	-2,471
N102I	VUS	22,50	0,434	0,382	0,177	0,055	0,475	-0,135	0,354	1,090	-0,320	-1,945	0,336	-1,551
N102K	VUS	17,26	0,261	0,390	0,136	-0,017	0,167	0,307	0,354	1,730	-0,320	-3,235	0,730	-2,728
N102S	VUS	21,60	0,381	0,350	0,084	-0,038	0,218	0,564	0,354	0,340	-0,320	-2,921	-0,817	-2,177
N102T	VUS	20,40	0,382	0,372	0,105	0,002	0,220	0,800	0,354	0,390	-0,320	-2,815	-0,752	-2,093
N102Y	VUS	20,60	0,328	0,381	0,118	0,041	0,301	0,227	0,354	0,820	-0,320	-2,646	-0,187	-2,057
L103F	VUS	18,75	0,437	0,352	0,096	-0,017	0,316	0,600	0,149	0,030	-0,890	-2,427	-1,945	-1,536
L103M	VUS	14,63	0,356	0,348	0,103	-0,053	0,212	0,095	0,149	0,000	-0,890	-3,138	-2,188	-2,116
L103S	VUS	21,60	0,413	0,355	0,124	0,069	0,298	1,135	0,149	0,820	-0,890	-1,973	-0,866	-1,338
L103V	VUS	12,87	0,310	0,332	0,094	-0,037	0,214	1,471	0,149	0,110	-0,890	-3,291	-2,136	-2,288
L103W	VUS	22,60	0,474	0,387	0,129	0,162	0,603	1,393	0,149	0,080	-0,890	-1,074	-1,545	-0,446

L104I	VUS		25,30	0,698	0,628	0,262	0,130	0,607	5,786	0,000	0,070	-1,160	0,629	-1,749	1,316
L104P	VUS		26,90	0,908	0,714	0,985	0,433	0,956	11,969	0,000	1,070	-1,160	3,717	0,100	3,705
L104Q	VUS		26,60	0,892	0,639	0,962	0,370	0,952	3,750	0,000	1,190	-1,160	2,978	0,226	3,000
L104R	VUS		26,80	0,905	0,684	0,974	0,430	0,952	6,795	0,000	1,750	-1,160	3,447	0,952	3,292
L104V	VUS		25,20	0,734	0,587	0,221	0,173	0,692	4,733	0,000	0,110	-1,160	0,737	-1,652	1,334
L105F	VUS		24,90	0,861	0,636	0,301	0,253	0,896	1,245	0,005	0,030	-1,120	1,494	-1,477	1,970
L105H	VUS		26,60	0,887	0,700	0,877	0,362	0,957	3,104	0,005	0,720	-1,120	2,931	-0,315	3,104
L105I	VUS		23,00	0,590	0,643	0,187	0,095	0,659	1,488	0,005	0,070	-1,120	0,014	-1,816	0,795
L105P	VUS		27,00	0,895	0,725	0,956	0,403	0,959	5,418	0,005	1,070	-1,120	3,344	0,165	3,392
L105R	VUS		27,00	0,900	0,700	0,855	0,400	0,955	6,367	0,005	1,750	-1,120	3,260	0,949	3,135
L105V	VUS		24,20	0,734	0,600	0,233	0,152	0,812	2,607	0,005	0,110	-1,120	0,737	-1,591	1,323
K106E	VUS		19,44	0,419	0,520	0,092	0,013	0,230	1,282	0,394	1,300	-0,500	-2,366	0,391	-1,822
K106I	VUS		22,60	0,707	0,502	0,431	0,056	0,698	-0,200	0,394	2,820	-0,500	-0,412	2,766	-0,595
K106N	VUS		22,40	0,297	0,543	0,241	0,011	0,234	0,652	0,394	1,730	-0,500	-2,163	0,997	-1,697
K106Q	VUS		18,80	0,412	0,480	0,076	-0,011	0,249	0,600	0,394	1,700	-0,500	-2,565	0,837	-2,115
K106R	VUS		14,36	0,269	0,467	0,067	-0,089	0,170	0,183	0,394	1,140	-0,500	-3,554	-0,116	-2,803
K106T	VUS		21,10	0,565	0,536	0,131	0,031	0,442	0,659	0,394	2,120	-0,500	-1,497	1,617	-1,303
S107A	VUS		21,80	0,388	0,490	0,106	0,051	0,285	-0,384	0,039	0,270	-0,970	-1,707	-1,770	-0,797
S107L	Pathogenic	Severe	26,90	0,755	0,521	0,351	0,149	0,853	2,749	0,039	0,820	-0,970	0,945	-0,469	1,250
S107P	Likely pathogenic		24,90	0,773	0,512	0,961	0,298	0,830	3,245	0,039	0,250	-0,970	1,792	-0,970	2,111
S107T	VUS		22,60	0,638	0,504	0,181	0,051	0,481	4,582	0,039	0,050	-0,970	-0,579	-1,840	0,183
S107W	Likely pathogenic		27,70	0,777	0,541	0,812	0,232	0,695	20,661	0,039	0,740	-0,970	2,295	-0,549	2,487
Y108C	VUS		28,80	0,874	0,665	0,479	0,343	0,956	4,555	0,004	0,250	-1,140	2,523	-1,001	2,817
Y108D	VUS		26,00	0,898	0,656	0,986	0,455	0,961	5,916	0,004	0,960	-1,140	3,294	-0,004	3,310
Y108F	VUS		26,50	0,801	0,594	0,238	0,205	0,731	0,743	0,004	0,370	-1,140	0,972	-1,174	1,457
Y108H	VUS		25,80	0,898	0,670	0,929	0,348	0,956	3,163	0,004	0,380	-1,140	2,830	-0,767	3,082
Y108N	VUS		25,90	0,856	0,664	0,915	0,361	0,958	3,960	0,004	0,820	-1,140	2,826	-0,253	2,972
Y108S	VUS		28,00	0,892	0,612	0,823	0,445	0,954	5,575	0,004	0,480	-1,140	3,054	-0,608	3,176
F109C	VUS		28,90	0,890	0,732	0,941	0,277	0,955	4,760	0,008	0,120	-0,970	3,028	-0,920	3,369
F109I	VUS		27,40	0,831	0,718	0,907	0,329	0,951	5,290	0,008	0,100	-0,970	2,877	-1,008	3,220
F109L	VUS		25,40	0,838	0,737	0,982	0,291	0,955	1,783	0,008	0,030	-0,970	2,643	-1,107	3,060
F109S	VUS		29,00	0,880	0,714	0,968	0,364	0,957	4,976	0,008	0,850	-0,970	3,278	0,018	3,380
F109V	VUS		27,50	0,868	0,700	0,868	0,255	0,958	4,368	0,008	0,140	-0,970	2,670	-0,987	3,031
F109Y	VUS		24,20	0,692	0,728	0,474	0,177	0,886	0,968	0,008	0,370	-0,970	1,315	-1,027	1,847
S110A	VUS		24,70	0,689	0,513	0,123	0,074	0,470	-0,287	0,019	0,270	-0,260	-0,637	-1,080	0,015
S110C	VUS		29,00	0,896	0,530	0,230	0,213	0,754	-0,126	0,019	0,730	-0,260	1,017	-0,085	1,252
S110F	VUS		29,40	0,888	0,558	0,493	0,241	0,866	2,387	0,019	0,850	-0,260	1,731	0,188	1,859
S110P	VUS		29,00	0,849	0,538	0,682	0,248	0,859	1,766	0,019	0,250	-0,260	1,730	-0,522	2,002
S110T	VUS		25,80	0,686	0,525	0,206	0,072	0,414	0,785	0,019	0,050	-0,260	-0,505	-1,320	0,208
S110Y	VUS		28,20	0,870	0,575	0,454	0,246	0,821	2,451	0,019	0,480	-0,260	1,530	-0,324	1,788
E111A	VUS		16,18	0,395	0,271	0,090	-0,044	0,250	0,807	0,094	1,040	0,580	-3,183	-0,139	-2,583
E111D	VUS		17,44	0,354	0,271	0,104	-0,086	0,186	1,575	0,094	0,290	0,580	-3,377	-1,088	-2,545
E111G	VUS		17,73	0,376	0,257	0,086	-0,014	0,250	1,803	0,094	0,750	0,580	-3,057	-0,458	-2,422
E111K	VUS		16,99	0,339	0,294	0,065	-0,032	0,304	0,428	0,094	1,300	0,580	-3,092	0,204	-2,553
E111Q	VUS		16,53	0,398	0,263	0,080	-0,065	0,288	-0,214	0,094	0,400	0,580	-3,266	-0,906	-2,513
E111V	VUS		17,40	0,386	0,265	0,178	-0,027	0,392	0,540	0,094	1,480	0,580	-2,759	0,511	-2,342
E112A	VUS		23,50	0,487	0,430	0,114	0,003	0,414	0,426	0,345	1,040	0,370	-2,051	0,658	-1,643
E112D	VUS		22,00	0,342	0,433	0,091	-0,055	0,292	0,834	0,345	0,290	0,370	-2,812	-0,455	-2,073
E112G	VUS		25,30	0,496	0,412	0,114	0,028	0,425	0,777	0,345	0,750	0,370	-1,881	0,365	-1,450
E112K	VUS		20,70	0,446	0,502	0,109	0,010	0,439	-0,479	0,345	1,300	0,370	-2,125	0,925	-1,720
E112Q	VUS		18,92	0,434	0,423	0,116	-0,039	0,303	0,039	0,345	0,400	0,370	-2,818	-0,338	-2,134
E112V	VUS		23,70	0,484	0,419	0,231	0,019	0,539	1,254	0,345	1,480	0,370	-1,649	1,274	-1,423
G113A	Likely pathogenic		23,10	0,884	0,607	0,715	0,312	0,873	7,537	0,029	0,290	-0,830	2,094	-0,901	2,378
G113E	Likely pathogenic		23,40	0,890	0,669	0,899	0,439	0,893	23,226	0,029	0,750	-0,830	3,491	-0,302	3,499

G113R	Likely pathogenic		24,30	0,873	0,680	0,959	0,456	0,951	27,569	0,029	0,910	-0,830	3,930	-0,075	3,843	
G113V	Likely pathogenic		23,30	0,865	0,619	0,922	0,466	0,928	22,395	0,029	0,730	-0,830	3,423	-0,321	3,399	
I114F	Likely pathogenic		22,60	0,814	0,561	0,501	0,263	0,877	11,064	0,000	0,100	-1,230	1,719	-1,607	2,124	
I114L	VUS		17,17	0,575	0,553	0,107	0,084	0,373	2,672	0,000	0,070	-1,230	-1,100	-2,232	-0,187	
I114M	VUS		18,36	0,616	0,521	0,147	0,192	0,735	2,005	0,000	0,070	-1,230	-0,198	-1,990	0,478	
I114N	Likely pathogenic		23,90	0,868	0,635	0,523	0,261	0,948	2,839	0,000	1,090	-1,230	1,949	-0,206	2,134	
I114S	Likely pathogenic		23,70	0,842	0,574	0,360	0,248	0,901	4,217	0,000	0,750	-1,230	1,492	-0,747	1,774	
I114T	Likely pathogenic		23,40	0,866	0,653	0,256	0,259	0,897	3,091	0,000	0,700	-1,230	1,558	-0,789	1,888	
I114V	Likely pathogenic		22,00	0,672	0,485	0,145	0,074	0,692	0,962	0,000	0,040	-1,230	-0,316	-1,985	0,396	
G115A	VUS		18,53	0,410	0,394	0,105	0,137	0,564	2,605	0,010	0,290	-1,150	-1,303	-1,910	-0,570	
G115E	VUS		13,50	0,344	0,392	0,086	0,160	0,323	3,298	0,010	0,750	-1,150	-2,048	-1,602	-1,292	
G115R	VUS		21,70	0,615	0,446	0,148	0,196	0,711	2,355	0,010	0,910	-1,150	-0,121	-0,844	0,287	
G115V	Likely pathogenic		22,20	0,660	0,381	0,420	0,229	0,776	3,877	0,010	0,730	-1,150	0,370	-0,948	0,701	
Y116C	Likely pathogenic		25,40	0,887	0,694	0,705	0,340	0,953	3,218	0,000	0,250	-0,940	2,525	-0,896	2,834	
Y116D	Likely pathogenic		25,80	0,934	0,690	0,971	0,452	0,952	5,500	0,000	0,960	-0,940	3,341	0,124	3,355	
Y116F	Likely pathogenic		24,10	0,808	0,625	0,246	0,197	0,735	-0,792	0,000	0,370	-0,940	0,788	-1,105	1,302	
Y116H	Likely pathogenic		25,60	0,926	0,702	0,947	0,345	0,950	2,931	0,000	0,380	-0,940	2,905	-0,635	3,152	Yes
Y116N	Likely pathogenic		25,80	0,928	0,694	0,897	0,431	0,954	3,185	0,000	0,820	-0,940	3,104	-0,062	3,186	
Y116S	Likely pathogenic		25,00	0,939	0,644	0,927	0,374	0,951	3,955	0,000	0,480	-0,940	2,851	-0,544	3,025	
N117D	Likely pathogenic	Mild	25,50	0,668	0,575	0,205	0,107	0,906	-0,174	0,036	0,140	-0,790	0,424	-1,285	0,983	
N117H	Likely pathogenic		25,20	0,745	0,541	0,202	0,176	0,846	14,903	0,036	0,440	-0,790	1,183	-1,010	1,522	
N117I	Likely pathogenic		24,80	0,839	0,542	0,855	0,221	0,935	0,375	0,036	1,090	-0,790	1,751	0,184	1,873	
N117K	Likely pathogenic		23,00	0,698	0,599	0,691	0,174	0,901	1,606	0,036	1,730	-0,790	1,245	0,775	1,352	
N117S	VUS		21,00	0,523	0,478	0,074	0,040	0,433	0,922	0,036	0,340	-0,790	-1,351	-1,522	-0,575	
N117T	VUS		21,90	0,515	0,522	0,213	0,089	0,496	2,071	0,036	0,390	-0,790	-0,780	-1,343	-0,070	
N117Y	Likely pathogenic		25,90	0,830	0,567	0,640	0,212	0,938	8,843	0,036	0,820	-0,790	1,943	-0,246	2,109	
I118F	VUS		22,10	0,525	0,543	0,231	0,076	0,501	2,241	0,000	0,100	-0,900	-0,645	-1,817	0,165	
I118L	VUS		21,20	0,485	0,534	0,107	-0,010	0,288	0,071	0,000	0,070	-0,900	-1,557	-2,040	-0,570	
I118M	VUS		22,10	0,463	0,494	0,115	0,005	0,440	0,135	0,000	0,070	-0,900	-1,357	-1,980	-0,451	
I118N	Likely pathogenic		24,90	0,883	0,611	0,680	0,248	0,895	3,327	0,000	1,090	-0,900	2,007	0,026	2,158	
I118S	Likely pathogenic		25,00	0,871	0,558	0,640	0,251	0,860	4,298	0,000	0,750	-0,900	1,793	-0,444	2,015	
I118T	Likely pathogenic		24,20	0,821	0,641	0,495	0,084	0,723	2,956	0,000	0,700	-0,900	1,015	-0,688	1,482	
I118V	VUS		21,00	0,458	0,447	0,088	-0,045	0,357	0,797	0,000	0,040	-0,900	-1,804	-2,138	-0,841	
I119F	Likely pathogenic		22,10	0,713	0,567	0,352	0,173	0,839	6,649	0,000	0,100	-1,200	0,878	-1,721	1,431	
I119L	Likely pathogenic		17,47	0,512	0,543	0,106	0,003	0,379	-0,141	0,000	0,070	-1,200	-1,530	-2,261	-0,537	Yes
I119M	Likely pathogenic		22,10	0,489	0,515	0,163	0,030	0,477	0,205	0,000	0,070	-1,200	-1,029	-2,093	-0,134	
I119N	Likely pathogenic		26,80	0,640	0,632	0,902	0,181	0,920	3,850	0,000	1,090	-1,200	1,933	-0,175	2,208	
I119S	Likely pathogenic		24,30	0,679	0,570	0,760	0,176	0,892	5,366	0,000	0,750	-1,200	1,511	-0,732	1,861	
I119T	Likely pathogenic	Severe	23,80	0,665	0,653	0,715	0,086	0,881	2,636	0,000	0,700	-1,200	1,248	-0,824	1,733	
I119V	Likely pathogenic		14,48	0,393	0,464	0,109	-0,046	0,267	1,361	0,000	0,040	-1,200	-2,377	-2,548	-1,280	
R120G	Likely pathogenic		22,60	0,947	0,536	0,972	0,434	0,927	6,153	0,000	0,910	-1,020	2,733	-0,152	2,734	
R120L	Likely pathogenic		24,20	0,963	0,567	0,962	0,401	0,944	1,700	0,000	1,750	-1,020	2,767	0,962	2,598	
R120P	Likely pathogenic		24,40	0,971	0,583	0,992	0,473	0,949	6,220	0,000	0,680	-1,020	3,151	-0,318	3,170	
R120Q	Pathogenic	Severe	24,30	0,964	0,478	0,873	0,306	0,956	4,431	0,000	0,560	-1,020	2,279	-0,622	2,405	Yes
R120W	Pathogenic	Severe	23,10	0,922	0,627	0,871	0,340	0,942	6,735	0,000	1,670	-1,020	2,705	0,735	2,622	Yes
V121A	Likely pathogenic		23,60	0,721	0,574	0,745	0,149	0,854	3,224	0,000	0,440	-1,360	1,334	-1,215	1,805	
V121E	Likely pathogenic		22,70	0,796	0,556	0,937	0,340	0,774	4,718	0,000	1,480	-1,360	2,061	0,183	2,139	
V121G	Likely pathogenic		24,00	0,769	0,476	0,837	0,342	0,757	5,215	0,000	0,730	-1,360	1,745	-0,781	1,979	
V121I	VUS		14,77	0,564	0,421	0,087	0,051	0,369	-0,656	0,000	0,040	-1,360	-1,825	-2,467	-0,888	
V121L	VUS		15,73	0,641	0,545	0,347	0,122	0,429	-0,397	0,000	0,110	-1,360	-0,753	-2,134	0,098	
P122A	Likely pathogenic		23,60	0,907	0,659	0,639	0,307	0,933	3,082	0,000	0,520	-1,370	2,270	-0,912	2,569	
P122H	Likely pathogenic		26,10	0,816	0,707	0,935	0,345	0,932	9,777	0,000	0,350	-1,370	3,068	-1,025	3,364	
P122L	Likely pathogenic	Severe	26,40	0,962	0,719	0,901	0,355	0,942	4,142	0,000	1,070	-1,370	3,219	-0,013	3,310	
P122R	Likely pathogenic		26,10	0,934	0,714	0,979	0,377	0,944	11,488	0,000	0,680	-1,370	3,547	-0,540	3,683	

P122S	Likely pathogenic	Mild	24,00	0,836	0,670	0,698	0,239	0,912	3,825	0,000	0,250	-1,370	2,071	-1,295	2,507	
P122T	Likely pathogenic		23,80	0,879	0,696	0,670	0,246	0,942	4,002	0,000	0,300	-1,370	2,241	-1,204	2,646	
M123I	Likely pathogenic		21,70	0,658	0,553	0,468	0,106	0,817	1,355	0,004	0,070	-0,950	0,374	-1,615	1,004	
M123K	Pathogenic		26,00	0,944	0,619	0,972	0,357	0,958	3,229	0,004	2,890	-0,950	3,034	2,438	2,610	
M123L	Likely pathogenic		23,80	0,701	0,554	0,320	0,099	0,796	1,464	0,004	0,000	-0,950	0,394	-1,675	1,029	
M123R	Likely pathogenic		26,20	0,935	0,600	0,971	0,384	0,958	6,058	0,004	1,750	-0,950	3,081	1,029	2,895	
M123T	Likely pathogenic	Mild	25,20	0,922	0,649	0,835	0,266	0,943	4,153	0,004	0,770	-0,950	2,493	-0,278	2,678	
M123V	Likely pathogenic	Mild	22,50	0,754	0,504	0,121	0,161	0,850	2,560	0,004	0,110	-0,950	0,346	-1,584	0,873	
A124D	Likely pathogenic		26,60	0,833	0,679	0,953	0,339	0,914	6,216	0,016	0,750	-0,940	2,824	-0,219	2,992	
A124G	VUS		23,60	0,514	0,576	0,085	0,148	0,439	-0,431	0,016	0,290	-0,940	-0,673	-1,528	0,098	
A124P	Likely pathogenic		25,20	0,898	0,641	0,983	0,378	0,937	7,204	0,016	0,520	-0,940	2,944	-0,493	3,096	
A124S	Likely pathogenic		24,50	0,811	0,557	0,248	0,142	0,727	0,172	0,016	0,270	-0,940	0,530	-1,251	1,060	
A124T	Likely pathogenic		25,00	0,880	0,593	0,876	0,160	0,886	8,884	0,016	0,220	-0,940	2,111	-1,076	2,475	
A124V	Likely pathogenic		26,40	0,847	0,614	0,960	0,157	0,934	9,174	0,016	0,440	-0,940	2,382	-0,739	2,681	
S125C	Likely pathogenic		24,10	0,881	0,551	0,606	0,118	0,749	2,156	0,013	0,730	-0,840	1,091	-0,534	1,446	Yes
S125G	Likely pathogenic		22,50	0,661	0,501	0,275	0,066	0,530	1,894	0,013	0,020	-0,840	-0,439	-1,772	0,301	
S125I	Likely pathogenic		26,60	0,937	0,568	0,991	0,242	0,948	13,556	0,013	0,750	-0,840	2,904	-0,252	2,980	
S125N	Likely pathogenic	Severe	25,50	0,869	0,590	0,954	0,183	0,909	15,764	0,013	0,340	-0,840	2,570	-0,883	2,832	
S125R	Pathogenic		25,70	0,938	0,602	0,996	0,255	0,948	21,974	0,013	0,930	-0,840	3,319	-0,104	3,319	
S125T	Likely pathogenic		25,10	0,825	0,547	0,804	0,084	0,795	5,972	0,013	0,050	-0,840	1,356	-1,344	1,858	
C126F	Likely pathogenic		26,20	0,934	0,689	0,979	0,345	0,924	11,789	0,000	0,120	-0,790	3,242	-0,924	3,487	
C126G	Likely pathogenic		28,30	0,905	0,647	0,460	0,344	0,912	-0,212	0,000	0,750	-0,790	2,193	-0,182	2,369	
C126R	Likely pathogenic		28,80	0,942	0,756	0,983	0,403	0,934	13,920	0,000	1,660	-0,790	3,961	1,082	3,784	
C126S	Likely pathogenic		22,50	0,815	0,677	0,349	0,243	0,642	1,175	0,000	0,730	-0,790	0,975	-0,591	1,409	
C126W	Likely pathogenic		25,50	0,822	0,727	0,993	0,348	0,914	27,534	0,000	0,010	-0,790	3,716	-1,246	3,965	
C126Y	Likely pathogenic		25,90	0,926	0,768	0,988	0,354	0,934	17,647	0,000	0,250	-0,790	3,691	-0,782	3,900	
D127A	Likely pathogenic		27,30	0,910	0,631	0,995	0,277	0,960	0,254	0,021	0,750	-0,760	2,585	-0,023	2,733	
D127E	Likely pathogenic		24,50	0,911	0,658	0,995	0,290	0,939	0,529	0,021	0,290	-0,760	2,430	-0,655	2,721	
D127G	Likely pathogenic		27,40	0,917	0,644	0,994	0,288	0,961	1,827	0,021	0,460	-0,760	2,706	-0,373	2,911	
D127H	Likely pathogenic		25,50	0,933	0,698	0,995	0,354	0,952	3,046	0,021	0,580	-0,760	2,935	-0,226	3,098	
D127N	Likely pathogenic		25,70	0,849	0,680	0,974	0,311	0,953	0,214	0,021	0,140	-0,760	2,472	-0,813	2,809	
D127V	Likely pathogenic	Mild	27,10	0,948	0,626	0,997	0,392	0,949	4,796	0,021	1,190	-0,760	3,112	0,548	3,045	
D127Y	Likely pathogenic		25,60	0,931	0,683	0,989	0,390	0,948	10,169	0,021	0,960	-0,760	3,295	0,191	3,300	
F128C	Likely pathogenic		28,20	0,940	0,709	0,962	0,406	0,951	4,783	0,004	0,120	-0,890	3,336	-0,810	3,570	
F128I	Likely pathogenic		26,70	0,948	0,686	0,979	0,381	0,937	7,324	0,004	0,100	-0,890	3,232	-0,917	3,474	
F128L	Likely pathogenic		26,90	0,860	0,713	0,997	0,276	0,941	5,100	0,004	0,030	-0,890	2,819	-1,063	3,196	
F128S	Likely pathogenic		28,20	0,962	0,688	0,968	0,416	0,952	5,489	0,004	0,850	-0,890	3,445	0,086	3,471	
F128V	Likely pathogenic		26,80	0,938	0,675	0,969	0,376	0,940	6,858	0,004	0,140	-0,890	3,158	-0,875	3,394	
F128Y	Likely pathogenic		26,20	0,862	0,698	0,787	0,251	0,906	7,355	0,004	0,370	-0,890	2,514	-0,776	2,837	
S129A	Likely pathogenic		25,90	0,824	0,566	0,489	0,278	0,838	1,394	0,000	0,270	-0,640	1,445	-0,888	1,798	
S129C	Likely pathogenic		27,50	0,925	0,586	0,720	0,329	0,927	1,808	0,000	0,730	-0,640	2,357	-0,101	2,473	
S129F	Likely pathogenic		27,80	0,954	0,607	0,992	0,356	0,943	4,480	0,000	0,850	-0,640	2,994	0,154	3,019	
S129P	Likely pathogenic		26,80	0,916	0,591	0,984	0,432	0,953	4,499	0,000	0,250	-0,640	2,956	-0,590	3,093	
S129T	Likely pathogenic		25,30	0,866	0,577	0,842	0,269	0,927	0,973	0,000	0,050	-0,640	1,977	-1,017	2,327	
S129Y	Likely pathogenic		26,80	0,958	0,627	0,988	0,361	0,948	4,989	0,000	0,480	-0,640	2,984	-0,317	3,107	
I130F	VUS		22,60	0,555	0,420	0,145	0,199	0,553	-0,268	0,492	0,100	-0,550	-1,415	-0,548	-0,962	
I130L	VUS		20,80	0,378	0,406	0,085	0,030	0,271	-0,230	0,492	0,070	-0,550	-2,806	-0,936	-2,057	
I130M	VUS		21,70	0,319	0,383	0,099	0,045	0,464	-0,325	0,492	0,070	-0,550	-2,571	-0,870	-1,897	
I130N	VUS		23,00	0,489	0,475	0,263	0,199	0,571	0,836	0,492	1,090	-0,550	-1,105	0,694	-0,867	
I130S	VUS		22,30	0,541	0,419	0,204	0,195	0,485	0,978	0,492	0,750	-0,550	-1,412	0,212	-1,093	
I130T	VUS		19,84	0,342	0,492	0,146	0,086	0,290	1,006	0,492	0,700	-0,550	-2,409	-0,130	-1,807	
I130V	VUS		19,06	0,301	0,347	0,062	-0,016	0,244	0,608	0,492	0,040	-0,550	-3,353	-1,130	-2,545	
R131C	Pathogenic	Severe	26,50	0,831	0,420	0,205	0,236	0,861	1,779	0,365	1,660	-0,230	0,311	1,655	0,121	Yes
R131G	Likely pathogenic		25,90	0,795	0,361	0,414	0,267	0,767	2,056	0,365	0,910	-0,230	0,183	0,723	0,168	

R131H	Likely pathogenic		22,10	0,852	0,368	0,083	0,167	0,770	2,322	0,365	1,030	-0,230	-0,544	0,643	-0,495
R131L	Likely pathogenic	Severe	25,10	0,878	0,382	0,499	0,234	0,927	1,454	0,365	1,750	-0,230	0,623	1,843	0,346
R131P	Likely pathogenic		27,20	0,879	0,387	0,937	0,442	0,929	4,233	0,365	0,680	-0,230	1,805	0,815	1,598
R131S	Likely pathogenic		25,40	0,814	0,365	0,545	0,219	0,567	1,640	0,365	0,930	-0,230	-0,092	0,691	-0,018
T132A	VUS		11,61	0,337	0,264	0,075	-0,054	0,173	0,828	0,360	0,220	-0,470	-4,005	-1,400	-3,140
T132I	VUS		5,39	0,283	0,278	0,065	-0,023	0,169	-0,958	0,360	0,700	-0,470	-4,492	-0,980	-3,676
T132N	VUS		9,26	0,327	0,275	0,100	0,031	0,253	0,724	0,360	0,390	-0,470	-3,802	-1,175	-3,034
T132P	VUS		15,03	0,330	0,272	0,059	0,104	0,267	-0,253	0,360	0,300	-0,470	-3,295	-1,084	-2,595
T132S	VUS		10,98	0,391	0,264	0,109	-0,069	0,226	1,308	0,360	0,050	-0,470	-3,858	-1,583	-2,983
Y133C	VUS		24,10	0,607	0,612	0,720	0,417	0,900	4,169	0,015	0,250	-0,540	1,765	-0,821	2,088
Y133D	Likely pathogenic		22,90	0,671	0,599	0,916	0,460	0,904	5,335	0,015	0,960	-0,540	2,200	0,113	2,281
Y133F	VUS		23,30	0,541	0,544	0,321	0,216	0,663	1,171	0,015	0,370	-0,540	0,032	-1,039	0,583
Y133H	VUS		22,80	0,640	0,616	0,743	0,286	0,745	4,689	0,015	0,380	-0,540	1,258	-0,803	1,681
Y133N	VUS		22,80	0,592	0,608	0,811	0,439	0,930	4,742	0,015	0,820	-0,540	1,902	-0,125	2,066
Y133S	VUS		23,80	0,579	0,562	0,780	0,383	0,743	3,970	0,015	0,480	-0,540	1,333	-0,635	1,666
T134A	Likely pathogenic		27,90	0,793	0,423	0,373	0,129	0,823	1,206	0,047	0,220	-0,490	0,575	-0,904	0,952
T134I	Likely pathogenic	Mild	25,80	0,948	0,516	0,932	0,243	0,944	3,545	0,047	0,700	-0,490	2,144	-0,015	2,234
T134N	Likely pathogenic		25,20	0,825	0,480	0,561	0,273	0,822	0,618	0,047	0,390	-0,490	1,120	-0,594	1,391
T134P	Likely pathogenic		28,00	0,911	0,423	0,752	0,382	0,886	1,405	0,047	0,300	-0,490	1,938	-0,476	2,042
T134S	Likely pathogenic		21,90	0,630	0,427	0,173	0,076	0,366	1,293	0,047	0,050	-0,490	-1,191	-1,602	-0,447
Y135C	Likely pathogenic		29,30	0,930	0,641	0,330	0,405	0,954	3,246	0,015	0,250	-0,490	2,388	-0,569	2,574
Y135D	Likely pathogenic		24,80	0,934	0,631	0,935	0,449	0,953	5,133	0,015	0,960	-0,490	2,959	0,355	2,922
Y135F	Likely pathogenic		25,10	0,865	0,582	0,342	0,272	0,870	-0,573	0,015	0,370	-0,490	1,250	-0,662	1,572
Y135H	Likely pathogenic		24,60	0,931	0,644	0,862	0,342	0,944	2,698	0,015	0,380	-0,490	2,477	-0,419	2,677
Y135N	Likely pathogenic		26,10	0,919	0,637	0,755	0,428	0,956	2,229	0,015	0,820	-0,490	2,660	0,176	2,696
Y135S	Likely pathogenic		26,20	0,929	0,595	0,733	0,439	0,959	3,769	0,015	0,480	-0,490	2,633	-0,260	2,714
A136E	Likely pathogenic		22,20	0,715	0,375	0,667	0,250	0,634	9,165	0,008	1,040	-0,140	0,617	0,027	0,767
A136G	Likely pathogenic		18,89	0,576	0,347	0,229	0,101	0,444	1,800	0,008	0,290	-0,140	-1,433	-1,275	-0,799
A136P	Likely pathogenic		23,10	0,773	0,375	0,920	0,304	0,791	5,780	0,008	0,520	-0,140	1,257	-0,370	1,414
A136S	Likely pathogenic		22,20	0,621	0,334	0,109	0,070	0,525	1,422	0,008	0,270	-0,140	-1,250	-1,212	-0,660
A136T	Likely pathogenic		22,70	0,675	0,351	0,174	0,086	0,588	0,811	0,008	0,220	-0,140	-0,895	-1,171	-0,344
A136V	Likely pathogenic		18,82	0,469	0,353	0,282	0,073	0,520	0,229	0,008	0,440	-0,140	-1,576	-1,106	-0,939
D137A	Likely pathogenic		25,50	0,878	0,490	0,442	0,231	0,938	0,417	0,378	0,750	0,360	0,603	1,038	0,572
D137E	Likely pathogenic		16,17	0,676	0,517	0,360	0,150	0,862	0,472	0,378	0,290	0,360	-0,806	0,032	-0,478
D137G	Likely pathogenic		25,60	0,952	0,502	0,394	0,310	0,949	0,269	0,378	0,460	0,360	0,903	0,762	0,869
D137H	Likely pathogenic		24,20	0,950	0,560	0,678	0,308	0,950	0,901	0,378	0,580	0,360	1,284	0,968	1,236
D137N	Likely pathogenic		24,30	0,830	0,540	0,186	0,193	0,942	0,297	0,378	0,140	0,360	0,125	0,162	0,333
D137V	Likely pathogenic		25,30	0,928	0,487	0,644	0,346	0,937	1,079	0,378	1,190	0,360	1,233	1,710	0,988
D137Y	Likely pathogenic		24,30	0,961	0,540	0,630	0,344	0,943	0,326	0,378	0,960	0,360	1,291	1,441	1,124
T138A	VUS		20,50	0,466	0,271	0,071	-0,051	0,229	0,103	0,355	0,220	0,760	-3,296	-0,361	-2,653
T138I	VUS		18,33	0,387	0,304	0,094	-0,026	0,287	-0,349	0,355	0,700	0,760	-3,334	0,184	-2,781
T138N	VUS		17,41	0,403	0,290	0,077	0,008	0,355	0,301	0,355	0,390	0,760	-3,232	-0,187	-2,657
T138P	VUS		22,60	0,615	0,272	0,191	0,202	0,517	0,017	0,355	0,300	0,760	-1,692	0,143	-1,412
T138S	VUS		16,42	0,395	0,270	0,077	-0,095	0,229	0,341	0,355	0,050	0,760	-3,823	-0,742	-3,054
P139A	VUS		16,42	0,524	0,377	0,066	0,038	0,360	0,887	0,610	0,520	1,010	-3,163	0,716	-2,761
P139H	VUS		21,40	0,454	0,418	0,154	0,091	0,332	1,241	0,610	0,350	1,010	-2,666	0,671	-2,246
P139L	VUS		21,70	0,580	0,429	0,101	0,099	0,347	0,754	0,610	1,070	1,010	-2,361	1,625	-2,167
P139R	Likely pathogenic		21,90	0,674	0,429	0,098	0,119	0,444	0,594	0,610	0,680	1,010	-2,015	1,244	-1,811
P139S	VUS		17,70	0,552	0,382	0,116	0,044	0,370	1,150	0,610	0,250	1,010	-2,933	0,456	-2,496
P139T	Likely pathogenic		20,90	0,656	0,405	0,094	0,062	0,357	1,051	0,610	0,300	1,010	-2,457	0,667	-2,091
D140A	Likely pathogenic		12,37	0,372	0,310	0,114	0,015	0,316	0,292	0,508	0,750	1,700	-3,970	0,963	-3,542
D140E	Likely pathogenic		3,00	0,348	0,319	0,142	-0,052	0,181	-0,360	0,508	0,290	1,700	-5,045	0,062	-4,328
D140G	Likely pathogenic		10,08	0,343	0,311	0,073	0,002	0,208	-0,594	0,508	0,460	1,700	-4,479	0,483	-3,897
D140H	Likely pathogenic	Mild	16,86	0,746	0,338	0,140	0,017	0,425	0,020	0,508	0,580	1,700	-2,702	1,125	-2,443

D140N	Likely pathogenic	13,31	0,288	0,330	0,076	-0,037	0,266	-0,412	0,508	0,140	1,700	-4,332	0,159	-3,665
D140V	Likely pathogenic	13,12	0,524	0,306	0,230	0,068	0,552	1,378	0,508	1,190	1,700	-2,955	1,730	-2,834
D140Y	Likely pathogenic	11,77	0,505	0,337	0,154	0,050	0,372	-0,660	0,508	0,960	1,700	-3,508	1,338	-3,207
D141A	Likely pathogenic	27,20	0,892	0,460	0,883	0,337	0,942	0,808	0,078	0,750	1,220	1,681	1,147	1,571
D141E	Likely pathogenic	22,90	0,721	0,480	0,931	0,214	0,902	2,360	0,078	0,290	1,220	0,828	0,308	1,016
D141G	Likely pathogenic	27,40	0,877	0,468	0,846	0,348	0,936	1,607	0,078	0,460	1,220	1,673	0,781	1,634
D141H	Likely pathogenic	22,50	0,891	0,519	0,938	0,346	0,869	5,565	0,078	0,580	1,220	1,645	0,789	1,629
D141N	Likely pathogenic	22,50	0,728	0,498	0,590	0,304	0,936	1,167	0,078	0,140	1,220	0,684	0,097	0,879
D141V	Likely pathogenic	27,00	0,904	0,454	0,922	0,316	0,939	3,227	0,078	1,190	1,220	1,796	1,662	1,570
D141Y	Likely pathogenic	22,50	0,847	0,502	0,891	0,314	0,947	4,663	0,078	0,960	1,220	1,499	1,227	1,400
F142C	Likely pathogenic	26,90	0,704	0,555	0,600	0,189	0,758	2,287	0,188	0,120	1,150	0,463	0,259	0,756
F142I	Likely pathogenic	24,80	0,674	0,530	0,305	0,081	0,684	1,729	0,188	0,100	1,150	-0,522	-0,014	-0,090
F142L	VUS	15,84	0,515	0,564	0,852	0,035	0,526	0,993	0,188	0,030	1,150	-1,151	-0,329	-0,504
F142S	Likely pathogenic	26,80	0,698	0,528	0,570	0,200	0,776	1,918	0,188	0,850	1,150	0,439	1,142	0,536
F142V	VUS	24,80	0,606	0,513	0,316	0,067	0,584	2,202	0,188	0,140	1,150	-0,838	-0,048	-0,354
F142Y	VUS	22,20	0,498	0,550	0,211	0,010	0,275	0,813	0,188	0,370	1,150	-1,893	-0,034	-1,224
Q143E	VUS	18,15	0,322	0,310	0,072	-0,072	0,160	0,126	0,604	0,400	1,660	-4,302	0,734	-3,734
Q143H	VUS	21,40	0,461	0,324	0,134	-0,039	0,479	0,294	0,604	0,470	1,660	-3,140	1,128	-2,805
Q143K	VUS	21,10	0,347	0,328	0,105	-0,028	0,299	-0,075	0,604	1,700	1,660	-3,567	2,508	-3,420
Q143L	VUS	21,80	0,377	0,302	0,106	-0,032	0,334	0,050	0,604	1,190	1,660	-3,510	1,918	-3,279
Q143P	VUS	21,90	0,518	0,317	0,126	0,133	0,671	-0,254	0,604	0,120	1,660	-2,372	0,902	-2,154
Q143R	VUS	18,43	0,451	0,323	0,082	-0,017	0,330	-0,076	0,604	0,560	1,660	-3,606	1,100	-3,221
L144F	Likely pathogenic	22,20	0,646	0,561	0,703	0,247	0,914	1,745	0,015	0,030	1,260	0,707	-0,177	1,044
L144M	Likely pathogenic	21,90	0,607	0,550	0,334	0,193	0,735	0,105	0,015	0,000	1,260	-0,289	-0,430	0,201
L144S	Likely pathogenic	25,30	0,859	0,584	0,948	0,417	0,940	3,542	0,015	0,820	1,260	2,234	1,148	2,140
L144V	Likely pathogenic	22,60	0,740	0,529	0,587	0,297	0,911	3,637	0,015	0,110	1,260	0,909	-0,057	1,140
L144W	Likely pathogenic	25,30	0,835	0,620	0,874	0,351	0,933	6,267	0,015	0,080	1,260	2,083	0,164	2,228
H145D	VUS	8,86	0,282	0,310	0,096	-0,062	0,156	0,773	0,621	0,580	2,230	-5,087	1,043	-4,533
H145L	VUS	13,28	0,278	0,288	0,082	-0,054	0,137	0,063	0,621	0,720	2,230	-4,875	1,327	-4,391
H145N	VUS	8,55	0,271	0,302	0,070	-0,095	0,221	0,103	0,621	0,440	2,230	-5,196	0,857	-4,602
H145P	VUS	13,67	0,367	0,296	0,177	0,150	0,390	2,246	0,621	0,350	2,230	-3,628	1,141	-3,325
H145Q	VUS	10,17	0,357	0,293	0,080	-0,135	0,216	0,035	0,621	0,470	2,230	-5,031	0,958	-4,469
H145R	VUS	11,60	0,268	0,303	0,051	-0,069	0,121	-0,402	0,621	1,030	2,230	-5,068	1,642	-4,616
H145Y	VUS	10,06	0,302	0,308	0,094	-0,109	0,210	0,407	0,621	0,380	2,230	-5,031	0,837	-4,434
N146D	VUS	17,12	0,421	0,429	0,075	0,032	0,283	1,527	0,441	0,140	1,990	-3,273	0,430	-2,700
N146H	VUS	15,29	0,390	0,393	0,057	0,018	0,273	0,304	0,441	0,440	1,990	-3,641	0,710	-3,110
N146I	Likely pathogenic	23,80	0,659	0,397	0,281	0,166	0,761	1,620	0,441	1,090	1,990	-1,089	2,178	-1,178
N146K	VUS	23,30	0,518	0,444	0,180	0,028	0,428	0,489	0,441	1,730	1,990	-2,219	2,686	-2,164
N146S	VUS	22,10	0,537	0,356	0,067	-0,002	0,450	0,695	0,441	0,340	1,990	-2,736	0,884	-2,362
N146T	VUS	22,30	0,537	0,385	0,132	0,048	0,516	0,798	0,441	0,390	1,990	-2,356	1,030	-2,043
N146Y	VUS	22,40	0,517	0,421	0,126	0,071	0,703	0,584	0,441	0,820	1,990	-1,951	1,640	-1,801
F147C	Likely pathogenic	27,30	0,772	0,605	0,954	0,394	0,852	3,658	0,121	0,120	0,510	2,036	0,054	2,200
F147I	Likely pathogenic	28,00	0,910	0,582	0,886	0,370	0,886	2,409	0,121	0,100	0,510	2,158	0,097	2,272
F147L	Likely pathogenic	28,30	0,884	0,611	0,986	0,264	0,903	1,523	0,121	0,030	0,510	2,040	0,002	2,248
F147S	Likely pathogenic	27,30	0,746	0,587	0,960	0,405	0,936	5,393	0,121	0,850	0,510	2,229	0,949	2,170
F147V	Likely pathogenic	28,10	0,923	0,567	0,878	0,364	0,920	3,331	0,121	0,140	0,510	2,223	0,147	2,301
F147Y	Likely pathogenic	25,70	0,735	0,599	0,616	0,232	0,862	2,454	0,121	0,370	0,510	1,062	0,121	1,315
S148C	VUS	26,00	0,581	0,378	0,106	0,016	0,712	0,709	0,477	0,730	0,270	-1,526	0,691	-1,283
S148G	VUS	22,50	0,498	0,354	0,108	-0,033	0,355	0,622	0,477	0,020	0,270	-2,691	-0,474	-2,065
S148I	VUS	22,00	0,466	0,393	0,114	0,044	0,520	0,942	0,477	0,750	0,270	-2,188	0,503	-1,816
S148N	VUS	17,86	0,401	0,401	0,114	-0,036	0,328	0,568	0,477	0,340	0,270	-3,108	-0,249	-2,454
S148R	VUS	20,10	0,537	0,403	0,198	0,040	0,471	0,077	0,477	0,930	0,270	-2,177	0,722	-1,839
S148T	VUS	17,51	0,390	0,376	0,074	-0,070	0,293	0,915	0,477	0,050	0,270	-3,400	-0,674	-2,645
L149F	Likely pathogenic	25,90	0,852	0,485	0,525	0,345	0,903	1,923	0,129	0,030	-0,190	1,341	-0,610	1,554

L149H	Likely pathogenic		26,20	0,842	0,559	0,927	0,454	0,949	3,703	0,129	0,720	-0,190	2,417	0,444	2,373
L149I	Likely pathogenic		25,40	0,691	0,499	0,268	0,286	0,708	1,320	0,129	0,070	-0,190	0,294	-0,818	0,699
L149P	Likely pathogenic		26,50	0,867	0,590	0,862	0,427	0,942	1,439	0,129	1,070	-0,190	2,345	0,888	2,254
L149R	Likely pathogenic		26,50	0,858	0,557	0,877	0,492	0,943	3,053	0,129	1,750	-0,190	2,539	1,728	2,221
L149V	Likely pathogenic		25,30	0,802	0,454	0,344	0,322	0,807	1,836	0,129	0,110	-0,190	0,732	-0,664	0,998
P150A	VUS		11,06	0,243	0,300	0,076	0,000	0,191	2,137	0,182	0,520	0,090	-3,770	-1,095	-2,943
P150L	VUS		14,63	0,279	0,329	0,093	0,078	0,239	2,511	0,182	1,070	0,090	-3,048	-0,232	-2,453
P150Q	VUS		13,49	0,282	0,300	0,136	0,048	0,263	1,926	0,182	0,120	0,090	-3,275	-1,427	-2,443
P150R	VUS		14,47	0,324	0,327	0,141	0,088	0,221	1,630	0,182	0,680	0,090	-2,993	-0,672	-2,318
P150S	VUS		13,96	0,298	0,291	0,113	0,015	0,165	0,894	0,182	0,250	0,090	-3,518	-1,301	-2,668
P150T	VUS		12,12	0,322	0,301	0,084	0,017	0,170	2,505	0,182	0,300	0,090	-3,526	-1,294	-2,689
E151A	VUS		23,30	0,536	0,333	0,104	-0,047	0,468	0,327	0,502	1,040	1,170	-2,632	1,409	-2,390
E151D	VUS		12,80	0,291	0,334	0,117	-0,095	0,253	0,888	0,502	0,290	1,170	-4,288	-0,022	-3,583
E151G	VUS		22,70	0,426	0,321	0,134	-0,009	0,449	1,016	0,502	0,750	1,170	-2,809	0,996	-2,477
E151K	VUS		18,11	0,445	0,386	0,085	-0,036	0,324	-0,608	0,502	1,300	1,170	-3,272	1,518	-2,942
E151Q	VUS		17,98	0,473	0,322	0,102	-0,079	0,462	-0,021	0,502	0,400	1,170	-3,301	0,426	-2,818
E151V	VUS		22,60	0,465	0,321	0,148	-0,032	0,508	0,394	0,502	1,480	1,170	-2,663	1,922	-2,528
E152A	Likely pathogenic		27,10	0,873	0,504	0,574	0,309	0,923	1,072	0,099	1,040	0,200	1,511	0,847	1,468
E152D	Likely pathogenic		23,50	0,682	0,503	0,652	0,195	0,734	3,401	0,099	0,290	0,200	0,464	-0,389	0,826
E152G	Likely pathogenic		28,20	0,848	0,476	0,701	0,335	0,919	2,432	0,099	0,750	0,200	1,705	0,538	1,690
E152K	Likely pathogenic		24,90	0,874	0,571	0,797	0,331	0,931	0,758	0,099	1,300	0,200	1,836	1,213	1,745
E152Q	Likely pathogenic		24,20	0,862	0,493	0,671	0,281	0,866	0,394	0,099	0,400	0,200	1,141	-0,027	1,310
E152V	Likely pathogenic		27,70	0,873	0,486	0,780	0,322	0,939	2,950	0,099	1,480	0,200	1,905	1,451	1,703
D153A	Likely pathogenic		29,80	0,813	0,505	0,958	0,349	0,942	2,693	0,000	0,750	0,080	2,343	0,393	2,352
D153E	Likely pathogenic		22,60	0,654	0,526	0,948	0,293	0,838	4,479	0,000	0,290	0,080	1,323	-0,523	1,634
D153G	Likely pathogenic		30,00	0,832	0,515	0,946	0,360	0,956	4,063	0,000	0,460	0,080	2,484	0,054	2,540
D153H	Likely pathogenic		27,70	0,826	0,560	0,987	0,358	0,936	19,234	0,000	0,580	0,080	3,060	0,030	3,048
D153N	Likely pathogenic		27,80	0,760	0,542	0,927	0,315	0,930	1,233	0,000	0,140	0,080	1,952	-0,439	2,197
D153V	Likely pathogenic		29,50	0,845	0,499	0,951	0,396	0,942	1,570	0,000	1,190	0,080	2,459	0,969	2,326
D153Y	Likely pathogenic		27,80	0,822	0,546	0,968	0,394	0,932	24,321	0,000	0,960	0,080	3,321	0,460	3,164
T154A	VUS		20,60	0,350	0,326	0,078	-0,019	0,278	-0,485	0,256	0,220	0,060	-2,970	-0,958	-2,205
T154I	VUS		19,54	0,281	0,351	0,074	-0,014	0,243	-1,599	0,256	0,700	0,060	-3,172	-0,426	-2,462
T154N	VUS		19,24	0,320	0,347	0,110	0,037	0,329	0,320	0,256	0,390	0,060	-2,785	-0,744	-2,090
T154P	VUS		21,90	0,396	0,324	0,228	0,221	0,516	1,975	0,256	0,300	0,060	-1,594	-0,566	-1,137
T154S	VUS		18,88	0,364	0,324	0,107	-0,047	0,255	0,394	0,256	0,050	0,060	-3,116	-1,230	-2,284
K155E	VUS		19,01	0,420	0,367	0,112	-0,025	0,264	0,818	0,483	1,300	0,110	-3,040	0,853	-2,626
K155M	VUS		22,10	0,501	0,335	0,166	0,054	0,629	0,917	0,483	2,890	0,110	-1,828	3,093	-2,057
K155N	VUS		18,37	0,343	0,378	0,251	-0,034	0,301	0,781	0,483	1,730	0,110	-2,986	1,375	-2,654
K155Q	VUS		18,16	0,422	0,339	0,087	-0,051	0,411	-0,045	0,483	1,700	0,110	-3,038	1,344	-2,759
K155R	VUS		18,49	0,361	0,326	0,066	-0,116	0,192	-0,368	0,483	1,140	0,110	-3,721	0,519	-3,166
K155T	VUS		19,32	0,422	0,372	0,114	-0,018	0,464	1,347	0,483	2,120	0,110	-2,603	1,935	-2,469
L156F	VUS		19,15	0,335	0,442	0,128	0,068	0,231	-0,698	0,174	0,030	-0,160	-2,501	-1,438	-1,614
L156H	VUS		23,80	0,540	0,504	0,337	0,205	0,754	1,177	0,174	0,720	-0,160	-0,169	-0,039	0,161
L156I	VUS		22,60	0,435	0,455	0,213	0,037	0,540	0,883	0,174	0,070	-0,160	-1,491	-1,139	-0,782
L156P	Likely pathogenic		24,00	0,778	0,541	0,918	0,314	0,890	3,109	0,174	1,070	-0,160	1,599	0,785	1,597
L156R	VUS		23,90	0,641	0,508	0,284	0,243	0,843	0,581	0,174	1,750	-0,160	0,256	1,315	0,247
L156V	VUS		21,60	0,436	0,403	0,194	0,068	0,546	1,212	0,174	0,110	-0,160	-1,600	-1,127	-0,938
K157E	Likely pathogenic		23,00	0,819	0,498	0,948	0,254	0,662	7,295	0,000	1,300	-0,680	1,584	0,248	1,698
K157M	Likely pathogenic		24,20	0,851	0,444	0,905	0,239	0,911	1,031	0,000	2,890	-0,680	1,748	2,336	1,387
K157N	Likely pathogenic	Severe	22,80	0,646	0,523	0,985	0,315	0,893	3,311	0,000	1,730	-0,680	1,699	0,849	1,687
K157Q	Likely pathogenic	Severe	23,00	0,892	0,467	0,809	0,218	0,751	2,584	0,000	1,700	-0,680	1,377	0,770	1,378
K157R	Likely pathogenic		22,60	0,804	0,441	0,237	0,144	0,616	-1,900	0,000	1,140	-0,680	-0,116	-0,195	0,207
K157T	Likely pathogenic		23,90	0,866	0,512	0,907	0,319	0,899	3,976	0,000	2,120	-0,680	2,157	1,437	1,948
I158K	VUS		26,90	0,906	0,685	0,960	0,486	0,906	3,293	0,020	2,820	-0,550	3,294	2,698	2,828

I158L	VUS		26,30	0,795	0,636	0,265	0,186	0,726	1,300	0,020	0,070	-0,550	0,884	-1,167	1,424
I158M	VUS		23,90	0,779	0,610	0,324	0,201	0,789	0,414	0,020	0,070	-0,550	0,781	-1,198	1,303
I158R	VUS		27,10	0,912	0,670	0,957	0,512	0,914	2,991	0,020	1,680	-0,550	3,261	1,326	3,044
I158T	VUS		26,60	0,923	0,714	0,965	0,345	0,910	2,913	0,020	0,700	-0,550	2,878	0,047	3,022
I158V	VUS		24,80	0,693	0,577	0,371	0,151	0,537	1,199	0,020	0,040	-0,550	0,189	-1,372	0,845
P159A	Likely pathogenic		23,90	0,844	0,504	0,354	0,290	0,784	2,476	0,314	0,520	-0,470	0,594	0,028	0,750
P159H	Likely pathogenic		25,90	0,910	0,547	0,548	0,256	0,840	2,930	0,314	0,350	-0,470	1,184	-0,021	1,344
P159L	Likely pathogenic	Mild	26,10	0,915	0,563	0,493	0,339	0,794	2,148	0,314	1,070	-0,470	1,335	0,894	1,297
P159R	Likely pathogenic		26,00	0,919	0,560	0,482	0,360	0,893	2,049	0,314	0,680	-0,470	1,482	0,458	1,489
P159S	Likely pathogenic		24,40	0,930	0,505	0,435	0,210	0,886	2,836	0,314	0,250	-0,470	0,838	-0,234	1,028
P159T	Likely pathogenic	Mild	24,20	0,904	0,530	0,473	0,302	0,860	2,502	0,314	0,300	-0,470	1,048	-0,126	1,199
L160M	VUS		20,60	0,588	0,468	0,208	0,011	0,455	0,482	0,000	0,000	-0,500	-1,219	-1,793	-0,391
L160P	Likely pathogenic		25,20	0,911	0,575	0,816	0,289	0,939	5,975	0,000	1,070	-0,500	2,341	0,296	2,373
L160Q	Likely pathogenic		24,90	0,852	0,488	0,667	0,153	0,877	3,216	0,000	1,190	-0,500	1,314	0,254	1,436
L160R	Likely pathogenic		25,00	0,882	0,541	0,799	0,286	0,927	6,039	0,000	1,750	-0,500	2,198	1,082	2,069
L160V	VUS		18,87	0,583	0,437	0,114	0,024	0,270	3,907	0,000	0,110	-0,500	-1,621	-1,832	-0,770
I161F	Likely pathogenic		23,40	0,852	0,594	0,375	0,166	0,781	5,509	0,000	0,100	-0,810	1,098	-1,387	1,601
I161L	Likely pathogenic		18,24	0,507	0,583	0,087	0,057	0,322	0,132	0,000	0,070	-0,810	-1,434	-1,998	-0,470
I161M	Likely pathogenic		22,80	0,702	0,556	0,162	0,076	0,638	0,240	0,000	0,070	-0,810	-0,203	-1,646	0,506
I161N	Likely pathogenic		25,00	0,900	0,652	0,936	0,315	0,934	3,404	0,000	1,090	-0,810	2,633	0,230	2,714
I161S	Likely pathogenic		25,10	0,929	0,606	0,875	0,318	0,926	4,902	0,000	0,750	-0,810	2,549	-0,219	2,677
I161T	Likely pathogenic		24,10	0,890	0,672	0,848	0,235	0,801	3,177	0,000	0,700	-0,810	2,073	-0,382	2,373
I161V	Likely pathogenic		19,23	0,602	0,519	0,204	0,033	0,374	1,336	0,000	0,040	-0,810	-1,140	-1,957	-0,259
H162D	Likely pathogenic		22,50	0,684	0,495	0,258	0,118	0,625	1,137	0,223	0,580	-0,250	-0,555	-0,245	-0,147
H162L	Likely pathogenic		22,10	0,467	0,466	0,112	0,029	0,323	-0,500	0,223	0,720	-0,250	-1,951	-0,386	-1,317
H162N	Likely pathogenic		22,10	0,498	0,489	0,138	-0,005	0,544	0,884	0,223	0,440	-0,250	-1,522	-0,650	-0,891
H162P	Likely pathogenic		24,40	0,788	0,446	0,480	0,234	0,695	3,678	0,223	0,350	-0,250	0,374	-0,311	0,635
H162Q	Likely pathogenic		16,89	0,341	0,470	0,120	-0,065	0,173	0,473	0,223	0,470	-0,250	-2,965	-1,006	-2,073
H162R	Likely pathogenic		21,30	0,450	0,499	0,066	0,013	0,287	0,298	0,223	1,030	-0,250	-2,047	-0,065	-1,439
H162Y	Likely pathogenic		22,40	0,624	0,497	0,106	-0,025	0,599	0,990	0,223	0,380	-0,250	-1,244	-0,652	-0,653
R163G	Likely pathogenic		24,00	0,672	0,251	0,144	0,116	0,620	2,869	0,197	0,910	0,060	-1,124	0,171	-0,921
R163L	VUS		20,90	0,516	0,251	0,170	0,070	0,498	0,223	0,197	1,750	0,060	-1,949	0,997	-1,779
R163P	Likely pathogenic		22,70	0,756	0,260	0,858	0,290	0,806	3,213	0,197	0,680	0,060	0,448	0,262	0,462
R163Q	VUS		17,27	0,352	0,244	0,063	0,024	0,306	0,897	0,197	0,560	0,060	-3,106	-0,770	-2,455
A164D	Likely pathogenic		25,50	0,848	0,514	0,953	0,342	0,896	2,482	0,000	0,750	-0,110	2,066	0,165	2,136
A164G	Likely pathogenic		25,10	0,787	0,450	0,344	0,254	0,736	2,107	0,000	0,290	-0,110	0,598	-0,740	0,936
A164P	Likely pathogenic		23,30	0,867	0,493	0,944	0,382	0,906	2,663	0,000	0,520	-0,110	1,986	-0,155	2,084
A164S	Likely pathogenic		22,70	0,783	0,440	0,167	0,141	0,662	1,061	0,000	0,270	-0,110	-0,222	-0,967	0,251
A164T	Likely pathogenic		23,10	0,821	0,466	0,210	0,155	0,785	0,649	0,000	0,220	-0,110	0,182	-0,923	0,606
A164V	VUS		23,30	0,610	0,467	0,147	0,143	0,577	3,012	0,000	0,440	-0,110	-0,498	-0,867	0,029
L165M	VUS		14,75	0,392	0,390	0,108	-0,041	0,266	0,149	0,289	0,000	0,100	-3,231	-1,266	-2,359
L165P	Likely pathogenic		22,80	0,807	0,443	0,750	0,243	0,831	4,247	0,289	1,070	0,100	0,727	0,993	0,689
L165Q	VUS		18,04	0,475	0,397	0,082	0,076	0,290	1,801	0,289	1,190	0,100	-2,382	0,375	-1,942
L165R	Likely pathogenic		19,24	0,760	0,435	0,103	0,158	0,692	1,072	0,289	1,750	0,100	-0,835	1,444	-0,829
L165V	VUS		13,91	0,439	0,373	0,108	-0,020	0,261	1,954	0,289	0,110	0,100	-3,117	-1,144	-2,311
Q166E	VUS		2,82	0,268	0,301	0,068	-0,049	0,121	-0,010	0,667	0,400	1,120	-5,498	0,091	-4,777
Q166H	VUS		7,89	0,244	0,307	0,129	-0,028	0,349	-0,701	0,667	0,470	1,120	-4,736	0,421	-4,157
Q166K	VUS		5,56	0,296	0,314	0,080	-0,020	0,116	-1,383	0,667	1,700	1,120	-5,105	1,805	-4,736
Q166L	VUS		12,31	0,290	0,297	0,112	0,063	0,294	-0,270	0,667	1,190	1,120	-4,172	1,467	-3,862
Q166P	VUS		11,82	0,369	0,305	0,254	0,218	0,390	2,587	0,667	0,120	1,120	-3,331	0,322	-2,940
Q166R	VUS		9,84	0,302	0,308	0,078	-0,013	0,151	-1,746	0,667	0,560	1,120	-4,838	0,549	-4,245
L167F	VUS		17,21	0,359	0,404	0,147	0,059	0,341	-0,140	0,219	0,030	0,420	-2,673	-1,035	-1,895
L167M	VUS		0,89	0,246	0,392	0,080	-0,002	0,161	-0,245	0,219	0,000	0,420	-4,560	-1,700	-3,500
L167S	VUS		19,49	0,424	0,416	0,246	0,236	0,491	1,500	0,219	0,820	0,420	-1,478	0,195	-1,110

L167V	VUS		2,55	0,301	0,382	0,098	0,020	0,167	3,474	0,219	0,110	0,420	-4,118	-1,508	-3,161	
L167W	VUS		22,00	0,410	0,433	0,210	0,238	0,642	1,603	0,219	0,080	0,420	-1,147	-0,598	-0,660	
A168D	VUS		21,70	0,450	0,378	0,386	0,230	0,495	3,067	0,132	0,750	0,800	-1,113	0,236	-0,783	
A168G	VUS		21,30	0,415	0,340	0,124	0,069	0,384	1,085	0,132	0,290	0,800	-2,253	-0,556	-1,642	
A168P	VUS		14,74	0,434	0,360	0,474	0,270	0,331	4,835	0,132	0,520	0,800	-1,671	-0,277	-1,213	
A168S	VUS		0,34	0,327	0,335	0,059	0,005	0,175	-0,043	0,132	0,270	0,800	-4,492	-1,326	-3,554	
A168T	VUS		4,03	0,331	0,341	0,081	0,022	0,175	-0,303	0,132	0,220	0,800	-4,162	-1,262	-3,250	
A168V	VUS		21,50	0,460	0,354	0,153	0,037	0,464	1,367	0,132	0,440	0,800	-2,026	-0,324	-1,477	
Q169E	VUS		14,84	0,341	0,260	0,075	-0,076	0,223	0,225	0,662	0,400	1,560	-4,587	0,710	-4,059	
Q169H	VUS		7,98	0,307	0,261	0,111	-0,072	0,300	0,374	0,662	0,470	1,560	-4,956	0,627	-4,411	
Q169K	VUS		13,24	0,332	0,264	0,068	-0,061	0,200	-0,216	0,662	1,700	1,560	-4,634	2,255	-4,402	
Q169L	VUS		13,15	0,284	0,257	0,120	0,028	0,266	-0,064	0,662	1,190	1,560	-4,410	1,688	-4,122	
Q169P	VUS		10,78	0,270	0,263	0,062	0,082	0,162	-1,125	0,662	0,120	1,560	-4,804	0,295	-4,213	
Q169R	VUS		10,35	0,299	0,260	0,058	-0,054	0,181	-0,531	0,662	0,560	1,560	-5,029	0,760	-4,481	
R170C	Likely pathogenic	Mild	23,50	0,831	0,361	0,107	0,079	0,918	2,063	0,142	1,660	0,770	-0,323	1,573	-0,422	
R170G	Likely pathogenic		21,50	0,509	0,328	0,105	0,079	0,541	2,941	0,142	0,910	0,770	-1,740	0,291	-1,397	
R170H	Likely pathogenic		18,44	0,342	0,330	0,060	0,021	0,385	0,735	0,142	1,030	0,770	-2,769	0,192	-2,257	
R170L	Likely pathogenic		18,43	0,448	0,332	0,121	0,064	0,447	1,708	0,142	1,750	0,770	-2,206	1,179	-1,969	
R170P	Likely pathogenic	Mild	18,94	0,656	0,343	0,277	0,192	0,707	1,539	0,142	0,680	0,770	-0,970	0,198	-0,736	Yes
R170S	Likely pathogenic		21,30	0,562	0,327	0,184	0,053	0,578	2,861	0,142	0,930	0,770	-1,577	0,358	-1,260	
P171A	VUS		19,22	0,403	0,359	0,112	0,045	0,392	0,727	0,673	0,520	0,050	-3,025	0,330	-2,577	
P171H	VUS		18,41	0,419	0,382	0,114	0,079	0,296	0,809	0,673	0,350	0,050	-3,067	0,102	-2,554	
P171L	VUS		21,60	0,479	0,388	0,242	0,094	0,629	0,605	0,673	1,070	0,050	-1,994	1,265	-1,840	
P171R	VUS		21,30	0,454	0,390	0,094	0,106	0,316	0,508	0,673	0,680	0,050	-2,694	0,625	-2,315	
P171S	VUS		17,24	0,372	0,359	0,095	0,027	0,282	0,698	0,673	0,250	0,050	-3,471	-0,123	-2,877	
P171T	VUS		19,76	0,384	0,375	0,109	0,047	0,470	0,557	0,673	0,300	0,050	-2,893	0,101	-2,409	
V172A	VUS		22,10	0,389	0,349	0,305	0,183	0,560	2,523	0,138	0,440	-0,590	-1,151	-0,988	-0,625	
V172D	VUS		23,50	0,525	0,352	0,798	0,282	0,778	4,413	0,138	1,190	-0,590	0,452	0,292	0,548	
V172F	VUS		6,29	0,378	0,341	0,221	0,179	0,573	6,705	0,138	0,140	-0,590	-2,236	-1,860	-1,532	
V172G	VUS		23,30	0,492	0,317	0,466	0,309	0,667	3,559	0,138	0,730	-0,590	-0,242	-0,417	0,016	
V172I	VUS		0,76	0,340	0,306	0,068	0,016	0,275	-1,033	0,138	0,040	-0,590	-4,112	-2,341	-3,077	
V172L	VUS		0,67	0,336	0,341	0,129	0,075	0,263	-1,451	0,138	0,110	-0,590	-3,865	-2,196	-2,864	
S173A	VUS		17,78	0,525	0,547	0,093	-0,046	0,278	-0,286	0,084	0,270	-0,670	-1,970	-1,586	-1,034	
S173L	VUS		22,00	0,525	0,570	0,108	0,020	0,627	-2,569	0,084	0,820	-0,670	-0,973	-0,607	-0,350	
S173P	Likely pathogenic		22,20	0,689	0,561	0,361	0,116	0,821	1,870	0,084	0,250	-0,670	0,269	-1,066	0,788	
S173T	VUS		17,60	0,475	0,558	0,129	-0,057	0,465	-0,003	0,084	0,050	-0,670	-1,762	-1,814	-0,813	
L174F	Likely pathogenic		22,40	0,820	0,606	0,254	0,146	0,812	3,899	0,005	0,030	-1,030	0,828	-1,650	1,405	
L174H	Likely pathogenic		24,90	0,915	0,678	0,901	0,353	0,958	3,306	0,005	0,720	-1,030	2,810	-0,305	2,971	
L174I	Likely pathogenic		23,10	0,697	0,621	0,160	0,089	0,670	3,673	0,005	0,070	-1,030	0,219	-1,742	0,930	
L174P	Likely pathogenic		25,20	0,926	0,705	0,870	0,394	0,960	2,205	0,005	1,070	-1,030	2,963	0,171	3,027	
L174R	Likely pathogenic		25,20	0,926	0,681	0,873	0,391	0,946	5,108	0,005	1,750	-1,030	3,050	0,963	2,929	
L174V	Likely pathogenic		23,10	0,789	0,577	0,173	0,140	0,837	3,621	0,005	0,110	-1,030	0,676	-1,576	1,230	
L175F	VUS		9,79	0,296	0,385	0,039	0,040	0,237	2,639	0,000	0,030	-1,040	-2,977	-2,673	-1,894	
L175H	Likely pathogenic		22,80	0,681	0,435	0,582	0,256	0,717	4,795	0,000	0,720	-1,040	0,766	-0,833	1,094	
L175I	VUS		12,81	0,342	0,416	0,101	-0,004	0,286	0,733	0,000	0,070	-1,040	-2,639	-2,477	-1,578	
L175P	Likely pathogenic		23,00	0,859	0,422	0,872	0,297	0,807	6,031	0,000	1,070	-1,040	1,718	-0,190	1,799	
L175R	Likely pathogenic		22,80	0,749	0,436	0,778	0,294	0,783	5,811	0,000	1,750	-1,040	1,421	0,551	1,406	
L175V	VUS		13,44	0,346	0,380	0,105	0,030	0,245	0,722	0,000	0,110	-1,040	-2,646	-2,418	-1,623	
A176D	Likely pathogenic		26,60	0,932	0,646	0,987	0,301	0,941	6,732	0,000	0,750	-1,180	3,012	-0,360	3,158	
A176G	Likely pathogenic		22,50	0,680	0,555	0,140	0,111	0,489	1,339	0,000	0,290	-1,180	-0,294	-1,657	0,426	
A176P	Likely pathogenic		23,30	0,923	0,612	0,953	0,341	0,949	6,738	0,000	0,520	-1,180	2,733	-0,737	2,925	
A176S	Likely pathogenic		22,80	0,796	0,538	0,290	0,094	0,714	2,322	0,000	0,270	-1,180	0,406	-1,520	0,989	
A176T	Likely pathogenic		23,10	0,834	0,567	0,532	0,100	0,839	3,207	0,000	0,220	-1,180	1,070	-1,434	1,589	
A176V	Likely pathogenic		26,40	0,863	0,585	0,677	0,187	0,861	4,060	0,000	0,440	-1,180	1,854	-0,961	2,214	

S177C	Likely pathogenic		25,00	0,892	0,611	0,556	0,176	0,936	1,841	0,000	0,730	-1,290	1,772	-0,682	2,081	
S177G	Likely pathogenic		24,40	0,862	0,567	0,675	0,126	0,869	1,111	0,000	0,020	-1,290	1,402	-1,609	1,932	
S177I	Likely pathogenic		25,10	0,860	0,628	0,893	0,274	0,954	3,245	0,000	0,750	-1,290	2,454	-0,518	2,678	
S177N	Likely pathogenic		22,70	0,671	0,647	0,951	0,215	0,892	5,470	0,000	0,340	-1,290	1,863	-1,226	2,329	
S177R	Likely pathogenic		24,90	0,925	0,650	0,997	0,287	0,956	13,921	0,000	0,930	-1,290	3,216	-0,313	3,308	
S177T	VUS		22,60	0,629	0,609	0,319	0,088	0,811	1,189	0,000	0,050	-1,290	0,362	-1,865	1,076	
P178A	Likely pathogenic		22,20	0,610	0,563	0,348	0,088	0,540	2,244	0,000	0,520	-1,380	-0,086	-1,475	0,602	
P178H	Likely pathogenic		28,20	0,860	0,615	0,980	0,230	0,865	26,063	0,000	0,350	-1,380	3,425	-1,196	3,650	
P178L	Likely pathogenic		28,70	0,895	0,636	0,865	0,240	0,809	5,722	0,000	1,070	-1,380	2,607	-0,155	2,784	
P178R	Likely pathogenic		28,20	0,888	0,626	0,979	0,193	0,858	12,398	0,000	0,680	-1,380	2,868	-0,684	3,109	
P178S	Likely pathogenic	Severe	25,30	0,819	0,562	0,629	0,100	0,715	1,950	0,000	0,250	-1,380	1,097	-1,465	1,656	
P178T	Likely pathogenic		25,10	0,792	0,598	0,792	0,114	0,829	3,295	0,000	0,300	-1,380	1,561	-1,326	2,066	
W179C	Likely pathogenic		26,70	0,929	0,744	0,992	0,509	0,954	1,308	0,004	0,010	-1,330	3,514	-1,139	3,790	
W179G	Likely pathogenic		26,00	0,923	0,715	0,959	0,490	0,947	1,860	0,004	0,760	-1,330	3,370	-0,283	3,472	
W179L	Likely pathogenic		25,80	0,921	0,753	0,965	0,520	0,956	-0,573	0,004	0,080	-1,330	3,386	-1,064	3,664	
W179R	Likely pathogenic		25,40	0,909	0,779	0,998	0,557	0,957	1,420	0,004	1,670	-1,330	3,722	0,887	3,595	
W179S	Likely pathogenic		26,50	0,897	0,737	0,961	0,571	0,952	1,910	0,004	0,740	-1,330	3,614	-0,251	3,685	
T180A	Likely pathogenic		23,60	0,720	0,448	0,466	0,130	0,667	2,555	0,017	0,220	-1,090	0,279	-1,493	0,827	
T180I	Likely pathogenic		25,40	0,826	0,532	0,932	0,170	0,856	6,100	0,017	0,700	-1,090	1,882	-0,577	2,148	
T180K	Likely pathogenic		25,70	0,833	0,511	0,957	0,295	0,856	2,162	0,017	2,120	-1,090	2,137	1,271	1,987	
T180P	Likely pathogenic		26,60	0,893	0,441	0,898	0,376	0,933	3,789	0,017	0,300	-1,090	2,325	-0,887	2,472	
T180R	Likely pathogenic		25,60	0,869	0,502	0,934	0,298	0,893	10,323	0,017	0,980	-1,090	2,465	-0,172	2,515	
T180S	VUS		21,60	0,476	0,445	0,237	0,067	0,343	1,121	0,017	0,050	-1,090	-1,293	-2,078	-0,413	
S181A	VUS		16,68	0,400	0,497	0,074	0,033	0,259	-0,792	0,000	0,270	-0,970	-2,108	-2,007	-1,120	
S181L	Likely pathogenic		25,00	0,838	0,523	0,664	0,151	0,808	6,012	0,000	0,820	-0,970	1,446	-0,510	1,723	
S181P	Likely pathogenic		22,30	0,674	0,523	0,292	0,198	0,596	-1,951	0,000	0,250	-0,970	-0,039	-1,451	0,580	
S181T	Likely pathogenic		21,90	0,653	0,512	0,469	0,065	0,561	2,953	0,000	0,050	-0,970	-0,125	-1,796	0,606	
P182A	Likely pathogenic		23,80	0,889	0,659	0,688	0,248	0,934	3,376	0,000	0,520	-0,930	2,089	-0,681	2,392	
P182H	Likely pathogenic		25,70	0,942	0,703	0,975	0,354	0,953	34,180	0,000	0,350	-0,930	4,280	-0,900	4,345	
P182L	Likely pathogenic	Severe	26,00	0,953	0,718	0,929	0,364	0,961	8,766	0,000	1,070	-0,930	3,353	0,206	3,378	
P182R	Likely pathogenic		25,70	0,955	0,714	0,974	0,385	0,957	11,033	0,000	0,680	-0,930	3,487	-0,275	3,579	
P182S	Likely pathogenic		24,20	0,881	0,661	0,828	0,247	0,922	4,517	0,000	0,250	-0,930	2,270	-0,977	2,628	
P182T	Likely pathogenic		24,00	0,958	0,687	0,908	0,260	0,960	5,701	0,000	0,300	-0,930	2,690	-0,840	2,986	
T183A	VUS		15,30	0,338	0,420	0,059	-0,027	0,221	-0,280	0,250	0,220	-0,690	-3,105	-1,551	-2,170	
T183I	VUS		23,10	0,656	0,524	0,106	0,015	0,432	-0,578	0,250	0,700	-0,690	-1,212	-0,436	-0,638	
T183N	VUS		24,20	0,591	0,480	0,149	0,136	0,669	0,181	0,250	0,390	-0,690	-0,664	-0,681	-0,185	
T183P	VUS		21,60	0,626	0,424	0,068	0,148	0,463	-2,594	0,250	0,300	-0,690	-1,402	-0,933	-0,830	
T183S	VUS		22,00	0,501	0,427	0,107	-0,044	0,316	0,642	0,250	0,050	-0,690	-2,127	-1,459	-1,296	
W184C	Likely pathogenic		26,70	0,909	0,743	0,986	0,499	0,946	4,449	0,053	0,010	-0,610	3,350	-0,668	3,554	
W184G	Likely pathogenic		25,90	0,827	0,714	0,936	0,548	0,947	5,064	0,053	0,760	-0,610	3,214	0,182	3,230	
W184L	Likely pathogenic		25,70	0,885	0,745	0,968	0,510	0,947	4,337	0,053	0,080	-0,610	3,248	-0,619	3,448	
W184R	Pathogenic	Severe	25,00	0,787	0,772	0,988	0,464	0,950	4,463	0,053	1,670	-0,610	3,116	1,248	3,009	Yes
W184S	Likely pathogenic		26,30	0,901	0,735	0,944	0,561	0,937	3,986	0,053	0,740	-0,610	3,411	0,229	3,411	
L185F	Likely pathogenic		24,60	0,777	0,569	0,668	0,274	0,659	5,660	0,000	0,030	-0,350	1,290	-1,119	1,741	
L185H	Likely pathogenic		24,60	0,846	0,622	0,948	0,383	0,822	8,005	0,000	0,720	-0,350	2,509	-0,043	2,630	
L185I	Likely pathogenic		23,90	0,668	0,573	0,457	0,138	0,566	1,632	0,000	0,070	-0,350	0,183	-1,276	0,829	
L185P	Likely pathogenic		24,90	0,902	0,647	0,984	0,424	0,877	6,203	0,000	1,070	-0,350	2,868	0,498	2,853	
L185R	Likely pathogenic		24,80	0,891	0,625	0,937	0,420	0,850	5,438	0,000	1,750	-0,350	2,709	1,295	2,543	
L185V	Likely pathogenic		23,80	0,706	0,542	0,482	0,183	0,587	2,474	0,000	0,110	-0,350	0,380	-1,191	0,948	
K186E	Likely pathogenic		25,50	0,935	0,662	0,984	0,253	0,923	5,989	0,000	1,300	-0,370	2,680	0,730	2,689	
K186M	Likely pathogenic		25,80	0,910	0,610	0,962	0,306	0,870	1,452	0,000	2,890	-0,370	2,484	2,691	2,104	
K186N	Likely pathogenic		24,30	0,929	0,685	0,995	0,314	0,916	4,063	0,000	1,730	-0,370	2,740	1,279	2,641	
K186Q	Likely pathogenic		25,10	0,940	0,631	0,912	0,227	0,907	3,403	0,000	1,700	-0,370	2,347	1,179	2,288	
K186R	Likely pathogenic		23,80	0,897	0,609	0,337	0,141	0,707	2,422	0,000	1,140	-0,370	0,897	0,154	1,162	

K186T	Likely pathogenic		25,30	0,898	0,668	0,965	0,318	0,891	2,600	0,000	2,120	-0,370	2,619	1,759	2,439	
T187A	Likely pathogenic		24,70	0,851	0,611	0,396	0,129	0,832	0,084	0,483	0,220	-0,340	0,270	0,108	0,556	
T187I	Likely pathogenic		26,40	0,820	0,710	0,824	0,234	0,895	0,075	0,483	0,700	-0,340	1,420	0,970	1,513	
T187N	Likely pathogenic		25,70	0,729	0,676	0,240	0,174	0,885	0,574	0,483	0,390	-0,340	0,311	0,304	0,585	
T187P	Likely pathogenic		25,60	0,884	0,603	0,779	0,373	0,869	3,384	0,483	0,300	-0,340	1,583	0,475	1,616	
T187S	Likely pathogenic		23,00	0,787	0,620	0,177	0,075	0,582	-0,018	0,483	0,050	-0,340	-0,708	-0,352	-0,199	
N188D	Likely pathogenic		22,50	0,665	0,587	0,325	0,123	0,690	-0,050	0,292	0,140	-0,370	-0,344	-0,630	0,176	
N188H	Likely pathogenic		23,80	0,808	0,551	0,510	0,140	0,877	0,162	0,292	0,440	-0,370	0,490	-0,045	0,763	
N188I	Likely pathogenic		24,50	0,697	0,551	0,865	0,253	0,933	-0,149	0,292	1,090	-0,370	1,111	0,901	1,149	
N188K	Pathogenic	Severe	20,50	0,587	0,610	0,893	0,127	0,902	-0,195	0,292	1,730	-0,370	0,485	1,469	0,556	Yes
N188S	Pathogenic	Severe	20,50	0,494	0,488	0,077	0,073	0,584	0,176	0,292	0,340	-0,370	-1,573	-0,703	-0,977	Yes
N188T	Likely pathogenic		22,60	0,601	0,524	0,273	0,134	0,681	0,494	0,292	0,390	-0,370	-0,615	-0,395	-0,161	
N188Y	Likely pathogenic		24,20	0,818	0,582	0,812	0,244	0,898	-0,215	0,292	0,820	-0,370	1,235	0,602	1,331	
G189A	Likely pathogenic		22,70	0,797	0,489	0,209	0,114	0,678	2,357	0,471	0,290	-0,270	-0,634	-0,054	-0,316	
G189E	Likely pathogenic		22,10	0,814	0,557	0,215	0,150	0,448	2,041	0,471	0,750	-0,270	-0,710	0,473	-0,413	
G189R	Likely pathogenic		24,20	0,826	0,585	0,192	0,183	0,780	2,114	0,471	0,910	-0,270	0,088	0,868	0,197	
G189V	Likely pathogenic	Mild	23,10	0,847	0,496	0,492	0,274	0,909	3,322	0,471	0,730	-0,270	0,619	0,763	0,602	
A190E	Likely pathogenic	Severe	12,49	0,620	0,469	0,154	0,044	0,267	-0,742	0,248	1,040	-0,330	-2,331	-0,177	-1,753	
A190G	Likely pathogenic		14,48	0,662	0,406	0,183	-0,012	0,424	0,469	0,248	0,290	-0,330	-2,136	-1,022	-1,466	
A190P	Likely pathogenic		15,29	0,673	0,465	0,727	0,189	0,709	4,772	0,248	0,520	-0,330	-0,217	-0,359	0,122	
A190S	Likely pathogenic		7,05	0,615	0,389	0,091	-0,065	0,212	-0,083	0,248	0,270	-0,330	-3,358	-1,407	-2,494	
A190T	Likely pathogenic	Severe	9,53	0,648	0,416	0,091	-0,040	0,765	0,097	0,248	0,220	-0,330	-2,169	-1,171	-1,531	
A190V	Likely pathogenic		14,47	0,640	0,436	0,133	-0,041	0,580	-0,020	0,248	0,440	-0,330	-2,008	-0,811	-1,385	
V191A	Likely pathogenic		22,00	0,592	0,600	0,182	0,009	0,385	0,641	0,126	0,440	-0,370	-1,097	-0,861	-0,361	
V191E	Likely pathogenic		22,20	0,636	0,581	0,369	0,131	0,787	-1,023	0,126	1,480	-0,370	0,062	0,703	0,293	
V191G	Likely pathogenic		22,10	0,624	0,502	0,161	0,129	0,759	1,952	0,126	0,730	-0,370	-0,363	-0,350	0,030	
V191L	Likely pathogenic		15,43	0,398	0,573	0,205	-0,016	0,279	-0,635	0,126	0,110	-0,370	-2,262	-1,587	-1,270	
V191M	Likely pathogenic		15,79	0,462	0,607	0,158	-0,009	0,268	-1,152	0,126	0,110	-0,370	-2,107	-1,541	-1,123	
N192D	VUS		19,56	0,545	0,607	0,163	-0,047	0,339	1,289	0,282	0,140	-0,410	-1,764	-1,078	-0,921	
N192H	VUS		22,50	0,467	0,570	0,096	-0,050	0,409	0,630	0,282	0,440	-0,410	-1,777	-0,675	-1,032	
N192I	VUS		22,60	0,562	0,575	0,214	-0,022	0,333	1,181	0,282	1,090	-0,410	-1,419	0,191	-0,869	
N192K	VUS		16,60	0,464	0,629	0,292	-0,067	0,271	0,774	0,282	1,730	-0,410	-1,984	0,759	-1,434	
N192S	VUS		21,00	0,460	0,514	0,073	-0,097	0,342	1,402	0,282	0,340	-0,410	-2,241	-0,927	-1,430	
N192T	VUS		21,80	0,498	0,561	0,113	-0,057	0,228	1,766	0,282	0,390	-0,410	-2,012	-0,814	-1,202	
N192Y	Likely pathogenic		21,30	0,676	0,599	0,132	-0,023	0,422	-0,169	0,282	0,820	-0,410	-1,273	-0,092	-0,699	
G193A	Likely pathogenic		24,70	0,869	0,641	0,784	0,272	0,829	2,716	0,115	0,290	-0,170	1,728	-0,268	2,001	
G193E	Likely pathogenic		25,20	0,941	0,698	0,918	0,332	0,918	2,950	0,115	0,750	-0,170	2,502	0,466	2,576	
G193R	Likely pathogenic	Mild	25,00	0,940	0,713	0,808	0,417	0,920	2,715	0,115	0,910	-0,170	2,609	0,680	2,608	
G193V	Likely pathogenic		25,10	0,922	0,651	0,938	0,427	0,927	4,654	0,115	0,730	-0,170	2,678	0,458	2,672	
G193W	Likely pathogenic	Severe	25,70	0,945	0,695	0,924	0,350	0,929	3,176	0,115	0,760	-0,170	2,612	0,507	2,661	
K194E	Likely pathogenic		23,10	0,673	0,556	0,357	0,029	0,573	0,814	0,458	1,300	0,740	-1,050	1,687	-0,884	
K194M	Likely pathogenic		22,90	0,649	0,502	0,426	0,005	0,608	0,683	0,458	2,890	0,740	-1,054	3,611	-1,289	
K194N	VUS		20,90	0,603	0,574	0,766	0,014	0,516	0,602	0,458	1,730	0,740	-0,944	2,215	-0,827	
K194Q	Likely pathogenic		22,90	0,682	0,512	0,209	-0,004	0,526	0,372	0,458	1,700	0,740	-1,456	2,083	-1,357	
K194R	VUS		21,40	0,504	0,492	0,082	-0,086	0,313	0,241	0,458	1,140	0,740	-2,645	1,108	-2,201	
K194T	Likely pathogenic		22,80	0,647	0,564	0,422	0,021	0,519	1,617	0,458	2,120	0,740	-1,036	2,661	-1,037	
G195A	Likely pathogenic		25,70	0,853	0,603	0,654	0,241	0,617	0,645	0,019	0,290	0,520	1,049	-0,172	1,408	
G195E	Likely pathogenic	Severe	26,60	0,891	0,671	0,965	0,317	0,938	8,559	0,019	0,750	0,520	2,710	0,638	2,735	Yes
G195R	Likely pathogenic		25,90	0,957	0,681	0,982	0,401	0,936	14,711	0,019	0,910	0,520	3,294	0,851	3,177	
G195V	Likely pathogenic		26,30	0,894	0,605	0,927	0,412	0,923	3,840	0,019	0,730	0,520	2,506	0,655	2,484	
G195W	Likely pathogenic	Severe	26,90	0,961	0,660	0,988	0,422	0,941	48,266	0,019	0,760	0,520	4,755	0,426	4,454	
S196A	Likely pathogenic		16,06	0,481	0,394	0,122	-0,089	0,223	0,525	0,200	0,270	0,540	-3,056	-0,821	-2,249	
S196L	Likely pathogenic		22,20	0,681	0,414	0,135	-0,002	0,434	0,444	0,200	0,820	0,540	-1,621	0,254	-1,196	
S196P	Likely pathogenic	Severe	17,32	0,618	0,412	0,585	0,064	0,328	0,089	0,200	0,250	0,540	-1,650	-0,478	-1,055	Yes

S196T	Likely pathogenic		14,04	0,449	0,409	0,090	-0,117	0,186	0,514	0,200	0,050	0,540	-3,399	-1,194	-2,470	
L197F	Pathogenic	Severe	23,70	0,925	0,547	0,879	0,225	0,760	9,622	0,010	0,030	0,500	1,703	-0,532	1,985	
L197H	Likely pathogenic		27,70	0,916	0,622	0,992	0,406	0,894	10,258	0,010	0,720	0,500	2,983	0,622	2,930	
L197I	Likely pathogenic		22,30	0,698	0,560	0,348	0,147	0,458	0,315	0,010	0,070	0,500	-0,398	-0,851	0,226	
L197P	Likely pathogenic		28,30	0,931	0,658	0,957	0,380	0,889	-0,212	0,010	1,070	0,500	2,624	1,149	2,570	
L197R	Likely pathogenic		28,20	0,941	0,619	0,970	0,444	0,885	7,187	0,010	1,750	0,500	3,061	1,943	2,741	
L197V	Likely pathogenic		22,20	0,817	0,504	0,440	0,193	0,697	0,922	0,010	0,110	0,500	0,283	-0,641	0,702	
K198E	Likely pathogenic	Severe	24,90	0,843	0,676	0,511	0,132	0,876	0,409	0,263	1,300	1,150	0,720	1,916	0,721	
K198M	Likely pathogenic		25,60	0,872	0,623	0,401	0,122	0,825	-0,038	0,263	2,890	1,150	0,577	3,821	0,186	
K198N	Likely pathogenic		23,30	0,699	0,696	0,887	0,133	0,630	0,672	0,263	1,730	1,150	0,476	2,357	0,510	
K198Q	Likely pathogenic		22,40	0,742	0,646	0,253	0,108	0,652	0,831	0,263	1,700	1,150	-0,349	2,105	-0,288	
K198R	Likely pathogenic		22,20	0,727	0,623	0,101	0,027	0,434	-0,670	0,263	1,140	1,150	-1,236	1,248	-0,893	
K198T	Likely pathogenic		25,10	0,897	0,682	0,588	0,210	0,781	1,807	0,263	2,120	1,150	1,101	2,975	0,855	
G199A	Likely pathogenic		23,70	0,910	0,644	0,734	0,170	0,896	4,317	0,567	0,290	0,880	0,779	1,194	0,830	
G199E	Likely pathogenic		24,00	0,918	0,705	0,865	0,214	0,920	6,951	0,567	0,750	0,880	1,389	1,842	1,289	
G199R	Likely pathogenic		24,80	0,917	0,718	0,923	0,239	0,926	6,258	0,567	0,910	0,880	1,589	2,103	1,433	
G199V	Likely pathogenic		23,90	0,966	0,653	0,917	0,262	0,941	9,127	0,567	0,730	0,880	1,641	1,848	1,453	
Q200E	VUS		11,93	0,582	0,287	0,081	-0,063	0,222	0,296	0,591	0,400	1,840	-4,187	0,784	-3,720	
Q200H	VUS		10,41	0,501	0,298	0,121	-0,051	0,245	0,476	0,591	0,470	1,840	-4,303	0,816	-3,825	
Q200K	VUS		12,62	0,506	0,303	0,067	-0,033	0,246	-0,097	0,591	1,700	1,840	-4,072	2,386	-3,914	
Q200L	VUS		12,16	0,534	0,283	0,110	-0,025	0,427	0,201	0,591	1,190	1,840	-3,790	1,831	-3,600	
Q200P	VUS		14,64	0,602	0,290	0,141	0,124	0,585	2,174	0,591	0,120	1,840	-2,839	0,758	-2,594	
Q200R	VUS		10,16	0,394	0,299	0,071	-0,023	0,236	-0,184	0,591	0,560	1,840	-4,544	0,870	-4,050	
P201A	VUS		12,52	0,597	0,403	0,111	0,056	0,361	2,798	0,094	0,520	0,840	-2,325	-0,490	-1,725	
P201H	Likely pathogenic	Severe	23,60	0,902	0,446	0,821	0,196	0,845	8,460	0,094	0,350	0,840	1,200	0,179	1,310	
P201L	Likely pathogenic		23,70	0,836	0,457	0,588	0,123	0,721	7,344	0,094	1,070	0,840	0,498	0,894	0,575	
P201R	Likely pathogenic		23,60	0,897	0,456	0,737	0,160	0,814	5,641	0,094	0,680	0,840	0,896	0,553	0,987	
P201S	Likely pathogenic		21,30	0,721	0,402	0,417	0,081	0,625	4,923	0,094	0,250	0,840	-0,613	-0,340	-0,210	
P201T	Likely pathogenic		21,40	0,829	0,426	0,455	0,097	0,705	3,463	0,094	0,300	0,840	-0,203	-0,154	0,123	
G202A	Likely pathogenic		22,70	0,843	0,579	0,485	0,211	0,803	3,494	0,433	0,290	0,880	0,261	0,758	0,400	
G202E	Likely pathogenic		23,10	0,912	0,643	0,521	0,344	0,913	3,844	0,433	0,750	0,880	1,143	1,509	1,041	
G202R	Likely pathogenic	Severe	18,69	0,798	0,653	0,486	0,274	0,801	3,087	0,433	0,910	0,880	0,246	1,449	0,279	Yes
G202V	Likely pathogenic		23,10	0,903	0,588	0,887	0,371	0,914	5,248	0,433	0,730	0,880	1,524	1,568	1,356	
D203A	Likely pathogenic		22,50	0,775	0,429	0,521	0,196	0,754	5,018	0,197	0,750	0,740	0,141	0,631	0,247	
D203E	VUS		14,75	0,535	0,446	0,598	0,056	0,432	3,857	0,197	0,290	0,740	-1,636	-0,415	-1,052	
D203G	VUS		16,76	0,513	0,439	0,197	0,102	0,423	3,520	0,197	0,460	0,740	-1,898	-0,255	-1,361	
D203H	Likely pathogenic		21,90	0,773	0,480	0,608	0,137	0,801	5,620	0,197	0,580	0,740	0,253	0,429	0,437	
D203N	VUS		15,88	0,508	0,463	0,159	0,076	0,428	3,160	0,197	0,140	0,740	-2,048	-0,685	-1,395	
D203V	Likely pathogenic		22,70	0,873	0,426	0,826	0,243	0,815	6,355	0,197	1,190	0,740	0,962	1,345	0,839	
D203Y	Likely pathogenic		22,10	0,818	0,470	0,634	0,241	0,850	4,793	0,197	0,960	0,740	0,673	1,006	0,662	
I204F	VUS		19,81	0,406	0,324	0,128	0,065	0,267	0,493	0,467	0,100	0,580	-3,047	-0,319	-2,445	
I204L	VUS		18,91	0,346	0,316	0,080	-0,024	0,229	-0,136	0,467	0,070	0,580	-3,595	-0,485	-2,883	
I204M	VUS		15,98	0,397	0,308	0,088	-0,008	0,337	-0,154	0,467	0,070	0,580	-3,514	-0,496	-2,852	
I204N	VUS		15,11	0,384	0,354	0,162	0,137	0,230	1,012	0,467	1,090	0,580	-3,100	0,794	-2,716	
I204S	VUS		14,88	0,379	0,327	0,149	0,131	0,240	1,921	0,467	0,750	0,580	-3,190	0,347	-2,736	
I204T	VUS		13,74	0,323	0,365	0,127	0,053	0,204	1,340	0,467	0,700	0,580	-3,581	0,185	-3,000	
I204V	VUS		17,50	0,348	0,292	0,072	-0,050	0,207	0,676	0,467	0,040	0,580	-3,819	-0,600	-3,079	
Y205C	Likely pathogenic	Severe	26,80	0,752	0,557	0,177	0,224	0,809	1,885	0,236	0,250	0,900	0,219	0,311	0,454	
Y205D	Likely pathogenic		24,10	0,751	0,543	0,703	0,340	0,873	3,562	0,236	0,960	0,900	1,073	1,323	1,011	
Y205F	Likely pathogenic		22,30	0,597	0,495	0,113	0,072	0,455	-0,710	0,236	0,370	0,900	-1,599	0,030	-1,082	
Y205H	Likely pathogenic		22,70	0,653	0,560	0,232	0,154	0,488	0,557	0,236	0,380	0,900	-0,881	0,187	-0,441	
Y205N	Likely pathogenic		24,10	0,730	0,554	0,449	0,252	0,869	1,342	0,236	0,820	0,900	0,459	1,040	0,529	
Y205S	Likely pathogenic		26,10	0,698	0,509	0,368	0,331	0,838	2,789	0,236	0,480	0,900	0,520	0,644	0,611	
H206D	Likely pathogenic		27,00	0,937	0,641	0,969	0,221	0,903	5,376	0,000	0,580	0,260	2,411	0,225	2,559	

H206L	Likely pathogenic		26,10	0,876	0,618	0,794	0,223	0,838	1,934	0,000	0,720	0,260	1,761	0,288	1,949	
H206N	Likely pathogenic		26,50	0,893	0,643	0,799	0,112	0,858	1,891	0,000	0,440	0,260	1,630	-0,077	1,949	
H206P	Likely pathogenic		25,70	0,931	0,599	0,851	0,269	0,933	1,137	0,000	0,350	0,260	2,048	-0,075	2,237	
H206Q	Likely pathogenic		23,50	0,804	0,626	0,871	0,142	0,854	2,385	0,000	0,470	0,260	1,374	-0,143	1,712	
H206R	Likely pathogenic		24,60	0,853	0,646	0,689	0,210	0,805	2,485	0,000	1,030	0,260	1,528	0,572	1,698	
H206Y	Likely pathogenic		22,50	0,692	0,646	0,321	0,038	0,447	-0,708	0,000	0,380	0,260	-0,457	-0,657	0,230	
Q207E	VUS		22,80	0,502	0,325	0,092	0,044	0,366	0,315	0,253	0,400	0,370	-2,246	-0,366	-1,700	
Q207H	Likely pathogenic		24,50	0,794	0,335	0,267	0,063	0,696	-0,363	0,253	0,470	0,370	-0,838	0,094	-0,572	
Q207K	VUS		20,90	0,374	0,329	0,072	0,055	0,278	-1,024	0,253	1,700	0,370	-2,699	1,090	-2,368	
Q207L	Likely pathogenic		22,80	0,758	0,312	0,213	0,081	0,737	-0,347	0,253	1,190	0,370	-0,982	0,908	-0,896	
Q207P	Likely pathogenic		24,00	0,813	0,322	0,490	0,303	0,799	3,524	0,253	0,120	0,370	0,242	-0,143	0,361	
Q207R	VUS		21,40	0,509	0,322	0,069	0,073	0,411	-1,623	0,253	0,560	0,370	-2,292	-0,166	-1,800	
T208A	VUS		21,40	0,628	0,480	0,101	0,075	0,271	0,470	0,000	0,220	0,010	-1,383	-1,240	-0,626	
T208I	Likely pathogenic		25,90	0,815	0,585	0,517	0,121	0,602	0,401	0,000	0,700	0,010	0,637	-0,134	1,036	
T208N	Likely pathogenic		25,40	0,862	0,542	0,397	0,231	0,801	1,044	0,000	0,390	0,010	1,019	-0,432	1,326	
T208P	Likely pathogenic		24,40	0,893	0,485	0,606	0,257	0,800	4,080	0,000	0,300	0,010	1,284	-0,528	1,536	
T208S	Likely pathogenic		23,30	0,728	0,492	0,213	0,050	0,378	1,414	0,000	0,050	0,010	-0,779	-1,293	-0,067	
W209C	Likely pathogenic		34,00	0,929	0,663	0,985	0,435	0,940	3,017	0,000	0,010	-0,590	3,558	-0,609	3,731	
W209G	Likely pathogenic		27,90	0,932	0,636	0,945	0,484	0,944	4,905	0,000	0,760	-0,590	3,281	0,130	3,267	
W209L	Likely pathogenic		27,40	0,857	0,671	0,820	0,370	0,855	0,752	0,000	0,080	-0,590	2,424	-0,833	2,758	
W209R	Likely pathogenic		26,80	0,942	0,704	0,994	0,482	0,950	1,642	0,000	1,670	-0,590	3,380	1,287	3,192	
W209S	Likely pathogenic		28,50	0,921	0,655	0,970	0,497	0,929	3,466	0,000	0,740	-0,590	3,324	0,145	3,327	
A210D	Likely pathogenic		28,40	0,969	0,610	0,979	0,390	0,946	7,730	0,000	0,750	-0,060	3,151	0,381	3,110	
A210G	Likely pathogenic		28,10	0,956	0,513	0,269	0,222	0,906	1,559	0,000	0,290	-0,060	1,340	-0,496	1,579	
A210P	Likely pathogenic		26,10	0,972	0,581	0,976	0,429	0,954	7,767	0,000	0,520	-0,060	3,015	0,049	3,006	
A210S	Likely pathogenic		25,30	0,952	0,495	0,201	0,188	0,791	0,131	0,000	0,270	-0,060	0,705	-0,673	1,040	
A210T	Likely pathogenic		28,70	0,971	0,526	0,714	0,206	0,905	3,607	0,000	0,220	-0,060	1,973	-0,449	2,191	
A210V	Likely pathogenic		28,00	0,937	0,540	0,817	0,195	0,932	7,123	0,000	0,440	-0,060	2,180	-0,207	2,339	
R211G	VUS		23,80	0,503	0,232	0,120	0,180	0,396	2,532	0,318	0,910	0,130	-1,903	0,315	-1,645	
R211I	VUS		17,71	0,464	0,249	0,233	0,106	0,417	1,148	0,318	1,680	0,130	-2,386	1,085	-2,208	
R211K	VUS		14,24	0,438	0,248	0,082	-0,063	0,117	0,510	0,318	1,140	0,130	-3,766	0,082	-3,193	
R211S	VUS		21,00	0,427	0,229	0,174	0,059	0,230	1,591	0,318	0,930	0,130	-2,765	0,123	-2,326	
R211T	VUS		16,07	0,424	0,239	0,125	0,069	0,279	1,979	0,318	0,980	0,130	-3,031	0,055	-2,583	
Y212C	Likely pathogenic		28,10	0,949	0,692	0,793	0,405	0,936	3,842	0,000	0,250	-0,380	2,971	-0,417	3,150	
Y212D	Likely pathogenic		25,60	0,953	0,685	0,973	0,449	0,929	6,335	0,000	0,960	-0,380	3,233	0,434	3,203	
Y212F	Likely pathogenic		25,90	0,918	0,631	0,402	0,199	0,807	2,211	0,000	0,370	-0,380	1,435	-0,628	1,798	
Y212H	Likely pathogenic		25,30	0,928	0,697	0,924	0,257	0,921	4,390	0,000	0,380	-0,380	2,544	-0,398	2,809	
Y212N	Likely pathogenic		25,50	0,943	0,692	0,895	0,428	0,945	4,053	0,000	0,820	-0,380	3,006	0,248	3,043	
Y212S	Likely pathogenic		27,30	0,961	0,649	0,902	0,439	0,944	4,560	0,000	0,480	-0,380	3,094	-0,123	3,164	
F213C	Likely pathogenic		28,90	0,909	0,625	0,846	0,183	0,939	4,242	0,000	0,120	-0,570	2,366	-0,820	2,701	
F213I	Pathogenic	Severe	22,70	0,786	0,605	0,529	0,130	0,842	1,736	0,000	0,100	-0,570	0,923	-1,219	1,454	Yes
F213L	Likely pathogenic		21,80	0,677	0,633	0,942	0,095	0,666	1,185	0,000	0,030	-0,570	0,800	-1,328	1,470	
F213S	Likely pathogenic		28,90	0,880	0,603	0,931	0,261	0,893	4,808	0,000	0,850	-0,570	2,549	0,097	2,667	
F213V	Likely pathogenic		25,60	0,884	0,586	0,574	0,153	0,789	2,052	0,000	0,140	-0,570	1,308	-1,044	1,761	
F213Y	Likely pathogenic		22,90	0,701	0,621	0,452	0,074	0,486	0,538	0,000	0,370	-0,570	0,031	-1,084	0,712	
V214A	Likely pathogenic		22,80	0,694	0,483	0,298	0,083	0,520	0,834	0,126	0,440	-0,570	-0,568	-0,832	-0,017	
V214E	Likely pathogenic		27,10	0,886	0,464	0,515	0,298	0,866	1,944	0,126	1,480	-0,570	1,449	0,937	1,349	
V214G	Likely pathogenic		27,10	0,883	0,407	0,311	0,300	0,659	2,145	0,126	0,730	-0,570	0,730	-0,135	0,877	
V214L	VUS		22,00	0,491	0,457	0,189	0,081	0,291	-0,633	0,126	0,110	-0,570	-1,625	-1,468	-0,821	
V214M	Likely pathogenic		23,60	0,718	0,481	0,228	0,106	0,620	0,047	0,126	0,110	-0,570	-0,401	-1,169	0,171	
K215E	Likely pathogenic		23,70	0,898	0,540	0,542	0,247	0,893	1,923	0,360	1,300	0,170	0,877	1,553	0,753	
K215M	VUS		24,50	0,852	0,495	0,563	0,164	0,781	-0,285	0,360	2,890	0,170	0,426	3,419	0,009	
K215N	Likely pathogenic		22,80	0,797	0,566	0,793	0,162	0,695	1,191	0,360	1,730	0,170	0,466	1,979	0,406	
K215Q	Likely pathogenic		23,40	0,832	0,507	0,251	0,153	0,863	1,105	0,360	1,700	0,170	0,058	1,849	-0,056	

K215R	VUS		19,61	0,440	0,501	0,079	0,047	0,531	-0,098	0,360	1,140	0,170	-1,997	0,651	-1,590
K215T	VUS		24,20	0,832	0,560	0,499	0,244	0,768	2,512	0,360	2,120	0,170	0,679	2,483	0,433
F216C	Likely pathogenic		25,40	0,945	0,719	0,963	0,416	0,935	5,740	0,000	0,120	-0,590	3,156	-0,725	3,387
F216I	Likely pathogenic		25,40	0,932	0,696	0,956	0,391	0,931	4,312	0,000	0,100	-0,590	2,947	-0,770	3,203
F216L	Likely pathogenic		25,60	0,940	0,719	0,996	0,353	0,925	1,375	0,000	0,030	-0,590	2,848	-0,824	3,165
F216S	Likely pathogenic		25,40	0,949	0,698	0,982	0,426	0,946	6,201	0,000	0,850	-0,590	3,252	0,173	3,281
F216V	Likely pathogenic		25,40	0,944	0,679	0,934	0,386	0,928	5,271	0,000	0,140	-0,590	2,929	-0,740	3,164
F216Y	Pathogenic	Mild	24,30	0,864	0,711	0,514	0,271	0,898	2,038	0,000	0,370	-0,590	1,875	-0,684	2,237
L217M	Likely pathogenic		23,00	0,850	0,559	0,337	0,135	0,830	0,040	0,015	0,000	-0,370	0,600	-1,216	1,112
L217P	Likely pathogenic		25,10	0,957	0,656	0,962	0,397	0,951	8,302	0,015	1,070	-0,370	3,099	0,540	3,040
L217Q	Likely pathogenic		24,90	0,937	0,578	0,897	0,334	0,945	3,187	0,015	1,190	-0,370	2,430	0,627	2,391
L217R	Likely pathogenic		25,00	0,942	0,629	0,896	0,393	0,947	5,932	0,015	1,750	-0,370	2,865	1,348	2,657
L217V	Likely pathogenic		21,90	0,848	0,528	0,386	0,137	0,720	4,751	0,015	0,110	-0,370	0,544	-1,187	1,034
D218A	Likely pathogenic		26,20	0,861	0,459	0,334	0,114	0,701	0,428	0,280	0,750	0,000	-0,003	0,450	0,184
D218E	VUS		17,55	0,548	0,473	0,249	0,026	0,305	-0,033	0,280	0,290	0,000	-2,122	-0,707	-1,420
D218G	Likely pathogenic		26,50	0,878	0,461	0,327	0,130	0,737	1,780	0,280	0,460	0,000	0,176	0,121	0,395
D218H	Likely pathogenic		23,50	0,873	0,513	0,409	0,196	0,739	0,474	0,280	0,580	0,000	0,287	0,275	0,485
D218N	Likely pathogenic		23,20	0,734	0,494	0,162	0,073	0,615	0,299	0,280	0,140	0,000	-0,840	-0,529	-0,346
D218V	Likely pathogenic		26,20	0,905	0,447	0,525	0,234	0,771	0,695	0,280	1,190	0,000	0,701	1,148	0,644
D218Y	Likely pathogenic		23,60	0,916	0,505	0,509	0,232	0,769	0,181	0,280	0,960	0,000	0,610	0,821	0,657
A219D	Likely pathogenic		24,30	0,842	0,416	0,356	0,197	0,791	2,032	0,109	0,750	-0,260	0,438	-0,060	0,618
A219G	Likely pathogenic		23,70	0,755	0,368	0,191	0,109	0,590	1,755	0,109	0,290	-0,260	-0,623	-0,864	-0,172
A219P	Likely pathogenic		23,40	0,836	0,408	0,964	0,310	0,819	2,523	0,109	0,520	-0,260	1,330	-0,129	1,447
A219S	Likely pathogenic		22,80	0,714	0,362	0,106	0,066	0,553	0,496	0,109	0,270	-0,260	-1,084	-0,987	-0,555
A219T	Likely pathogenic		23,20	0,795	0,378	0,095	0,081	0,599	0,457	0,109	0,220	-0,260	-0,777	-0,969	-0,289
A219V	Likely pathogenic		24,20	0,812	0,383	0,145	0,080	0,603	0,220	0,109	0,440	-0,260	-0,595	-0,644	-0,176
Y220C	Likely pathogenic		32,00	0,912	0,611	0,470	0,417	0,903	5,532	0,000	0,250	-0,230	2,643	-0,385	2,777
Y220D	Likely pathogenic		26,80	0,930	0,601	0,965	0,461	0,920	7,644	0,000	0,960	-0,230	3,104	0,499	3,015
Y220F	Likely pathogenic		27,90	0,855	0,536	0,260	0,284	0,772	0,488	0,000	0,370	-0,230	1,124	-0,547	1,424
Y220H	Likely pathogenic		26,60	0,924	0,613	0,858	0,354	0,902	3,929	0,000	0,380	-0,230	2,512	-0,279	2,673
Y220N	Likely pathogenic		26,70	0,926	0,606	0,876	0,440	0,938	5,131	0,000	0,820	-0,230	2,865	0,314	2,842
Y220S	Likely pathogenic		29,90	0,920	0,554	0,765	0,383	0,934	6,254	0,000	0,480	-0,230	2,713	-0,110	2,764
A221D	Likely pathogenic		24,70	0,649	0,519	0,233	0,112	0,713	1,915	0,364	0,750	0,410	-0,604	0,699	-0,363
A221G	VUS		24,50	0,605	0,434	0,138	0,020	0,535	0,164	0,364	0,290	0,410	-1,600	-0,050	-1,128
A221P	Likely pathogenic		24,10	0,782	0,490	0,826	0,168	0,850	5,175	0,364	0,520	0,410	0,647	0,677	0,740
A221S	VUS		21,50	0,612	0,419	0,091	-0,010	0,437	0,374	0,364	0,270	0,410	-2,095	-0,225	-1,547
A221T	Likely pathogenic		22,80	0,684	0,445	0,088	0,007	0,458	1,381	0,364	0,220	0,410	-1,701	-0,197	-1,190
A221V	VUS		23,40	0,596	0,463	0,138	0,009	0,576	1,166	0,364	0,440	0,410	-1,540	0,110	-1,092
E222A	VUS		18,93	0,566	0,333	0,076	0,025	0,226	-0,350	0,704	1,040	1,620	-3,494	1,930	-3,290
E222D	VUS		12,69	0,596	0,336	0,129	-0,003	0,250	0,584	0,704	0,290	1,620	-3,860	0,855	-3,435
E222G	VUS		22,10	0,585	0,326	0,080	0,072	0,332	0,243	0,704	0,750	1,620	-2,973	1,728	-2,805
E222K	VUS		13,35	0,544	0,362	0,063	0,032	0,252	-0,744	0,704	1,300	1,620	-3,818	2,105	-3,623
E222Q	Likely pathogenic		15,07	0,669	0,323	0,070	0,006	0,377	-0,719	0,704	0,400	1,620	-3,484	1,129	-3,179
E222V	VUS		21,90	0,604	0,320	0,169	0,065	0,446	0,010	0,704	1,480	1,620	-2,669	2,685	-2,729
H223D	VUS		21,70	0,618	0,448	0,338	0,096	0,591	2,387	0,317	0,580	0,890	-1,170	0,520	-0,855
H223L	VUS		22,90	0,600	0,430	0,209	0,017	0,513	0,881	0,317	0,720	0,890	-1,663	0,614	-1,298
H223N	VUS		17,51	0,462	0,454	0,122	-0,043	0,270	1,141	0,317	0,440	0,890	-2,861	-0,081	-2,191
H223P	Likely pathogenic		24,30	0,670	0,425	0,555	0,212	0,737	4,165	0,317	0,350	0,890	-0,150	0,490	0,020
H223Q	VUS		18,53	0,593	0,435	0,146	-0,080	0,427	1,513	0,317	0,470	0,890	-2,395	0,079	-1,839
H223R	VUS		21,60	0,563	0,454	0,084	-0,007	0,473	0,352	0,317	1,030	0,890	-2,020	0,890	-1,655
H223Y	VUS		16,83	0,441	0,449	0,092	-0,080	0,256	-0,279	0,317	0,380	0,890	-3,165	-0,207	-2,431
K224E	VUS		22,00	0,520	0,391	0,086	0,105	0,304	0,392	0,775	1,300	0,940	-2,820	2,154	-2,720
K224M	VUS		22,50	0,633	0,352	0,316	0,170	0,541	0,521	0,775	2,890	0,940	-1,776	4,334	-2,271
K224N	VUS		13,78	0,416	0,410	0,109	0,070	0,184	-0,082	0,775	1,730	0,940	-3,779	2,357	-3,604

K224Q	VUS		22,60	0,518	0,360	0,083	0,086	0,310	0,050	0,775	1,700	0,940	-2,880	2,640	-2,883
K224R	VUS		20,50	0,493	0,356	0,083	0,018	0,320	-0,457	0,775	1,140	0,940	-3,299	1,852	-3,100
K224T	VUS		22,10	0,620	0,404	0,165	0,114	0,405	1,678	0,775	2,120	0,940	-2,219	3,266	-2,408
L225F	Likely pathogenic		22,70	0,669	0,375	0,533	0,135	0,747	2,537	0,025	0,030	0,110	-0,113	-1,046	0,348
L225I	VUS		12,10	0,529	0,397	0,100	0,043	0,238	1,326	0,025	0,070	0,110	-2,579	-1,693	-1,701
L225S	Likely pathogenic		23,50	0,683	0,387	0,918	0,305	0,882	4,259	0,025	0,820	0,110	1,163	0,191	1,235
L225V	VUS		12,34	0,537	0,349	0,109	0,083	0,320	2,572	0,025	0,110	0,110	-2,379	-1,610	-1,599
Q226E	VUS		11,32	0,458	0,259	0,081	-0,053	0,230	0,823	0,680	0,400	0,180	-4,264	-0,079	-3,681
Q226H	VUS		18,05	0,401	0,264	0,101	-0,048	0,326	0,117	0,680	0,470	0,180	-3,735	0,214	-3,233
Q226K	VUS		12,23	0,453	0,266	0,075	-0,028	0,189	0,333	0,680	1,700	0,180	-4,125	1,538	-3,858
Q226L	VUS		13,84	0,412	0,250	0,135	0,053	0,295	0,356	0,680	1,190	0,180	-3,747	1,030	-3,452
Q226P	VUS		12,69	0,542	0,270	0,086	0,113	0,526	1,872	0,680	0,120	0,180	-3,116	-0,152	-2,720
Q226R	VUS		11,50	0,360	0,261	0,065	-0,026	0,203	0,058	0,680	0,560	0,180	-4,447	0,081	-3,866
F227C	Likely pathogenic		28,00	0,946	0,636	0,898	0,300	0,931	4,363	0,038	0,120	-0,650	2,693	-0,716	2,943
F227I	Likely pathogenic		24,30	0,822	0,623	0,583	0,176	0,902	3,727	0,038	0,100	-0,650	1,453	-1,074	1,892
F227L	Likely pathogenic		24,20	0,826	0,638	0,962	0,151	0,915	2,350	0,038	0,030	-0,650	1,805	-1,043	2,258
F227S	Likely pathogenic		28,00	0,947	0,618	0,976	0,311	0,943	5,981	0,038	0,850	-0,650	2,904	0,189	2,943
F227V	Likely pathogenic		26,50	0,860	0,602	0,647	0,185	0,903	4,139	0,038	0,140	-0,650	1,743	-0,933	2,118
F227Y	Likely pathogenic		26,30	0,828	0,629	0,617	0,151	0,869	4,349	0,038	0,370	-0,650	1,593	-0,703	1,972
W228C	Likely pathogenic		28,20	0,951	0,673	0,944	0,505	0,917	3,924	0,144	0,010	-0,490	3,124	-0,392	3,238
W228G	Likely pathogenic		29,90	0,938	0,641	0,696	0,555	0,930	3,747	0,144	0,760	-0,490	3,055	0,514	2,948
W228L	Likely pathogenic		26,90	0,901	0,677	0,782	0,517	0,883	2,761	0,144	0,080	-0,490	2,703	-0,408	2,855
W228R	Likely pathogenic		28,50	0,921	0,710	0,953	0,485	0,942	3,150	0,144	1,670	-0,490	3,267	1,652	3,009
W228S	Likely pathogenic		27,70	0,923	0,662	0,677	0,568	0,920	3,296	0,144	0,740	-0,490	2,899	0,432	2,828
A229E	Likely pathogenic		29,70	0,968	0,577	0,952	0,303	0,920	5,966	0,000	1,040	-0,870	2,998	0,240	2,996
A229G	VUS		23,30	0,638	0,507	0,151	0,123	0,493	1,627	0,000	0,290	-0,870	-0,434	-1,487	0,241
A229P	Likely pathogenic		25,70	0,951	0,577	0,978	0,357	0,943	5,482	0,000	0,520	-0,870	2,816	-0,467	2,938
A229S	Likely pathogenic		24,80	0,813	0,488	0,288	0,116	0,690	0,853	0,000	0,270	-0,870	0,351	-1,282	0,871
A229T	Likely pathogenic		25,60	0,917	0,522	0,617	0,133	0,867	3,814	0,000	0,220	-0,870	1,477	-1,115	1,862
A229V	Likely pathogenic		29,90	0,923	0,536	0,680	0,203	0,916	1,488	0,000	0,440	-0,870	2,053	-0,625	2,302
V230A	Likely pathogenic		27,70	0,807	0,414	0,619	0,181	0,851	3,431	0,000	0,440	-0,860	1,249	-0,855	1,537
V230E	Likely pathogenic		28,70	0,898	0,397	0,959	0,305	0,930	4,636	0,000	1,480	-0,860	2,369	0,666	2,219
V230G	Likely pathogenic		28,50	0,926	0,345	0,817	0,306	0,888	4,900	0,000	0,730	-0,860	2,024	-0,320	2,047
V230L	VUS		16,71	0,593	0,393	0,205	0,077	0,286	-0,750	0,000	0,110	-0,860	-1,730	-2,015	-0,878
V230M	Likely pathogenic		22,80	0,668	0,413	0,233	0,097	0,594	-0,440	0,000	0,110	-0,860	-0,558	-1,678	0,097
T231A	Likely pathogenic		27,90	0,900	0,521	0,702	0,208	0,895	0,012	0,000	0,220	-0,680	1,721	-0,844	2,039
T231I	Likely pathogenic		28,90	0,951	0,632	0,977	0,301	0,939	0,842	0,000	0,700	-0,680	2,817	-0,019	2,941
T231K	Likely pathogenic		29,60	0,946	0,602	0,979	0,358	0,940	7,232	0,000	2,120	-0,680	3,295	1,701	2,987
T231P	Likely pathogenic		29,00	0,944	0,524	0,893	0,440	0,864	0,211	0,000	0,300	-0,680	2,626	-0,521	2,754
T231R	Likely pathogenic	Severe	29,20	0,961	0,589	0,972	0,361	0,938	15,380	0,000	0,980	-0,680	3,509	0,230	3,423
T231S	Likely pathogenic		26,60	0,839	0,530	0,453	0,142	0,611	1,189	0,000	0,050	-0,680	0,717	-1,328	1,270
A232D	Likely pathogenic		22,90	0,666	0,382	0,951	0,211	0,813	4,288	0,000	0,750	-0,640	0,963	-0,473	1,205
A232G	VUS	Mild	19,73	0,548	0,314	0,353	0,041	0,508	1,590	0,000	0,290	-0,640	-1,315	-1,558	-0,646
A232P	VUS		20,10	0,624	0,353	0,363	0,158	0,703	3,605	0,000	0,520	-0,640	-0,368	-1,095	0,074
A232S	VUS		15,94	0,513	0,302	0,188	0,006	0,294	1,619	0,000	0,270	-0,640	-2,263	-1,850	-1,439
A232T	VUS		16,25	0,464	0,309	0,135	-0,005	0,269	0,567	0,000	0,220	-0,640	-2,487	-1,945	-1,606
A232V	VUS		18,78	0,561	0,341	0,127	-0,001	0,213	-1,264	0,000	0,440	-0,640	-2,187	-1,549	-1,375
E233A	Likely pathogenic		25,80	0,775	0,500	0,236	0,153	0,764	-2,213	0,000	1,040	-0,720	0,423	-0,184	0,728
E233D	Pathogenic		23,10	0,748	0,500	0,925	0,118	0,586	0,813	0,000	0,290	-0,720	0,665	-1,095	1,205
E233G	Likely pathogenic		22,80	0,571	0,460	0,136	0,073	0,437	-0,871	0,000	0,750	-0,720	-1,028	-0,935	-0,400
E233K	Likely pathogenic		23,90	0,725	0,576	0,825	0,175	0,748	-0,699	0,000	1,300	-0,720	1,136	0,252	1,387
E233Q	Likely pathogenic		17,76	0,604	0,485	0,261	0,019	0,467	-1,250	0,000	0,400	-0,720	-1,250	-1,457	-0,483
E233V	Likely pathogenic		26,60	0,800	0,481	0,568	0,166	0,797	-1,505	0,000	1,480	-0,720	0,991	0,490	1,117
N234D	Likely pathogenic		25,40	0,918	0,603	0,989	0,330	0,949	3,879	0,026	0,140	-0,550	2,558	-0,704	2,788

N234H	Likely pathogenic		25,00	0,920	0,575	0,995	0,332	0,941	1,350	0,026	0,440	-0,550	2,386	-0,338	2,549
N234I	Likely pathogenic		27,10	0,918	0,568	0,995	0,377	0,948	8,300	0,026	1,090	-0,550	2,966	0,481	2,882
N234K	Likely pathogenic		23,40	0,817	0,620	0,998	0,330	0,935	1,636	0,026	1,730	-0,550	2,281	1,157	2,203
N234S	Likely pathogenic		25,50	0,917	0,527	0,973	0,297	0,932	1,292	0,026	0,340	-0,550	2,168	-0,496	2,359
N234T	Likely pathogenic		26,30	0,921	0,557	0,992	0,347	0,941	0,651	0,026	0,390	-0,550	2,437	-0,356	2,588
N234Y	Likely pathogenic		25,60	0,929	0,594	0,995	0,300	0,932	3,385	0,026	0,820	-0,550	2,511	0,118	2,593
E235A	Likely pathogenic		27,40	0,943	0,610	0,988	0,319	0,934	-1,015	0,081	1,040	-0,210	2,436	0,805	2,419
E235D	Likely pathogenic		23,60	0,838	0,605	0,996	0,285	0,895	1,917	0,081	0,290	-0,210	1,891	-0,326	2,143
E235G	Likely pathogenic		28,70	0,954	0,575	0,985	0,345	0,944	-0,368	0,081	0,750	-0,210	2,542	0,489	2,543
E235K	Likely pathogenic		26,60	0,976	0,677	0,965	0,341	0,953	-1,362	0,081	1,300	-0,210	2,661	1,159	2,586
E235Q	Likely pathogenic		25,40	0,913	0,595	0,952	0,224	0,935	-1,354	0,081	0,400	-0,210	1,872	-0,114	2,094
E235V	Likely pathogenic		28,10	0,951	0,589	0,996	0,332	0,951	0,227	0,081	1,480	-0,210	2,598	1,363	2,434
P236A	Likely pathogenic		25,90	0,958	0,563	0,856	0,326	0,923	2,888	0,000	0,520	-0,370	2,365	-0,212	2,489
P236H	Likely pathogenic		26,90	0,982	0,613	0,994	0,365	0,936	61,727	0,000	0,350	-0,370	5,268	-0,782	5,072
P236L	Likely pathogenic		27,30	0,965	0,625	0,957	0,307	0,949	3,217	0,000	1,070	-0,370	2,783	0,553	2,780
P236R	Likely pathogenic		26,90	0,970	0,623	0,987	0,396	0,951	16,409	0,000	0,680	-0,370	3,521	0,015	3,482
P236S	Likely pathogenic		26,80	0,962	0,575	0,974	0,326	0,941	4,334	0,000	0,250	-0,370	2,659	-0,482	2,823
P236T	Likely pathogenic		26,30	0,967	0,601	0,962	0,338	0,952	5,260	0,000	0,300	-0,370	2,772	-0,422	2,921
S237A	VUS		20,30	0,510	0,423	0,155	-0,028	0,378	-0,381	0,142	0,270	-0,400	-1,991	-1,236	-1,216
S237C	Likely pathogenic		25,50	0,737	0,437	0,361	0,104	0,740	0,185	0,142	0,730	-0,400	-0,018	-0,159	0,292
S237F	Likely pathogenic		23,60	0,724	0,460	0,630	0,044	0,811	-1,112	0,142	0,850	-0,400	0,094	0,017	0,404
S237P	Likely pathogenic	Severe	23,40	0,749	0,434	0,819	0,207	0,807	4,066	0,142	0,250	-0,400	0,826	-0,620	1,121
S237T	VUS		16,17	0,456	0,432	0,072	-0,062	0,240	0,199	0,142	0,050	-0,400	-2,733	-1,737	-1,774
S237Y	Likely pathogenic		25,00	0,763	0,480	0,663	0,136	0,822	-1,224	0,142	0,480	-0,400	0,554	-0,304	0,874
A238D	Likely pathogenic		22,80	0,708	0,584	0,852	0,109	0,574	7,240	0,016	0,750	-0,330	0,842	-0,354	1,270
A238G	Likely pathogenic		25,50	0,767	0,499	0,449	0,048	0,596	1,224	0,016	0,290	-0,330	0,102	-0,939	0,652
A238P	Likely pathogenic		22,90	0,858	0,555	0,957	0,181	0,893	5,074	0,016	0,520	-0,330	1,724	-0,372	1,980
A238S	Likely pathogenic		20,20	0,666	0,481	0,194	-0,004	0,376	0,824	0,016	0,270	-0,330	-1,256	-1,342	-0,496
A238T	Likely pathogenic		20,90	0,695	0,510	0,285	0,000	0,614	1,513	0,016	0,220	-0,330	-0,593	-1,253	0,075
A238V	Likely pathogenic		25,30	0,801	0,528	0,525	0,017	0,814	2,668	0,016	0,440	-0,330	0,627	-0,665	1,068
G239A	Likely pathogenic		23,30	0,842	0,624	0,621	0,203	0,814	4,633	0,010	0,290	-0,130	1,383	-0,627	1,757
G239E	Likely pathogenic		25,10	0,939	0,684	0,980	0,358	0,945	23,190	0,010	0,750	-0,130	3,595	0,137	3,559
G239R	Likely pathogenic		24,40	0,941	0,691	0,971	0,297	0,945	37,054	0,010	0,910	-0,130	3,992	0,167	3,886
G239V	Likely pathogenic		25,00	0,880	0,630	0,941	0,307	0,943	22,645	0,010	0,730	-0,130	3,158	0,024	3,165
G239W	Likely pathogenic		25,00	0,932	0,678	0,979	0,396	0,932	58,641	0,010	0,760	-0,130	5,090	-0,136	4,836
L240M	VUS		16,60	0,473	0,430	0,146	-0,070	0,207	0,373	0,289	0,000	-0,040	-2,933	-1,267	-2,043
L240P	VUS	Severe	22,60	0,614	0,525	0,821	0,137	0,777	2,685	0,289	1,070	-0,040	0,252	0,801	0,425
L240Q	Likely pathogenic		22,30	0,706	0,449	0,160	0,053	0,376	0,831	0,289	1,190	-0,040	-1,374	0,595	-1,041
L240R	VUS		20,60	0,610	0,499	0,210	0,114	0,492	0,885	0,289	1,750	-0,040	-1,140	1,292	-0,955
L240V	VUS		15,79	0,541	0,411	0,156	-0,057	0,346	1,808	0,289	0,110	-0,040	-2,589	-1,084	-1,822
L241F	VUS		12,61	0,297	0,331	0,096	0,001	0,202	-0,162	0,507	0,030	0,010	-4,005	-0,965	-3,189
L241M	VUS		9,58	0,360	0,319	0,147	-0,038	0,144	0,307	0,507	0,000	0,010	-4,231	-1,090	-3,375
L241S	VUS		22,20	0,388	0,347	0,592	0,126	0,457	1,567	0,507	0,820	0,010	-1,761	0,604	-1,454
L241V	VUS		9,04	0,418	0,307	0,110	-0,025	0,234	0,846	0,507	0,110	0,010	-4,032	-0,924	-3,267
L241W	VUS		22,30	0,442	0,355	0,243	0,128	0,535	0,690	0,507	0,080	0,010	-1,988	-0,333	-1,520
S242C	VUS		18,88	0,662	0,421	0,176	0,031	0,415	-0,084	0,677	0,730	-0,050	-2,343	0,707	-2,011
S242G	VUS		13,85	0,569	0,388	0,091	-0,095	0,240	0,293	0,677	0,020	-0,050	-3,646	-0,518	-2,936
S242I	VUS		16,68	0,604	0,438	0,198	-0,029	0,232	-0,272	0,677	0,750	-0,050	-2,965	0,561	-2,488
S242N	VUS		14,34	0,491	0,440	0,131	-0,092	0,205	-0,068	0,677	0,340	-0,050	-3,631	-0,127	-2,939
S242R	VUS		13,93	0,590	0,446	0,327	-0,006	0,245	-0,405	0,677	0,930	-0,050	-2,942	0,756	-2,506
S242T	VUS		13,08	0,576	0,410	0,073	-0,131	0,197	-0,103	0,677	0,050	-0,050	-3,818	-0,526	-3,060
G243A	Likely pathogenic		13,58	0,671	0,412	0,160	0,007	0,319	2,621	0,654	0,290	-0,360	-2,766	-0,278	-2,226
G243E	Likely pathogenic		17,33	0,726	0,457	0,281	0,066	0,495	2,502	0,654	0,750	-0,360	-1,723	0,563	-1,434
G243R	Likely pathogenic		21,30	0,763	0,477	0,252	0,085	0,510	2,310	0,654	0,910	-0,360	-1,286	0,904	-1,087

G243V	Likely pathogenic		15,93	0,748	0,409	0,297	0,092	0,426	4,830	0,654	0,730	-0,360	-1,823	0,466	-1,553
Y244C	Likely pathogenic		29,40	0,880	0,548	0,815	0,124	0,856	2,877	0,129	0,250	-0,780	1,683	-0,608	2,003
Y244D	Likely pathogenic		24,60	0,814	0,542	0,906	0,160	0,750	4,029	0,129	0,960	-0,780	1,334	0,092	1,545
Y244F	Likely pathogenic		22,80	0,659	0,476	0,209	-0,034	0,481	-0,443	0,129	0,370	-0,780	-1,109	-1,146	-0,417
Y244H	Likely pathogenic		25,10	0,892	0,553	0,887	0,064	0,802	1,904	0,129	0,380	-0,780	1,242	-0,583	1,618
Y244N	Likely pathogenic		23,40	0,784	0,552	0,780	0,137	0,810	2,836	0,129	0,820	-0,780	1,051	-0,143	1,329
Y244S	Likely pathogenic		23,70	0,769	0,491	0,753	0,147	0,553	3,215	0,129	0,480	-0,780	0,503	-0,675	0,927
P245A	Likely pathogenic		23,10	0,751	0,410	0,155	-0,014	0,562	1,466	0,434	0,520	-0,580	-1,327	-0,190	-0,918
P245H	Likely pathogenic		29,20	0,704	0,444	0,314	0,031	0,700	1,848	0,434	0,350	-0,580	-0,413	-0,123	-0,070
P245L	Likely pathogenic		29,60	0,845	0,463	0,348	0,048	0,549	1,121	0,434	1,070	-0,580	-0,197	0,819	-0,031
P245R	Likely pathogenic		25,40	0,753	0,464	0,254	0,049	0,690	0,783	0,434	0,680	-0,580	-0,598	0,205	-0,311
P245S	Likely pathogenic		22,20	0,659	0,397	0,174	-0,030	0,306	1,547	0,434	0,250	-0,580	-2,014	-0,688	-1,400
P245T	Likely pathogenic		23,40	0,758	0,420	0,208	-0,002	0,770	1,863	0,434	0,300	-0,580	-0,870	-0,353	-0,498
F246C	Likely pathogenic		29,20	0,915	0,600	0,982	0,293	0,929	2,231	0,075	0,120	-0,150	2,470	-0,309	2,667
F246I	Likely pathogenic		24,20	0,775	0,571	0,962	0,177	0,810	2,802	0,075	0,100	-0,150	1,327	-0,669	1,723
F246L	Likely pathogenic		23,60	0,776	0,605	0,996	0,143	0,843	3,733	0,075	0,030	-0,150	1,405	-0,762	1,839
F246S	Likely pathogenic		29,10	0,931	0,567	0,972	0,236	0,943	2,995	0,075	0,850	-0,150	2,375	0,539	2,401
F246V	Likely pathogenic		26,90	0,889	0,554	0,958	0,184	0,861	2,675	0,075	0,140	-0,150	1,779	-0,474	2,070
F246Y	Likely pathogenic		26,90	0,824	0,588	0,726	0,136	0,763	1,045	0,075	0,370	-0,150	1,169	-0,325	1,535
Q247E	Likely pathogenic		25,70	0,822	0,519	0,388	0,105	0,895	2,469	0,004	0,400	-0,440	0,881	-0,744	1,255
Q247H	Likely pathogenic		22,90	0,799	0,556	0,932	0,174	0,937	4,316	0,004	0,470	-0,440	1,637	-0,539	1,935
Q247K	Likely pathogenic		27,00	0,921	0,561	0,944	0,206	0,932	4,628	0,004	1,700	-0,440	2,356	1,159	2,257
Q247L	Likely pathogenic		27,50	0,904	0,517	0,759	0,191	0,931	2,009	0,004	1,190	-0,440	1,871	0,483	1,916
Q247P	Likely pathogenic		24,40	0,863	0,524	0,336	0,251	0,937	0,913	0,004	0,120	-0,440	1,148	-1,008	1,494
Q247R	Likely pathogenic		26,60	0,918	0,555	0,883	0,131	0,948	6,808	0,004	0,560	-0,440	2,091	-0,322	2,303
C248F	Likely pathogenic		26,20	0,785	0,657	0,952	0,285	0,808	18,155	0,006	0,120	-0,270	2,686	-0,831	2,980
C248G	Likely pathogenic		29,00	0,757	0,616	0,565	0,283	0,767	0,786	0,006	0,750	-0,270	1,574	0,004	1,825
C248R	Likely pathogenic		29,40	0,877	0,712	0,969	0,343	0,847	6,514	0,006	1,660	-0,270	3,070	1,356	2,965
C248S	Likely pathogenic		22,60	0,697	0,636	0,534	0,181	0,488	0,313	0,006	0,730	-0,270	0,339	-0,377	0,846
C248W	Likely pathogenic		27,70	0,865	0,686	0,989	0,288	0,836	24,484	0,006	0,010	-0,270	3,356	-0,902	3,596
C248Y	Likely pathogenic		25,90	0,753	0,726	0,972	0,294	0,820	21,571	0,006	0,250	-0,270	2,980	-0,681	3,256
L249M	VUS		22,20	0,617	0,651	0,336	0,047	0,536	2,737	0,000	0,000	-0,600	-0,154	-1,652	0,664
L249P	Likely pathogenic		28,40	0,931	0,753	0,974	0,339	0,957	8,209	0,000	1,070	-0,600	3,458	0,469	3,481
L249Q	Likely pathogenic		28,10	0,922	0,669	0,849	0,276	0,949	3,538	0,000	1,190	-0,600	2,739	0,535	2,778
L249R	Likely pathogenic		28,30	0,944	0,726	0,921	0,336	0,947	11,951	0,000	1,750	-0,600	3,534	1,242	3,364
L249V	Likely pathogenic		23,10	0,704	0,619	0,298	0,078	0,695	3,383	0,000	0,110	-0,600	0,302	-1,407	0,961
G250A	Likely pathogenic		24,50	0,920	0,689	0,439	0,137	0,791	2,317	0,000	0,290	-0,480	1,366	-0,828	1,832
G250C	Likely pathogenic		28,10	0,898	0,726	0,830	0,216	0,915	3,683	0,000	0,750	-0,480	2,561	0,026	2,783
G250D	Likely pathogenic		28,00	0,890	0,764	0,968	0,293	0,923	6,856	0,000	0,460	-0,480	3,087	-0,261	3,322
G250R	Likely pathogenic		27,40	0,886	0,758	0,939	0,241	0,943	7,701	0,000	0,910	-0,480	2,963	0,230	3,114
G250S	Likely pathogenic		26,80	0,875	0,682	0,585	0,162	0,907	5,109	0,000	0,020	-0,480	1,913	-1,047	2,357
G250V	Likely pathogenic		27,80	0,858	0,695	0,887	0,238	0,925	10,494	0,000	0,730	-0,480	2,798	-0,064	2,970
F251C	Likely pathogenic		33,00	0,940	0,705	0,973	0,288	0,950	3,960	0,004	0,120	-0,570	3,296	-0,549	3,543
F251I	Likely pathogenic		29,60	0,891	0,684	0,928	0,196	0,938	5,448	0,004	0,100	-0,570	2,686	-0,780	3,031
F251L	Likely pathogenic	Severe	29,90	0,920	0,709	0,986	0,143	0,943	1,540	0,004	0,030	-0,570	2,601	-0,811	3,008
F251S	Likely pathogenic		33,00	0,937	0,682	0,986	0,231	0,950	4,528	0,004	0,850	-0,570	3,190	0,301	3,282
F251V	Likely pathogenic		29,80	0,935	0,666	0,942	0,258	0,927	3,512	0,004	0,140	-0,570	2,811	-0,661	3,094
F251Y	Likely pathogenic		28,00	0,760	0,694	0,538	0,122	0,775	0,670	0,004	0,370	-0,570	1,318	-0,725	1,824
T252A	Likely pathogenic		25,40	0,907	0,520	0,239	0,108	0,721	-0,134	0,320	0,220	-0,540	0,077	-0,422	0,438
T252I	Likely pathogenic		24,70	0,955	0,616	0,648	0,139	0,904	0,616	0,320	0,700	-0,540	1,212	0,399	1,367
T252N	Likely pathogenic		23,00	0,772	0,579	0,292	0,148	0,742	-0,627	0,320	0,390	-0,540	-0,032	-0,276	0,352
T252P	Likely pathogenic		25,90	0,943	0,515	0,256	0,264	0,697	-2,534	0,320	0,300	-0,540	0,432	-0,192	0,678
T252S	Likely pathogenic		21,60	0,767	0,524	0,119	0,044	0,398	-1,709	0,320	0,050	-0,540	-1,297	-0,972	-0,634
P253A	Likely pathogenic		14,01	0,597	0,405	0,065	0,099	0,291	2,569	0,082	0,520	-0,520	-1,988	-1,272	-1,292

P253H	Likely pathogenic		25,10	0,876	0,445	0,822	0,243	0,799	17,906	0,082	0,350	-0,520	1,974	-0,672	2,122	
P253L	Likely pathogenic		25,40	0,858	0,458	0,710	0,253	0,842	4,201	0,082	1,070	-0,520	1,446	0,314	1,491	
P253R	Likely pathogenic		25,20	0,900	0,461	0,658	0,274	0,833	3,602	0,082	0,680	-0,520	1,446	-0,148	1,569	
P253S	Likely pathogenic		22,10	0,751	0,393	0,271	0,115	0,423	3,612	0,082	0,250	-0,520	-0,670	-1,201	-0,114	
P253T	Likely pathogenic		22,20	0,814	0,408	0,528	0,216	0,752	5,034	0,082	0,300	-0,520	0,573	-0,869	0,890	
E254A	Likely pathogenic		23,50	0,781	0,565	0,153	-0,002	0,762	1,806	0,332	1,040	-0,450	-0,382	0,472	-0,100	
E254D	Likely pathogenic		22,90	0,710	0,563	0,379	-0,030	0,609	1,103	0,332	0,290	-0,450	-0,695	-0,494	-0,137	
E254G	Likely pathogenic		25,30	0,802	0,517	0,227	0,036	0,784	2,735	0,332	0,750	-0,450	-0,107	0,201	0,161	
E254K	Likely pathogenic		22,50	0,816	0,654	0,162	0,024	0,777	1,430	0,332	1,300	-0,450	-0,080	0,840	0,152	
E254Q	Likely pathogenic		20,80	0,763	0,547	0,116	-0,025	0,558	1,028	0,332	0,400	-0,450	-1,131	-0,489	-0,576	
E254V	Likely pathogenic		28,10	0,825	0,544	0,301	0,023	0,881	2,278	0,332	1,480	-0,450	0,427	1,245	0,469	
H255D	Likely pathogenic		22,70	0,704	0,392	0,194	-0,033	0,653	1,310	0,138	0,580	-0,370	-0,994	-0,608	-0,512	
H255L	Likely pathogenic		22,50	0,544	0,368	0,104	-0,033	0,341	-0,409	0,138	0,720	-0,370	-1,994	-0,649	-1,363	
H255N	Likely pathogenic		22,40	0,514	0,389	0,131	-0,071	0,405	1,119	0,138	0,440	-0,370	-1,932	-1,005	-1,222	
H255P	Likely pathogenic		23,80	0,741	0,363	0,715	0,170	0,768	2,338	0,138	0,350	-0,370	0,346	-0,558	0,639	
H255Q	Likely pathogenic	Severe	17,95	0,610	0,376	0,120	-0,126	0,262	0,450	0,138	0,470	-0,370	-2,474	-1,126	-1,689	Yes
H255R	Likely pathogenic		22,10	0,694	0,395	0,121	-0,033	0,544	0,701	0,138	1,030	-0,370	-1,280	-0,131	-0,850	
H255Y	Likely pathogenic		22,90	0,686	0,395	0,140	-0,021	0,545	0,661	0,138	0,380	-0,370	-1,240	-0,895	-0,663	
Q256E	Likely pathogenic		21,50	0,721	0,494	0,188	0,035	0,789	1,320	0,000	0,400	-0,310	-0,280	-0,978	0,249	
Q256H	Likely pathogenic		23,50	0,886	0,523	0,965	0,050	0,916	4,106	0,000	0,470	-0,310	1,447	-0,490	1,767	
Q256K	Likely pathogenic		24,10	0,918	0,534	0,915	0,161	0,929	0,289	0,000	1,700	-0,310	1,743	1,134	1,702	
Q256L	Likely pathogenic		24,60	0,907	0,483	0,646	0,057	0,831	-1,050	0,000	1,190	-0,310	0,849	0,340	1,037	
Q256P	Likely pathogenic		27,30	0,959	0,494	0,993	0,234	0,951	7,213	0,000	0,120	-0,310	2,410	-0,692	2,599	
Q256R	Likely pathogenic		26,80	0,954	0,528	0,929	0,160	0,947	3,864	0,000	0,560	-0,310	2,087	-0,190	2,260	
R257G	Likely pathogenic		25,60	0,786	0,534	0,645	0,237	0,880	3,285	0,204	0,910	-0,160	1,162	0,571	1,238	
R257L	Likely pathogenic		24,60	0,928	0,564	0,851	0,224	0,938	2,069	0,204	1,750	-0,160	1,726	1,734	1,542	
R257P	Likely pathogenic		24,90	0,946	0,575	0,995	0,436	0,945	8,328	0,204	0,680	-0,160	2,664	0,557	2,557	
R257Q	Pathogenic	Severe	24,70	0,942	0,491	0,355	0,269	0,912	2,659	0,204	0,560	-0,160	1,044	0,128	1,126	Yes
D258A	Likely pathogenic		27,70	0,905	0,500	0,640	0,127	0,942	-0,866	0,155	0,750	0,190	1,091	0,574	1,191	
D258E	Likely pathogenic		23,30	0,780	0,526	0,659	0,058	0,839	0,211	0,155	0,290	0,190	0,318	-0,238	0,697	
D258G	Likely pathogenic		27,90	0,922	0,511	0,722	0,216	0,952	0,453	0,155	0,460	0,190	1,516	0,304	1,611	
D258H	Likely pathogenic		24,40	0,938	0,572	0,763	0,134	0,941	-0,674	0,155	0,580	0,190	1,240	0,358	1,411	
D258N	Likely pathogenic		24,20	0,756	0,548	0,343	0,079	0,886	-0,960	0,155	0,140	0,190	0,099	-0,454	0,525	
D258V	Likely pathogenic		27,30	0,914	0,493	0,713	0,179	0,939	0,473	0,155	1,190	0,190	1,354	1,141	1,299	
D258Y	Likely pathogenic		24,60	0,959	0,556	0,663	0,250	0,945	-0,423	0,155	0,960	0,190	1,467	0,866	1,462	
F259C	Likely pathogenic		32,00	0,922	0,635	0,981	0,330	0,932	4,582	0,000	0,120	0,100	3,006	-0,221	3,167	
F259I	Likely pathogenic		27,20	0,889	0,619	0,945	0,306	0,901	5,762	0,000	0,100	0,100	2,468	-0,446	2,700	
F259L	Pathogenic		27,60	0,895	0,640	0,994	0,268	0,939	1,557	0,000	0,030	0,100	2,401	-0,468	2,683	
F259S	Likely pathogenic		32,00	0,928	0,610	0,993	0,273	0,956	4,702	0,000	0,850	0,100	2,928	0,644	2,923	
F259V	Likely pathogenic		27,30	0,884	0,592	0,941	0,300	0,880	2,854	0,000	0,140	0,100	2,237	-0,395	2,475	
F259Y	Likely pathogenic		28,70	0,785	0,632	0,714	0,254	0,782	1,122	0,000	0,370	0,100	1,679	-0,218	1,996	
I260F	Likely pathogenic		26,40	0,918	0,563	0,667	0,183	0,915	4,911	0,000	0,100	0,070	1,725	-0,630	2,024	
I260L	Likely pathogenic		22,80	0,712	0,546	0,222	0,082	0,541	-0,438	0,000	0,070	0,070	-0,474	-1,143	0,193	
I260M	Likely pathogenic		22,30	0,732	0,517	0,344	0,108	0,805	-0,146	0,000	0,070	0,070	0,063	-1,021	0,592	
I260N	Likely pathogenic		29,70	0,919	0,625	0,983	0,259	0,960	3,268	0,000	1,090	0,070	2,713	0,861	2,696	
I260S	Likely pathogenic		29,50	0,937	0,570	0,926	0,249	0,949	4,253	0,000	0,750	0,070	2,514	0,392	2,558	
I260T	Likely pathogenic		27,50	0,932	0,648	0,654	0,162	0,952	2,466	0,000	0,700	0,070	1,965	0,193	2,155	
I260V	Likely pathogenic		22,10	0,642	0,467	0,115	0,031	0,355	1,304	0,000	0,040	0,070	-1,292	-1,403	-0,522	
A261D	Likely pathogenic		25,40	0,765	0,599	0,690	0,210	0,849	4,985	0,109	0,750	0,590	1,239	0,597	1,389	
A261G	Likely pathogenic		23,50	0,655	0,484	0,142	0,035	0,626	1,407	0,109	0,290	0,590	-0,914	-0,421	-0,397	
A261P	Likely pathogenic		23,70	0,756	0,557	0,938	0,250	0,891	8,272	0,109	0,520	0,590	1,554	0,328	1,683	
A261S	VUS		20,80	0,634	0,464	0,091	0,004	0,291	0,620	0,109	0,270	0,590	-1,856	-0,679	-1,163	
A261T	Likely pathogenic		21,50	0,741	0,494	0,120	0,014	0,677	3,360	0,109	0,220	0,590	-0,791	-0,532	-0,285	
A261V	Likely pathogenic		22,40	0,676	0,520	0,157	0,001	0,468	2,606	0,109	0,440	0,590	-1,108	-0,322	-0,539	

R262C	Likely pathogenic		23,20	0,838	0,360	0,126	0,041	0,753	1,988	0,438	1,660	1,920	-1,287	2,766	-1,500	
R262G	Likely pathogenic		21,20	0,739	0,298	0,140	0,053	0,602	2,692	0,438	0,910	1,920	-1,969	1,673	-1,930	
R262H	Likely pathogenic		17,58	0,625	0,307	0,079	-0,015	0,453	-0,183	0,438	1,030	1,920	-2,982	1,583	-2,779	Yes
R262L	Likely pathogenic		16,62	0,586	0,320	0,121	0,001	0,349	0,226	0,438	1,750	1,920	-3,095	2,404	-3,016	
R262P	Likely pathogenic		22,10	0,755	0,321	0,847	0,242	0,849	5,928	0,438	0,680	1,920	-0,102	1,800	-0,284	
R262S	Likely pathogenic		20,10	0,681	0,299	0,276	0,018	0,436	2,467	0,438	0,930	1,920	-2,346	1,603	-2,206	
D263A	Likely pathogenic		27,50	0,820	0,536	0,849	0,188	0,952	-1,308	0,000	0,750	0,610	1,505	0,568	1,629	
D263E	Likely pathogenic		24,40	0,859	0,558	0,875	0,133	0,905	2,466	0,000	0,290	0,610	1,362	-0,123	1,645	
D263G	Likely pathogenic		27,60	0,852	0,539	0,846	0,199	0,950	-0,191	0,000	0,460	0,610	1,627	0,229	1,798	
D263H	Likely pathogenic		23,00	0,790	0,597	0,730	0,104	0,935	15,802	0,000	0,580	0,610	1,609	0,017	1,820	
D263N	Likely pathogenic		22,90	0,714	0,579	0,510	0,054	0,916	-0,650	0,000	0,140	0,610	0,315	-0,530	0,800	
D263V	Likely pathogenic		27,20	0,833	0,526	0,766	0,235	0,952	-0,096	0,000	1,190	0,610	1,589	1,092	1,569	
D263Y	Likely pathogenic		24,20	0,802	0,584	0,537	0,140	0,939	26,791	0,000	0,960	0,610	2,044	0,390	2,071	
L264I	Likely pathogenic	Mild	23,40	0,773	0,470	0,394	0,200	0,850	2,936	0,000	0,070	0,080	0,576	-0,928	0,965	
L264P	Likely pathogenic		27,20	0,938	0,554	0,986	0,410	0,957	11,220	0,000	1,070	0,080	3,087	0,770	2,919	
L264Q	Likely pathogenic		27,30	0,957	0,463	0,978	0,415	0,961	2,690	0,000	1,190	0,080	2,582	0,965	2,381	
L264R	Likely pathogenic		27,50	0,956	0,521	0,965	0,474	0,956	6,355	0,000	1,750	0,080	3,044	1,675	2,676	
L264V	Likely pathogenic		23,40	0,856	0,424	0,476	0,304	0,926	4,202	0,000	0,110	0,080	1,133	-0,760	1,354	
G265A	Likely pathogenic		24,80	0,902	0,635	0,584	0,264	0,931	7,501	0,019	0,290	0,390	1,911	-0,192	2,118	
G265C	Likely pathogenic		27,20	0,959	0,667	0,874	0,344	0,946	13,905	0,019	0,750	0,390	3,096	0,559	3,054	
G265D	Likely pathogenic		25,20	0,957	0,714	0,982	0,413	0,951	24,446	0,019	0,460	0,390	3,764	0,150	3,719	
G265R	Likely pathogenic		26,70	0,970	0,703	0,959	0,429	0,949	26,679	0,019	0,910	0,390	4,006	0,727	3,810	
G265S	Likely pathogenic		26,10	0,945	0,630	0,743	0,358	0,941	14,228	0,019	0,020	0,390	2,743	-0,421	2,877	
G265V	Likely pathogenic		25,20	0,953	0,639	0,837	0,363	0,952	20,624	0,019	0,730	0,390	3,164	0,410	3,084	
P266A	Likely pathogenic		24,60	0,948	0,475	0,407	0,322	0,936	2,744	0,176	0,520	0,500	1,138	0,448	1,138	
P266H	Likely pathogenic		29,60	0,942	0,527	0,563	0,361	0,941	2,966	0,176	0,350	0,500	1,873	0,464	1,861	
P266L	Pathogenic	Severe	31,00	0,938	0,542	0,471	0,303	0,942	2,732	0,176	1,070	0,500	1,805	1,331	1,660	
P266R	Likely pathogenic		29,70	0,949	0,535	0,368	0,392	0,948	1,165	0,176	0,680	0,500	1,732	0,855	1,643	
P266S	Likely pathogenic		25,20	0,943	0,484	0,555	0,254	0,927	3,252	0,176	0,250	0,500	1,180	0,134	1,279	
P266T	Likely pathogenic		24,80	0,946	0,512	0,497	0,334	0,954	3,142	0,176	0,300	0,500	1,391	0,234	1,437	
T267A	VUS		6,76	0,446	0,307	0,050	0,041	0,134	0,150	0,134	0,220	0,570	-3,819	-1,288	-2,969	
T267I	Likely pathogenic		20,20	0,679	0,377	0,121	0,090	0,476	-1,131	0,134	0,700	0,570	-1,574	-0,009	-1,159	
T267N	VUS		22,90	0,628	0,342	0,160	0,138	0,436	-0,477	0,134	0,390	0,570	-1,461	-0,333	-1,008	
T267P	VUS		18,55	0,681	0,322	0,348	0,315	0,589	3,925	0,134	0,300	0,570	-0,680	-0,378	-0,403	
T267S	VUS		21,70	0,578	0,310	0,103	0,028	0,383	0,522	0,134	0,050	0,570	-2,108	-0,924	-1,472	
L268F	VUS		23,90	0,889	0,459	0,311	0,194	0,735	1,207	0,045	0,030	0,610	0,285	-0,565	0,635	
L268H	Likely pathogenic		27,50	0,911	0,526	0,944	0,387	0,937	2,348	0,045	0,720	0,610	2,303	0,766	2,233	
L268I	Likely pathogenic		23,60	0,781	0,468	0,175	0,124	0,652	0,832	0,045	0,070	0,610	-0,372	-0,677	0,101	
L268P	Likely pathogenic		28,10	0,920	0,561	0,962	0,428	0,950	6,150	0,045	1,070	0,610	2,765	1,230	2,564	
L268R	Likely pathogenic		28,00	0,923	0,527	0,899	0,425	0,943	1,097	0,045	1,750	0,610	2,439	2,066	2,095	
L268V	Likely pathogenic		23,50	0,830	0,420	0,230	0,170	0,598	2,206	0,045	0,110	0,610	-0,252	-0,615	0,153	
A269D	Likely pathogenic		22,00	0,731	0,541	0,233	0,095	0,568	1,087	0,364	0,750	1,050	-1,004	0,991	-0,743	
A269G	Likely pathogenic		22,10	0,688	0,453	0,141	0,012	0,360	1,178	0,364	0,290	1,050	-1,929	0,231	-1,446	
A269P	Likely pathogenic		21,40	0,804	0,508	0,781	0,213	0,759	1,673	0,364	0,520	1,050	0,195	1,000	0,290	
A269S	Likely pathogenic		15,59	0,710	0,441	0,094	-0,036	0,298	0,682	0,364	0,270	1,050	-2,654	-0,026	-2,071	
A269T	Likely pathogenic		16,59	0,665	0,469	0,097	-0,016	0,321	0,810	0,364	0,220	1,050	-2,518	-0,052	-1,921	
A269V	Likely pathogenic		22,00	0,749	0,479	0,141	-0,023	0,427	0,887	0,364	0,440	1,050	-1,743	0,458	-1,308	
N270D	VUS		21,10	0,537	0,423	0,105	-0,001	0,384	1,511	0,754	0,140	1,580	-3,028	1,023	-2,641	
N270H	VUS		22,60	0,617	0,401	0,081	0,018	0,531	-0,445	0,754	0,440	1,580	-2,641	1,532	-2,433	
N270I	Likely pathogenic		22,60	0,765	0,403	0,276	0,131	0,709	0,759	0,754	1,090	1,580	-1,507	2,570	-1,669	
N270K	VUS		22,30	0,566	0,441	0,158	-0,012	0,312	0,090	0,754	1,730	1,580	-2,861	3,018	-2,836	
N270S	VUS		17,94	0,489	0,366	0,068	-0,044	0,280	0,348	0,754	0,340	1,580	-3,809	1,072	-3,379	
N270T	Likely pathogenic		20,10	0,675	0,391	0,143	0,017	0,352	1,046	0,754	0,390	1,580	-2,874	1,370	-2,607	
N270Y	Likely pathogenic		24,10	0,751	0,414	0,157	0,122	0,667	0,213	0,754	0,820	1,580	-1,660	2,230	-1,725	

S271C	Likely pathogenic		27,50	0,867	0,447	0,229	0,102	0,858	2,747	0,123	0,730	1,200	0,231	0,830	0,322	
S271G	Likely pathogenic	Mild	24,20	0,737	0,415	0,131	0,044	0,849	2,096	0,123	0,020	1,200	-0,663	-0,265	-0,287	Yes
S271I	Likely pathogenic		26,70	0,857	0,457	0,760	0,200	0,932	4,368	0,123	0,750	1,200	1,183	1,051	1,149	
S271N	Likely pathogenic	Mild	22,60	0,663	0,474	0,451	0,049	0,870	2,614	0,123	0,340	1,200	-0,335	0,167	-0,007	
S271R	Likely pathogenic		24,70	0,761	0,482	0,850	0,126	0,813	3,505	0,123	0,930	1,200	0,641	1,130	0,715	
S271T	Likely pathogenic		25,40	0,804	0,441	0,226	0,037	0,753	1,517	0,123	0,050	1,200	-0,467	-0,156	-0,087	
T272A	VUS		13,00	0,552	0,343	0,066	-0,055	0,137	-0,495	0,663	0,220	1,210	-4,177	0,362	-3,589	
T272I	VUS		17,23	0,647	0,400	0,136	-0,013	0,275	-0,645	0,663	0,700	1,210	-3,152	1,228	-2,806	
T272N	VUS		15,56	0,519	0,375	0,093	0,004	0,189	-0,095	0,663	0,390	1,210	-3,708	0,692	-3,214	
T272P	VUS		14,28	0,600	0,349	0,059	0,107	0,193	-1,890	0,663	0,300	1,210	-3,570	0,639	-3,139	
T272S	VUS		13,74	0,500	0,341	0,076	-0,104	0,126	-0,275	0,663	0,050	1,210	-4,356	0,118	-3,680	
H273D	VUS		24,00	0,610	0,490	0,580	0,129	0,709	2,827	0,116	0,580	0,960	-0,122	0,352	0,184	
H273L	VUS		24,10	0,597	0,467	0,290	0,048	0,508	-0,123	0,116	0,720	0,960	-1,117	0,338	-0,691	
H273N	VUS		23,70	0,546	0,492	0,306	0,012	0,588	1,627	0,116	0,440	0,960	-1,081	-0,033	-0,571	
H273P	VUS		26,80	0,621	0,449	0,411	0,172	0,700	2,874	0,116	0,350	0,960	-0,113	0,109	0,194	
H273Q	VUS		23,00	0,585	0,471	0,469	-0,018	0,624	1,437	0,116	0,470	0,960	-0,953	0,039	-0,474	
H273R	VUS		26,00	0,651	0,494	0,202	0,045	0,704	-0,128	0,116	1,030	0,960	-0,608	0,847	-0,344	
H273Y	VUS		20,00	0,630	0,494	0,103	-0,051	0,271	0,544	0,116	0,380	0,960	-2,075	-0,360	-1,378	
H274D	VUS		17,12	0,608	0,216	0,105	-0,043	0,135	0,577	0,464	0,580	0,920	-3,615	0,328	-3,142	
H274L	VUS		8,93	0,536	0,207	0,115	-0,020	0,134	-0,137	0,464	0,720	0,920	-4,303	0,253	-3,787	
H274N	VUS		16,46	0,449	0,215	0,089	-0,093	0,123	-0,501	0,464	0,440	0,920	-4,175	0,032	-3,559	
H274P	VUS		8,59	0,513	0,212	0,081	0,066	0,125	0,542	0,464	0,350	0,920	-4,206	-0,190	-3,636	
H274Q	VUS		7,36	0,607	0,200	0,092	-0,117	0,122	-0,426	0,464	0,470	0,920	-4,604	-0,127	-3,977	
H274R	VUS		5,65	0,479	0,209	0,048	-0,066	0,162	-2,102	0,464	1,030	0,920	-4,838	0,495	-4,315	
H274Y	VUS		20,50	0,414	0,212	0,110	-0,106	0,196	-0,107	0,464	0,380	0,920	-3,850	0,074	-3,261	
N275D	VUS		7,18	0,432	0,286	0,054	-0,047	0,154	0,036	0,482	0,140	0,760	-4,566	-0,607	-3,803	
N275H	VUS		10,17	0,403	0,284	0,062	-0,039	0,239	0,322	0,482	0,440	0,760	-4,228	-0,139	-3,588	
N275I	VUS		18,03	0,555	0,287	0,276	0,105	0,480	-0,052	0,482	1,090	0,760	-2,406	1,177	-2,233	
N275K	VUS		14,67	0,364	0,296	0,140	-0,037	0,164	-0,268	0,482	1,730	0,760	-3,907	1,551	-3,571	
N275S	VUS		12,64	0,382	0,267	0,066	-0,066	0,322	-0,126	0,482	0,340	0,760	-4,099	-0,192	-3,465	
N275T	VUS		13,89	0,443	0,282	0,112	-0,007	0,288	0,621	0,482	0,390	0,760	-3,680	-0,031	-3,117	
N275Y	VUS		19,11	0,608	0,291	0,163	0,096	0,385	-0,221	0,482	0,820	0,760	-2,535	0,836	-2,275	
V276A	VUS		23,70	0,648	0,337	0,358	0,093	0,570	1,828	0,080	0,440	0,070	-0,796	-0,580	-0,362	
V276D	VUS		24,40	0,673	0,340	0,736	0,260	0,786	4,018	0,080	1,190	0,070	0,593	0,626	0,610	
V276F	VUS		21,70	0,642	0,324	0,444	0,171	0,579	2,781	0,080	0,140	0,070	-0,670	-0,949	-0,214	
V276G	VUS		24,20	0,664	0,298	0,443	0,219	0,606	3,420	0,080	0,730	0,070	-0,286	-0,127	-0,055	
V276I	VUS		12,92	0,473	0,288	0,076	-0,004	0,227	0,104	0,080	0,040	0,070	-3,162	-1,714	-2,286	
V276L	VUS		14,15	0,553	0,330	0,315	0,062	0,241	0,710	0,080	0,110	0,070	-2,350	-1,432	-1,579	
R277C	VUS	Mild	23,10	0,635	0,218	0,105	0,082	0,766	0,541	0,412	1,660	0,160	-1,595	1,569	-1,673	
R277G	VUS		21,20	0,678	0,208	0,167	0,176	0,522	1,432	0,412	0,910	0,160	-1,760	0,595	-1,639	
R277H	VUS		11,72	0,375	0,210	0,069	0,013	0,182	0,068	0,412	1,030	0,160	-4,053	0,105	-3,530	
R277L	VUS		12,15	0,527	0,209	0,146	0,052	0,236	-0,627	0,412	1,750	0,160	-3,451	1,140	-3,211	
R277P	VUS		19,35	0,651	0,214	0,445	0,283	0,705	1,640	0,412	0,680	0,160	-1,100	0,451	-1,050	
R277S	VUS		16,99	0,638	0,209	0,243	0,054	0,445	1,223	0,412	0,930	0,160	-2,462	0,412	-2,199	
L278I	VUS		15,50	0,619	0,436	0,114	0,045	0,222	0,438	0,025	0,070	-0,850	-1,924	-2,086	-1,011	
L278P	VUS		24,30	0,864	0,522	0,904	0,359	0,943	3,358	0,025	1,070	-0,850	2,262	0,162	2,276	
L278Q	VUS		24,20	0,763	0,449	0,922	0,297	0,923	3,653	0,025	1,190	-0,850	1,750	0,187	1,786	
L278R	VUS		24,30	0,787	0,488	0,910	0,356	0,933	3,126	0,025	1,750	-0,850	2,061	0,940	1,931	
L278V	VUS		15,45	0,634	0,401	0,148	0,089	0,232	1,670	0,025	0,110	-0,850	-1,770	-2,017	-0,929	
L279F	VUS		15,94	0,632	0,340	0,189	0,098	0,332	0,061	0,000	0,030	-0,880	-1,698	-2,131	-0,891	
L279H	VUS		23,70	0,751	0,388	0,887	0,292	0,892	4,838	0,000	0,720	-0,880	1,505	-0,533	1,658	
L279I	VUS		11,98	0,525	0,356	0,226	0,019	0,273	1,485	0,000	0,070	-0,880	-2,322	-2,304	-1,385	
L279P	VUS		24,00	0,900	0,413	0,941	0,333	0,952	5,373	0,000	1,070	-0,880	2,164	0,041	2,129	
L279R	VUS		23,80	0,819	0,387	0,920	0,330	0,926	8,520	0,000	1,750	-0,880	2,045	0,774	1,862	

L279V	VUS		15,14	0,634	0,313	0,270	0,092	0,428	2,473	0,000	0,110	-0,880	-1,495	-2,033	-0,764
M280I	VUS		10,45	0,464	0,377	0,166	0,025	0,231	1,742	0,000	0,070	-0,980	-2,577	-2,456	-1,574
M280K	VUS		23,00	0,776	0,414	0,946	0,280	0,848	4,620	0,000	2,890	-0,980	1,708	2,063	1,369
M280L	VUS		15,68	0,484	0,366	0,131	0,018	0,286	1,029	0,000	0,000	-0,980	-2,206	-2,380	-1,249
M280R	VUS		23,20	0,799	0,395	0,940	0,307	0,859	9,371	0,000	1,750	-0,980	1,907	0,656	1,780
M280T	VUS		21,70	0,647	0,459	0,547	0,098	0,552	3,361	0,000	0,770	-0,980	-0,050	-0,917	0,462
M280V	VUS		14,83	0,559	0,329	0,097	0,073	0,272	3,173	0,000	0,110	-0,980	-2,042	-2,249	-1,187
L281M	VUS		21,40	0,614	0,489	0,260	0,075	0,468	0,294	0,000	0,000	-0,980	-0,744	-1,970	0,065
L281P	VUS		27,50	0,916	0,597	0,948	0,288	0,945	13,606	0,000	1,070	-0,980	3,128	0,070	3,131
L281Q	VUS		27,20	0,885	0,515	0,804	0,293	0,938	2,306	0,000	1,190	-0,980	2,239	0,212	2,264
L281R	VUS		27,40	0,909	0,565	0,904	0,353	0,938	7,417	0,000	1,750	-0,980	2,921	0,954	2,748
L281V	VUS		22,30	0,695	0,468	0,398	0,093	0,705	1,768	0,000	0,110	-0,980	0,041	-1,661	0,660
D282A	Likely pathogenic		26,10	0,941	0,542	0,988	0,272	0,954	1,393	0,000	0,750	-0,940	2,431	-0,240	2,558
D282E	VUS		22,80	0,773	0,570	0,965	0,209	0,913	5,134	0,000	0,290	-0,940	1,825	-1,053	2,202
D282G	Likely pathogenic		26,30	0,943	0,553	0,972	0,351	0,954	1,657	0,000	0,460	-0,940	2,636	-0,547	2,787
D282H	Likely pathogenic		24,70	0,945	0,613	0,994	0,349	0,954	25,287	0,000	0,580	-0,940	3,666	-0,610	3,694
D282N	VUS		24,80	0,915	0,591	0,974	0,307	0,957	0,096	0,000	0,140	-0,940	2,379	-0,988	2,681
D282V	Likely pathogenic		26,00	0,950	0,540	0,996	0,387	0,950	3,839	0,000	1,190	-0,940	2,847	0,343	2,780
D282Y	Likely pathogenic		24,80	0,954	0,597	0,991	0,385	0,947	28,474	0,000	0,960	-0,940	3,886	-0,154	3,773
D283A	VUS		29,00	0,946	0,491	0,994	0,328	0,942	0,441	0,000	0,750	-0,900	2,597	-0,118	2,649
D283E	VUS		24,30	0,883	0,515	0,982	0,195	0,852	4,279	0,000	0,290	-0,900	1,863	-0,972	2,193
D283G	VUS		29,20	0,948	0,505	0,979	0,266	0,949	1,560	0,000	0,460	-0,900	2,519	-0,506	2,676
D283H	VUS		24,90	0,808	0,561	0,990	0,257	0,935	15,246	0,000	0,580	-0,900	2,625	-0,645	2,799
D283N	VUS		25,00	0,934	0,538	0,960	0,294	0,940	1,311	0,000	0,140	-0,900	2,274	-0,999	2,549
D283V	VUS		28,70	0,942	0,490	0,997	0,375	0,939	2,619	0,000	1,190	-0,900	2,801	0,417	2,709
D283Y	VUS		25,00	0,955	0,545	0,989	0,373	0,940	5,379	0,000	0,960	-0,900	2,779	0,035	2,777
Q284E	VUS		22,80	0,686	0,334	0,256	0,082	0,424	1,466	0,129	0,400	-0,030	-1,218	-0,679	-0,725
Q284H	VUS		20,20	0,578	0,350	0,740	0,088	0,569	-1,611	0,129	0,470	-0,030	-0,923	-0,494	-0,452
Q284K	VUS		23,20	0,730	0,361	0,844	0,188	0,605	-1,595	0,129	1,700	-0,030	0,104	1,270	0,116
Q284L	VUS		23,70	0,830	0,324	0,682	0,101	0,737	-1,811	0,129	1,190	-0,030	0,005	0,648	0,101
Q284P	VUS		23,50	0,857	0,342	0,859	0,329	0,813	0,106	0,129	0,120	-0,030	0,939	-0,459	1,102
Q284R	VUS		23,40	0,818	0,354	0,776	0,187	0,706	-3,200	0,129	0,560	-0,030	0,190	-0,050	0,418
R285C	Pathogenic		24,10	0,866	0,580	0,757	0,393	0,933	0,817	0,051	1,660	0,690	1,890	1,840	1,670
R285G	Likely pathogenic		23,50	0,884	0,502	0,964	0,420	0,949	1,866	0,051	0,910	0,690	1,998	0,954	1,883
R285H	Pathogenic	Severe	23,70	0,942	0,525	0,803	0,354	0,954	1,517	0,051	1,030	0,690	1,845	1,067	1,738
R285L	Likely pathogenic		23,70	0,898	0,530	0,903	0,311	0,901	-0,817	0,051	1,750	0,690	1,660	1,934	1,449
R285P	Likely pathogenic		23,90	0,942	0,546	0,982	0,526	0,958	0,915	0,051	0,680	0,690	2,476	0,806	2,345
R285S	Likely pathogenic		23,20	0,872	0,513	0,987	0,385	0,950	1,318	0,051	0,930	0,690	1,904	0,961	1,818
L286F	VUS		16,01	0,650	0,426	0,195	-0,014	0,344	0,190	0,348	0,030	-0,010	-2,346	-0,928	-1,642
L286M	VUS		16,75	0,634	0,418	0,196	-0,037	0,358	0,385	0,348	0,000	-0,010	-2,371	-0,964	-1,654
L286S	VUS		22,60	0,702	0,441	0,646	0,090	0,651	2,110	0,348	0,820	-0,010	-0,406	0,529	-0,177
L286V	VUS		11,95	0,638	0,396	0,108	-0,038	0,220	0,714	0,348	0,110	-0,010	-3,036	-1,046	-2,264
L286W	VUS		24,00	0,770	0,485	0,411	0,108	0,793	0,947	0,348	0,080	-0,010	-0,179	-0,284	0,171
L287M	VUS		21,40	0,616	0,301	0,217	-0,060	0,373	0,414	0,189	0,000	-0,310	-2,086	-1,392	-1,359
L287P	VUS		23,20	0,881	0,368	0,812	0,221	0,897	4,850	0,189	1,070	-0,310	1,078	0,594	1,025
L287Q	VUS		22,80	0,795	0,316	0,292	0,069	0,458	1,602	0,189	1,190	-0,310	-0,956	0,309	-0,715
L287R	VUS		23,00	0,814	0,353	0,454	0,150	0,729	1,059	0,189	1,750	-0,310	-0,020	1,212	-0,066
L287V	VUS		17,26	0,687	0,289	0,285	-0,041	0,373	3,341	0,189	0,110	-0,310	-2,022	-1,336	-1,356
L288M	VUS		23,10	0,747	0,524	0,635	0,159	0,731	-0,002	0,035	0,000	-0,290	0,522	-1,126	1,045
L288P	Likely pathogenic		28,20	0,941	0,630	0,981	0,429	0,959	1,241	0,035	1,070	-0,290	3,004	0,780	2,917
L288Q	Likely pathogenic		27,80	0,928	0,548	0,970	0,367	0,946	3,105	0,035	1,190	-0,290	2,661	0,819	2,550
L288R	Likely pathogenic		28,10	0,942	0,600	0,978	0,426	0,950	11,814	0,035	1,750	-0,290	3,388	1,504	3,069
L288V	VUS		22,90	0,838	0,494	0,530	0,170	0,736	4,363	0,035	0,110	-0,290	0,715	-1,023	1,139
P289A	Likely pathogenic		24,60	0,869	0,576	0,749	0,256	0,905	3,270	0,157	0,520	-0,090	1,565	0,117	1,715

P289H	Likely pathogenic		27,80	0,921	0,619	0,870	0,221	0,940	4,048	0,157	0,350	-0,090	2,117	0,063	2,277
P289L	Likely pathogenic	Mild	28,20	0,887	0,636	0,722	0,220	0,944	2,061	0,157	1,070	-0,090	1,930	0,919	1,953
P289R	Likely pathogenic		27,70	0,948	0,628	0,835	0,250	0,934	4,007	0,157	0,680	-0,090	2,225	0,485	2,287
P289S	Likely pathogenic		25,20	0,890	0,579	0,818	0,177	0,831	3,418	0,157	0,250	-0,090	1,421	-0,234	1,694
P289T	Likely pathogenic		24,90	0,902	0,607	0,752	0,192	0,923	3,796	0,157	0,300	-0,090	1,605	-0,147	1,836
H290D	VUS		19,83	0,595	0,291	0,189	-0,050	0,526	-0,478	0,478	0,580	-0,090	-2,472	0,033	-2,053
H290L	VUS		21,50	0,534	0,282	0,119	-0,047	0,288	-0,629	0,478	0,720	-0,090	-2,915	0,118	-2,431
H290N	VUS		18,52	0,447	0,292	0,120	-0,095	0,343	0,298	0,478	0,440	-0,090	-3,280	-0,361	-2,662
H290P	VUS		22,70	0,655	0,275	0,202	0,079	0,705	-0,073	0,478	0,350	-0,090	-1,603	-0,012	-1,328
H290Q	VUS		22,00	0,649	0,276	0,159	-0,126	0,333	-0,732	0,478	0,470	-0,090	-2,772	-0,134	-2,249
H290R	VUS		20,40	0,482	0,294	0,080	-0,069	0,212	-0,185	0,478	1,030	-0,090	-3,228	0,393	-2,744
H290Y	VUS		17,54	0,474	0,293	0,073	-0,141	0,247	-1,431	0,478	0,380	-0,090	-3,674	-0,505	-2,969
W291C	VUS		26,80	0,884	0,657	0,985	0,417	0,948	4,253	0,025	0,010	-0,280	2,875	-0,622	3,088
W291G	VUS		28,50	0,912	0,622	0,898	0,466	0,949	5,611	0,025	0,760	-0,280	3,100	0,333	3,059
W291L	VUS		26,00	0,841	0,659	0,937	0,347	0,900	2,019	0,025	0,080	-0,280	2,364	-0,629	2,660
W291R	VUS		27,30	0,819	0,694	0,986	0,465	0,951	2,251	0,025	1,670	-0,280	3,035	1,448	2,862
W291S	VUS		26,70	0,911	0,644	0,934	0,403	0,937	5,356	0,025	0,740	-0,280	2,882	0,240	2,912
A292E	VUS		26,10	0,922	0,610	0,942	0,177	0,944	11,469	0,000	1,040	-0,340	2,578	0,327	2,636
A292G	VUS		26,10	0,869	0,545	0,305	0,108	0,833	2,004	0,000	0,290	-0,340	0,844	-0,824	1,258
A292P	VUS		23,00	0,781	0,608	0,618	0,211	0,903	0,757	0,000	0,520	-0,340	1,268	-0,466	1,608
A292S	VUS		24,10	0,825	0,528	0,244	0,068	0,747	0,662	0,000	0,270	-0,340	0,227	-0,991	0,740
A292T	VUS		24,40	0,852	0,561	0,350	0,063	0,897	4,034	0,000	0,220	-0,340	0,844	-0,966	1,292
A292V	VUS		23,80	0,768	0,576	0,425	0,056	0,884	2,811	0,000	0,440	-0,340	0,689	-0,726	1,138
K293E	VUS		16,19	0,406	0,323	0,075	-0,007	0,177	0,526	0,356	1,300	0,080	-3,326	0,445	-2,829
K293M	VUS		23,00	0,740	0,304	0,240	0,009	0,621	-0,163	0,356	2,890	0,080	-1,285	2,950	-1,545
K293N	VUS		20,10	0,404	0,337	0,208	-0,011	0,212	0,742	0,356	1,730	0,080	-2,792	1,130	-2,444
K293Q	VUS		15,68	0,393	0,320	0,080	-0,031	0,147	0,682	0,356	1,700	0,080	-3,456	0,888	-3,023
K293R	VUS		18,89	0,571	0,323	0,075	-0,090	0,321	0,380	0,356	1,140	0,080	-2,828	0,410	-2,376
K293T	VUS		19,48	0,603	0,325	0,127	0,016	0,394	1,958	0,356	2,120	0,080	-2,159	1,727	-2,066
V294A	VUS		24,70	0,799	0,597	0,190	0,051	0,742	0,339	0,121	0,440	-0,450	0,132	-0,593	0,618
V294E	VUS		25,00	0,834	0,586	0,307	0,175	0,850	-0,029	0,121	1,480	-0,450	0,843	0,842	0,934
V294G	VUS		25,50	0,862	0,490	0,315	0,196	0,840	1,723	0,121	0,730	-0,450	0,766	-0,090	0,963
V294L	VUS		22,80	0,742	0,578	0,494	0,049	0,676	0,136	0,121	0,110	-0,450	0,047	-1,019	0,647
V294M	VUS		24,60	0,829	0,607	0,264	0,059	0,772	0,016	0,121	0,110	-0,450	0,315	-0,941	0,856
V295A	VUS		26,30	0,892	0,621	0,676	0,125	0,922	2,557	0,000	0,440	-0,130	1,656	-0,329	1,969
V295E	Likely pathogenic		27,30	0,953	0,610	0,939	0,248	0,947	5,409	0,000	1,480	-0,130	2,635	1,125	2,540
V295G	Likely pathogenic		27,20	0,942	0,520	0,731	0,264	0,897	4,303	0,000	0,730	-0,130	2,027	0,104	2,108
V295I	VUS		22,10	0,661	0,482	0,095	0,042	0,645	0,138	0,000	0,040	-0,130	-0,792	-1,394	-0,113
V295L	VUS		24,20	0,846	0,603	0,499	0,119	0,914	-0,927	0,000	0,110	-0,130	0,988	-0,850	1,446
L296M	VUS		24,50	0,799	0,542	0,235	0,097	0,864	0,600	0,080	0,000	0,140	0,248	-0,823	0,715
L296P	VUS		28,00	0,931	0,641	0,951	0,370	0,957	8,128	0,080	1,070	0,140	2,953	1,014	2,829
L296Q	VUS		27,60	0,893	0,563	0,673	0,307	0,956	2,509	0,080	1,190	0,140	1,987	1,031	1,908
L296R	VUS		27,90	0,863	0,617	0,658	0,283	0,951	2,525	0,080	1,750	0,140	2,041	1,712	1,876
L296V	VUS		24,00	0,889	0,516	0,305	0,115	0,845	3,933	0,080	0,110	0,140	0,559	-0,672	0,929
T297A	VUS		9,59	0,361	0,319	0,057	-0,068	0,105	0,526	0,599	0,220	0,760	-4,709	-0,258	-3,958
T297I	VUS		15,54	0,614	0,373	0,192	-0,015	0,325	0,038	0,599	0,700	0,760	-3,060	0,789	-2,667
T297K	VUS		14,42	0,456	0,354	0,094	0,011	0,182	-0,378	0,599	2,120	0,760	-3,656	2,349	-3,501
T297P	VUS		14,29	0,679	0,336	0,196	0,123	0,281	0,414	0,599	0,300	0,760	-2,858	0,342	-2,470
T297R	VUS		15,02	0,576	0,347	0,078	0,022	0,196	-0,098	0,599	0,980	0,760	-3,446	1,032	-3,080
T297S	VUS		8,72	0,481	0,308	0,065	-0,112	0,185	0,330	0,599	0,050	0,760	-4,566	-0,428	-3,820
D298A	VUS		28,30	0,906	0,474	0,414	0,146	0,936	2,237	0,223	0,750	1,320	0,671	1,290	0,635
D298E	VUS		24,00	0,864	0,496	0,470	0,078	0,906	1,331	0,223	0,290	1,320	0,123	0,571	0,313
D298G	VUS		28,50	0,919	0,481	0,690	0,162	0,938	2,683	0,223	0,460	1,320	1,071	1,036	1,069
D298H	VUS		23,90	0,866	0,537	0,380	0,145	0,938	3,123	0,223	0,580	1,320	0,422	0,948	0,501

D298N	VUS		22,70	0,738	0,516	0,180	0,085	0,746	1,196	0,223	0,140	1,320	-0,719	0,164	-0,350	
D298V	VUS		28,00	0,923	0,467	0,575	0,193	0,931	2,416	0,223	1,190	1,320	0,989	1,894	0,799	
D298Y	VUS		24,00	0,901	0,523	0,457	0,183	0,936	1,323	0,223	0,960	1,320	0,589	1,484	0,538	
P299A	VUS		22,10	0,600	0,332	0,072	-0,045	0,350	1,509	0,566	0,520	1,170	-2,879	0,844	-2,513	
P299L	VUS		22,70	0,705	0,371	0,111	0,017	0,492	1,197	0,566	1,070	1,170	-2,115	1,697	-2,003	
P299Q	VUS		21,40	0,630	0,333	0,103	-0,003	0,347	1,640	0,566	0,120	1,170	-2,763	0,380	-2,334	
P299R	VUS		22,60	0,726	0,373	0,082	0,045	0,553	1,113	0,566	0,680	1,170	-1,983	1,257	-1,820	
P299S	VUS		22,50	0,629	0,325	0,098	-0,044	0,407	1,859	0,566	0,250	1,170	-2,702	0,561	-2,313	
P299T	VUS		22,40	0,638	0,346	0,081	-0,025	0,425	1,892	0,566	0,300	1,170	-2,584	0,644	-2,219	
E300A	VUS		21,20	0,634	0,392	0,088	-0,063	0,431	0,161	0,448	1,040	1,780	-2,616	1,642	-2,370	
E300D	VUS		17,75	0,577	0,392	0,119	-0,106	0,280	-0,011	0,448	0,290	1,780	-3,328	0,535	-2,769	
E300G	VUS		22,00	0,647	0,366	0,118	-0,033	0,488	0,668	0,448	0,750	1,780	-2,410	1,345	-2,159	
E300K	VUS		17,56	0,609	0,440	0,078	-0,053	0,394	-0,525	0,448	1,300	1,780	-2,859	1,861	-2,605	
E300Q	VUS		16,80	0,674	0,375	0,086	-0,099	0,502	-0,443	0,448	0,400	1,780	-2,948	0,758	-2,534	
E300V	VUS		21,50	0,680	0,382	0,124	-0,056	0,498	0,055	0,448	1,480	1,780	-2,348	2,244	-2,264	
A301E	Likely pathogenic		27,70	0,955	0,572	0,913	0,242	0,865	10,634	0,000	1,040	0,740	2,420	1,004	2,354	
A301G	VUS		27,30	0,912	0,519	0,351	0,173	0,710	1,342	0,000	0,290	0,740	0,730	-0,143	1,033	
A301P	Likely pathogenic		24,90	0,952	0,569	0,908	0,301	0,947	2,874	0,000	0,520	0,740	2,111	0,411	2,170	
A301S	VUS		24,30	0,845	0,506	0,198	0,128	0,765	0,761	0,000	0,270	0,740	0,137	-0,335	0,511	
A301T	VUS		24,50	0,898	0,534	0,181	0,126	0,852	0,974	0,000	0,220	0,740	0,432	-0,331	0,777	
A301V	VUS		27,50	0,905	0,542	0,252	0,124	0,804	2,415	0,000	0,440	0,740	0,752	0,020	1,027	
A302D	VUS		27,30	0,875	0,591	0,347	0,098	0,863	1,407	0,240	0,750	0,450	0,639	0,763	0,796	
A302G	VUS		23,20	0,706	0,498	0,116	-0,005	0,505	1,117	0,240	0,290	0,450	-1,275	-0,278	-0,736	
A302P	VUS		23,90	0,916	0,552	0,801	0,150	0,884	6,771	0,240	0,520	0,450	1,246	0,514	1,351	
A302S	VUS		20,40	0,686	0,484	0,080	-0,035	0,411	0,986	0,240	0,270	0,450	-1,801	-0,454	-1,179	
A302T	VUS		22,50	0,723	0,510	0,108	-0,019	0,675	1,249	0,240	0,220	0,450	-1,055	-0,324	-0,549	
A302V	VUS		27,10	0,847	0,532	0,190	-0,004	0,798	0,563	0,240	0,440	0,450	-0,141	0,224	0,180	
K303E	Likely pathogenic		22,50	0,723	0,541	0,125	0,012	0,530	1,013	0,470	1,300	0,730	-1,403	1,616	-1,220	
K303I	Likely pathogenic		24,30	0,774	0,528	0,448	0,117	0,905	0,189	0,470	2,820	0,730	0,037	3,830	-0,410	
K303N	Likely pathogenic		22,20	0,559	0,563	0,317	0,008	0,383	1,206	0,470	1,730	0,730	-1,660	2,062	-1,465	
K303Q	Likely pathogenic		20,40	0,651	0,508	0,103	-0,019	0,357	0,730	0,470	1,700	0,730	-2,105	1,917	-1,904	
K303R	Likely pathogenic		20,10	0,605	0,501	0,070	-0,092	0,435	0,241	0,470	1,140	0,730	-2,384	1,180	-2,009	
K303T	Likely pathogenic		22,70	0,750	0,552	0,154	0,025	0,644	1,154	0,470	2,120	0,730	-1,010	2,695	-1,087	
Y304C	Likely pathogenic	Severe	32,00	0,899	0,676	0,545	0,351	0,943	2,274	0,251	0,250	0,320	2,170	0,492	2,241	Yes
Y304D	Likely pathogenic		25,30	0,895	0,671	0,724	0,395	0,918	4,775	0,251	0,960	0,320	2,100	1,216	2,006	
Y304F	Likely pathogenic		24,50	0,792	0,605	0,141	0,124	0,619	-0,540	0,251	0,370	0,320	-0,310	0,019	0,103	
Y304H	Likely pathogenic		25,10	0,875	0,680	0,406	0,210	0,647	1,400	0,251	0,380	0,320	0,690	0,237	1,004	
Y304N	Likely pathogenic		25,30	0,863	0,676	0,557	0,374	0,933	2,299	0,251	0,820	0,320	1,729	0,997	1,716	
Y304S	Likely pathogenic		29,70	0,905	0,623	0,680	0,385	0,928	2,892	0,251	0,480	0,320	2,148	0,739	2,127	
V305A	Likely pathogenic		25,00	0,828	0,600	0,517	0,119	0,749	2,595	0,075	0,440	-0,240	0,866	-0,425	1,262	
V305D	Likely pathogenic		25,90	0,891	0,592	0,957	0,372	0,924	5,996	0,075	1,190	-0,240	2,581	0,843	2,490	
V305F	Likely pathogenic		22,90	0,814	0,558	0,604	0,194	0,891	4,040	0,075	0,140	-0,240	1,116	-0,772	1,477	
V305G	Likely pathogenic		25,60	0,901	0,494	0,670	0,332	0,778	3,670	0,075	0,730	-0,240	1,585	0,102	1,673	
V305I	Likely pathogenic		20,60	0,644	0,438	0,071	0,036	0,369	-0,541	0,075	0,040	-0,240	-1,595	-1,472	-0,828	
V305L	Likely pathogenic		21,60	0,794	0,574	0,333	0,122	0,650	0,681	0,075	0,110	-0,240	0,052	-1,021	0,615	
H306D	VUS		16,76	0,583	0,618	0,114	0,020	0,314	-0,015	0,321	0,580	-0,480	-1,855	-0,530	-1,140	
H306L	Likely pathogenic		16,76	0,723	0,590	0,267	0,055	0,703	-0,944	0,321	0,720	-0,480	-0,849	-0,097	-0,403	
H306N	VUS		16,15	0,572	0,619	0,103	-0,011	0,266	0,154	0,321	0,440	-0,480	-2,078	-0,761	-1,283	
H306P	Likely pathogenic		17,33	0,758	0,563	0,319	0,236	0,762	1,367	0,321	0,350	-0,480	-0,160	-0,411	0,197	
H306Q	VUS		15,03	0,621	0,592	0,208	-0,040	0,618	-0,648	0,321	0,470	-0,480	-1,588	-0,601	-0,936	
H306R	Likely pathogenic		15,68	0,648	0,620	0,147	0,037	0,297	-0,006	0,321	1,030	-0,480	-1,719	0,036	-1,135	
H306Y	VUS		16,23	0,604	0,621	0,148	-0,033	0,386	-1,550	0,321	0,380	-0,480	-1,905	-0,760	-1,131	
G307A	Likely pathogenic		25,10	0,927	0,700	0,561	0,299	0,936	2,627	0,000	0,290	-0,610	2,224	-0,708	2,534	
G307C	Likely pathogenic		27,60	0,961	0,736	0,819	0,426	0,945	4,463	0,000	0,750	-0,610	3,263	0,088	3,333	

G307D	Likely pathogenic		25,50	0,963	0,777	0,962	0,427	0,948	9,485	0,000	0,460	-0,610	3,565	-0,306	3,700
G307R	Likely pathogenic		27,00	0,968	0,770	0,951	0,443	0,945	7,681	0,000	0,910	-0,610	3,647	0,306	3,658
G307S	Likely pathogenic		26,40	0,967	0,693	0,600	0,373	0,944	3,338	0,000	0,020	-0,610	2,617	-0,936	2,908
G307V	Likely pathogenic		25,50	0,969	0,709	0,928	0,453	0,944	8,958	0,000	0,730	-0,610	3,435	0,008	3,463
I308F	Likely pathogenic		23,00	0,883	0,590	0,499	0,172	0,835	10,482	0,000	0,100	-0,750	1,542	-1,333	1,970
I308L	Likely pathogenic		22,10	0,723	0,575	0,189	0,095	0,471	0,363	0,000	0,070	-0,750	-0,349	-1,653	0,407
I308M	Likely pathogenic		22,10	0,664	0,550	0,191	0,109	0,678	0,647	0,000	0,070	-0,750	-0,162	-1,615	0,521
I308N	Likely pathogenic		27,30	0,906	0,654	0,914	0,334	0,929	4,614	0,000	1,090	-0,750	2,862	0,324	2,903
I308S	Likely pathogenic		27,40	0,946	0,595	0,818	0,269	0,910	5,548	0,000	0,750	-0,750	2,530	-0,170	2,661
I308T	Likely pathogenic		25,60	0,857	0,673	0,546	0,164	0,801	3,608	0,000	0,700	-0,750	1,620	-0,452	1,980
I308V	Likely pathogenic		17,64	0,598	0,498	0,086	0,039	0,279	0,982	0,000	0,040	-0,750	-1,592	-2,037	-0,670
A309D	Likely pathogenic		28,90	0,958	0,688	0,976	0,323	0,921	7,340	0,000	0,750	-0,510	3,224	0,118	3,303
A309G	Likely pathogenic		22,60	0,630	0,582	0,179	0,128	0,428	1,530	0,000	0,290	-0,510	-0,451	-1,283	0,259
A309P	Likely pathogenic		24,40	0,952	0,655	0,962	0,363	0,927	3,927	0,000	0,520	-0,510	2,749	-0,257	2,901
A309S	Likely pathogenic		23,90	0,838	0,563	0,226	0,138	0,739	2,017	0,000	0,270	-0,510	0,548	-1,055	1,038
A309T	Likely pathogenic		24,20	0,906	0,598	0,508	0,139	0,782	3,269	0,000	0,220	-0,510	1,209	-0,973	1,655
A309V	Likely pathogenic		28,60	0,943	0,618	0,820	0,209	0,928	3,951	0,000	0,440	-0,510	2,410	-0,383	2,639
V310A	Likely pathogenic		26,20	0,877	0,685	0,717	0,154	0,742	2,227	0,011	0,440	-0,240	1,610	-0,385	2,008
V310E	Likely pathogenic		27,00	0,919	0,668	0,947	0,345	0,909	4,807	0,011	1,480	-0,240	2,852	1,131	2,757
V310G	Likely pathogenic		26,90	0,909	0,580	0,795	0,347	0,750	3,512	0,011	0,730	-0,240	2,108	0,084	2,236
V310I	Likely pathogenic		17,72	0,645	0,519	0,086	0,055	0,338	-1,013	0,011	0,040	-0,240	-1,521	-1,640	-0,671
V310L	Likely pathogenic		19,76	0,742	0,662	0,329	0,130	0,618	-0,972	0,011	0,110	-0,240	0,020	-1,185	0,696
H311D	Likely pathogenic		25,20	0,954	0,747	0,994	0,342	0,953	6,119	0,013	0,580	-0,010	3,030	0,183	3,147
H311L	Likely pathogenic		26,10	0,928	0,731	0,981	0,345	0,930	-0,644	0,013	0,720	-0,010	2,692	0,404	2,816
H311N	Likely pathogenic		24,80	0,928	0,750	0,985	0,302	0,944	1,002	0,013	0,440	-0,010	2,615	0,002	2,837
H311P	Likely pathogenic		26,10	0,945	0,704	0,966	0,458	0,951	0,635	0,013	0,350	-0,010	2,974	0,005	3,086
H311Q	Likely pathogenic		23,20	0,888	0,733	0,986	0,195	0,916	0,987	0,013	0,470	-0,010	2,094	-0,100	2,406
H311R	Likely pathogenic	Severe	25,40	0,952	0,751	0,972	0,331	0,946	1,926	0,013	1,030	-0,010	2,847	0,758	2,889
H311Y	Likely pathogenic		24,60	0,951	0,749	0,979	0,267	0,937	4,873	0,013	0,380	-0,010	2,699	-0,121	2,932
W312C	Likely pathogenic	Mild	28,00	0,907	0,817	0,973	0,480	0,924	-0,817	0,179	0,010	1,810	2,650	1,076	2,701
W312G	Likely pathogenic		29,80	0,929	0,793	0,748	0,530	0,922	0,390	0,179	0,760	1,810	2,734	1,996	2,549
W312L	Likely pathogenic		26,90	0,839	0,818	0,963	0,424	0,874	-2,322	0,179	0,080	1,810	2,169	1,060	2,301
W312R	Likely pathogenic		28,40	0,928	0,836	0,991	0,528	0,932	-0,457	0,179	1,670	1,810	3,048	3,167	2,657
W312S	Pathogenic		27,70	0,920	0,809	0,791	0,475	0,898	-0,904	0,179	0,740	1,810	2,433	1,900	2,326
Y313C	Likely pathogenic		28,60	0,939	0,798	0,981	0,423	0,932	1,266	0,171	0,250	2,490	2,570	1,728	2,512
Y313D	Likely pathogenic		27,80	0,931	0,792	0,945	0,466	0,923	1,053	0,171	0,960	2,490	2,580	2,580	2,336
Y313F	Likely pathogenic		26,30	0,931	0,743	0,796	0,290	0,780	1,289	0,171	0,370	2,490	1,523	1,606	1,584
Y313H	Likely pathogenic		27,50	0,952	0,801	0,987	0,360	0,916	1,482	0,171	0,380	2,490	2,376	1,824	2,334
Y313N	Likely pathogenic		27,70	0,932	0,796	0,891	0,445	0,934	1,388	0,171	0,820	2,490	2,493	2,381	2,296
Y313S	Likely pathogenic		27,70	0,939	0,754	0,927	0,457	0,937	1,522	0,171	0,480	2,490	2,458	1,969	2,312
L314M	VUS		22,50	0,497	0,531	0,110	-0,064	0,244	0,234	0,289	0,000	2,350	-2,682	0,339	-1,993
L314P	Likely pathogenic		26,20	0,788	0,647	0,888	0,218	0,787	2,892	0,289	1,070	2,350	0,940	2,484	0,836
L314Q	VUS		23,70	0,597	0,557	0,405	0,070	0,542	0,716	0,289	1,190	2,350	-1,147	2,144	-0,998
L314R	Likely pathogenic		23,00	0,691	0,619	0,528	0,136	0,701	-0,973	0,289	1,750	2,350	-0,369	3,020	-0,459
L314V	Likely pathogenic		22,40	0,824	0,507	0,187	-0,039	0,684	1,321	0,289	0,110	2,350	-1,270	0,800	-0,932
D315A	Likely pathogenic		27,60	0,942	0,693	0,960	0,277	0,902	-1,200	0,130	0,750	2,640	1,800	2,196	1,702
D315E	Likely pathogenic		23,80	0,849	0,714	0,928	0,140	0,835	-1,463	0,130	0,290	2,640	0,898	1,385	1,100
D315G	Likely pathogenic		27,70	0,932	0,698	0,913	0,220	0,895	-0,161	0,130	0,460	2,640	1,625	1,786	1,638
D315H	Likely pathogenic		26,00	0,972	0,753	0,978	0,286	0,922	-0,499	0,130	0,580	2,640	1,977	1,994	1,930
D315N	Likely pathogenic		24,10	0,798	0,737	0,807	0,146	0,796	-1,121	0,130	0,140	2,640	0,702	1,146	0,986
D315V	Likely pathogenic		27,40	0,940	0,695	0,982	0,324	0,920	-0,401	0,130	1,190	2,640	2,017	2,760	1,773
D315Y	Likely pathogenic		26,10	0,958	0,746	0,952	0,249	0,919	-2,005	0,130	0,960	2,640	1,783	2,435	1,678
F316C	Likely pathogenic		23,10	0,743	0,739	0,553	0,114	0,618	1,276	0,613	0,120	3,170	-0,761	2,189	-0,577
F316I	VUS		22,90	0,626	0,714	0,241	0,006	0,448	0,904	0,613	0,100	3,170	-1,932	1,886	-1,554

F316L	VUS		22,80	0,579	0,746	0,803	-0,036	0,219	0,515	0,613	0,030	3,170	-1,792	1,852	-1,292	
F316S	Likely pathogenic		21,10	0,651	0,708	0,384	0,027	0,244	1,542	0,613	0,850	3,170	-2,038	2,741	-1,793	
F316V	Likely pathogenic		23,00	0,714	0,707	0,252	0,006	0,386	1,322	0,613	0,140	3,170	-1,833	1,957	-1,484	
F316Y	VUS		18,84	0,638	0,734	0,245	-0,050	0,201	0,248	0,613	0,370	3,170	-2,651	2,008	-2,175	
L317M	Likely pathogenic		23,90	0,657	0,640	0,214	-0,001	0,427	-0,036	0,552	0,000	2,920	-1,969	1,498	-1,559	
L317P	Likely pathogenic		26,40	0,836	0,738	0,744	0,193	0,588	4,445	0,552	1,070	2,920	0,336	3,283	0,200	
L317Q	Likely pathogenic		25,60	0,814	0,655	0,742	0,128	0,571	1,309	0,552	1,190	2,920	-0,265	3,346	-0,388	
L317R	Likely pathogenic		26,20	0,873	0,714	0,770	0,190	0,612	0,927	0,552	1,750	2,920	0,299	4,160	-0,016	
L317V	Likely pathogenic		21,20	0,708	0,609	0,137	-0,003	0,270	1,844	0,552	0,110	2,920	-2,374	1,478	-1,949	
A318D	Likely pathogenic		25,20	0,732	0,619	0,765	0,051	0,631	0,326	0,186	0,750	3,070	-0,206	2,073	-0,045	
A318G	Likely pathogenic		24,50	0,680	0,526	0,187	-0,050	0,242	0,948	0,186	0,290	3,070	-2,043	1,067	-1,544	
A318P	Likely pathogenic		23,00	0,756	0,590	0,822	0,086	0,575	1,282	0,186	0,520	3,070	-0,296	1,740	-0,094	
A318S	Likely pathogenic		20,40	0,605	0,516	0,148	-0,080	0,178	-0,406	0,186	0,270	3,070	-2,766	0,849	-2,153	
A318T	Likely pathogenic		20,80	0,585	0,539	0,135	-0,074	0,204	-0,785	0,186	0,220	3,070	-2,701	0,810	-2,071	
A318V	Likely pathogenic		22,70	0,576	0,556	0,128	-0,083	0,192	-0,581	0,186	0,440	3,070	-2,568	1,121	-1,987	
P319A	Likely pathogenic		24,10	0,797	0,692	0,283	0,170	0,614	1,061	0,597	0,520	2,960	-0,788	2,514	-0,742	
P319L	Likely pathogenic		27,10	0,911	0,749	0,524	0,213	0,789	1,129	0,597	1,070	2,960	0,451	3,502	0,213	
P319Q	Likely pathogenic		26,50	0,838	0,700	0,472	0,266	0,653	1,120	0,597	0,120	2,960	-0,050	2,236	-0,025	
P319R	Likely pathogenic		26,70	0,896	0,743	0,447	0,307	0,779	1,358	0,597	0,680	2,960	0,486	3,029	0,298	
P319S	Likely pathogenic		23,20	0,798	0,691	0,256	0,150	0,592	1,485	0,597	0,250	2,960	-0,965	2,129	-0,827	
P319T	Likely pathogenic		24,60	0,839	0,715	0,344	0,167	0,732	1,621	0,597	0,300	2,960	-0,372	2,341	-0,332	
A320D	Likely pathogenic		27,80	0,827	0,681	0,629	0,116	0,578	-0,031	0,054	0,750	2,460	0,520	1,577	0,736	
A320G	Likely pathogenic		23,20	0,691	0,587	0,269	0,013	0,302	1,853	0,054	0,290	2,460	-1,297	0,511	-0,743	
A320P	VUS		20,50	0,615	0,650	0,114	0,131	0,282	-2,120	0,054	0,520	2,460	-1,549	0,751	-1,005	
A320S	Likely pathogenic		22,10	0,717	0,574	0,160	-0,022	0,252	1,016	0,054	0,270	2,460	-1,674	0,402	-1,069	
A320T	Likely pathogenic		22,50	0,747	0,600	0,183	-0,005	0,365	0,216	0,054	0,220	2,460	-1,327	0,439	-0,762	
A320V	Likely pathogenic		23,40	0,689	0,621	0,178	-0,014	0,337	0,787	0,054	0,440	2,460	-1,352	0,692	-0,804	
K321E	VUS		16,85	0,460	0,606	0,069	-0,059	0,119	-0,892	0,627	1,300	2,710	-3,676	2,640	-3,316	
K321I	Likely pathogenic		22,70	0,698	0,589	0,364	-0,024	0,378	-0,213	0,627	2,820	2,710	-1,917	4,959	-2,212	
K321N	VUS		20,20	0,445	0,632	0,219	-0,054	0,124	0,177	0,627	1,730	2,710	-3,149	3,301	-2,922	
K321Q	Likely pathogenic		17,14	0,646	0,572	0,108	-0,082	0,201	-0,023	0,627	1,700	2,710	-3,210	3,235	-3,059	
K321R	VUS		22,20	0,595	0,559	0,087	-0,146	0,170	0,218	0,627	1,140	2,710	-3,235	2,607	-2,922	
K321T	Likely pathogenic		22,30	0,665	0,614	0,122	-0,037	0,216	0,586	0,627	2,120	2,710	-2,509	3,947	-2,527	
A322D	VUS		20,50	0,640	0,588	0,097	-0,001	0,273	0,529	0,411	0,750	1,580	-2,183	1,137	-1,731	
A322G	Likely pathogenic		19,44	0,648	0,486	0,116	-0,077	0,250	1,319	0,411	0,290	1,580	-2,684	0,456	-2,110	
A322P	Likely pathogenic		17,21	0,649	0,547	0,166	0,050	0,229	0,041	0,411	0,520	1,580	-2,401	0,788	-1,906	
A322S	VUS		15,54	0,494	0,470	0,085	-0,122	0,165	-0,070	0,411	0,270	1,580	-3,614	0,190	-2,883	
A322T	VUS		16,44	0,570	0,508	0,080	-0,105	0,169	-0,008	0,411	0,220	1,580	-3,276	0,215	-2,566	
A322V	VUS		18,73	0,616	0,513	0,086	-0,112	0,237	0,916	0,411	0,440	1,580	-2,872	0,586	-2,274	
T323A	Likely pathogenic		21,80	0,646	0,666	0,180	0,084	0,456	1,388	0,023	0,220	0,950	-0,675	-0,471	-0,009	
T323I	Pathogenic	Severe	25,90	0,704	0,750	0,642	0,019	0,675	4,272	0,023	0,700	0,950	0,760	0,442	1,202	Yes
T323N	Likely pathogenic		25,50	0,694	0,721	0,479	0,058	0,583	0,562	0,023	0,390	0,950	0,247	0,009	0,800	
T323P	Likely pathogenic		24,50	0,838	0,656	0,395	0,180	0,714	2,540	0,023	0,300	0,950	0,763	-0,013	1,134	
T323S	Likely pathogenic		22,40	0,633	0,666	0,183	-0,052	0,290	-0,758	0,023	0,050	0,950	-1,326	-0,781	-0,462	
L324I	Likely pathogenic		21,70	0,682	0,631	0,247	0,126	0,585	1,427	0,000	0,070	0,430	-0,202	-0,915	0,441	
L324P	Likely pathogenic		26,70	0,933	0,715	0,938	0,420	0,938	3,689	0,000	1,070	0,430	2,987	1,068	2,914	Yes
L324Q	Likely pathogenic		26,40	0,932	0,633	0,915	0,357	0,923	2,693	0,000	1,190	0,430	2,537	1,135	2,455	
L324R	Likely pathogenic		26,60	0,931	0,686	0,921	0,416	0,920	5,696	0,000	1,750	0,430	2,987	1,855	2,737	
L324V	Likely pathogenic		21,40	0,743	0,592	0,211	0,149	0,413	2,424	0,000	0,110	0,430	-0,396	-0,924	0,244	
G325A	Likely pathogenic		17,54	0,598	0,611	0,125	0,021	0,233	0,342	0,135	0,290	0,640	-1,868	-0,619	-1,081	
G325E	Likely pathogenic		17,08	0,611	0,673	0,146	0,069	0,412	0,548	0,135	0,750	0,640	-1,282	0,054	-0,679	Yes
G325R	Likely pathogenic	Severe	17,10	0,593	0,689	0,213	0,096	0,692	0,851	0,135	0,910	0,640	-0,697	0,375	-0,244	
G325V	Likely pathogenic		19,59	0,726	0,614	0,268	0,099	0,384	3,184	0,135	0,730	0,640	-0,757	0,152	-0,270	
G325W	Likely pathogenic		23,10	0,727	0,674	0,417	0,133	0,861	0,178	0,135	0,760	0,640	0,472	0,561	0,760	

E326A	VUS		15,69	0,507	0,530	0,153	-0,109	0,184	0,281	0,242	1,040	0,970	-2,878	0,499	-2,228	
E326D	VUS		18,03	0,508	0,529	0,189	-0,133	0,266	0,599	0,242	0,290	0,970	-2,653	-0,330	-1,859	
E326G	VUS		18,64	0,622	0,487	0,273	0,016	0,263	1,470	0,242	0,750	0,970	-1,978	0,387	-1,463	
E326K	VUS	Risk variant	15,49	0,595	0,600	0,134	-0,082	0,557	0,126	0,242	1,300	0,970	-1,946	1,021	-1,511	Yes
E326Q	VUS		15,16	0,614	0,504	0,145	-0,128	0,207	0,292	0,242	0,400	0,970	-2,842	-0,261	-2,081	
E326V	VUS		17,97	0,493	0,502	0,229	-0,090	0,245	0,306	0,242	1,480	0,970	-2,555	1,135	-2,076	
T327A	VUS		24,10	0,857	0,602	0,396	0,136	0,761	1,039	0,006	0,220	0,260	0,703	-0,564	1,141	
T327I	VUS		25,80	0,867	0,699	0,698	0,158	0,700	1,625	0,006	0,700	0,260	1,427	0,188	1,757	
T327K	VUS		26,30	0,924	0,679	0,961	0,306	0,912	3,334	0,006	2,120	0,260	2,659	2,173	2,414	
T327P	VUS		24,80	0,927	0,593	0,844	0,387	0,871	4,415	0,006	0,300	0,260	2,270	-0,139	2,407	
T327R	VUS		26,10	0,944	0,668	0,941	0,241	0,927	10,125	0,006	0,980	0,260	2,695	0,687	2,714	
T327S	VUS		24,00	0,898	0,609	0,596	0,118	0,786	1,499	0,006	0,050	0,260	1,013	-0,699	1,466	
H328D	VUS		25,30	0,940	0,644	0,634	0,261	0,934	-0,356	0,196	0,580	0,410	1,544	0,652	1,638	
H328L	VUS		26,20	0,878	0,617	0,333	0,188	0,918	-1,575	0,196	0,720	0,410	0,853	0,684	0,998	
H328N	VUS		24,70	0,870	0,642	0,310	0,138	0,901	0,127	0,196	0,440	0,410	0,670	0,252	0,938	
H328P	VUS		26,20	0,925	0,588	0,702	0,309	0,943	1,785	0,196	0,350	0,410	1,720	0,397	1,795	
H328Q	VUS		23,30	0,744	0,618	0,379	0,092	0,901	-0,405	0,196	0,470	0,410	0,221	0,176	0,558	
H328R	VUS		25,50	0,902	0,656	0,155	0,169	0,932	-1,000	0,196	1,030	0,410	0,768	1,009	0,866	
H328Y	VUS		24,50	0,922	0,655	0,150	0,102	0,905	-1,094	0,196	0,380	0,410	0,475	0,149	0,783	
R329C	VUS	Mild	31,00	0,780	0,470	0,102	0,119	0,862	0,901	0,566	1,660	0,760	-0,252	2,671	-0,500	Yes
R329G	VUS		23,50	0,689	0,400	0,134	0,070	0,517	0,808	0,566	0,910	0,760	-1,776	1,337	-1,623	
R329H	VUS		18,01	0,601	0,410	0,064	-0,020	0,343	0,671	0,566	1,030	0,760	-2,856	1,163	-2,526	
R329L	VUS		19,22	0,613	0,420	0,125	0,035	0,616	0,145	0,566	1,750	0,760	-2,086	2,233	-2,076	
R329P	VUS		21,00	0,759	0,432	0,736	0,253	0,747	3,272	0,566	0,680	0,760	-0,212	1,367	-0,250	
R329S	VUS		23,00	0,630	0,405	0,189	0,024	0,365	0,187	0,566	0,930	0,760	-2,211	1,264	-1,951	
L330M	VUS		22,60	0,612	0,609	0,168	0,001	0,481	0,794	0,279	0,000	0,170	-1,228	-0,722	-0,520	
L330P	VUS		25,20	0,834	0,722	0,827	0,270	0,890	4,727	0,279	1,070	0,170	1,869	1,260	1,876	
L330Q	VUS		24,50	0,717	0,629	0,117	0,125	0,818	2,231	0,279	1,190	0,170	0,044	1,003	0,211	
L330R	VUS		22,50	0,617	0,683	0,123	0,170	0,777	1,881	0,279	1,750	0,170	-0,080	1,619	0,018	
L330V	VUS		18,72	0,673	0,577	0,161	0,097	0,666	1,607	0,279	0,110	0,170	-0,922	-0,571	-0,380	
F331C	VUS		30,00	0,856	0,708	0,760	0,199	0,862	4,013	0,142	0,120	0,030	2,044	-0,186	2,354	
F331I	VUS		25,30	0,801	0,693	0,805	0,166	0,830	3,032	0,142	0,100	0,030	1,455	-0,382	1,856	
F331L	VUS		25,20	0,817	0,713	0,961	0,126	0,746	2,870	0,142	0,030	0,030	1,468	-0,459	1,934	
F331S	VUS		29,90	0,869	0,687	0,844	0,210	0,914	5,127	0,142	0,850	0,030	2,309	0,740	2,390	
F331V	VUS		25,40	0,860	0,667	0,791	0,237	0,864	3,752	0,142	0,140	0,030	1,751	-0,271	2,048	
F331Y	VUS		23,10	0,608	0,703	0,205	0,086	0,297	2,376	0,142	0,370	0,030	-0,700	-0,599	0,021	
P332A	VUS		25,30	0,876	0,660	0,345	0,237	0,912	1,910	0,453	0,520	0,340	0,801	0,913	0,880	
P332H	VUS		27,50	0,909	0,706	0,500	0,343	0,926	2,722	0,453	0,350	0,340	1,595	0,903	1,619	
P332L	VUS		28,00	0,891	0,719	0,549	0,285	0,915	2,267	0,453	1,070	0,340	1,562	1,776	1,459	
P332R	VUS		27,60	0,907	0,720	0,310	0,306	0,937	1,329	0,453	0,680	0,340	1,317	1,254	1,306	
P332S	VUS		26,00	0,879	0,666	0,357	0,228	0,883	1,717	0,453	0,250	0,340	0,791	0,597	0,948	
P332T	Likely pathogenic		25,60	0,934	0,688	0,459	0,248	0,921	2,431	0,453	0,300	0,340	1,170	0,731	1,269	
N333D	VUS		7,89	0,405	0,410	0,049	0,049	0,239	0,242	0,815	0,140	0,560	-4,333	0,093	-3,713	
N333H	VUS		18,63	0,567	0,408	0,078	0,081	0,360	0,134	0,815	0,440	0,560	-2,965	0,906	-2,632	
N333I	VUS		26,90	0,739	0,417	0,369	0,205	0,706	0,373	0,815	1,090	0,560	-0,845	2,288	-1,021	
N333K	VUS		23,00	0,569	0,436	0,147	0,082	0,471	-0,166	0,815	1,730	0,560	-2,261	2,678	-2,308	
N333S	VUS		23,40	0,562	0,382	0,067	0,043	0,427	0,496	0,815	0,340	0,560	-2,696	0,900	-2,387	
N333T	VUS		23,90	0,637	0,401	0,131	0,091	0,461	0,458	0,815	0,390	0,560	-2,235	1,076	-2,009	
N333Y	VUS		20,30	0,666	0,421	0,171	0,195	0,542	-0,434	0,815	0,820	0,560	-1,975	1,628	-1,920	
T334A	VUS		22,30	0,626	0,463	0,083	0,107	0,309	0,754	0,140	0,220	0,190	-1,465	-0,811	-0,822	
T334I	VUS		20,30	0,637	0,526	0,105	0,057	0,319	-1,945	0,140	0,700	0,190	-1,591	-0,244	-0,986	
T334N	VUS		19,77	0,524	0,497	0,147	0,095	0,391	0,570	0,140	0,390	0,190	-1,588	-0,669	-0,939	
T334P	VUS		22,90	0,722	0,474	0,305	0,204	0,630	1,004	0,140	0,300	0,190	-0,242	-0,419	0,149	
T334S	VUS		22,10	0,596	0,466	0,126	0,071	0,333	1,418	0,140	0,050	0,190	-1,517	-1,043	-0,811	

M335I	VUS		20,90	0,566	0,545	0,292	0,095	0,360	1,155	0,129	0,070	-0,310	-1,085	-1,271	-0,324
M335K	VUS		22,80	0,652	0,593	0,279	0,251	0,497	2,934	0,129	2,890	-0,310	0,172	2,406	0,064
M335L	VUS		21,90	0,607	0,541	0,093	0,070	0,299	2,249	0,129	0,000	-0,310	-1,277	-1,412	-0,479
M335R	VUS		23,00	0,661	0,579	0,231	0,352	0,483	2,717	0,129	1,750	-0,310	0,245	1,055	0,351
M335T	VUS		22,50	0,570	0,619	0,140	0,155	0,252	3,455	0,129	0,770	-0,310	-0,827	-0,406	-0,217
M335V	VUS		21,60	0,593	0,501	0,070	0,138	0,333	2,481	0,129	0,110	-0,310	-1,212	-1,268	-0,504
L336F	VUS		26,50	0,900	0,692	0,583	0,154	0,744	4,415	0,010	0,030	-0,600	1,682	-1,131	2,194
L336H	Likely pathogenic		29,00	0,947	0,748	0,918	0,331	0,928	3,902	0,010	0,720	-0,600	3,177	0,089	3,311
L336I	VUS		23,20	0,634	0,697	0,133	0,056	0,355	0,305	0,010	0,070	-0,600	-0,524	-1,586	0,367
L336P	Likely pathogenic		29,70	0,950	0,779	0,856	0,372	0,956	2,678	0,010	1,070	-0,600	3,353	0,575	3,385
L336R	Likely pathogenic		29,60	0,953	0,752	0,882	0,301	0,936	7,823	0,010	1,750	-0,600	3,380	1,314	3,254
L336V	VUS		25,40	0,831	0,660	0,211	0,116	0,635	1,337	0,010	0,110	-0,600	0,612	-1,251	1,233
F337C	VUS		29,40	0,922	0,719	0,920	0,287	0,861	4,225	0,000	0,120	-1,080	2,966	-1,004	3,328
F337I	VUS		22,50	0,675	0,699	0,422	0,165	0,638	1,942	0,000	0,100	-1,080	0,691	-1,619	1,404
F337L	VUS		21,60	0,615	0,719	0,868	0,116	0,378	0,998	0,000	0,030	-1,080	0,506	-1,731	1,374
F337S	Likely pathogenic		29,20	0,933	0,702	0,971	0,298	0,860	5,873	0,000	0,850	-1,080	3,135	-0,112	3,290
F337V	VUS		25,40	0,824	0,681	0,535	0,190	0,668	2,827	0,000	0,140	-1,080	1,405	-1,374	1,981
F337Y	VUS		27,10	0,863	0,711	0,557	0,211	0,636	1,553	0,000	0,370	-1,080	1,662	-0,996	2,171
A338D	VUS		26,60	0,880	0,703	0,904	0,205	0,934	0,276	0,031	0,750	-0,700	2,309	-0,048	2,558
A338G	VUS		22,50	0,527	0,607	0,169	0,015	0,344	1,173	0,031	0,290	-0,700	-1,023	-1,464	-0,156
A338P	VUS		23,20	0,839	0,672	0,736	0,166	0,890	3,633	0,031	0,520	-0,700	1,697	-0,569	2,063
A338S	VUS		21,50	0,662	0,588	0,101	-0,011	0,362	0,307	0,031	0,270	-0,700	-1,027	-1,478	-0,189
A338T	VUS		24,20	0,819	0,618	0,334	0,026	0,707	-1,311	0,031	0,220	-0,700	0,321	-1,155	0,946
A338V	VUS		26,10	0,790	0,640	0,503	0,023	0,703	-1,390	0,031	0,440	-0,700	0,639	-0,792	1,209
S339A	VUS		22,70	0,715	0,658	0,390	0,148	0,670	1,172	0,000	0,270	-0,350	0,491	-0,982	1,080
S339L	VUS		28,50	0,911	0,684	0,767	0,293	0,931	5,872	0,000	0,820	-0,350	2,721	0,199	2,828
S339P	VUS		24,70	0,886	0,672	0,934	0,301	0,858	5,362	0,000	0,250	-0,350	2,410	-0,589	2,703
S339T	VUS		18,14	0,450	0,670	0,079	0,094	0,314	1,669	0,000	0,050	-0,350	-1,306	-1,750	-0,338
E340A	Likely pathogenic		27,70	0,933	0,742	0,980	0,321	0,937	-0,180	0,040	1,040	0,420	2,714	1,136	2,719
E340D	VUS		23,60	0,896	0,742	0,993	0,286	0,907	0,606	0,040	0,290	0,420	2,219	0,055	2,479
E340G	Likely pathogenic		29,00	0,941	0,705	0,975	0,346	0,938	-1,618	0,040	0,750	0,420	2,709	0,832	2,747
E340K	Likely pathogenic		25,10	0,952	0,801	0,960	0,343	0,959	3,443	0,040	1,300	0,420	2,938	1,399	2,878
E340Q	VUS		24,30	0,931	0,725	0,956	0,293	0,958	0,299	0,040	0,400	0,420	2,341	0,232	2,529
E340V	Likely pathogenic		28,40	0,943	0,732	0,996	0,334	0,952	0,722	0,040	1,480	0,420	2,900	1,704	2,764
A341D	Likely pathogenic		28,20	0,961	0,764	0,969	0,328	0,944	3,295	0,008	0,750	1,090	2,904	1,114	2,912
A341G	Likely pathogenic		28,00	0,862	0,680	0,353	0,235	0,825	1,071	0,008	0,290	1,090	1,294	0,211	1,574
A341P	Likely pathogenic		25,90	0,951	0,739	0,986	0,436	0,952	4,697	0,008	0,520	1,090	2,993	0,809	2,989
A341S	Likely pathogenic		23,80	0,814	0,661	0,279	0,194	0,783	-0,139	0,008	0,270	1,090	0,571	-0,009	0,951
A341T	Likely pathogenic	Severe	25,80	0,952	0,699	0,845	0,285	0,943	-0,855	0,008	0,220	1,090	2,112	0,343	2,301
A341V	Likely pathogenic		28,00	0,968	0,702	0,895	0,282	0,938	0,350	0,008	0,440	1,090	2,412	0,683	2,519
C342F	Likely pathogenic		27,70	0,943	0,764	0,969	0,431	0,910	5,996	0,012	0,120	1,800	2,946	0,762	2,999
C342G	Likely pathogenic	Mild	26,00	0,898	0,718	0,663	0,429	0,915	1,366	0,012	0,750	1,800	2,153	1,395	2,109
C342R	Likely pathogenic	Mild	26,20	0,930	0,819	0,987	0,489	0,941	1,806	0,012	1,660	1,800	3,095	2,702	2,788
C342S	Likely pathogenic		23,70	0,844	0,744	0,461	0,330	0,543	0,118	0,012	0,730	1,800	0,881	1,060	1,111
C342W	Likely pathogenic		25,40	0,882	0,799	0,993	0,434	0,864	16,604	0,012	0,010	1,800	3,145	0,453	3,235
C342Y	Likely pathogenic	Severe	27,30	0,951	0,834	0,988	0,440	0,930	8,622	0,012	0,250	1,800	3,290	0,938	3,312
V343A	VUS		21,20	0,426	0,677	0,115	-0,036	0,210	2,427	0,023	0,440	2,700	-2,125	0,511	-1,384
V343E	VUS		21,30	0,576	0,664	0,301	0,090	0,600	0,765	0,023	1,480	2,700	-0,766	2,124	-0,565
V343G	Likely pathogenic		22,20	0,657	0,587	0,288	0,191	0,492	3,363	0,023	0,730	2,700	-0,614	1,235	-0,340
V343L	VUS		21,50	0,597	0,657	0,289	-0,007	0,340	-0,395	0,023	0,110	2,700	-1,511	0,326	-0,827
V343M	VUS		22,10	0,597	0,689	0,181	0,002	0,426	0,273	0,023	0,110	2,700	-1,335	0,354	-0,677
G344A	Likely pathogenic		25,40	0,953	0,745	0,700	0,256	0,894	6,040	0,048	0,290	3,360	1,671	1,699	1,693
G344C	Likely pathogenic		25,40	0,914	0,776	0,954	0,395	0,912	14,167	0,048	0,750	3,360	2,681	2,344	2,464
G344D	Likely pathogenic		25,80	0,937	0,818	0,944	0,315	0,905	27,797	0,048	0,460	3,360	3,176	1,866	3,013

G344R	Likely pathogenic		25,00	0,949	0,810	0,970	0,339	0,918	38,119	0,048	0,910	3,360	3,688	2,337	3,335	
G344S	Likely pathogenic		24,60	0,946	0,736	0,726	0,341	0,900	13,186	0,048	0,020	3,360	2,098	1,337	2,094	
G344V	Likely pathogenic		25,80	0,943	0,753	0,953	0,349	0,916	19,301	0,048	0,730	3,360	2,814	2,269	2,575	
S345A	Likely pathogenic		20,60	0,646	0,584	0,110	-0,084	0,200	-0,412	0,348	0,270	3,680	-2,877	1,581	-2,361	
S345C	Likely pathogenic		24,10	0,673	0,607	0,257	0,063	0,489	-0,721	0,348	0,730	3,680	-1,557	2,488	-1,385	
S345F	Likely pathogenic		22,10	0,581	0,631	0,248	-0,009	0,210	-2,823	0,348	0,850	3,680	-2,492	2,423	-2,129	
S345P	Likely pathogenic		21,90	0,748	0,615	0,474	0,077	0,403	-0,969	0,348	0,250	3,680	-1,447	1,924	-1,158	
S345T	Likely pathogenic		20,20	0,657	0,604	0,194	-0,086	0,214	0,204	0,348	0,050	3,680	-2,716	1,338	-2,152	
S345Y	Likely pathogenic		21,40	0,614	0,653	0,232	0,002	0,330	-3,310	0,348	0,480	3,680	-2,283	2,024	-1,875	
K346E	Likely pathogenic		22,30	0,647	0,558	0,259	-0,086	0,291	1,482	0,339	1,300	3,410	-2,299	2,760	-2,088	
K346M	Likely pathogenic		22,40	0,718	0,520	0,296	-0,105	0,391	0,391	0,339	2,890	3,410	-2,029	4,770	-2,271	
K346N	VUS		21,70	0,537	0,576	0,501	-0,095	0,302	-0,253	0,339	1,730	3,410	-2,285	3,305	-2,128	
K346Q	Likely pathogenic		22,20	0,669	0,524	0,158	-0,113	0,367	-0,398	0,339	1,700	3,410	-2,454	3,241	-2,357	
K346R	Likely pathogenic		21,80	0,662	0,519	0,077	-0,177	0,308	-0,013	0,339	1,140	3,410	-2,865	2,457	-2,563	
K346T	Likely pathogenic		21,80	0,694	0,549	0,235	-0,092	0,377	1,986	0,339	2,120	3,410	-2,096	3,783	-2,132	
F347C	Likely pathogenic		25,40	0,720	0,736	0,614	0,178	0,634	0,659	0,817	0,120	3,210	-0,720	2,743	-0,673	
F347I	VUS		22,30	0,603	0,711	0,258	0,064	0,423	0,490	0,817	0,100	3,210	-2,206	2,329	-1,922	
F347L	VUS		21,70	0,601	0,738	0,765	0,024	0,411	0,178	0,817	0,030	3,210	-1,760	2,360	-1,447	
F347S	VUS		23,00	0,590	0,715	0,505	0,098	0,296	0,419	0,817	0,850	3,210	-1,954	3,308	-1,843	
F347V	VUS		22,30	0,605	0,699	0,275	0,067	0,409	0,674	0,817	0,140	3,210	-2,216	2,374	-1,947	
F347Y	VUS		23,00	0,534	0,727	0,225	0,034	0,484	0,674	0,817	0,370	3,210	-2,242	2,644	-1,998	
W348C	Likely pathogenic		28,10	0,776	0,679	0,930	0,258	0,687	0,906	0,579	0,010	2,630	0,506	2,019	0,552	
W348G	Likely pathogenic		25,40	0,701	0,644	0,541	0,218	0,531	1,292	0,579	0,760	2,630	-0,594	2,620	-0,588	
W348L	VUS		22,30	0,639	0,681	0,595	0,170	0,310	0,211	0,579	0,080	2,630	-1,323	1,609	-0,975	
W348R	Likely pathogenic		25,70	0,770	0,716	0,907	0,228	0,494	0,595	0,579	1,670	2,630	0,139	3,908	-0,088	
W348S	Likely pathogenic		24,00	0,763	0,665	0,684	0,233	0,584	1,150	0,579	0,740	2,630	-0,263	2,664	-0,293	
E349A	Likely pathogenic		29,20	0,728	0,571	0,546	-0,016	0,504	0,144	0,184	1,040	3,050	-0,614	2,360	-0,468	
E349D	Likely pathogenic		21,60	0,542	0,568	0,406	-0,070	0,192	0,212	0,184	0,290	3,050	-2,312	0,961	-1,697	
E349G	Likely pathogenic		24,40	0,643	0,538	0,466	0,010	0,395	1,255	0,184	0,750	3,050	-1,357	1,761	-1,061	
E349K	Likely pathogenic		23,50	0,711	0,642	0,604	0,001	0,519	0,847	0,184	1,300	3,050	-0,706	2,564	-0,565	
E349Q	Likely pathogenic		22,90	0,620	0,553	0,279	-0,054	0,310	0,233	0,184	0,400	3,050	-2,025	1,176	-1,528	
E349V	Likely pathogenic		26,30	0,792	0,557	0,652	-0,003	0,524	0,440	0,184	1,480	3,050	-0,508	2,885	-0,501	
Q350E	VUS		19,71	0,563	0,518	0,072	-0,097	0,275	-0,121	0,484	0,400	2,550	-3,153	1,277	-2,646	
Q350H	VUS		20,40	0,600	0,535	0,204	-0,098	0,276	0,174	0,484	0,470	2,550	-2,830	1,443	-2,363	
Q350K	VUS		20,40	0,566	0,536	0,080	-0,070	0,257	0,358	0,484	1,700	2,550	-2,891	2,902	-2,712	
Q350L	Likely pathogenic		21,20	0,672	0,504	0,185	-0,055	0,345	0,266	0,484	1,190	2,550	-2,471	2,409	-2,277	
Q350P	VUS		19,60	0,536	0,518	0,093	0,068	0,237	-1,174	0,484	0,120	2,550	-2,914	1,012	-2,411	
Q350R	VUS		17,25	0,519	0,528	0,085	-0,079	0,189	-0,221	0,484	0,560	2,550	-3,448	1,372	-2,919	
S351C	Likely pathogenic		24,10	0,753	0,581	0,273	-0,019	0,530	1,946	0,155	0,730	1,590	-0,793	0,865	-0,418	
S351G	VUS		19,83	0,478	0,540	0,083	-0,096	0,251	0,952	0,155	0,020	1,590	-2,616	-0,459	-1,778	
S351I	Likely pathogenic		23,60	0,753	0,598	0,445	0,006	0,623	6,939	0,155	0,750	1,590	-0,190	0,937	0,102	
S351N	VUS		21,70	0,592	0,607	0,150	-0,062	0,401	0,808	0,155	0,340	1,590	-1,712	0,159	-1,061	
S351R	VUS		20,40	0,630	0,611	0,420	-0,004	0,323	2,485	0,155	0,930	1,590	-1,287	0,934	-0,818	
S351T	VUS		21,20	0,628	0,570	0,138	-0,095	0,325	2,728	0,155	0,050	1,590	-1,916	-0,271	-1,182	
V352A	Likely pathogenic		28,70	0,813	0,747	0,823	0,196	0,816	2,119	0,023	0,440	0,800	1,914	0,383	2,204	
V352E	Likely pathogenic		29,90	0,902	0,735	0,960	0,400	0,707	2,921	0,023	1,480	0,800	2,727	1,835	2,615	
V352G	Likely pathogenic		29,70	0,865	0,649	0,859	0,334	0,768	3,133	0,023	0,730	0,800	2,213	0,813	2,286	
V352L	Likely pathogenic	Mild	23,80	0,860	0,732	0,863	0,198	0,865	0,914	0,023	0,110	0,800	1,671	-0,100	2,039	
V352M	Likely pathogenic		24,30	0,887	0,760	0,900	0,285	0,878	-0,144	0,023	0,110	0,800	2,050	0,010	2,361	
R353G	Likely pathogenic	Severe	22,80	0,787	0,487	0,394	0,076	0,772	-0,017	0,299	0,910	0,800	-0,427	1,052	-0,281	
R353L	Likely pathogenic		21,60	0,560	0,516	0,208	0,020	0,432	-1,393	0,299	1,750	0,800	-1,715	1,756	-1,484	
R353P	Likely pathogenic		21,90	0,678	0,524	0,838	0,251	0,681	2,453	0,299	0,680	0,800	0,250	0,881	0,398	
R353Q	Likely pathogenic		21,40	0,669	0,442	0,088	-0,006	0,423	-0,371	0,299	0,560	0,800	-1,954	0,259	-1,485	
R353W	Likely pathogenic		25,60	0,786	0,578	0,203	0,126	0,755	0,481	0,299	1,670	0,800	-0,054	2,059	-0,089	Yes

L354I	Likely pathogenic		22,80	0,776	0,651	0,328	0,148	0,726	3,136	0,090	0,070	-0,360	0,557	-1,034	1,107	
L354P	Likely pathogenic		27,90	0,914	0,729	0,440	0,357	0,958	1,644	0,090	1,070	-0,360	2,349	0,670	2,383	
L354Q	Likely pathogenic		27,60	0,915	0,651	0,658	0,378	0,953	2,598	0,090	1,190	-0,360	2,475	0,841	2,416	
L354R	Likely pathogenic		27,80	0,921	0,707	0,593	0,437	0,943	2,381	0,090	1,750	-0,360	2,722	1,570	2,520	
L354V	Likely pathogenic		22,80	0,870	0,609	0,340	0,189	0,883	2,934	0,090	0,110	-0,360	0,977	-0,874	1,380	
G355A	Likely pathogenic		25,10	0,938	0,618	0,915	0,382	0,930	5,845	0,000	0,290	-0,280	2,696	-0,426	2,850	
G355C	Likely pathogenic		25,00	0,944	0,653	0,988	0,441	0,955	9,163	0,000	0,750	-0,280	3,217	0,187	3,203	
G355D	Likely pathogenic	Severe	25,50	0,954	0,703	0,985	0,442	0,956	18,257	0,000	0,460	-0,280	3,741	-0,204	3,761	
G355R	Likely pathogenic		24,70	0,958	0,694	0,993	0,458	0,955	27,833	0,000	0,910	-0,280	4,143	0,253	3,990	
G355S	Likely pathogenic		24,40	0,930	0,607	0,940	0,387	0,946	10,392	0,000	0,020	-0,280	2,839	-0,805	3,026	
G355V	Likely pathogenic		25,50	0,954	0,626	0,992	0,468	0,952	15,117	0,000	0,730	-0,280	3,515	0,136	3,438	
S356A	Likely pathogenic		23,90	0,826	0,555	0,184	0,015	0,574	-0,119	0,032	0,270	-0,310	-0,252	-1,001	0,363	
S356C	Likely pathogenic		23,70	0,898	0,572	0,186	0,046	0,539	-0,591	0,032	0,730	-0,310	-0,050	-0,392	0,430	
S356F	Pathogenic		26,00	0,921	0,605	0,580	0,104	0,895	1,071	0,032	0,850	-0,310	1,417	0,097	1,653	
S356P	Likely pathogenic		24,60	0,938	0,585	0,951	0,248	0,843	3,357	0,032	0,250	-0,310	2,029	-0,526	2,298	
S356T	Likely pathogenic		23,70	0,842	0,573	0,354	0,085	0,666	0,080	0,032	0,050	-0,310	0,292	-1,143	0,874	
S356Y	Likely pathogenic		25,30	0,896	0,623	0,451	0,099	0,852	0,574	0,032	0,480	-0,310	1,095	-0,431	1,474	
W357C	Likely pathogenic		27,40	0,927	0,772	0,998	0,510	0,953	3,638	0,000	0,010	-0,520	3,573	-0,665	3,785	
W357G	Likely pathogenic		29,10	0,899	0,743	0,970	0,560	0,948	4,607	0,000	0,760	-0,520	3,747	0,284	3,729	
W357L	Likely pathogenic		29,20	0,867	0,777	0,974	0,522	0,906	1,103	0,000	0,080	-0,520	3,431	-0,552	3,663	
W357R	Likely pathogenic		28,10	0,901	0,800	0,997	0,558	0,956	5,063	0,000	1,670	-0,520	3,941	1,398	3,727	
W357S	Likely pathogenic		31,00	0,871	0,762	0,980	0,573	0,934	5,607	0,000	0,740	-0,520	3,933	0,303	3,916	
D358A	VUS		22,60	0,604	0,463	0,131	-0,005	0,593	2,083	0,373	0,750	0,430	-1,550	0,489	-1,182	
D358E	VUS		14,83	0,474	0,470	0,097	-0,079	0,295	-0,740	0,373	0,290	0,430	-3,139	-0,481	-2,372	
D358G	Likely pathogenic		23,60	0,675	0,459	0,117	0,006	0,565	2,756	0,373	0,460	0,430	-1,378	0,183	-0,976	
D358H	Likely pathogenic		22,90	0,673	0,510	0,170	0,007	0,670	2,493	0,373	0,580	0,430	-1,095	0,383	-0,735	
D358N	VUS		22,10	0,480	0,492	0,099	-0,042	0,469	2,035	0,373	0,140	0,430	-2,110	-0,392	-1,458	
D358V	Likely pathogenic		23,50	0,666	0,451	0,194	0,049	0,753	2,228	0,373	1,190	0,430	-0,922	1,179	-0,794	
D358Y	Likely pathogenic		22,00	0,666	0,506	0,181	0,037	0,780	1,678	0,373	0,960	0,430	-0,940	0,882	-0,719	
R359G	Likely pathogenic		26,50	0,947	0,601	0,924	0,412	0,954	2,955	0,015	0,910	0,850	2,575	1,104	2,456	
R359L	Likely pathogenic		24,70	0,883	0,625	0,930	0,304	0,948	-0,230	0,015	1,750	0,850	2,051	2,025	1,864	
R359P	Likely pathogenic		25,00	0,916	0,635	0,990	0,519	0,900	1,707	0,015	0,680	0,850	2,669	0,849	2,601	
R359Q	Likely pathogenic	Mild	24,80	0,929	0,553	0,603	0,352	0,952	1,291	0,015	0,560	0,850	1,707	0,487	1,742	Yes
G360A	Likely pathogenic		23,50	0,611	0,617	0,361	0,176	0,418	3,384	0,000	0,290	0,020	-0,071	-0,887	0,567	
G360E	Likely pathogenic		27,20	0,863	0,680	0,981	0,342	0,929	29,161	0,000	0,750	0,020	3,757	0,161	3,707	
G360R	Likely pathogenic		24,70	0,898	0,695	0,985	0,359	0,933	34,248	0,000	0,910	0,020	3,955	0,280	3,839	
G360V	Likely pathogenic		27,00	0,910	0,623	0,964	0,369	0,872	17,003	0,000	0,730	0,020	3,159	0,222	3,146	
G360W	Likely pathogenic		25,40	0,897	0,678	0,993	0,380	0,892	69,365	0,000	0,760	0,020	5,393	-0,182	5,107	
M361I	VUS		19,71	0,396	0,342	0,164	0,002	0,765	1,538	0,067	0,070	0,080	-1,712	-1,326	-1,053	
M361K	Likely pathogenic		14,35	0,485	0,379	0,160	0,138	0,506	2,090	0,067	2,890	0,080	-1,664	2,013	-1,659	
M361L	Likely pathogenic		13,53	0,402	0,341	0,119	-0,028	0,537	-0,438	0,067	0,000	0,080	-2,687	-1,672	-1,849	
M361R	Likely pathogenic		15,56	0,527	0,371	0,125	0,170	0,583	3,060	0,067	1,750	0,080	-1,410	0,696	-1,207	Yes
M361T	Likely pathogenic		14,11	0,312	0,395	0,102	0,045	0,224	1,322	0,067	0,770	0,080	-2,869	-0,824	-2,103	
M361V	Likely pathogenic		12,18	0,285	0,318	0,063	0,017	0,263	1,823	0,067	0,110	0,080	-3,318	-1,746	-2,397	
Q362E	VUS		21,30	0,427	0,441	0,083	-0,087	0,273	-0,088	0,262	0,400	0,760	-2,788	-0,258	-2,065	
Q362H	VUS		19,93	0,599	0,466	0,216	-0,087	0,682	0,361	0,262	0,470	0,760	-1,716	0,059	-1,216	
Q362K	VUS		21,70	0,456	0,475	0,095	-0,065	0,256	-0,418	0,262	1,700	0,760	-2,501	1,383	-2,100	
Q362L	VUS		22,30	0,608	0,435	0,125	-0,065	0,699	-1,053	0,262	1,190	0,760	-1,632	1,001	-1,348	
Q362P	Likely pathogenic		22,40	0,750	0,454	0,519	0,173	0,812	2,073	0,262	0,120	0,760	-0,088	0,031	0,175	
Q362R	VUS		19,59	0,471	0,470	0,091	-0,067	0,207	-0,287	0,262	0,560	0,760	-2,792	-0,084	-2,086	
Y363C	Likely pathogenic		32,00	0,965	0,722	0,940	0,405	0,947	5,559	0,011	0,250	0,180	3,469	0,091	3,555	
Y363D	Likely pathogenic		31,00	0,964	0,719	0,972	0,448	0,949	7,039	0,011	0,960	0,180	3,645	0,951	3,527	
Y363F	Likely pathogenic		27,70	0,917	0,654	0,599	0,199	0,823	0,996	0,011	0,370	0,180	1,678	-0,150	1,979	
Y363H	Likely pathogenic		29,90	0,980	0,728	0,979	0,342	0,953	4,916	0,011	0,380	0,180	3,247	0,185	3,352	

Y363N	Likely pathogenic		31,00	0,944	0,723	0,895	0,427	0,954	5,743	0,011	0,820	0,180	3,424	0,750	3,373	
Y363S	Likely pathogenic		29,70	0,957	0,672	0,950	0,439	0,938	6,660	0,011	0,480	0,180	3,313	0,293	3,318	
S364C	Likely pathogenic		27,80	0,801	0,592	0,739	0,151	0,820	2,898	0,000	0,730	-0,170	1,543	-0,018	1,814	
S364G	Likely pathogenic		26,40	0,730	0,554	0,364	0,034	0,741	1,340	0,000	0,020	-0,170	0,290	-1,162	0,891	
S364I	Likely pathogenic		25,40	0,776	0,603	0,975	0,086	0,848	5,716	0,000	0,750	-0,170	1,618	-0,050	1,915	
S364N	Likely pathogenic	Mild	27,40	0,667	0,626	0,974	0,123	0,934	6,407	0,000	0,340	-0,170	1,818	-0,496	2,194	Yes
S364R	Likely pathogenic	Severe	27,60	0,854	0,631	0,999	0,195	0,931	26,101	0,000	0,930	-0,170	3,249	0,208	3,255	
S364T	Likely pathogenic	Mild	23,10	0,660	0,593	0,414	0,006	0,786	2,394	0,000	0,050	-0,170	0,116	-1,231	0,772	
H365D	Likely pathogenic		23,70	0,647	0,550	0,208	0,008	0,638	1,101	0,268	0,580	-0,020	-0,820	-0,054	-0,349	
H365L	VUS		23,50	0,627	0,521	0,226	0,001	0,656	-1,450	0,268	0,720	-0,020	-1,006	0,118	-0,563	
H365N	VUS		22,60	0,470	0,552	0,132	-0,039	0,257	0,377	0,268	0,440	-0,020	-2,036	-0,515	-1,276	
H365P	Likely pathogenic		23,90	0,769	0,501	0,737	0,208	0,752	6,962	0,268	0,350	-0,020	0,766	-0,036	0,987	
H365Q	VUS		22,00	0,549	0,526	0,154	-0,074	0,308	-0,145	0,268	0,470	-0,020	-1,992	-0,458	-1,272	
H365R	Likely pathogenic		22,40	0,701	0,553	0,067	-0,011	0,605	-0,610	0,268	1,030	-0,020	-1,090	0,439	-0,695	
H365Y	VUS		22,50	0,584	0,555	0,116	-0,071	0,254	-1,125	0,268	0,380	-0,020	-1,983	-0,544	-1,221	
S366C	Likely pathogenic		28,30	0,793	0,583	0,333	0,187	0,654	0,074	0,013	0,730	-0,300	0,826	-0,191	1,179	
S366G	Likely pathogenic	Mild	25,10	0,770	0,540	0,218	0,146	0,706	0,605	0,013	0,020	-0,300	0,274	-1,220	0,826	
S366I	Likely pathogenic		27,30	0,754	0,590	0,897	0,218	0,847	1,551	0,013	0,750	-0,300	1,743	0,022	1,980	
S366N	VUS		22,60	0,543	0,614	0,263	0,065	0,637	3,821	0,013	0,340	-0,300	-0,250	-1,062	0,419	
S366R	Likely pathogenic		28,00	0,849	0,616	0,969	0,155	0,925	6,927	0,013	0,930	-0,300	2,314	0,288	2,452	
S366T	Likely pathogenic		25,80	0,749	0,584	0,406	0,128	0,838	0,187	0,013	0,050	-0,300	0,734	-1,063	1,254	
I367F	Likely pathogenic		24,80	0,914	0,644	0,851	0,359	0,909	11,897	0,005	0,100	-0,570	2,821	-0,913	3,056	
I367L	Likely pathogenic		24,50	0,853	0,643	0,308	0,199	0,663	0,403	0,005	0,070	-0,570	0,859	-1,222	1,417	
I367M	Likely pathogenic		25,10	0,862	0,610	0,271	0,211	0,820	0,006	0,005	0,070	-0,570	1,044	-1,162	1,520	
I367N	Likely pathogenic		27,70	0,948	0,721	0,984	0,435	0,950	3,414	0,005	1,090	-0,570	3,389	0,591	3,356	
I367S	Likely pathogenic		27,90	0,952	0,663	0,970	0,439	0,937	3,707	0,005	0,750	-0,570	3,232	0,150	3,256	
I367T	Likely pathogenic		26,50	0,957	0,738	0,962	0,356	0,935	3,443	0,005	0,700	-0,570	3,094	0,036	3,226	
I367V	Likely pathogenic		23,60	0,777	0,561	0,143	0,164	0,406	0,727	0,005	0,040	-0,570	-0,184	-1,512	0,507	
I368F	Likely pathogenic		26,00	0,892	0,649	0,413	0,180	0,800	2,863	0,005	0,100	-0,750	1,464	-1,180	1,945	
I368L	Likely pathogenic		23,00	0,666	0,642	0,111	0,082	0,353	-0,457	0,005	0,070	-0,750	-0,566	-1,681	0,293	
I368M	Likely pathogenic		22,60	0,809	0,613	0,115	0,106	0,490	-0,121	0,005	0,070	-0,750	-0,112	-1,575	0,616	
I368N	Likely pathogenic		24,70	0,932	0,708	0,866	0,339	0,918	3,346	0,005	1,090	-0,750	2,740	0,292	2,824	
I368S	Likely pathogenic		24,80	0,945	0,660	0,803	0,343	0,874	3,361	0,005	0,750	-0,750	2,506	-0,165	2,665	
I368T	Likely pathogenic		24,10	0,913	0,721	0,675	0,180	0,818	2,167	0,005	0,700	-0,750	1,873	-0,373	2,225	
I368V	Likely pathogenic		23,50	0,755	0,572	0,174	0,072	0,437	0,813	0,005	0,040	-0,750	-0,310	-1,658	0,452	
T369A	VUS		19,08	0,525	0,478	0,089	0,009	0,412	-0,077	0,157	0,220	-0,210	-1,901	-1,150	-1,138	
T369K	VUS		21,40	0,652	0,556	0,171	0,093	0,570	-0,947	0,157	2,120	-0,210	-0,677	1,463	-0,520	
T369M	Benign	Risk variant	21,80	0,731	0,516	0,133	0,051	0,566	-1,498	0,157	0,770	-0,210	-0,866	-0,185	-0,398	Yes
T369P	Likely pathogenic		22,30	0,747	0,486	0,639	0,190	0,735	3,840	0,157	0,300	-0,210	0,456	-0,513	0,801	
T369R	VUS		21,90	0,695	0,546	0,123	0,111	0,740	-0,456	0,157	0,980	-0,210	-0,406	0,150	-0,069	
T369S	VUS		18,19	0,431	0,479	0,102	-0,029	0,345	0,602	0,157	0,050	-0,210	-2,299	-1,474	-1,408	
N370D	Likely pathogenic		20,50	0,452	0,543	0,176	0,079	0,428	-1,143	0,041	0,140	-0,230	-1,383	-1,388	-0,559	
N370H	Likely pathogenic		24,40	0,792	0,516	0,558	0,189	0,841	-0,951	0,041	0,440	-0,230	0,804	-0,446	1,135	
N370I	Likely pathogenic		28,60	0,929	0,517	0,847	0,234	0,891	0,300	0,041	1,090	-0,230	1,967	0,650	1,981	
N370K	Likely pathogenic		24,00	0,733	0,569	0,969	0,187	0,863	-0,804	0,041	1,730	-0,230	1,374	1,237	1,404	
N370S	Pathogenic	Mild	23,90	0,673	0,464	0,148	0,154	0,816	0,206	0,041	0,340	-0,230	-0,115	-0,817	0,321	Yes
N370T	Likely pathogenic		24,60	0,739	0,504	0,398	0,124	0,798	-0,066	0,041	0,390	-0,230	0,324	-0,637	0,742	
N370Y	Likely pathogenic		24,80	0,805	0,541	0,773	0,137	0,859	-2,107	0,041	0,820	-0,230	1,039	0,096	1,296	
L371F	Likely pathogenic		27,50	0,899	0,566	0,303	0,200	0,898	-1,096	0,025	0,030	-0,430	1,200	-0,983	1,598	
L371H	Likely pathogenic		30,00	0,971	0,641	0,903	0,314	0,945	2,466	0,025	0,720	-0,430	2,897	0,219	2,963	
L371I	Likely pathogenic		26,50	0,830	0,578	0,213	0,123	0,623	0,489	0,025	0,070	-0,430	0,402	-1,167	0,976	
L371P	Likely pathogenic		32,00	0,972	0,680	0,988	0,355	0,953	5,807	0,025	1,070	-0,430	3,499	0,741	3,428	
L371R	Likely pathogenic		31,00	0,974	0,641	0,944	0,420	0,940	3,401	0,025	1,750	-0,430	3,378	1,568	3,112	
L371V	Likely pathogenic	Mild	26,30	0,924	0,532	0,276	0,167	0,885	1,706	0,025	0,110	-0,430	1,073	-0,974	1,448	

L372M	VUS		23,00	0,529	0,499	0,122	0,145	0,552	0,238	0,368	0,000	-0,390	-1,253	-0,842	-0,666	
L372P	Likely pathogenic		25,20	0,720	0,576	0,745	0,331	0,903	4,737	0,368	1,070	-0,390	1,370	1,018	1,327	
L372Q	VUS		23,20	0,622	0,499	0,069	0,180	0,494	1,193	0,368	1,190	-0,390	-0,994	0,641	-0,736	
L372R	VUS		23,30	0,603	0,568	0,091	0,241	0,637	-0,379	0,368	1,750	-0,390	-0,498	1,448	-0,423	
L372V	VUS		21,70	0,487	0,475	0,114	0,158	0,481	1,473	0,368	0,110	-0,390	-1,507	-0,804	-0,919	
Y373C	Likely pathogenic		26,00	0,840	0,547	0,169	0,276	0,761	2,055	0,285	0,250	0,460	0,377	0,184	0,579	
Y373D	Likely pathogenic		23,30	0,738	0,553	0,316	0,246	0,749	2,302	0,285	0,960	0,460	0,146	0,949	0,250	
Y373F	Likely pathogenic		23,00	0,668	0,496	0,194	0,143	0,503	-0,314	0,285	0,370	0,460	-1,046	0,007	-0,618	
Y373H	VUS		20,90	0,387	0,549	0,102	0,104	0,231	0,430	0,285	0,380	0,460	-2,170	-0,304	-1,470	
Y373N	VUS		21,30	0,439	0,553	0,131	0,194	0,297	0,796	0,285	0,820	0,460	-1,641	0,350	-1,153	
Y373S	VUS		22,50	0,588	0,503	0,199	0,232	0,547	1,853	0,285	0,480	0,460	-0,834	0,138	-0,477	
H374D	Likely pathogenic		24,40	0,772	0,521	0,286	0,064	0,822	-0,316	0,165	0,580	0,170	-0,062	0,044	0,267	
H374L	Likely pathogenic		23,10	0,754	0,494	0,598	0,143	0,712	-1,666	0,165	0,720	0,170	0,071	0,266	0,349	
H374N	Likely pathogenic		24,00	0,706	0,522	0,169	0,011	0,754	-0,256	0,165	0,440	0,170	-0,580	-0,258	-0,122	
H374P	Likely pathogenic		26,40	0,801	0,477	0,599	0,256	0,850	2,711	0,165	0,350	0,170	0,984	-0,005	1,156	
H374Q	Likely pathogenic		21,10	0,673	0,499	0,436	0,061	0,727	0,471	0,165	0,470	0,170	-0,496	-0,238	-0,063	
H374R	Likely pathogenic		25,40	0,766	0,521	0,269	0,061	0,813	-0,108	0,165	1,030	0,170	-0,001	0,607	0,218	
H374Y	Likely pathogenic		20,20	0,508	0,522	0,115	-0,038	0,238	-2,195	0,165	0,380	0,170	-2,256	-0,738	-1,443	
V375A	Likely pathogenic		23,40	0,825	0,662	0,264	0,141	0,887	2,269	0,006	0,440	-0,830	1,072	-0,940	1,533	
V375E	Likely pathogenic		25,30	0,941	0,647	0,861	0,356	0,935	4,385	0,006	1,480	-0,830	2,803	0,726	2,741	
V375G	Likely pathogenic	Mild	24,90	0,942	0,560	0,499	0,277	0,731	3,805	0,006	0,730	-0,830	1,595	-0,450	1,832	
V375L	Pathogenic	Mild	24,20	0,871	0,645	0,731	0,155	0,925	1,571	0,006	0,110	-0,830	1,727	-1,144	2,186	
V375M	Likely pathogenic		24,70	0,842	0,675	0,396	0,159	0,927	0,893	0,006	0,110	-0,830	1,395	-1,220	1,899	
V376A	VUS		12,15	0,425	0,395	0,050	0,035	0,156	1,487	0,098	0,440	-0,910	-2,838	-1,781	-1,927	
V376D	Likely pathogenic		19,64	0,703	0,413	0,277	0,301	0,691	3,013	0,098	1,190	-0,910	0,066	-0,128	0,252	
V376F	Likely pathogenic		18,92	0,679	0,385	0,339	0,211	0,773	2,307	0,098	0,140	-0,910	-0,228	-1,452	0,241	
V376G	Likely pathogenic		14,92	0,681	0,339	0,139	0,175	0,434	2,412	0,098	0,730	-0,910	-1,378	-1,054	-0,881	
V376I	VUS		9,15	0,359	0,337	0,113	-0,019	0,357	-0,222	0,098	0,040	-0,910	-3,170	-2,337	-2,172	
V376L	VUS		14,29	0,491	0,388	0,294	0,062	0,510	-0,025	0,098	0,110	-0,910	-1,802	-1,875	-1,010	
G377A	Likely pathogenic		24,10	0,860	0,580	0,460	0,289	0,676	4,042	0,019	0,290	-0,360	1,200	-0,768	1,566	
G377C	Likely pathogenic	Mild	21,30	0,960	0,625	0,869	0,428	0,911	9,225	0,019	0,750	-0,360	2,684	0,016	2,712	
G377D	Likely pathogenic		24,50	0,971	0,678	0,989	0,429	0,918	17,162	0,019	0,460	-0,360	3,505	-0,251	3,542	
G377R	Likely pathogenic		20,90	0,956	0,669	0,985	0,445	0,900	27,079	0,019	0,910	-0,360	3,649	0,107	3,548	
G377S	Likely pathogenic	Severe	20,30	0,957	0,578	0,591	0,299	0,786	6,967	0,019	0,020	-0,360	1,544	-1,101	1,896	Yes
G377V	Likely pathogenic		24,40	0,977	0,588	0,954	0,455	0,909	16,789	0,019	0,730	-0,360	3,312	0,048	3,234	
W378C	Likely pathogenic		25,10	0,967	0,733	0,990	0,506	0,949	4,034	0,018	0,010	0,900	3,079	0,123	3,168	
W378G	Pathogenic	Severe	24,20	0,950	0,705	0,965	0,556	0,946	6,258	0,018	0,760	0,900	3,152	0,996	3,020	
W378L	Likely pathogenic		24,40	0,932	0,741	0,970	0,450	0,925	1,414	0,018	0,080	0,900	2,688	0,152	2,842	
W378R	Likely pathogenic		23,80	0,946	0,771	0,998	0,554	0,949	3,741	0,018	1,670	0,900	3,275	2,153	2,966	
W378S	Likely pathogenic		24,80	0,951	0,727	0,979	0,568	0,946	5,343	0,018	0,740	0,900	3,255	1,016	3,128	
T379A	Likely pathogenic		23,50	0,776	0,502	0,203	0,161	0,746	2,082	0,000	0,220	-0,150	0,224	-0,966	0,685	
T379I	Likely pathogenic		19,64	0,651	0,617	0,197	0,086	0,544	-0,561	0,000	0,700	-0,150	-0,571	-0,592	0,037	
T379N	Likely pathogenic		23,00	0,849	0,565	0,580	0,149	0,891	1,164	0,000	0,390	-0,150	1,058	-0,551	1,413	
T379P	Likely pathogenic		24,20	0,925	0,502	0,840	0,325	0,902	4,871	0,000	0,300	-0,150	2,010	-0,470	2,170	
T379S	Likely pathogenic		23,50	0,881	0,512	0,359	0,125	0,707	2,131	0,000	0,050	-0,150	0,467	-1,107	0,958	
D380A	Likely pathogenic	Severe	25,00	0,968	0,603	0,982	0,267	0,939	1,517	0,005	0,750	-0,780	2,474	-0,140	2,620	
D380E	Likely pathogenic		23,30	0,903	0,632	0,946	0,200	0,869	2,625	0,005	0,290	-0,780	2,004	-0,851	2,377	
D380G	Likely pathogenic		25,00	0,969	0,616	0,981	0,346	0,932	2,914	0,005	0,460	-0,780	2,719	-0,460	2,888	
D380H	Likely pathogenic		23,10	0,973	0,673	0,993	0,344	0,935	27,299	0,005	0,580	-0,780	3,753	-0,539	3,794	
D380N	Pathogenic		23,10	0,942	0,652	0,970	0,301	0,939	1,341	0,005	0,140	-0,780	2,423	-0,915	2,745	
D380V	Likely pathogenic		24,80	0,967	0,603	0,991	0,382	0,931	2,316	0,005	1,190	-0,780	2,795	0,448	2,764	
D380Y	Likely pathogenic		23,10	0,972	0,660	0,989	0,380	0,940	14,415	0,005	0,960	-0,780	3,308	0,046	3,299	
W381C	Likely pathogenic		25,30	0,971	0,754	0,992	0,507	0,951	0,629	0,025	0,010	-1,200	3,426	-1,031	3,690	
W381G	Likely pathogenic		26,10	0,926	0,726	0,953	0,556	0,947	0,886	0,025	0,760	-1,200	3,467	-0,113	3,519	

W381L	Likely pathogenic		24,60	0,940	0,756	0,985	0,518	0,949	-0,805	0,025	0,080	-1,200	3,288	-0,964	3,555	
W381R	Likely pathogenic		25,40	0,892	0,778	0,997	0,555	0,939	-0,161	0,025	1,670	-1,200	3,535	1,002	3,414	
W381S	Likely pathogenic		25,00	0,951	0,747	0,962	0,496	0,941	1,390	0,025	0,740	-1,200	3,363	-0,184	3,464	
N382D	Likely pathogenic		24,40	0,906	0,661	0,980	0,254	0,907	1,589	0,005	0,140	-1,270	2,426	-1,213	2,829	
N382H	Likely pathogenic		24,10	0,946	0,630	0,968	0,324	0,930	30,049	0,005	0,440	-1,270	3,787	-1,040	3,882	
N382I	Likely pathogenic		24,70	0,960	0,629	0,969	0,369	0,927	1,430	0,005	1,090	-1,270	2,831	0,039	2,889	
N382K	.	Severe	23,90	0,916	0,687	0,997	0,322	0,923	10,514	0,005	1,730	-1,270	3,162	0,707	3,091	
N382S	Likely pathogenic		23,80	0,939	0,575	0,828	0,216	0,903	1,756	0,005	0,340	-1,270	1,997	-1,071	2,341	
N382T	Likely pathogenic		24,20	0,955	0,611	0,958	0,338	0,894	1,450	0,005	0,390	-1,270	2,557	-0,872	2,816	
N382Y	Likely pathogenic		24,60	0,968	0,657	0,977	0,360	0,933	28,849	0,005	0,820	-1,270	4,007	-0,506	3,992	
L383F	Likely pathogenic		24,10	0,772	0,497	0,367	0,172	0,680	7,101	0,005	0,030	-1,290	0,770	-1,845	1,330	
L383H	Likely pathogenic		26,10	0,954	0,572	0,965	0,361	0,926	3,811	0,005	0,720	-1,290	2,832	-0,437	2,942	
L383I	Likely pathogenic		22,70	0,691	0,509	0,110	0,096	0,550	2,015	0,005	0,070	-1,290	-0,320	-1,989	0,420	
L383P	Likely pathogenic		26,50	0,923	0,613	0,963	0,326	0,942	6,745	0,005	1,070	-1,290	2,981	-0,036	3,031	
L383R	Likely pathogenic		26,40	0,977	0,570	0,966	0,398	0,946	6,525	0,005	1,750	-1,290	3,202	0,846	3,003	
L383V	Likely pathogenic		23,70	0,769	0,455	0,167	0,156	0,795	3,371	0,005	0,110	-1,290	0,400	-1,774	0,931	
A384D	Likely pathogenic		26,50	0,942	0,743	0,989	0,309	0,949	6,946	0,000	0,750	-1,550	3,368	-0,527	3,571	
A384G	Likely pathogenic		26,30	0,913	0,653	0,487	0,153	0,841	1,548	0,000	0,290	-1,550	1,738	-1,369	2,228	
A384P	Likely pathogenic		22,10	0,906	0,713	0,946	0,281	0,907	3,596	0,000	0,520	-1,550	2,585	-0,976	2,934	
A384S	Likely pathogenic		21,70	0,775	0,640	0,291	0,114	0,739	0,248	0,000	0,270	-1,550	0,605	-1,697	1,266	
A384T	Likely pathogenic		22,00	0,796	0,673	0,329	0,130	0,895	0,259	0,000	0,220	-1,550	1,053	-1,654	1,661	
A384V	Likely pathogenic		24,00	0,747	0,682	0,123	0,099	0,817	1,202	0,000	0,440	-1,550	0,762	-1,460	1,380	
L385M	Likely pathogenic		21,80	0,861	0,594	0,839	0,307	0,777	1,646	0,000	0,000	-1,320	1,783	-1,601	2,259	
L385P	Likely pathogenic	Severe	24,90	0,975	0,707	0,996	0,425	0,954	7,283	0,000	1,070	-1,320	3,518	0,019	3,535	
L385Q	Likely pathogenic		24,70	0,979	0,621	0,977	0,430	0,946	5,158	0,000	1,190	-1,320	3,209	0,140	3,172	
L385R	Likely pathogenic		24,90	0,977	0,681	0,965	0,422	0,947	11,266	0,000	1,750	-1,320	3,622	0,795	3,446	
L385V	Likely pathogenic		21,50	0,934	0,559	0,596	0,247	0,798	1,987	0,000	0,110	-1,320	1,458	-1,553	1,911	
N386D	VUS		18,14	0,540	0,602	0,095	0,123	0,387	0,407	0,169	0,140	-0,910	-1,264	-1,544	-0,445	
N386H	Likely pathogenic		23,60	0,840	0,573	0,414	0,167	0,829	1,588	0,169	0,440	-0,910	0,811	-0,631	1,168	
N386I	Likely pathogenic		24,50	0,958	0,567	0,854	0,281	0,867	1,258	0,169	1,090	-0,910	1,933	0,450	1,964	
N386K	Likely pathogenic	Severe	22,60	0,912	0,621	0,905	0,234	0,902	1,029	0,169	1,730	-0,910	1,874	1,181	1,819	
N386S	Likely pathogenic		23,50	0,854	0,519	0,130	0,122	0,750	0,871	0,169	0,340	-0,910	0,124	-0,899	0,558	
N386T	Likely pathogenic		24,10	0,934	0,559	0,344	0,250	0,808	1,955	0,169	0,390	-0,910	1,093	-0,622	1,375	
N386Y	Likely pathogenic		24,00	0,923	0,598	0,666	0,271	0,872	1,389	0,169	0,820	-0,910	1,669	0,042	1,813	
P387A	Likely pathogenic		21,70	0,415	0,328	0,067	-0,024	0,278	2,029	0,371	0,520	-0,840	-2,641	-0,840	-1,970	
P387H	Likely pathogenic		15,04	0,368	0,359	0,132	0,007	0,230	2,005	0,371	0,350	-0,840	-3,068	-1,224	-2,278	
P387L	VUS		12,88	0,562	0,366	0,060	-0,007	0,627	0,290	0,371	1,070	-0,840	-2,372	-0,184	-1,934	
P387R	Likely pathogenic		15,07	0,498	0,378	0,087	0,038	0,246	1,110	0,371	0,680	-0,840	-2,735	-0,731	-2,085	
P387S	Likely pathogenic		22,20	0,470	0,318	0,107	-0,029	0,268	2,133	0,371	0,250	-0,840	-2,527	-1,129	-1,817	
P387T	Likely pathogenic		21,80	0,476	0,334	0,072	-0,033	0,281	1,890	0,371	0,300	-0,840	-2,540	-1,075	-1,833	
E388A	Likely pathogenic		22,50	0,649	0,423	0,126	-0,035	0,539	-0,160	0,426	1,040	0,130	-1,808	0,764	-1,493	
E388D	VUS		18,39	0,508	0,421	0,147	-0,079	0,321	-0,018	0,426	0,290	0,130	-2,826	-0,432	-2,138	
E388G	VUS		21,90	0,599	0,388	0,192	-0,019	0,383	0,343	0,426	0,750	0,130	-2,150	0,324	-1,714	
E388K	VUS	Risk variant	16,99	0,579	0,500	0,097	-0,030	0,492	-0,627	0,426	1,300	0,130	-2,236	0,907	-1,860	Yes
E388Q	VUS		15,15	0,516	0,403	0,091	-0,085	0,260	-0,867	0,426	0,400	0,130	-3,276	-0,424	-2,566	
E388V	Likely pathogenic		22,50	0,647	0,405	0,190	-0,023	0,590	0,163	0,426	1,480	0,130	-1,634	1,333	-1,464	
G389A	Likely pathogenic		24,30	0,945	0,557	0,919	0,372	0,940	8,504	0,000	0,290	-0,590	2,678	-0,671	2,819	
G389E	Likely pathogenic	Severe	24,70	0,966	0,628	0,989	0,432	0,950	30,319	0,000	0,750	-0,590	4,079	-0,182	3,961	
G389R	Likely pathogenic		24,00	0,944	0,642	0,990	0,376	0,951	30,483	0,000	0,910	-0,590	3,908	-0,043	3,805	
G389V	Likely pathogenic		24,60	0,961	0,567	0,990	0,391	0,951	24,070	0,000	0,730	-0,590	3,565	-0,207	3,487	
G390A	Likely pathogenic		24,70	0,956	0,679	0,649	0,272	0,936	4,042	0,000	0,290	-0,700	2,310	-0,766	2,612	
G390E	Likely pathogenic		25,20	0,966	0,739	0,967	0,350	0,957	4,453	0,000	0,750	-0,700	3,127	-0,022	3,254	
G390R	Likely pathogenic	Severe	24,20	0,968	0,748	0,955	0,367	0,956	4,937	0,000	0,910	-0,700	3,141	0,152	3,229	
G390V	Likely pathogenic		25,10	0,961	0,686	0,939	0,377	0,952	7,363	0,000	0,730	-0,700	3,129	-0,092	3,212	

P391A	Likely pathogenic		24,40	0,946	0,613	0,864	0,325	0,916	3,803	0,208	0,520	-0,740	2,180	-0,037	2,288
P391H	Likely pathogenic		27,70	0,973	0,658	0,933	0,364	0,940	4,235	0,208	0,350	-0,740	2,779	-0,076	2,872
P391L	Pathogenic		28,10	0,970	0,671	0,856	0,374	0,949	3,679	0,208	1,070	-0,740	2,815	0,810	2,736
P391R	Likely pathogenic		27,70	0,985	0,675	0,870	0,395	0,945	3,405	0,208	0,680	-0,740	2,846	0,348	2,849
P391S	Likely pathogenic		25,00	0,960	0,625	0,934	0,325	0,927	4,101	0,208	0,250	-0,740	2,361	-0,318	2,520
P391T	Likely pathogenic		24,70	0,978	0,652	0,895	0,337	0,947	4,686	0,208	0,300	-0,740	2,483	-0,248	2,620
N392D	Likely pathogenic		26,40	0,931	0,620	0,886	0,264	0,947	3,177	0,046	0,140	-0,830	2,418	-0,844	2,708
N392H	Likely pathogenic		26,00	0,953	0,588	0,816	0,193	0,944	26,722	0,046	0,440	-0,830	3,095	-0,745	3,211
N392I	Likely pathogenic	Severe	31,00	0,922	0,583	0,926	0,311	0,946	2,646	0,046	1,090	-0,830	2,840	0,467	2,821
N392K	Likely pathogenic		23,40	0,853	0,641	0,993	0,264	0,936	11,599	0,046	1,730	-0,830	2,675	0,944	2,591
N392S	Likely pathogenic		27,40	0,801	0,528	0,141	0,141	0,880	-0,341	0,046	0,340	-0,830	0,673	-0,950	1,090
N392T	Likely pathogenic		28,60	0,866	0,567	0,366	0,187	0,916	0,651	0,046	0,390	-0,830	1,431	-0,713	1,755
N392Y	Likely pathogenic		26,80	0,967	0,610	0,935	0,302	0,946	23,780	0,046	0,820	-0,830	3,530	-0,124	3,492
W393C	Likely pathogenic		28,00	0,949	0,759	0,963	0,454	0,927	2,396	0,312	0,010	-0,960	3,025	-0,298	3,184
W393G	Likely pathogenic		29,50	0,956	0,732	0,835	0,436	0,935	2,535	0,312	0,760	-0,960	2,966	0,607	2,933
W393L	Likely pathogenic		26,30	0,897	0,763	0,878	0,386	0,896	1,088	0,312	0,080	-0,960	2,465	-0,346	2,709
W393R	Likely pathogenic	Mild	28,10	0,947	0,798	0,961	0,434	0,946	1,718	0,312	1,670	-0,960	3,193	1,749	2,975
W393S	Likely pathogenic		27,40	0,937	0,754	0,874	0,516	0,927	2,764	0,312	0,740	-0,960	3,067	0,575	3,022
V394A	Likely pathogenic		23,40	0,737	0,735	0,253	0,087	0,515	1,715	0,253	0,440	0,100	-0,164	-0,066	0,375
V394E	Likely pathogenic		29,30	0,948	0,723	0,683	0,245	0,912	1,939	0,253	1,480	0,100	2,155	1,814	2,034
V394G	Likely pathogenic		29,00	0,946	0,636	0,408	0,315	0,769	2,765	0,253	0,730	0,100	1,552	0,756	1,602
V394L	Likely pathogenic	Severe	24,00	0,851	0,717	0,835	0,110	0,864	1,511	0,253	0,110	0,100	1,236	-0,103	1,617
V394M	Likely pathogenic		25,00	0,867	0,744	0,667	0,198	0,827	0,431	0,253	0,110	0,100	1,330	-0,059	1,685
R395C	Likely pathogenic	Mild	25,40	0,805	0,590	0,234	0,188	0,880	0,586	0,591	1,660	2,260	-0,323	3,580	-0,660
R395G	VUS		22,10	0,528	0,512	0,121	0,121	0,519	-0,533	0,591	0,910	2,260	-2,193	2,219	-2,062
R395H	VUS		23,50	0,541	0,523	0,084	0,065	0,561	0,672	0,591	1,030	2,260	-2,097	2,379	-1,991
R395L	VUS		23,50	0,635	0,537	0,262	0,108	0,700	0,153	0,591	1,750	2,260	-1,345	3,438	-1,531
R395P	Likely pathogenic		24,00	0,683	0,547	0,663	0,321	0,903	3,701	0,591	0,680	2,260	0,127	2,445	-0,071
R395S	VUS		21,90	0,541	0,511	0,295	0,090	0,493	0,532	0,591	0,930	2,260	-2,061	2,260	-1,928
N396D	Likely pathogenic		25,50	0,901	0,745	0,953	0,249	0,909	2,370	0,292	0,140	1,810	1,677	1,200	1,776
N396H	Likely pathogenic		25,20	0,951	0,715	0,934	0,242	0,922	0,221	0,292	0,440	1,810	1,597	1,585	1,606
N396I	Likely pathogenic		27,20	0,945	0,713	0,943	0,364	0,939	2,942	0,292	1,090	1,810	2,209	2,484	1,948
N396K	Likely pathogenic		23,80	0,862	0,757	0,993	0,317	0,921	2,048	0,292	1,730	1,810	1,841	3,138	1,540
N396S	Likely pathogenic		25,60	0,942	0,671	0,798	0,283	0,921	2,009	0,292	0,340	1,810	1,516	1,419	1,505
N396T	Likely pathogenic	Mild	26,30	0,943	0,702	0,952	0,265	0,923	3,576	0,292	0,390	1,810	1,839	1,535	1,815
N396Y	Likely pathogenic		25,90	0,969	0,737	0,898	0,354	0,929	0,370	0,292	0,820	1,810	2,006	2,139	1,850
F397C	Likely pathogenic		27,90	0,856	0,717	0,705	0,239	0,826	2,117	0,100	0,120	1,350	1,618	0,447	1,871
F397I	Likely pathogenic		29,10	0,838	0,695	0,562	0,142	0,807	1,257	0,100	0,100	1,350	1,159	0,344	1,486
F397L	Likely pathogenic		28,30	0,761	0,723	0,910	0,096	0,570	0,522	0,100	0,030	1,350	0,909	0,210	1,397
F397S	Likely pathogenic	Mild	27,80	0,872	0,693	0,572	0,182	0,823	2,655	0,100	0,850	1,350	1,370	1,259	1,472
F397V	Likely pathogenic		28,90	0,860	0,674	0,454	0,131	0,593	1,441	0,100	0,140	1,350	0,684	0,278	1,077
F397Y	Likely pathogenic		22,50	0,631	0,708	0,131	0,059	0,432	0,454	0,100	0,370	1,350	-0,917	0,101	-0,295
V398A	Likely pathogenic		24,10	0,777	0,735	0,531	0,211	0,912	1,736	0,011	0,440	0,560	1,396	0,030	1,731
V398D	Likely pathogenic		24,50	0,865	0,738	0,981	0,385	0,905	3,916	0,011	1,190	0,560	2,650	1,208	2,617
V398F	Likely pathogenic	Severe	24,40	0,930	0,705	0,946	0,374	0,947	3,754	0,011	0,140	0,560	2,601	-0,060	2,780
V398G	Likely pathogenic		24,10	0,854	0,639	0,767	0,422	0,785	2,386	0,011	0,730	0,560	1,940	0,519	2,029
V398I	Likely pathogenic	Mild	22,40	0,753	0,593	0,352	0,154	0,902	0,969	0,011	0,040	0,560	0,483	-0,656	0,932
V398L	Likely pathogenic	Severe	22,60	0,791	0,716	0,853	0,211	0,914	2,124	0,011	0,110	0,560	1,622	-0,326	2,010
D399A	Likely pathogenic		28,40	0,954	0,679	0,965	0,332	0,947	4,499	0,010	0,750	-0,690	3,111	0,049	3,202
D399E	Likely pathogenic		23,10	0,798	0,708	0,956	0,209	0,926	5,201	0,010	0,290	-0,690	2,169	-0,809	2,568
D399G	Likely pathogenic		27,80	0,954	0,692	0,962	0,275	0,949	5,676	0,010	0,460	-0,690	2,990	-0,358	3,190
D399H	Pathogenic	Mild	24,40	0,936	0,751	0,992	0,341	0,950	34,581	0,010	0,580	-0,690	4,248	-0,484	4,275
D399N	Pathogenic	Severe	24,50	0,805	0,730	0,940	0,223	0,942	5,653	0,010	0,140	-0,690	2,382	-0,936	2,797
D399V	Likely pathogenic		27,40	0,962	0,674	0,986	0,379	0,943	6,265	0,010	1,190	-0,690	3,281	0,578	3,228

D399Y	Likely pathogenic		27,20	0,929	0,733	0,985	0,377	0,943	36,243	0,010	0,960	-0,690	4,552	0,049	4,431
S400C	Likely pathogenic		26,90	0,949	0,672	0,458	0,265	0,925	1,485	0,000	0,730	-0,930	2,164	-0,348	2,394
S400G	Likely pathogenic		26,20	0,917	0,623	0,491	0,148	0,849	0,314	0,000	0,020	-0,930	1,479	-1,342	1,982
S400I	Likely pathogenic		25,50	0,942	0,676	0,972	0,296	0,949	7,511	0,000	0,750	-0,930	2,990	-0,240	3,133
S400N	Likely pathogenic		24,70	0,850	0,701	0,961	0,237	0,948	7,346	0,000	0,340	-0,930	2,625	-0,834	2,959
S400R	Likely pathogenic		29,90	0,956	0,708	0,997	0,309	0,948	11,701	0,000	0,930	-0,930	3,644	0,101	3,685
S400T	Likely pathogenic		24,80	0,852	0,660	0,660	0,205	0,864	1,855	0,000	0,050	-0,930	1,757	-1,289	2,244
P401A	Likely pathogenic		24,10	0,787	0,699	0,193	0,171	0,711	2,681	0,000	0,520	-1,030	0,937	-1,021	1,470
P401H	Likely pathogenic		27,50	0,877	0,756	0,952	0,300	0,937	66,184	0,000	0,350	-1,030	5,564	-1,225	5,533
P401L	Likely pathogenic	Mild	28,30	0,896	0,773	0,703	0,232	0,946	3,690	0,000	1,070	-1,030	2,760	0,105	2,951
P401R	Likely pathogenic		27,50	0,903	0,766	0,970	0,331	0,951	11,830	0,000	0,680	-1,030	3,546	-0,327	3,706
P401S	Likely pathogenic		26,90	0,857	0,708	0,389	0,176	0,740	3,173	0,000	0,250	-1,030	1,563	-1,170	2,086
P401T	Likely pathogenic		26,80	0,809	0,736	0,554	0,187	0,787	2,490	0,000	0,300	-1,030	1,786	-1,048	2,297
I402F	Likely pathogenic	Severe	22,80	0,806	0,460	0,785	0,304	0,821	11,039	0,000	0,100	-1,120	1,783	-1,497	2,113
I402L	Likely pathogenic		23,90	0,690	0,451	0,346	0,150	0,660	0,932	0,000	0,070	-1,120	0,108	-1,747	0,719
I402M	Likely pathogenic		26,70	0,673	0,419	0,355	0,166	0,853	0,726	0,000	0,070	-1,120	0,523	-1,611	1,026
I402N	Likely pathogenic		34,00	0,879	0,522	0,967	0,380	0,953	4,336	0,000	1,090	-1,120	3,238	0,282	3,168
I402S	Likely pathogenic		28,60	0,900	0,460	0,922	0,316	0,913	4,614	0,000	0,750	-1,120	2,471	-0,364	2,547
I402T	Likely pathogenic	Mild	26,90	0,925	0,549	0,870	0,300	0,945	2,406	0,000	0,700	-1,120	2,470	-0,418	2,614
I402V	Likely pathogenic		15,19	0,485	0,375	0,064	0,086	0,302	0,506	0,000	0,040	-1,120	-2,098	-2,394	-1,172
I403F	Likely pathogenic		27,40	0,881	0,618	0,799	0,349	0,957	10,564	0,000	0,100	-0,970	2,902	-1,102	3,149
I403L	Likely pathogenic		24,80	0,821	0,615	0,242	0,182	0,846	0,784	0,000	0,070	-0,970	1,012	-1,452	1,546
I403M	Likely pathogenic		23,60	0,724	0,585	0,361	0,279	0,891	1,990	0,000	0,070	-0,970	1,151	-1,453	1,628
I403N	Likely pathogenic		27,80	0,897	0,695	0,971	0,425	0,957	3,150	0,000	1,090	-0,970	3,283	0,311	3,296
I403S	Likely pathogenic		28,00	0,936	0,627	0,937	0,428	0,956	4,815	0,000	0,750	-0,970	3,225	-0,129	3,265
I403T	Likely pathogenic		26,80	0,936	0,711	0,786	0,345	0,950	3,063	0,000	0,700	-0,970	2,887	-0,267	3,060
I403V	Likely pathogenic		24,10	0,755	0,542	0,157	0,150	0,596	0,673	0,000	0,040	-0,970	0,112	-1,697	0,770
V404A	Likely pathogenic		26,80	0,848	0,726	0,490	0,247	0,906	2,970	0,000	0,440	-0,360	1,982	-0,423	2,312
V404E	Likely pathogenic		27,80	0,947	0,717	0,979	0,444	0,941	8,069	0,000	1,480	-0,360	3,572	1,141	3,402
V404G	Likely pathogenic		27,70	0,949	0,629	0,831	0,446	0,781	4,442	0,000	0,730	-0,360	2,754	0,120	2,816
V404I	Likely pathogenic		23,60	0,726	0,586	0,105	0,166	0,712	-0,280	0,000	0,040	-0,360	0,121	-1,313	0,725
V404L	Likely pathogenic		23,90	0,876	0,711	0,638	0,320	0,923	2,737	0,000	0,110	-0,360	2,128	-0,808	2,483
D405A	Likely pathogenic		28,20	0,926	0,653	0,624	0,212	0,875	1,742	0,083	0,750	0,500	1,764	0,690	1,881
D405E	Likely pathogenic		25,20	0,793	0,683	0,536	0,090	0,821	0,896	0,083	0,290	0,500	0,829	-0,114	1,252
D405G	Likely pathogenic		28,30	0,939	0,665	0,719	0,224	0,902	2,885	0,083	0,460	0,500	2,022	0,383	2,179
D405H	Likely pathogenic		24,40	0,929	0,720	0,658	0,222	0,875	2,624	0,083	0,580	0,500	1,744	0,412	1,947
D405N	VUS		21,30	0,609	0,703	0,178	0,074	0,598	0,664	0,083	0,140	0,500	-0,537	-0,676	0,152
D405V	Likely pathogenic		27,90	0,942	0,649	0,702	0,187	0,894	1,090	0,083	1,190	0,500	1,822	1,246	1,834
D405Y	Likely pathogenic		24,50	0,932	0,709	0,668	0,175	0,888	0,569	0,083	0,960	0,500	1,592	0,875	1,731
I406F	VUS		26,20	0,634	0,509	0,114	0,119	0,624	0,740	0,437	0,100	0,750	-1,061	0,228	-0,681
I406L	VUS		22,90	0,472	0,503	0,087	-0,030	0,482	0,596	0,437	0,070	0,750	-2,223	-0,121	-1,603
I406M	VUS		13,96	0,413	0,475	0,081	-0,019	0,434	-0,216	0,437	0,070	0,750	-3,115	-0,415	-2,400
I406N	Likely pathogenic		22,10	0,673	0,560	0,138	0,119	0,560	1,565	0,437	1,090	0,750	-1,115	1,342	-0,920
I406S	Likely pathogenic		21,60	0,653	0,504	0,127	0,099	0,312	1,490	0,437	0,750	0,750	-1,779	0,782	-1,410
I406T	VUS		21,40	0,602	0,571	0,108	0,024	0,421	0,940	0,437	0,700	0,750	-1,798	0,711	-1,355
I406V	VUS		22,40	0,430	0,440	0,067	-0,083	0,359	0,701	0,437	0,040	0,750	-2,817	-0,295	-2,119
T407A	VUS		12,97	0,294	0,406	0,052	-0,054	0,144	0,158	0,703	0,220	0,610	-4,435	-0,012	-3,696
T407I	VUS		17,95	0,483	0,486	0,115	-0,007	0,417	0,942	0,703	0,700	0,610	-2,878	0,970	-2,470
T407N	VUS		16,93	0,362	0,443	0,067	0,016	0,309	0,382	0,703	0,390	0,610	-3,488	0,452	-2,937
T407P	VUS		15,11	0,360	0,424	0,058	0,126	0,314	-0,329	0,703	0,300	0,610	-3,437	0,349	-2,923
T407S	VUS		15,10	0,389	0,395	0,061	-0,099	0,239	-0,334	0,703	0,050	0,610	-4,118	-0,103	-3,400
K408E	VUS		22,00	0,676	0,646	0,067	-0,023	0,340	1,012	0,487	1,300	0,620	-1,711	1,491	-1,364
K408M	VUS		24,80	0,688	0,584	0,202	-0,017	0,603	0,382	0,487	2,890	0,620	-0,989	3,631	-1,188
K408N	VUS		22,20	0,407	0,672	0,171	-0,032	0,415	-0,153	0,487	1,730	0,620	-1,959	1,960	-1,625

K408Q	VUS		22,10	0,639	0,608	0,077	-0,044	0,427	-0,190	0,487	1,700	0,620	-1,792	1,981	-1,558	
K408R	VUS		22,90	0,494	0,587	0,067	-0,118	0,402	-0,073	0,487	1,140	0,620	-2,327	1,184	-1,861	
K408T	VUS		24,30	0,652	0,659	0,115	-0,023	0,446	1,383	0,487	2,120	0,620	-1,274	2,598	-1,174	
D409A	Likely pathogenic		23,90	0,734	0,612	0,422	0,108	0,690	-0,249	0,228	0,750	0,040	0,060	0,326	0,403	
D409E	Likely pathogenic		22,30	0,738	0,633	0,416	0,056	0,649	-0,380	0,228	0,290	0,040	-0,227	-0,313	0,296	
D409G	Likely pathogenic	Mild	22,80	0,719	0,624	0,177	0,098	0,516	-0,201	0,228	0,460	0,040	-0,593	-0,198	-0,072	
D409H	Pathogenic	Severe	22,80	0,738	0,672	0,545	0,115	0,711	-0,380	0,228	0,580	0,040	0,298	0,162	0,696	Yes
D409N	Likely pathogenic		22,80	0,570	0,654	0,138	0,058	0,566	-2,351	0,228	0,140	0,040	-0,981	-0,649	-0,291	
D409V	Pathogenic	Severe	26,30	0,887	0,607	0,642	0,163	0,906	0,764	0,228	1,190	0,040	1,278	1,164	1,300	
D409Y	Likely pathogenic		26,20	0,903	0,657	0,485	0,229	0,887	-0,398	0,228	0,960	0,040	1,314	0,902	1,391	
T410A	VUS		21,30	0,319	0,402	0,068	0,036	0,268	1,657	0,081	0,220	-0,350	-2,286	-1,511	-1,426	
T410K	VUS		22,10	0,422	0,446	0,169	0,132	0,430	0,083	0,081	2,120	-0,350	-1,271	1,053	-1,017	
T410M	VUS		22,40	0,468	0,412	0,082	0,093	0,558	-0,753	0,081	0,770	-0,350	-1,375	-0,575	-0,841	
T410P	VUS		22,40	0,551	0,414	0,588	0,286	0,722	10,777	0,081	0,300	-0,350	0,490	-0,896	0,829	
T410R	VUS		22,20	0,472	0,436	0,103	0,134	0,562	0,224	0,081	0,980	-0,350	-1,142	-0,291	-0,682	
T410S	VUS		21,50	0,359	0,405	0,098	0,017	0,403	1,659	0,081	0,050	-0,350	-2,011	-1,647	-1,166	
F411C	Likely pathogenic		29,80	0,934	0,744	0,943	0,387	0,937	3,780	0,000	0,120	-0,530	3,326	-0,568	3,562	
F411I	Pathogenic		25,70	0,883	0,726	0,693	0,287	0,941	0,902	0,000	0,100	-0,530	2,269	-0,840	2,649	
F411L	Likely pathogenic		26,00	0,848	0,748	0,984	0,325	0,929	0,807	0,000	0,030	-0,530	2,659	-0,825	3,038	
F411S	Likely pathogenic		29,80	0,957	0,728	0,978	0,398	0,950	5,630	0,000	0,850	-0,530	3,547	0,335	3,566	
F411V	Likely pathogenic		25,80	0,891	0,712	0,664	0,290	0,918	1,616	0,000	0,140	-0,530	2,228	-0,812	2,593	
F411Y	Likely pathogenic		27,40	0,818	0,739	0,540	0,222	0,808	4,652	0,000	0,370	-0,530	1,944	-0,647	2,355	
Y412C	Likely pathogenic		29,40	0,965	0,779	0,367	0,388	0,947	5,164	0,015	0,250	-0,740	2,907	-0,655	3,157	
Y412D	Likely pathogenic		28,50	0,943	0,772	0,930	0,431	0,934	6,621	0,015	0,960	-0,740	3,607	0,345	3,627	
Y412F	Likely pathogenic		26,80	0,901	0,719	0,132	0,175	0,860	1,585	0,015	0,370	-0,740	1,421	-0,822	1,860	
Y412H	Likely pathogenic		28,20	0,944	0,781	0,412	0,325	0,942	4,795	0,015	0,380	-0,740	2,675	-0,559	2,951	
Y412N	Likely pathogenic		28,40	0,942	0,777	0,798	0,410	0,950	5,522	0,015	0,820	-0,740	3,382	0,137	3,461	
Y412S	Likely pathogenic		28,40	0,953	0,738	0,653	0,422	0,947	7,050	0,015	0,480	-0,740	3,208	-0,338	3,345	
K413E	Likely pathogenic		28,00	0,950	0,589	0,971	0,331	0,938	3,207	0,004	1,300	-0,870	2,887	0,565	2,836	
K413I	Likely pathogenic		29,20	0,899	0,570	0,948	0,344	0,927	2,197	0,004	2,820	-0,870	2,889	2,429	2,474	
K413N	Likely pathogenic		24,40	0,840	0,611	0,996	0,324	0,922	-0,125	0,004	1,730	-0,870	2,359	0,971	2,311	
K413Q	Likely pathogenic	Mild	27,30	0,951	0,558	0,801	0,232	0,915	0,491	0,004	1,700	-0,870	2,224	0,933	2,164	
K413R	Likely pathogenic		27,80	0,887	0,534	0,214	0,158	0,896	-0,740	0,004	1,140	-0,870	1,132	0,014	1,319	
K413T	Likely pathogenic		28,00	0,907	0,596	0,967	0,328	0,924	0,909	0,004	2,120	-0,870	2,756	1,561	2,545	
Q414E	Likely pathogenic		26,80	0,955	0,667	0,419	0,205	0,931	0,155	0,009	0,400	-0,860	1,873	-0,726	2,216	
Q414H	Likely pathogenic		24,00	0,935	0,696	0,956	0,274	0,921	0,765	0,009	0,470	-0,860	2,481	-0,532	2,768	
Q414K	Likely pathogenic		27,70	0,931	0,700	0,937	0,306	0,925	3,031	0,009	1,700	-0,860	2,990	1,070	2,921	
Q414L	Likely pathogenic		28,30	0,967	0,660	0,846	0,292	0,926	-1,656	0,009	1,190	-0,860	2,644	0,470	2,704	
Q414P	Likely pathogenic		27,60	0,962	0,671	0,958	0,447	0,944	2,237	0,009	0,120	-0,860	3,210	-0,765	3,410	
Q414R	Likely pathogenic	Severe	27,20	0,955	0,698	0,959	0,305	0,945	4,685	0,009	0,560	-0,860	3,031	-0,328	3,215	
P415A	Likely pathogenic		26,60	0,907	0,763	0,769	0,323	0,919	3,360	0,006	0,520	-0,940	2,807	-0,486	3,078	
P415H	Likely pathogenic		28,70	0,959	0,801	0,983	0,362	0,936	50,125	0,006	0,350	-0,940	5,408	-0,892	5,396	
P415L	Likely pathogenic		29,20	0,936	0,815	0,939	0,372	0,942	5,415	0,006	1,070	-0,940	3,634	0,362	3,701	
P415R	Likely pathogenic	Severe	28,70	0,968	0,810	0,979	0,393	0,943	25,457	0,006	0,680	-0,940	4,539	-0,258	4,563	
P415S	Likely pathogenic		27,60	0,823	0,772	0,898	0,323	0,914	4,852	0,006	0,250	-0,940	2,914	-0,803	3,265	
P415T	Likely pathogenic		27,10	0,960	0,792	0,926	0,267	0,941	4,937	0,006	0,300	-0,940	3,132	-0,694	3,452	
M416I	Likely pathogenic		25,10	0,689	0,548	0,800	0,116	0,733	3,376	0,004	0,070	-0,730	0,964	-1,324	1,527	
M416K	Likely pathogenic		24,90	0,742	0,609	0,913	0,350	0,866	2,869	0,004	2,890	-0,730	2,269	2,381	1,955	
M416L	Likely pathogenic		23,90	0,694	0,539	0,339	0,088	0,528	1,178	0,004	0,000	-0,730	-0,110	-1,644	0,610	
M416R	Likely pathogenic		25,10	0,761	0,591	0,916	0,377	0,909	6,131	0,004	1,750	-0,730	2,458	0,998	2,354	
M416T	Likely pathogenic		23,20	0,670	0,627	0,284	0,161	0,666	3,830	0,004	0,770	-0,730	0,491	-0,649	0,970	
M416V	Likely pathogenic		23,70	0,760	0,485	0,244	0,169	0,741	3,862	0,004	0,110	-0,730	0,401	-1,436	0,911	
F417C	Likely pathogenic		31,00	0,968	0,827	0,962	0,265	0,931	3,065	0,000	0,120	-1,180	3,492	-0,917	3,870	
F417I	Likely pathogenic		27,80	0,967	0,811	0,918	0,309	0,937	2,402	0,000	0,100	-1,180	3,264	-1,019	3,643	

F417L	Likely pathogenic		28,30	0,955	0,832	0,988	0,271	0,916	0,017	0,000	0,030	-1,180	3,176	-1,076	3,621	
F417S	Likely pathogenic		31,00	0,969	0,811	0,975	0,344	0,947	3,813	0,000	0,850	-1,180	3,768	0,016	3,902	
F417V	Likely pathogenic		28,00	0,976	0,803	0,904	0,303	0,919	2,942	0,000	0,140	-1,180	3,245	-0,981	3,614	
F417Y	Likely pathogenic		24,40	0,722	0,821	0,212	0,155	0,660	0,498	0,000	0,370	-1,180	0,961	-1,268	1,656	
Y418C	Likely pathogenic		32,00	0,964	0,787	0,739	0,401	0,944	4,061	0,000	0,250	-1,340	3,643	-0,834	3,898	
Y418D	Likely pathogenic		32,00	0,964	0,787	0,969	0,377	0,940	2,930	0,000	0,960	-1,340	3,838	0,095	3,928	
Y418F	Likely pathogenic		28,40	0,889	0,720	0,225	0,190	0,794	0,665	0,000	0,370	-1,340	1,656	-1,140	2,151	
Y418H	Likely pathogenic		31,00	0,916	0,793	0,734	0,254	0,930	4,200	0,000	0,380	-1,340	3,131	-0,813	3,480	
Y418N	Likely pathogenic		32,00	0,958	0,793	0,899	0,424	0,947	4,020	0,000	0,820	-1,340	3,920	-0,078	4,016	
Y418S	Likely pathogenic		32,00	0,967	0,733	0,836	0,435	0,947	4,257	0,000	0,480	-1,340	3,738	-0,527	3,883	
H419D	Likely pathogenic		33,00	0,744	0,694	0,939	0,161	0,897	1,013	0,000	0,580	-1,350	2,549	-0,621	2,931	
H419L	Likely pathogenic		25,90	0,705	0,663	0,536	0,163	0,756	-0,393	0,000	0,720	-1,350	1,205	-0,831	1,696	
H419N	Likely pathogenic		32,00	0,670	0,694	0,572	0,052	0,803	0,589	0,000	0,440	-1,350	1,503	-1,051	2,085	
H419P	Likely pathogenic		26,50	0,767	0,629	0,836	0,277	0,887	2,509	0,000	0,350	-1,350	2,175	-1,079	2,557	
H419Q	Likely pathogenic		24,70	0,701	0,668	0,851	0,082	0,759	1,381	0,000	0,470	-1,350	1,334	-1,137	1,917	
H419R	Likely pathogenic		28,20	0,699	0,700	0,935	0,150	0,839	7,009	0,000	1,030	-1,350	2,302	-0,301	2,627	
H419Y	VUS		32,00	0,611	0,702	0,563	0,085	0,723	2,228	0,000	0,380	-1,350	1,423	-1,176	2,046	
L420F	Likely pathogenic		29,20	0,819	0,634	0,436	0,148	0,786	0,030	0,000	0,030	-1,470	1,475	-1,637	2,063	
L420H	Likely pathogenic		26,80	0,868	0,708	0,880	0,325	0,915	1,489	0,000	0,720	-1,470	2,790	-0,549	3,047	
L420I	Likely pathogenic		27,80	0,644	0,635	0,137	0,062	0,473	1,570	0,000	0,070	-1,470	0,107	-1,967	0,944	
L420P	Likely pathogenic		27,30	0,930	0,741	0,977	0,366	0,941	8,484	0,000	1,070	-1,470	3,580	-0,062	3,657	
L420R	Likely pathogenic		27,20	0,908	0,714	0,877	0,363	0,927	7,727	0,000	1,750	-1,470	3,348	0,716	3,276	
L420V	Likely pathogenic		27,60	0,750	0,586	0,163	0,111	0,698	2,034	0,000	0,110	-1,470	0,682	-1,784	1,332	
G421A	Likely pathogenic		23,30	0,627	0,704	0,118	0,149	0,512	0,152	0,000	0,290	-1,450	0,120	-1,725	0,916	
G421C	Likely pathogenic		26,00	0,939	0,731	0,498	0,313	0,863	3,931	0,000	0,750	-1,450	2,494	-0,634	2,770	
G421D	Likely pathogenic		28,70	0,941	0,790	0,967	0,314	0,936	8,735	0,000	0,460	-1,450	3,633	-0,767	3,896	
G421R	Likely pathogenic		25,60	0,931	0,773	0,906	0,330	0,904	22,111	0,000	0,910	-1,450	3,861	-0,445	3,967	
G421S	Likely pathogenic		25,10	0,914	0,681	0,193	0,172	0,850	3,517	0,000	0,020	-1,450	1,500	-1,750	2,064	
G421V	Likely pathogenic		28,70	0,921	0,710	0,708	0,273	0,870	9,324	0,000	0,730	-1,450	2,966	-0,604	3,201	
H422D	Likely pathogenic		26,60	0,977	0,722	0,983	0,330	0,948	3,077	0,000	0,580	-1,580	3,270	-0,699	3,503	
H422L	Likely pathogenic		27,20	0,976	0,700	0,811	0,333	0,948	-0,128	0,000	0,720	-1,580	2,953	-0,542	3,166	
H422N	Likely pathogenic		26,00	0,942	0,722	0,854	0,290	0,949	0,349	0,000	0,440	-1,580	2,801	-0,941	3,134	
H422P	Likely pathogenic		27,20	0,978	0,669	0,935	0,379	0,952	1,710	0,000	0,350	-1,580	3,185	-0,959	3,425	
H422Q	Likely pathogenic		24,00	0,859	0,702	0,892	0,168	0,929	2,575	0,000	0,470	-1,580	2,268	-1,090	2,701	
H422R	Likely pathogenic		26,30	0,977	0,729	0,973	0,320	0,951	10,474	0,000	1,030	-1,580	3,570	-0,223	3,663	
H422Y	Likely pathogenic		25,80	0,977	0,728	0,878	0,255	0,943	9,128	0,000	0,380	-1,580	3,156	-1,087	3,468	Yes
F423C	Likely pathogenic		29,60	0,972	0,785	0,943	0,376	0,949	4,977	0,000	0,120	-1,420	3,697	-1,065	4,000	
F423I	Likely pathogenic		27,80	0,958	0,759	0,696	0,273	0,944	4,591	0,000	0,100	-1,420	2,940	-1,283	3,341	
F423L	Likely pathogenic		28,30	0,957	0,788	0,983	0,238	0,948	3,101	0,000	0,030	-1,420	3,213	-1,266	3,648	
F423S	Likely pathogenic		29,60	0,970	0,770	0,983	0,319	0,956	4,861	0,000	0,850	-1,420	3,625	-0,196	3,775	
F423V	Likely pathogenic		27,90	0,966	0,755	0,675	0,261	0,934	3,647	0,000	0,140	-1,420	2,849	-1,237	3,252	
F423Y	Likely pathogenic		27,30	0,924	0,775	0,761	0,221	0,890	2,569	0,000	0,370	-1,420	2,682	-0,989	3,099	
S424C	Likely pathogenic		27,40	0,979	0,700	0,464	0,294	0,933	1,026	0,006	0,730	-1,160	2,430	-0,403	2,651	
S424G	Likely pathogenic		26,70	0,969	0,652	0,420	0,185	0,944	1,124	0,006	0,020	-1,160	1,907	-1,385	2,356	
S424I	Likely pathogenic		25,70	0,978	0,715	0,961	0,325	0,952	1,128	0,006	0,750	-1,160	3,001	-0,254	3,177	
S424N	Likely pathogenic		24,90	0,932	0,727	0,956	0,266	0,954	5,055	0,006	0,340	-1,160	2,871	-0,862	3,190	
S424R	Likely pathogenic		27,20	0,946	0,738	0,987	0,265	0,940	4,022	0,006	0,930	-1,160	3,099	-0,053	3,265	
S424T	VUS		24,60	0,895	0,692	0,404	0,147	0,937	-0,024	0,006	0,050	-1,160	1,546	-1,446	2,084	
K425E	Likely pathogenic	Severe	27,30	0,964	0,671	0,650	0,295	0,930	3,814	0,008	1,300	-1,390	2,729	0,173	2,795	
K425M	Likely pathogenic		32,00	0,940	0,617	0,545	0,280	0,887	-1,114	0,008	2,890	-1,390	2,586	2,202	2,273	
K425N	Likely pathogenic		25,00	0,868	0,697	0,804	0,288	0,901	3,143	0,008	1,730	-1,390	2,560	0,634	2,596	
K425Q	Likely pathogenic		26,70	0,936	0,636	0,249	0,190	0,891	3,321	0,008	1,700	-1,390	1,805	0,434	1,883	
K425R	Likely pathogenic		29,80	0,900	0,613	0,067	0,113	0,828	1,507	0,008	1,140	-1,390	1,303	-0,293	1,591	
K425T	Likely pathogenic		31,00	0,941	0,682	0,515	0,219	0,891	3,478	0,008	2,120	-1,390	2,628	1,180	2,551	

F426C	VUS		30,00	0,951	0,809	0,877	0,318	0,953	4,332	0,004	0,120	-1,450	3,514	-1,107	3,874
F426I	VUS		31,00	0,941	0,793	0,838	0,362	0,944	2,692	0,004	0,100	-1,450	3,505	-1,092	3,848
F426L	VUS		32,00	0,879	0,812	0,978	0,251	0,947	1,162	0,004	0,030	-1,450	3,328	-1,179	3,779
F426S	VUS		30,00	0,962	0,790	0,943	0,329	0,953	5,211	0,004	0,850	-1,450	3,678	-0,199	3,833
F426V	VUS		31,00	0,923	0,776	0,808	0,284	0,949	3,925	0,004	0,140	-1,450	3,271	-1,119	3,645
F426Y	VUS		27,60	0,810	0,804	0,277	0,225	0,798	-0,005	0,004	0,370	-1,450	1,783	-1,182	2,335
I427F	Likely pathogenic		27,70	0,899	0,647	0,366	0,162	0,871	6,563	0,005	0,100	-1,180	1,842	-1,408	2,306
I427L	VUS		22,90	0,591	0,641	0,164	0,060	0,385	1,234	0,005	0,070	-1,180	-0,510	-1,970	0,398
I427M	VUS		25,30	0,758	0,607	0,271	0,097	0,752	-0,555	0,005	0,070	-1,180	0,576	-1,643	1,233
I427N	Likely pathogenic		29,90	0,916	0,718	0,932	0,316	0,958	2,470	0,005	1,090	-1,180	3,227	0,212	3,313
I427S	Likely pathogenic		31,00	0,966	0,659	0,876	0,319	0,956	3,537	0,005	0,750	-1,180	3,222	-0,196	3,334
I427T	Likely pathogenic		28,20	0,936	0,737	0,884	0,236	0,885	2,335	0,005	0,700	-1,180	2,804	-0,376	3,078
I427V	VUS		22,90	0,639	0,560	0,170	0,023	0,382	1,137	0,005	0,040	-1,180	-0,705	-2,035	0,182
P428A	VUS		24,30	0,767	0,523	0,081	0,055	0,547	2,109	0,208	0,520	-0,880	-0,489	-0,734	0,027
P428H	VUS		27,90	0,697	0,591	0,177	0,096	0,673	2,749	0,208	0,350	-0,880	0,202	-0,760	0,705
P428L	VUS		25,60	0,814	0,611	0,104	0,089	0,428	0,733	0,208	1,070	-0,880	-0,183	0,030	0,236
P428R	VUS		24,90	0,694	0,602	0,095	0,110	0,608	1,390	0,208	0,680	-0,880	-0,177	-0,464	0,305
P428S	VUS		25,20	0,858	0,527	0,115	0,047	0,770	2,602	0,208	0,250	-0,880	0,110	-0,914	0,569
P428T	VUS		27,10	0,734	0,560	0,108	0,060	0,642	2,418	0,208	0,300	-0,880	-0,086	-0,885	0,448
E429A	VUS		25,00	0,796	0,632	0,156	0,058	0,696	0,291	0,368	1,040	-0,580	-0,126	0,570	0,159
E429D	VUS		22,80	0,817	0,631	0,291	0,040	0,623	0,656	0,368	0,290	-0,580	-0,288	-0,397	0,214
E429G	VUS		29,60	0,902	0,578	0,243	0,100	0,857	0,787	0,368	0,750	-0,580	0,706	0,474	0,867
E429K	VUS		22,40	0,706	0,716	0,186	0,081	0,602	0,404	0,368	1,300	-0,580	-0,309	0,794	0,022
E429Q	VUS		24,20	0,781	0,605	0,166	0,047	0,786	0,349	0,368	0,400	-0,580	-0,203	-0,226	0,211
E429V	VUS		25,40	0,830	0,614	0,336	0,077	0,781	0,211	0,368	1,480	-0,580	0,325	1,221	0,424
G430A	VUS		25,00	0,917	0,628	0,243	0,206	0,727	2,809	0,269	0,290	-0,480	0,755	-0,335	1,081
G430C	VUS		28,40	0,929	0,665	0,437	0,334	0,846	2,574	0,269	0,750	-0,480	1,827	0,512	1,891
G430D	VUS		23,50	0,824	0,716	0,108	0,239	0,603	3,204	0,269	0,460	-0,480	0,454	-0,246	0,856
G430R	Likely pathogenic		27,70	0,938	0,707	0,397	0,351	0,842	2,561	0,269	0,910	-0,480	1,895	0,706	1,935
G430S	VUS		26,90	0,929	0,623	0,130	0,202	0,750	3,171	0,269	0,020	-0,480	0,793	-0,639	1,163
G430V	Likely pathogenic		25,60	0,936	0,634	0,463	0,361	0,835	4,073	0,269	0,730	-0,480	1,708	0,407	1,760
S431A	VUS		25,40	0,834	0,601	0,162	0,136	0,613	-0,801	0,000	0,270	-0,460	0,347	-1,010	0,907
S431C	VUS		32,00	0,927	0,618	0,179	0,278	0,864	-0,295	0,000	0,730	-0,460	1,819	-0,046	2,003
S431F	Likely pathogenic		32,00	0,933	0,645	0,676	0,305	0,933	30,893	0,000	0,850	-0,460	3,902	0,039	3,817
S431P	Likely pathogenic		26,40	0,939	0,621	0,930	0,381	0,944	4,205	0,000	0,250	-0,460	2,798	-0,518	2,970
S431T	VUS		24,90	0,909	0,614	0,233	0,132	0,665	0,147	0,000	0,050	-0,460	0,656	-1,222	1,223
S431Y	Likely pathogenic		31,00	0,946	0,667	0,646	0,242	0,926	28,162	0,000	0,480	-0,460	3,573	-0,450	3,648
Q432E	VUS		22,00	0,531	0,634	0,095	-0,023	0,560	0,452	0,191	0,400	-0,460	-1,124	-0,827	-0,395
Q432H	VUS		22,00	0,640	0,666	0,274	-0,035	0,440	2,292	0,191	0,470	-0,460	-0,769	-0,686	-0,057
Q432K	VUS		22,00	0,544	0,673	0,128	-0,008	0,579	0,047	0,191	1,700	-0,460	-0,828	0,812	-0,419
Q432L	VUS		22,30	0,574	0,625	0,138	-0,017	0,549	-0,634	0,191	1,190	-0,460	-0,985	0,185	-0,468
Q432P	VUS		23,60	0,832	0,640	0,560	0,222	0,868	3,328	0,191	0,120	-0,460	1,232	-0,623	1,590
Q432R	VUS		22,10	0,528	0,670	0,090	-0,016	0,249	0,251	0,191	0,560	-0,460	-1,487	-0,718	-0,670
R433G	VUS	Mild	26,70	0,864	0,567	0,807	0,303	0,952	1,936	0,069	0,910	0,280	1,952	0,751	1,952
R433I	VUS		24,70	0,920	0,600	0,572	0,300	0,897	-2,029	0,069	1,680	0,280	1,542	1,622	1,423
R433K	VUS		24,20	0,906	0,636	0,469	0,151	0,883	-0,543	0,069	1,140	0,280	1,093	0,826	1,227
R433S	VUS		26,70	0,828	0,575	0,934	0,344	0,951	1,916	0,069	0,930	0,280	2,141	0,820	2,120
R433T	Likely pathogenic		24,20	0,960	0,612	0,838	0,348	0,945	0,796	0,069	0,980	0,280	2,157	0,874	2,120
V434A	VUS		27,00	0,906	0,547	0,271	0,161	0,873	1,826	0,017	0,440	-0,540	1,150	-0,643	1,472
V434E	Likely pathogenic		28,00	0,942	0,531	0,841	0,352	0,956	3,722	0,017	1,480	-0,540	2,618	0,951	2,458
V434G	Likely pathogenic		27,80	0,933	0,448	0,454	0,354	0,905	3,002	0,017	0,730	-0,540	1,807	-0,131	1,848
V434L	VUS		22,10	0,742	0,526	0,318	0,141	0,710	0,221	0,017	0,110	-0,540	0,115	-1,312	0,687
V434M	VUS		23,60	0,830	0,556	0,295	0,160	0,847	-0,202	0,017	0,110	-0,540	0,667	-1,160	1,148
G435A	VUS		22,50	0,475	0,689	0,103	0,052	0,221	0,482	0,048	0,290	-0,520	-1,181	-1,353	-0,248

G435E	VUS	24,50	0,740	0,748	0,214	0,108	0,738	0,280	0,048	0,750	-0,520	0,657	-0,338	1,156	
G435R	VUS	25,10	0,859	0,757	0,243	0,138	0,688	0,767	0,048	0,910	-0,520	1,008	-0,060	1,416	
G435V	VUS	26,50	0,817	0,694	0,282	0,231	0,818	1,339	0,048	0,730	-0,520	1,347	-0,185	1,666	
G435W	VUS	26,10	0,888	0,750	0,428	0,242	0,844	0,344	0,048	0,760	-0,520	1,774	-0,037	2,064	
L436M	VUS	22,30	0,525	0,528	0,154	-0,005	0,391	-0,581	0,209	0,000	-0,700	-1,560	-1,479	-0,716	
L436P	VUS	28,10	0,817	0,644	0,823	0,197	0,940	4,313	0,209	1,070	-0,700	2,002	0,634	2,078	
L436Q	VUS	27,70	0,749	0,550	0,627	0,129	0,836	3,492	0,209	1,190	-0,700	1,063	0,576	1,197	
L436R	VUS	28,00	0,812	0,613	0,725	0,184	0,930	2,746	0,209	1,750	-0,700	1,743	1,422	1,670	
L436V	VUS	18,77	0,379	0,494	0,121	-0,010	0,255	2,745	0,209	0,110	-0,700	-2,273	-1,614	-1,350	
V437A	VUS	9,59	0,318	0,393	0,064	-0,068	0,111	0,382	0,425	0,440	-0,470	-4,106	-1,041	-3,205	
V437D	VUS	12,18	0,369	0,401	0,091	0,102	0,143	0,081	0,425	1,190	-0,470	-3,278	0,092	-2,713	
V437F	VUS	17,15	0,528	0,386	0,089	0,025	0,280	-0,010	0,425	0,140	-0,470	-2,727	-0,977	-2,016	
V437G	VUS	12,83	0,489	0,352	0,101	0,082	0,159	0,613	0,425	0,730	-0,470	-3,147	-0,420	-2,540	
V437I	VUS	12,91	0,280	0,346	0,080	-0,130	0,149	-0,414	0,425	0,040	-0,470	-4,194	-1,485	-3,200	
V437L	VUS	13,23	0,281	0,381	0,085	-0,065	0,152	-0,276	0,425	0,110	-0,470	-3,908	-1,339	-2,961	
A438D	VUS	22,20	0,708	0,604	0,186	0,023	0,661	0,648	0,302	0,750	-0,250	-0,645	0,115	-0,215	
A438G	VUS	22,00	0,584	0,519	0,108	-0,075	0,394	1,018	0,302	0,290	-0,250	-1,835	-0,720	-1,118	
A438P	VUS	20,30	0,725	0,579	0,097	0,057	0,717	-0,911	0,302	0,520	-0,250	-0,820	-0,196	-0,363	
A438S	VUS	17,21	0,416	0,506	0,084	-0,112	0,285	1,018	0,302	0,270	-0,250	-2,799	-1,030	-1,917	
A438T	VUS	19,98	0,534	0,532	0,078	-0,089	0,320	0,813	0,302	0,220	-0,250	-2,231	-0,921	-1,418	
A438V	VUS	21,90	0,644	0,544	0,092	-0,103	0,301	0,337	0,302	0,440	-0,250	-1,907	-0,546	-1,182	
S439C	VUS	22,90	0,774	0,576	0,174	-0,006	0,443	-0,345	0,465	0,730	-0,180	-1,221	0,417	-0,792	
S439G	VUS	21,30	0,675	0,530	0,090	-0,064	0,434	0,881	0,465	0,020	-0,180	-1,879	-0,632	-1,208	
S439I	VUS	21,70	0,659	0,587	0,208	0,004	0,679	0,071	0,465	0,750	-0,180	-1,066	0,449	-0,680	
S439N	VUS	17,60	0,393	0,602	0,119	-0,060	0,293	-0,805	0,465	0,340	-0,180	-2,719	-0,478	-1,900	
S439R	VUS	15,66	0,606	0,614	0,415	0,026	0,620	-0,123	0,465	0,930	-0,180	-1,329	0,539	-0,912	
S439T	VUS	17,15	0,619	0,563	0,093	-0,093	0,410	0,244	0,465	0,050	-0,180	-2,323	-0,743	-1,562	
Q440E	VUS	5,31	0,314	0,346	0,058	-0,133	0,168	0,339	0,444	0,400	-0,080	-4,713	-0,983	-3,804	
Q440H	VUS	12,59	0,272	0,360	0,120	-0,123	0,211	0,679	0,444	0,470	-0,080	-4,070	-0,674	-3,241	
Q440K	VUS	5,83	0,400	0,373	0,065	-0,107	0,118	-0,627	0,444	1,700	-0,080	-4,396	0,682	-3,821	
Q440L	VUS	12,52	0,363	0,349	0,113	-0,103	0,195	-0,237	0,444	1,190	-0,080	-3,895	0,256	-3,281	
Q440P	VUS	12,40	0,463	0,363	0,095	0,051	0,230	2,814	0,444	0,120	-0,080	-3,235	-0,934	-2,517	
Q440R	VUS	8,79	0,393	0,362	0,064	-0,103	0,177	-1,351	0,444	0,560	-0,080	-4,244	-0,609	-3,437	
K441E	VUS	12,46	0,271	0,502	0,071	-0,073	0,199	-0,116	0,831	1,300	0,370	-4,288	1,458	-3,795	
K441M	VUS	18,39	0,389	0,468	0,146	-0,059	0,376	0,544	0,831	2,890	0,370	-3,200	3,697	-3,283	
K441N	VUS	13,76	0,249	0,524	0,124	-0,074	0,194	0,654	0,831	1,730	0,370	-4,072	2,025	-3,683	
K441Q	VUS	12,87	0,308	0,479	0,082	-0,087	0,145	0,470	0,831	1,700	0,370	-4,288	1,941	-3,897	
K441R	VUS	13,82	0,287	0,473	0,078	-0,162	0,163	0,334	0,831	1,140	0,370	-4,481	1,234	-3,914	
K441T	VUS	14,71	0,377	0,514	0,097	-0,068	0,247	0,493	0,831	2,120	0,370	-3,699	2,603	-3,486	
N442D	VUS	13,80	0,396	0,464	0,085	-0,068	0,230	1,057	0,256	0,140	0,220	-3,193	-1,128	-2,287	
N442H	VUS	13,97	0,352	0,445	0,091	-0,062	0,251	-0,397	0,256	0,440	0,220	-3,295	-0,762	-2,458	
N442I	VUS	7,69	0,487	0,468	0,163	-0,014	0,228	-0,744	0,256	1,090	0,220	-3,230	-0,023	-2,569	
N442K	VUS	1,80	0,338	0,470	0,173	-0,072	0,224	0,313	0,256	1,730	0,220	-3,968	0,483	-3,327	
N442S	VUS	1,23	0,329	0,417	0,070	-0,113	0,124	0,993	0,256	0,340	0,220	-4,587	-1,350	-3,557	
N442T	VUS	1,07	0,340	0,433	0,068	-0,068	0,195	0,698	0,256	0,390	0,220	-4,335	-1,230	-3,365	
N442Y	VUS	15,24	0,523	0,461	0,111	-0,011	0,342	0,557	0,256	0,820	0,220	-2,495	-0,113	-1,891	
D443A	VUS	13,99	0,516	0,395	0,082	-0,059	0,285	0,226	0,565	0,750	0,600	-3,502	0,534	-2,988	
D443E	VUS	13,81	0,311	0,396	0,096	-0,117	0,177	0,225	0,565	0,290	0,600	-4,220	-0,199	-3,438	
D443G	VUS	14,21	0,381	0,391	0,074	-0,051	0,232	0,214	0,565	0,460	0,600	-3,843	0,101	-3,194	
D443H	VUS	9,40	0,446	0,423	0,091	-0,042	0,258	0,408	0,565	0,580	0,600	-3,886	0,176	-3,258	
D443N	VUS	7,74	0,333	0,412	0,077	-0,098	0,297	0,024	0,565	0,140	0,600	-4,383	-0,486	-3,573	Yes
D443V	VUS	14,75	0,559	0,384	0,109	-0,005	0,443	0,078	0,565	1,190	0,600	-2,970	1,203	-2,683	
D443Y	VUS	17,53	0,562	0,426	0,101	0,010	0,533	0,147	0,565	0,960	0,600	-2,524	1,053	-2,228	
L444M	Likely pathogenic	20,80	0,671	0,529	0,161	0,091	0,469	-0,227	0,080	0,000	0,060	-0,990	-1,194	-0,288	

L444P	Pathogenic	Severe	25,50	0,858	0,632	0,602	0,353	0,929	3,112	0,080	1,070	0,060	1,960	0,781	1,959	Yes
L444Q	Likely pathogenic		25,70	0,885	0,553	0,489	0,222	0,931	3,915	0,080	1,190	0,060	1,444	0,804	1,450	
L444R	Pathogenic	Severe	25,80	0,891	0,599	0,499	0,350	0,934	7,008	0,080	1,750	0,060	2,061	1,561	1,850	
L444V	Likely pathogenic		13,22	0,691	0,502	0,096	0,082	0,644	1,958	0,080	0,110	0,060	-1,285	-1,253	-0,635	
D445A	VUS		22,60	0,596	0,488	0,145	0,020	0,414	1,088	0,140	0,750	0,400	-1,413	-0,034	-0,885	
D445E	VUS		5,49	0,342	0,489	0,097	-0,062	0,188	0,966	0,140	0,290	0,400	-3,725	-1,333	-2,716	
D445G	VUS		22,60	0,458	0,484	0,216	0,036	0,357	2,292	0,140	0,460	0,400	-1,627	-0,459	-0,978	
D445H	VUS		23,60	0,627	0,518	0,186	0,030	0,430	1,548	0,140	0,580	0,400	-1,113	-0,167	-0,569	
D445N	VUS		22,60	0,423	0,513	0,137	-0,019	0,400	0,232	0,140	0,140	0,400	-1,886	-0,876	-1,098	
D445V	VUS		22,60	0,618	0,479	0,177	0,075	0,451	0,723	0,140	1,190	0,400	-1,155	0,566	-0,795	
D445Y	Likely pathogenic		22,80	0,661	0,523	0,179	0,065	0,511	-0,700	0,140	0,960	0,400	-0,963	0,357	-0,554	
A446E	Likely pathogenic		15,05	0,522	0,442	0,294	0,014	0,389	0,484	0,155	1,040	-0,250	-1,974	-0,253	-1,402	
A446G	Likely pathogenic		14,53	0,356	0,417	0,155	-0,080	0,174	1,439	0,155	0,290	-0,250	-3,102	-1,450	-2,150	
A446P	Likely pathogenic	Mild	8,94	0,646	0,426	0,453	0,141	0,696	0,708	0,155	0,520	-0,250	-1,299	-0,785	-0,811	
A446S	Likely pathogenic		0,42	0,316	0,415	0,088	-0,112	0,189	0,241	0,155	0,270	-0,250	-4,357	-1,910	-3,249	
A446T	Likely pathogenic		0,68	0,354	0,436	0,061	-0,107	0,177	-0,741	0,155	0,220	-0,250	-4,296	-1,938	-3,175	
A446V	Likely pathogenic		14,18	0,428	0,420	0,087	-0,105	0,180	-1,863	0,155	0,440	-0,250	-3,235	-1,244	-2,306	
V447A	Likely pathogenic		20,20	0,756	0,568	0,256	0,030	0,464	2,966	0,006	0,440	-0,420	-0,465	-1,077	0,197	
V447E	Likely pathogenic		22,60	0,779	0,559	0,873	0,175	0,933	2,779	0,006	1,480	-0,420	1,524	0,689	1,599	
V447G	Likely pathogenic		21,50	0,801	0,497	0,604	0,168	0,714	4,622	0,006	0,730	-0,420	0,719	-0,445	1,042	
V447L	Likely pathogenic		15,32	0,570	0,558	0,279	0,035	0,286	1,169	0,006	0,110	-0,420	-1,511	-1,749	-0,592	
V447M	Likely pathogenic		22,20	0,588	0,586	0,229	0,058	0,600	1,735	0,006	0,110	-0,420	-0,437	-1,437	0,305	
A448E	Likely pathogenic		25,80	0,927	0,657	0,960	0,247	0,926	5,156	0,008	1,040	-0,500	2,599	0,353	2,685	
A448G	Likely pathogenic		22,80	0,753	0,600	0,173	0,154	0,642	2,171	0,008	0,290	-0,500	0,226	-1,108	0,794	
A448P	Likely pathogenic		22,90	0,912	0,659	0,898	0,374	0,934	0,244	0,008	0,520	-0,500	2,380	-0,274	2,572	
A448S	Likely pathogenic		22,50	0,795	0,589	0,214	0,127	0,774	1,972	0,008	0,270	-0,500	0,427	-1,080	0,947	
A448T	Likely pathogenic		22,80	0,864	0,619	0,376	0,137	0,725	1,573	0,008	0,220	-0,500	0,761	-1,048	1,280	
A448V	Likely pathogenic		26,00	0,894	0,630	0,406	0,137	0,890	0,268	0,008	0,440	-0,500	1,311	-0,595	1,690	
L449M	VUS		22,10	0,493	0,499	0,133	0,117	0,461	0,257	0,000	0,000	-0,570	-1,035	-1,785	-0,224	
L449P	Likely pathogenic		25,80	0,860	0,592	0,897	0,376	0,886	7,096	0,000	1,070	-0,570	2,605	0,297	2,618	
L449Q	Likely pathogenic		25,50	0,771	0,514	0,703	0,313	0,860	4,155	0,000	1,190	-0,570	1,714	0,285	1,779	
L449R	Likely pathogenic		25,60	0,787	0,568	0,822	0,373	0,872	8,156	0,000	1,750	-0,570	2,378	1,040	2,252	
L449V	VUS		17,58	0,563	0,467	0,069	0,107	0,399	4,314	0,000	0,110	-0,570	-1,307	-1,833	-0,517	
M450I	VUS		8,55	0,367	0,389	0,099	-0,039	0,203	0,178	0,094	0,070	0,730	-3,672	-1,424	-2,708	
M450K	VUS		10,48	0,498	0,420	0,158	0,111	0,335	1,706	0,094	2,890	0,730	-2,320	2,287	-2,248	
M450L	VUS		3,39	0,602	0,377	0,062	-0,079	0,552	0,172	0,094	0,000	0,730	-3,237	-1,455	-2,413	
M450R	VUS		15,19	0,459	0,412	0,134	0,149	0,399	2,218	0,094	1,750	0,730	-1,983	1,034	-1,707	
M450T	VUS		9,55	0,371	0,445	0,114	0,027	0,271	2,014	0,094	0,770	0,730	-3,056	-0,466	-2,325	
M450V	VUS		5,98	0,398	0,358	0,067	-0,006	0,198	1,119	0,094	0,110	0,730	-3,788	-1,444	-2,862	
H451D	Likely pathogenic		14,08	0,556	0,381	0,270	0,076	0,392	3,534	0,098	0,580	0,550	-1,989	-0,501	-1,429	
H451L	Likely pathogenic		12,18	0,553	0,360	0,069	0,075	0,306	-0,558	0,098	0,720	0,550	-2,688	-0,447	-2,086	
H451N	Likely pathogenic		13,38	0,475	0,378	0,119	0,025	0,207	1,631	0,098	0,440	0,550	-2,852	-0,852	-2,101	
H451P	Likely pathogenic		15,26	0,604	0,370	0,224	0,204	0,403	1,126	0,098	0,350	0,550	-1,683	-0,649	-1,166	
H451Q	Likely pathogenic		15,87	0,552	0,355	0,136	-0,001	0,323	1,518	0,098	0,470	0,550	-2,458	-0,686	-1,801	
H451R	Likely pathogenic		5,77	0,546	0,372	0,059	0,036	0,642	0,719	0,098	1,030	0,550	-2,651	-0,165	-2,172	
H451Y	Likely pathogenic		12,83	0,482	0,379	0,099	0,004	0,302	4,645	0,098	0,380	0,550	-2,687	-0,947	-1,957	
P452A	Likely pathogenic		22,60	0,762	0,561	0,092	0,161	0,713	1,986	0,585	0,520	0,310	-0,771	0,811	-0,581	
P452H	Likely pathogenic		25,90	0,880	0,604	0,161	0,208	0,836	2,587	0,585	0,350	0,310	0,171	0,853	0,268	
P452L	Likely pathogenic		26,00	0,797	0,619	0,130	0,211	0,805	1,654	0,585	1,070	0,310	0,000	1,692	-0,024	Yes
P452R	Likely pathogenic		25,90	0,879	0,612	0,111	0,312	0,842	1,261	0,585	0,680	0,310	0,362	1,315	0,326	
P452S	Likely pathogenic		23,00	0,802	0,562	0,112	0,161	0,730	2,332	0,585	0,250	0,310	-0,624	0,520	-0,396	
P452T	Likely pathogenic		22,80	0,814	0,587	0,104	0,168	0,629	2,266	0,585	0,300	0,310	-0,695	0,560	-0,439	
D453A	Likely pathogenic		23,50	0,835	0,537	0,189	0,115	0,712	2,379	0,487	0,750	0,230	-0,447	0,896	-0,295	
D453E	VUS		0,39	0,599	0,556	0,254	0,044	0,648	1,561	0,487	0,290	0,230	-2,740	-0,454	-2,129	

D453G	Likely pathogenic		23,60	0,843	0,546	0,120	0,125	0,796	2,503	0,487	0,460	0,230	-0,347	0,565	-0,158	
D453H	Likely pathogenic		22,00	0,862	0,595	0,249	0,204	0,852	2,167	0,487	0,580	0,230	0,105	0,800	0,215	
D453N	Likely pathogenic		22,00	0,761	0,577	0,093	0,074	0,685	0,826	0,487	0,140	0,230	-0,952	0,042	-0,545	
D453V	Likely pathogenic		23,70	0,856	0,533	0,331	0,242	0,866	1,927	0,487	1,190	0,230	0,304	1,616	0,195	
D453Y	Likely pathogenic		22,10	0,853	0,582	0,272	0,172	0,846	1,364	0,487	0,960	0,230	-0,002	1,251	0,038	
G454A	Likely pathogenic		22,60	0,737	0,544	0,197	0,105	0,569	2,999	0,221	0,290	-0,120	-0,484	-0,540	0,009	
G454C	Likely pathogenic		23,80	0,873	0,573	0,304	0,247	0,848	2,942	0,221	0,750	-0,120	0,836	0,340	0,970	
G454D	Likely pathogenic		23,80	0,809	0,628	0,141	0,160	0,721	3,044	0,221	0,460	-0,120	0,248	-0,163	0,612	
G454R	Likely pathogenic		23,40	0,827	0,618	0,182	0,183	0,771	2,668	0,221	0,910	-0,120	0,423	0,425	0,636	
G454S	Likely pathogenic		22,30	0,749	0,529	0,107	0,104	0,661	3,044	0,221	0,020	-0,120	-0,501	-0,874	0,021	
G454V	Likely pathogenic		24,30	0,884	0,551	0,418	0,274	0,856	4,276	0,221	0,730	-0,120	1,094	0,366	1,181	
S455A	VUS		15,42	0,536	0,500	0,089	-0,117	0,301	-0,502	0,032	0,270	-0,520	-2,335	-1,715	-1,359	
S455C	Likely pathogenic		21,60	0,761	0,518	0,134	-0,041	0,503	-0,249	0,032	0,730	-0,520	-0,851	-0,740	-0,241	
S455F	Likely pathogenic		22,20	0,844	0,541	0,175	0,062	0,642	-0,241	0,032	0,850	-0,520	-0,086	-0,410	0,350	
S455P	Likely pathogenic		22,70	0,778	0,524	0,488	0,138	0,738	-0,064	0,032	0,250	-0,520	0,417	-1,005	0,910	
S455T	VUS		16,62	0,640	0,513	0,096	-0,120	0,375	-0,026	0,032	0,050	-0,520	-1,907	-1,872	-0,950	
S455Y	Likely pathogenic		18,80	0,817	0,560	0,153	-0,017	0,638	0,205	0,032	0,480	-0,520	-0,561	-1,018	0,060	
A456D	Likely pathogenic		19,74	0,772	0,521	0,576	0,154	0,787	3,737	0,000	0,750	-0,800	0,692	-0,689	1,065	
A456G	VUS		18,01	0,521	0,434	0,180	-0,012	0,394	1,424	0,000	0,290	-0,800	-1,664	-1,780	-0,820	
A456P	Likely pathogenic		11,66	0,672	0,500	0,430	0,194	0,712	3,292	0,000	0,520	-0,800	-0,331	-1,295	0,206	Yes
A456S	Likely pathogenic		9,25	0,486	0,420	0,127	-0,039	0,369	1,984	0,000	0,270	-0,800	-2,520	-2,114	-1,569	
A456T	VUS		9,42	0,483	0,443	0,091	-0,055	0,328	2,266	0,000	0,220	-0,800	-2,591	-2,198	-1,591	
A456V	VUS		16,07	0,514	0,460	0,094	-0,063	0,282	0,291	0,000	0,440	-0,800	-2,173	-1,725	-1,251	
V457A	VUS		22,90	0,765	0,599	0,140	0,145	0,619	3,300	0,000	0,440	-0,990	0,329	-1,246	0,903	
V457D	VUS		24,60	0,920	0,602	0,788	0,408	0,926	4,972	0,000	1,190	-0,990	2,678	0,219	2,668	
V457F	VUS		22,40	0,923	0,563	0,351	0,318	0,928	7,213	0,000	0,140	-0,990	1,757	-1,334	2,074	
V457G	VUS		24,40	0,906	0,492	0,365	0,299	0,756	4,941	0,000	0,730	-0,990	1,360	-0,641	1,580	
V457I	VUS		21,90	0,687	0,459	0,094	0,090	0,448	0,737	0,000	0,040	-0,990	-0,796	-1,939	-0,034	
V457L	VUS		22,20	0,813	0,579	0,291	0,166	0,754	0,671	0,000	0,110	-0,990	0,609	-1,531	1,183	
V458A	VUS		15,95	0,589	0,463	0,220	0,083	0,529	3,860	0,000	0,440	-1,140	-0,961	-1,715	-0,250	
V458E	VUS		21,10	0,719	0,451	0,906	0,296	0,853	4,881	0,000	1,480	-1,140	1,495	0,178	1,553	
V458G	VUS		21,10	0,723	0,389	0,522	0,298	0,715	5,813	0,000	0,730	-1,140	0,711	-0,928	0,994	
V458L	VUS		8,85	0,522	0,443	0,128	0,068	0,310	-0,598	0,000	0,110	-1,140	-2,305	-2,430	-1,321	
V458M	VUS		21,10	0,558	0,475	0,163	0,099	0,585	-0,311	0,000	0,110	-1,140	-0,764	-1,940	0,001	
V459A	VUS		24,10	0,848	0,567	0,494	0,159	0,790	2,569	0,000	0,440	-1,180	1,182	-1,120	1,622	
V459D	VUS		24,60	0,893	0,567	0,969	0,402	0,924	3,545	0,000	1,190	-1,180	2,705	0,141	2,704	
V459F	VUS		22,50	0,855	0,530	0,633	0,244	0,885	8,492	0,000	0,140	-1,180	1,715	-1,473	2,092	
V459G	VUS		24,50	0,894	0,474	0,759	0,293	0,854	4,524	0,000	0,730	-1,180	1,889	-0,613	2,062	
V459I	VUS		19,08	0,580	0,438	0,093	0,068	0,427	-0,977	0,000	0,040	-1,180	-1,364	-2,194	-0,503	
V459L	VUS		22,20	0,815	0,546	0,492	0,153	0,745	2,442	0,000	0,110	-1,180	0,822	-1,620	1,382	
V460A	Likely pathogenic		24,70	0,856	0,685	0,440	0,084	0,755	3,468	0,000	0,440	-1,070	1,241	-1,065	1,775	
V460E	Likely pathogenic		25,80	0,944	0,671	0,950	0,298	0,910	5,071	0,000	1,480	-1,070	2,905	0,580	2,904	
V460G	Likely pathogenic		25,70	0,930	0,573	0,670	0,300	0,764	5,722	0,000	0,730	-1,070	2,088	-0,521	2,301	
V460L	Likely pathogenic		16,86	0,732	0,666	0,201	0,073	0,802	-1,562	0,000	0,110	-1,070	-0,041	-1,773	0,709	
V460M	Likely pathogenic		22,50	0,818	0,697	0,279	0,106	0,886	-0,586	0,000	0,110	-1,070	0,924	-1,500	1,547	
L461I	Likely pathogenic		22,60	0,676	0,693	0,180	0,046	0,506	1,559	0,000	0,070	-0,800	-0,140	-1,671	0,698	
L461P	Likely pathogenic		25,90	0,907	0,767	0,979	0,342	0,945	7,513	0,000	1,070	-0,800	3,276	0,283	3,354	
L461Q	Likely pathogenic		25,60	0,890	0,700	0,418	0,197	0,919	3,243	0,000	1,190	-0,800	1,890	0,170	2,081	
L461R	Likely pathogenic		25,90	0,908	0,750	0,639	0,338	0,931	7,656	0,000	1,750	-0,800	2,887	1,001	2,822	
L461V	Likely pathogenic		22,40	0,715	0,659	0,171	0,077	0,621	1,074	0,000	0,110	-0,800	0,061	-1,564	0,802	
N462D	Likely pathogenic		23,90	0,909	0,735	0,894	0,315	0,943	2,202	0,000	0,140	-0,310	2,515	-0,635	2,838	
N462H	Likely pathogenic		23,70	0,913	0,709	0,644	0,317	0,936	26,778	0,000	0,440	-0,310	3,197	-0,559	3,318	
N462I	Likely pathogenic		25,40	0,925	0,709	0,916	0,362	0,936	4,913	0,000	1,090	-0,310	2,897	0,573	2,916	
N462K	Likely pathogenic	Severe	23,90	0,891	0,753	0,965	0,315	0,932	5,581	0,000	1,730	-0,310	2,844	1,296	2,775	

N462S	Likely pathogenic		24,30	0,903	0,661	0,327	0,202	0,906	3,360	0,000	0,340	-0,310	1,468	-0,660	1,826	
N462T	Likely pathogenic		24,90	0,916	0,695	0,844	0,331	0,928	2,190	0,000	0,390	-0,310	2,483	-0,325	2,716	
N462Y	Likely pathogenic		24,10	0,928	0,736	0,853	0,352	0,940	14,430	0,000	0,820	-0,310	3,160	0,118	3,222	
R463C	Pathogenic	Severe	27,60	0,817	0,599	0,126	0,212	0,944	0,600	0,365	1,660	0,760	0,521	2,326	0,330	Yes
R463G	Likely pathogenic		23,70	0,684	0,511	0,115	0,166	0,811	1,492	0,365	0,910	0,760	-0,557	1,117	-0,449	
R463H	Pathogenic	Severe	23,30	0,840	0,530	0,077	0,173	0,957	1,948	0,365	1,030	0,760	-0,024	1,377	-0,052	Yes
R463L	Likely pathogenic		20,40	0,735	0,544	0,179	0,128	0,929	-0,453	0,365	1,750	0,760	-0,476	2,145	-0,565	
R463P	Likely pathogenic		22,20	0,745	0,551	0,258	0,264	0,932	-1,005	0,365	0,680	0,760	0,002	0,998	0,072	
R463S	Likely pathogenic		22,50	0,652	0,514	0,224	0,115	0,617	1,375	0,365	0,930	0,760	-0,987	1,032	-0,765	
S464A	VUS		21,70	0,728	0,541	0,097	-0,087	0,274	0,525	0,155	0,270	0,570	-1,730	-0,546	-1,002	
S464C	VUS		24,60	0,814	0,558	0,149	-0,025	0,565	0,820	0,155	0,730	0,570	-0,635	0,293	-0,217	
S464F	VUS		24,20	0,836	0,585	0,132	-0,005	0,437	0,634	0,155	0,850	0,570	-0,719	0,414	-0,287	
S464P	VUS		23,60	0,823	0,561	0,576	0,089	0,642	1,006	0,155	0,250	0,570	0,148	-0,106	0,560	
S464T	VUS		21,50	0,721	0,548	0,099	-0,092	0,322	0,670	0,155	0,050	0,570	-1,690	-0,808	-0,916	
S464Y	VUS		23,70	0,830	0,608	0,130	0,000	0,430	0,839	0,155	0,480	0,570	-0,730	-0,048	-0,194	
S465A	VUS		18,65	0,402	0,518	0,071	-0,102	0,240	-0,072	0,535	0,270	0,520	-3,270	-0,075	-2,515	
S465C	VUS		24,10	0,714	0,540	0,133	0,035	0,469	0,182	0,535	0,730	0,520	-1,459	0,972	-1,144	
S465F	VUS		24,10	0,698	0,565	0,125	-0,020	0,455	-0,238	0,535	0,850	0,520	-1,600	1,087	-1,256	
S465P	VUS		20,20	0,557	0,542	0,066	0,042	0,267	-0,851	0,535	0,250	0,520	-2,466	0,123	-1,863	
S465T	VUS		18,04	0,518	0,536	0,094	-0,110	0,242	0,960	0,535	0,050	0,520	-3,018	-0,303	-2,246	
S465Y	VUS		22,60	0,654	0,590	0,115	-0,010	0,394	-0,239	0,535	0,480	0,520	-1,837	0,562	-1,347	
K466E	VUS		18,88	0,367	0,482	0,064	-0,084	0,209	0,656	0,619	1,300	0,470	-3,416	1,291	-2,948	
K466M	VUS		22,40	0,609	0,436	0,143	-0,081	0,308	0,033	0,619	2,890	0,470	-2,483	3,497	-2,583	
K466N	VUS		16,84	0,242	0,513	0,172	-0,084	0,204	0,770	0,619	1,730	0,470	-3,576	1,739	-3,141	
K466Q	VUS		18,52	0,448	0,444	0,081	-0,109	0,186	0,059	0,619	1,700	0,470	-3,449	1,782	-3,096	
K466R	VUS		19,09	0,327	0,437	0,068	-0,171	0,245	-0,179	0,619	1,140	0,470	-3,781	1,035	-3,234	
K466T	VUS		18,90	0,483	0,491	0,087	-0,082	0,189	0,673	0,619	2,120	0,470	-3,109	2,356	-2,877	
D467A	VUS		22,70	0,610	0,535	0,120	-0,032	0,305	0,579	0,503	0,750	0,210	-2,063	0,557	-1,570	
D467E	VUS		15,93	0,558	0,554	0,161	-0,090	0,177	-0,111	0,503	0,290	0,210	-2,939	-0,270	-2,168	
D467G	VUS		23,90	0,633	0,535	0,153	0,000	0,531	1,109	0,503	0,460	0,210	-1,484	0,347	-1,046	
D467H	VUS		25,50	0,714	0,602	0,260	0,003	0,570	0,773	0,503	0,580	0,210	-0,878	0,655	-0,505	
D467N	VUS		22,40	0,476	0,577	0,102	-0,067	0,313	0,000	0,503	0,140	0,210	-2,399	-0,263	-1,661	
D467V	VUS		22,90	0,654	0,529	0,172	0,016	0,457	0,813	0,503	1,190	0,210	-1,539	1,211	-1,263	
D467Y	VUS		25,60	0,731	0,593	0,192	0,039	0,676	0,628	0,503	0,960	0,210	-0,671	1,164	-0,458	
V468A	VUS		23,30	0,716	0,613	0,166	0,004	0,336	2,564	0,172	0,440	-0,020	-0,895	-0,502	-0,243	
V468E	VUS		22,50	0,662	0,596	0,179	0,128	0,341	2,553	0,172	1,480	-0,020	-0,699	0,788	-0,359	
V468G	VUS		25,30	0,751	0,521	0,219	0,147	0,509	4,059	0,172	0,730	-0,020	-0,165	0,029	0,184	
V468L	VUS		19,57	0,645	0,591	0,137	-0,001	0,275	0,441	0,172	0,110	-0,020	-1,591	-1,068	-0,773	
V468M	VUS		23,40	0,597	0,623	0,103	0,013	0,410	0,121	0,172	0,110	-0,020	-1,154	-0,925	-0,387	
P469A	VUS		21,40	0,659	0,501	0,068	-0,062	0,227	1,825	0,547	0,520	-0,030	-2,367	0,134	-1,796	
P469H	VUS		22,30	0,726	0,549	0,094	-0,013	0,309	1,671	0,547	0,350	-0,030	-1,813	0,067	-1,274	
P469L	VUS		22,30	0,686	0,567	0,072	-0,010	0,256	1,144	0,547	1,070	-0,030	-1,910	0,917	-1,501	
P469R	VUS		22,30	0,721	0,561	0,082	0,009	0,273	1,024	0,547	0,680	-0,030	-1,809	0,475	-1,339	
P469S	VUS		21,80	0,699	0,496	0,080	-0,071	0,204	1,967	0,547	0,250	-0,030	-2,330	-0,178	-1,702	
P469T	VUS		21,30	0,692	0,530	0,081	-0,062	0,206	1,837	0,547	0,300	-0,030	-2,274	-0,113	-1,644	
L470F	VUS		15,50	0,442	0,456	0,070	0,041	0,166	7,459	0,010	0,030	0,020	-2,222	-1,833	-1,293	
L470H	VUS		25,00	0,763	0,523	0,593	0,255	0,675	5,291	0,010	0,720	0,020	1,029	-0,079	1,278	
L470I	VUS		16,01	0,448	0,464	0,077	-0,011	0,195	0,355	0,010	0,070	0,020	-2,520	-1,723	-1,544	
L470P	VUS		25,80	0,811	0,555	0,898	0,296	0,820	7,154	0,010	1,070	0,020	2,007	0,553	2,049	
L470R	VUS		25,70	0,795	0,534	0,712	0,225	0,807	6,299	0,010	1,750	0,020	1,538	1,275	1,479	
L470V	VUS		15,82	0,452	0,441	0,069	0,023	0,166	1,333	0,010	0,110	0,020	-2,505	-1,687	-1,564	
T471A	VUS		21,90	0,636	0,542	0,081	-0,080	0,214	2,430	0,023	0,220	-0,160	-1,580	-1,375	-0,693	
T471I	VUS		23,60	0,719	0,642	0,165	-0,050	0,392	0,129	0,023	0,700	-0,160	-0,693	-0,538	-0,001	
T471N	VUS		21,70	0,514	0,616	0,173	-0,022	0,225	3,225	0,023	0,390	-0,160	-1,348	-1,147	-0,472	

T471P	VUS	24,00	0,758	0,555	0,600	0,174	0,576	4,718	0,023	0,300	-0,160	0,652	-0,758	1,123
T471S	VUS	21,30	0,606	0,547	0,112	-0,123	0,196	2,346	0,023	0,050	-0,160	-1,778	-1,632	-0,800
I472F	VUS	24,60	0,775	0,649	0,329	0,158	0,784	3,696	0,000	0,100	-0,340	0,932	-1,099	1,462
I472L	VUS	22,90	0,690	0,640	0,119	0,061	0,382	-0,004	0,000	0,070	-0,340	-0,588	-1,450	0,234
I472M	VUS	22,80	0,704	0,621	0,182	0,090	0,635	-0,273	0,000	0,070	-0,340	-0,109	-1,331	0,586
I472N	VUS	25,90	0,820	0,704	0,977	0,312	0,929	3,747	0,000	1,090	-0,340	2,616	0,513	2,708
I472S	VUS	26,00	0,876	0,651	0,961	0,242	0,865	5,991	0,000	0,750	-0,340	2,392	0,023	2,576
I472T	VUS	25,10	0,822	0,716	0,946	0,232	0,837	4,073	0,000	0,700	-0,340	2,214	-0,067	2,495
I472V	VUS	21,30	0,669	0,583	0,103	0,017	0,317	1,460	0,000	0,040	-0,340	-1,036	-1,627	-0,167
K473E	VUS	10,44	0,424	0,417	0,057	-0,090	0,187	0,623	0,301	1,300	-0,250	-3,534	-0,020	-2,863
K473M	VUS	19,25	0,528	0,399	0,136	-0,086	0,362	-0,593	0,301	2,890	-0,250	-2,333	2,308	-2,232
K473N	VUS	15,40	0,457	0,437	0,188	-0,096	0,204	0,516	0,301	1,730	-0,250	-2,892	0,709	-2,381
K473Q	VUS	11,81	0,412	0,397	0,064	-0,115	0,207	0,889	0,301	1,700	-0,250	-3,489	0,486	-2,925
K473R	VUS	16,23	0,442	0,402	0,066	-0,183	0,250	-0,900	0,301	1,140	-0,250	-3,321	-0,067	-2,628
K473T	VUS	16,24	0,474	0,423	0,088	-0,088	0,210	2,254	0,301	2,120	-0,250	-2,814	1,175	-2,426
D474A	Likely pathogenic	24,40	0,837	0,607	0,759	0,171	0,725	4,587	0,000	0,750	-0,120	1,396	-0,065	1,690
D474E	Likely pathogenic	23,00	0,712	0,624	0,763	0,115	0,625	5,361	0,000	0,290	-0,120	0,821	-0,791	1,359
D474G	Likely pathogenic	26,20	0,856	0,610	0,836	0,188	0,775	6,380	0,000	0,460	-0,120	1,820	-0,325	2,119
D474H	Likely pathogenic	24,10	0,828	0,661	0,876	0,192	0,748	6,435	0,000	0,580	-0,120	1,764	-0,226	2,088
D474N	Likely pathogenic	24,10	0,760	0,644	0,722	0,134	0,725	5,348	0,000	0,140	-0,120	1,174	-0,880	1,686
D474V	Likely pathogenic	24,30	0,817	0,599	0,715	0,219	0,817	3,668	0,000	1,190	-0,120	1,534	0,510	1,670
D474Y	Likely pathogenic	24,10	0,851	0,652	0,711	0,222	0,767	5,165	0,000	0,960	-0,120	1,682	0,232	1,893
P475A	VUS	22,70	0,656	0,561	0,070	-0,026	0,269	3,005	0,503	0,520	0,050	-1,893	0,174	-1,339
P475H	VUS	26,80	0,809	0,605	0,112	0,021	0,626	2,263	0,503	0,350	0,050	-0,564	0,340	-0,199
P475L	VUS	23,10	0,692	0,624	0,090	0,026	0,516	1,714	0,503	1,070	0,050	-1,146	1,031	-0,832
P475R	VUS	24,30	0,762	0,618	0,098	0,048	0,533	1,797	0,503	0,680	0,050	-0,881	0,639	-0,532
P475S	VUS	23,00	0,682	0,558	0,087	-0,029	0,262	3,079	0,503	0,250	0,050	-1,848	-0,136	-1,240
P475T	VUS	22,90	0,670	0,588	0,079	-0,013	0,389	3,096	0,503	0,300	0,050	-1,582	-0,021	-1,023
A476D	VUS	14,03	0,599	0,561	0,081	0,021	0,212	-0,027	0,457	0,750	0,020	-2,613	0,109	-1,997
A476G	VUS	13,52	0,494	0,465	0,091	-0,068	0,201	1,014	0,457	0,290	0,020	-3,286	-0,616	-2,503
A476P	VUS	20,20	0,706	0,521	0,203	0,073	0,374	2,764	0,457	0,520	0,020	-1,470	0,133	-1,021
A476S	VUS	14,00	0,405	0,450	0,079	-0,101	0,201	0,332	0,457	0,270	0,020	-3,579	-0,693	-2,742
A476T	VUS	14,94	0,524	0,470	0,073	-0,095	0,209	0,061	0,457	0,220	0,020	-3,232	-0,654	-2,433
A476V	VUS	14,96	0,551	0,496	0,084	-0,085	0,334	-0,618	0,457	0,440	0,020	-2,908	-0,304	-2,210
V477A	VUS	21,00	0,530	0,422	0,076	-0,012	0,182	2,345	0,184	0,440	-0,050	-2,248	-0,814	-1,518
V477E	VUS	21,60	0,623	0,409	0,151	0,120	0,303	2,649	0,184	1,480	-0,050	-1,390	0,648	-1,092
V477G	VUS	21,50	0,678	0,375	0,119	0,108	0,294	3,560	0,184	0,730	-0,050	-1,468	-0,284	-1,015
V477L	VUS	7,70	0,421	0,397	0,097	-0,025	0,189	1,037	0,184	0,110	-0,050	-3,529	-1,640	-2,571
V477M	VUS	10,34	0,432	0,413	0,092	-0,005	0,140	0,328	0,184	0,110	-0,050	-3,343	-1,556	-2,393
G478A	Likely pathogenic	22,80	0,860	0,625	0,354	0,154	0,627	3,748	0,106	0,290	-0,170	0,533	-0,619	0,992
G478C	Likely pathogenic	24,00	0,891	0,662	0,581	0,231	0,785	5,621	0,106	0,750	-0,170	1,546	0,153	1,757
G478D	Likely pathogenic	23,00	0,868	0,699	0,604	0,216	0,740	8,807	0,106	0,460	-0,170	1,551	-0,269	1,872
G478R	Likely pathogenic	23,70	0,905	0,692	0,647	0,230	0,812	8,049	0,106	0,910	-0,170	1,847	0,368	2,000
G478S	Likely pathogenic	22,60	0,843	0,617	0,257	0,159	0,713	6,523	0,106	0,020	-0,170	0,596	-0,985	1,082
G478V	Likely pathogenic	24,10	0,874	0,629	0,678	0,253	0,821	10,947	0,106	0,730	-0,170	1,871	0,118	2,015
F479C	VUS	24,20	0,800	0,733	0,283	0,051	0,684	2,787	0,121	0,120	-0,210	0,467	-0,817	1,078
F479I	VUS	21,10	0,723	0,707	0,196	0,013	0,510	2,916	0,121	0,100	-0,210	-0,402	-1,082	0,349
F479L	VUS	21,70	0,746	0,734	0,796	-0,031	0,474	2,288	0,121	0,030	-0,210	0,222	-0,987	0,990
F479S	VUS	22,50	0,713	0,714	0,307	0,045	0,433	4,480	0,121	0,850	-0,210	-0,100	-0,118	0,456
F479V	VUS	21,10	0,742	0,695	0,210	0,004	0,488	2,444	0,121	0,140	-0,210	-0,449	-1,033	0,295
F479Y	VUS	21,50	0,671	0,727	0,120	-0,054	0,276	0,199	0,121	0,370	-0,210	-1,108	-0,875	-0,250
L480M	VUS	20,30	0,430	0,468	0,136	0,011	0,302	0,039	0,000	0,000	-0,050	-1,955	-1,688	-1,032
L480P	VUS	24,30	0,778	0,564	0,687	0,215	0,711	5,674	0,000	1,070	-0,050	1,235	0,307	1,428
L480Q	VUS	24,20	0,739	0,494	0,445	0,152	0,508	4,101	0,000	1,190	-0,050	0,211	0,242	0,499

L480R	VUS	24,30	0,756	0,532	0,489	0,200	0,563	7,440	0,000	1,750	-0,050	0,766	0,984	0,855
L480V	VUS	18,61	0,448	0,450	0,069	0,016	0,201	4,368	0,000	0,110	-0,050	-2,109	-1,686	-1,201
E481A	VUS	17,98	0,474	0,568	0,148	-0,109	0,204	1,848	0,345	1,040	0,330	-2,616	0,394	-1,962
E481D	VUS	12,66	0,508	0,569	0,149	-0,144	0,223	0,921	0,345	0,290	0,330	-3,070	-0,658	-2,185
E481G	VUS	20,50	0,580	0,537	0,127	-0,068	0,330	1,944	0,345	0,750	0,330	-2,065	0,209	-1,486
E481K	VUS	14,12	0,548	0,630	0,102	-0,089	0,178	1,714	0,345	1,300	0,330	-2,627	0,657	-2,007
E481Q	VUS	13,16	0,442	0,558	0,089	-0,139	0,146	0,905	0,345	0,400	0,330	-3,349	-0,587	-2,444
E481V	VUS	19,67	0,540	0,544	0,271	-0,092	0,375	1,613	0,345	1,480	0,330	-1,973	1,108	-1,555
T482A	VUS	15,21	0,588	0,563	0,079	-0,088	0,322	0,688	0,157	0,220	-0,460	-2,147	-1,430	-1,234
T482I	VUS	21,60	0,696	0,652	0,233	-0,053	0,491	0,824	0,157	0,700	-0,460	-0,733	-0,456	-0,086
T482K	VUS	22,90	0,764	0,630	0,368	0,014	0,641	3,242	0,157	2,120	-0,460	0,173	1,448	0,311
T482P	VUS	21,50	0,733	0,571	0,399	0,176	0,687	5,519	0,157	0,300	-0,460	0,325	-0,760	0,771
T482R	VUS	23,00	0,749	0,617	0,286	0,025	0,652	2,709	0,157	0,980	-0,460	-0,036	0,035	0,377
T482S	VUS	15,25	0,638	0,565	0,108	-0,119	0,305	0,065	0,157	0,050	-0,460	-2,150	-1,621	-1,188
I483F	VUS	14,71	0,375	0,374	0,083	0,004	0,239	0,491	0,371	0,100	-0,400	-3,256	-1,276	-2,421
I483L	VUS	12,83	0,423	0,364	0,070	-0,092	0,203	-0,252	0,371	0,070	-0,400	-3,654	-1,409	-2,742
I483M	VUS	12,91	0,391	0,351	0,079	-0,078	0,235	-0,063	0,371	0,070	-0,400	-3,641	-1,408	-2,744
I483N	VUS	17,18	0,426	0,407	0,111	0,078	0,393	1,782	0,371	1,090	-0,400	-2,344	0,132	-1,885
I483S	VUS	15,94	0,454	0,378	0,105	0,064	0,291	2,112	0,371	0,750	-0,400	-2,650	-0,363	-2,078
I483T	VUS	14,70	0,493	0,428	0,087	-0,025	0,293	1,065	0,371	0,700	-0,400	-2,820	-0,464	-2,158
I483V	VUS	10,31	0,325	0,326	0,059	-0,138	0,225	0,624	0,371	0,040	-0,400	-4,163	-1,607	-3,187
S484A	VUS	15,10	0,377	0,426	0,076	-0,097	0,205	-1,038	0,000	0,270	-0,480	-2,930	-1,896	-1,887
S484L	VUS	19,13	0,505	0,432	0,136	-0,014	0,385	-1,231	0,000	0,820	-0,480	-1,822	-0,919	-1,111
S484P	VUS	18,23	0,533	0,440	0,534	0,081	0,344	4,255	0,000	0,250	-0,480	-1,023	-1,516	-0,276
S484T	VUS	15,16	0,441	0,428	0,127	-0,086	0,277	0,191	0,000	0,050	-0,480	-2,575	-2,095	-1,547
P485A	VUS	23,70	0,879	0,521	0,096	0,082	0,613	2,973	0,145	0,520	-0,550	-0,086	-0,598	0,343
P485H	Likely pathogenic	27,90	0,935	0,570	0,185	0,208	0,812	2,609	0,145	0,350	-0,550	1,102	-0,476	1,384
P485L	VUS	28,00	0,881	0,586	0,131	0,137	0,801	2,143	0,145	1,070	-0,550	0,834	0,333	1,023
P485R	VUS	27,90	0,907	0,582	0,111	0,159	0,866	2,489	0,145	0,680	-0,550	0,979	-0,110	1,213
P485S	VUS	24,20	0,876	0,528	0,136	0,084	0,662	2,724	0,145	0,250	-0,550	0,052	-0,882	0,526
P485T	VUS	23,90	0,888	0,559	0,121	0,093	0,660	3,556	0,145	0,300	-0,550	0,169	-0,817	0,633
G486A	VUS	18,28	0,411	0,418	0,070	0,066	0,169	1,569	0,163	0,290	-0,430	-2,454	-1,352	-1,624
G486C	VUS	23,60	0,690	0,444	0,239	0,232	0,453	2,375	0,163	0,750	-0,430	-0,412	-0,262	-0,055
G486D	VUS	22,70	0,651	0,508	0,344	0,159	0,280	3,750	0,163	0,460	-0,430	-0,680	-0,716	-0,110
G486R	VUS	23,20	0,686	0,482	0,244	0,173	0,401	3,137	0,163	0,910	-0,430	-0,529	-0,120	-0,140
G486S	VUS	19,75	0,423	0,392	0,081	0,088	0,228	2,779	0,163	0,020	-0,430	-2,207	-1,620	-1,382
G486V	VUS	22,70	0,693	0,423	0,293	0,259	0,344	8,116	0,163	0,730	-0,430	-0,325	-0,374	0,020
Y487C	VUS	23,60	0,750	0,487	0,104	0,153	0,573	0,486	0,194	0,250	0,060	-0,609	-0,507	-0,159
Y487D	VUS	21,20	0,626	0,483	0,091	0,105	0,258	1,131	0,194	0,960	0,060	-1,544	0,088	-1,059
Y487F	VUS	22,50	0,585	0,436	0,114	-0,056	0,321	-0,555	0,194	0,370	0,060	-2,021	-0,683	-1,323
Y487H	VUS	21,90	0,414	0,482	0,086	-0,011	0,186	0,669	0,194	0,380	0,060	-2,355	-0,793	-1,551
Y487N	VUS	20,80	0,459	0,482	0,064	0,080	0,202	0,560	0,194	0,820	0,060	-2,101	-0,214	-1,473
Y487S	VUS	22,60	0,712	0,435	0,091	0,098	0,298	1,132	0,194	0,480	0,060	-1,387	-0,427	-0,860
S488A	VUS	24,30	0,835	0,514	0,182	0,151	0,564	0,737	0,000	0,270	-0,640	0,150	-1,195	0,692
S488C	VUS	29,60	0,924	0,534	0,436	0,285	0,704	-0,004	0,000	0,730	-0,640	1,555	-0,231	1,771
S488F	Likely pathogenic	31,00	0,953	0,558	0,961	0,313	0,871	8,667	0,000	0,850	-0,640	3,025	0,130	3,036
S488P	Likely pathogenic	27,40	0,945	0,531	0,984	0,315	0,936	7,070	0,000	0,250	-0,640	2,712	-0,675	2,871
S488T	VUS	23,90	0,873	0,524	0,398	0,140	0,542	-0,206	0,000	0,050	-0,640	0,342	-1,396	0,935
S488Y	Likely pathogenic	28,70	0,939	0,579	0,956	0,317	0,889	9,813	0,000	0,480	-0,640	2,939	-0,387	3,058
I489F	VUS	27,00	0,875	0,657	0,558	0,167	0,703	7,725	0,000	0,100	-1,020	1,764	-1,363	2,275
I489L	VUS	23,20	0,767	0,652	0,226	0,085	0,357	0,492	0,000	0,070	-1,020	-0,096	-1,760	0,732
I489M	VUS	23,60	0,712	0,621	0,271	0,101	0,584	1,171	0,000	0,070	-1,020	0,208	-1,695	0,941
I489N	VUS	26,90	0,850	0,730	0,971	0,334	0,848	4,339	0,000	1,090	-1,020	2,891	0,151	3,038
I489S	VUS	27,10	0,877	0,668	0,923	0,337	0,785	4,228	0,000	0,750	-1,020	2,641	-0,304	2,856

I489T	VUS		25,90	0,824	0,742	0,922	0,254	0,746	3,106	0,000	0,700	-1,020	2,325	-0,444	2,686	
I489V	VUS		22,80	0,702	0,576	0,166	0,054	0,443	0,646	0,000	0,040	-1,020	-0,436	-1,877	0,386	
H490D	VUS		23,40	0,742	0,555	0,492	0,090	0,633	3,182	0,000	0,580	-0,810	0,462	-0,910	0,980	
H490L	VUS		22,60	0,590	0,527	0,237	-0,005	0,290	-1,240	0,000	0,720	-0,810	-1,139	-1,053	-0,376	
H490N	VUS		21,90	0,497	0,552	0,130	-0,043	0,362	0,741	0,000	0,440	-0,810	-1,350	-1,495	-0,473	
H490P	VUS		24,20	0,801	0,506	0,875	0,206	0,636	2,976	0,000	0,350	-0,810	1,197	-0,981	1,622	
H490Q	VUS		15,00	0,501	0,532	0,198	-0,093	0,163	0,167	0,000	0,470	-0,810	-2,238	-1,728	-1,223	
H490R	VUS		22,40	0,656	0,559	0,326	-0,001	0,451	3,686	0,000	1,030	-0,810	-0,380	-0,592	0,202	
H490Y	VUS		23,00	0,655	0,560	0,236	-0,058	0,442	1,003	0,000	0,380	-0,810	-0,747	-1,409	0,050	
T491A	Likely pathogenic		24,00	0,839	0,552	0,691	0,294	0,703	2,130	0,000	0,220	-0,900	1,447	-1,135	1,855	
T491I	Likely pathogenic	Severe	25,90	0,913	0,668	0,970	0,320	0,901	3,198	0,000	0,700	-0,900	2,739	-0,259	2,923	
T491N	Likely pathogenic		25,30	0,910	0,628	0,875	0,350	0,856	1,020	0,000	0,390	-0,900	2,384	-0,680	2,650	
T491P	Likely pathogenic		24,40	0,856	0,552	0,936	0,459	0,819	9,114	0,000	0,300	-0,900	2,636	-0,875	2,815	
T491S	Likely pathogenic		23,60	0,779	0,561	0,302	0,170	0,657	2,074	0,000	0,050	-0,900	0,515	-1,576	1,116	
Y492C	VUS		27,70	0,752	0,662	0,467	0,213	0,654	5,790	0,000	0,250	-0,800	1,418	-1,093	1,928	
Y492D	VUS		24,90	0,719	0,653	0,970	0,256	0,770	6,411	0,000	0,960	-0,800	2,047	-0,119	2,307	
Y492F	VUS		22,40	0,685	0,581	0,091	-0,014	0,384	1,313	0,000	0,370	-0,800	-0,812	-1,449	-0,009	
Y492H	VUS		24,80	0,716	0,664	0,799	0,150	0,690	5,371	0,000	0,380	-0,800	1,413	-0,955	1,933	
Y492N	VUS		24,90	0,747	0,658	0,921	0,235	0,796	5,657	0,000	0,820	-0,800	2,006	-0,285	2,302	
Y492S	VUS		27,00	0,757	0,601	0,839	0,247	0,778	7,289	0,000	0,480	-0,800	1,989	-0,697	2,319	
L493M	VUS		22,60	0,584	0,493	0,172	0,064	0,525	0,364	0,005	0,000	-0,520	-0,842	-1,687	-0,073	
L493P	VUS		24,70	0,871	0,611	0,891	0,323	0,873	6,153	0,005	1,070	-0,520	2,384	0,291	2,447	
L493Q	VUS		24,50	0,817	0,508	0,167	0,192	0,828	2,227	0,005	1,190	-0,520	0,691	0,100	0,873	
L493R	VUS		24,60	0,837	0,577	0,137	0,319	0,849	3,825	0,005	1,750	-0,520	1,311	0,881	1,284	
L493V	VUS		16,64	0,633	0,460	0,084	0,054	0,208	2,091	0,005	0,110	-0,520	-1,761	-1,861	-0,886	
W494C	VUS		27,90	0,838	0,665	0,970	0,433	0,737	6,869	0,000	0,010	-0,470	2,771	-0,860	3,074	
W494G	VUS		26,20	0,781	0,619	0,798	0,414	0,835	8,376	0,000	0,760	-0,470	2,463	-0,076	2,578	
W494L	VUS		25,70	0,753	0,666	0,746	0,377	0,705	4,470	0,000	0,080	-0,470	1,933	-0,964	2,345	
W494R	VUS		25,40	0,779	0,701	0,982	0,481	0,832	5,970	0,000	1,670	-0,470	2,922	1,154	2,819	
W494S	VUS		26,40	0,813	0,645	0,926	0,495	0,810	7,276	0,000	0,740	-0,470	2,850	0,013	2,923	
R495C	VUS		22,60	0,588	0,405	0,135	0,098	0,449	1,141	0,372	1,660	1,720	-1,911	2,330	-1,883	
R495G	VUS		19,92	0,457	0,344	0,100	0,124	0,262	1,458	0,372	0,910	1,720	-2,790	1,187	-2,482	
R495H	VUS		18,30	0,533	0,351	0,075	0,050	0,162	1,911	0,372	1,030	1,720	-3,068	1,244	-2,721	
R495L	VUS		15,50	0,528	0,362	0,133	0,078	0,267	0,318	0,372	1,750	1,720	-2,973	2,129	-2,823	
R495P	VUS		16,29	0,575	0,374	0,209	0,231	0,274	-1,009	0,372	0,680	1,720	-2,475	0,990	-2,180	
R495S	VUS		18,71	0,557	0,345	0,171	0,075	0,222	1,448	0,372	0,930	1,720	-2,779	1,208	-2,464	
R496C	Pathogenic	Mild	24,50	0,615	0,424	0,146	0,221	0,766	3,080	0,182	1,660	0,080	-0,231	1,232	-0,232	
R496G	Likely pathogenic		23,50	0,586	0,365	0,166	0,248	0,561	4,410	0,182	0,910	0,080	-0,720	0,188	-0,496	
R496H	Pathogenic	Mild	21,10	0,566	0,377	0,102	0,104	0,592	6,803	0,182	1,030	0,080	-1,159	0,157	-0,863	Yes
R496L	Likely pathogenic		21,60	0,553	0,381	0,173	0,147	0,480	2,517	0,182	1,750	0,080	-1,248	1,087	-1,092	
R496P	Likely pathogenic		22,90	0,523	0,395	0,261	0,354	0,659	2,816	0,182	0,680	0,080	-0,385	0,005	-0,161	
R496S	Likely pathogenic		22,20	0,550	0,369	0,318	0,137	0,479	3,728	0,182	0,930	0,080	-1,116	0,121	-0,779	
Q497E	VUS		18,43	0,501	0,485	0,077	0,109	0,220	-0,082	0,898	0,400	1,590	-3,397	1,583	-3,065	
Q497H	VUS		21,10	0,590	0,511	0,179	0,103	0,380	0,288	0,898	0,470	1,590	-2,619	1,875	-2,412	
Q497K	VUS		21,70	0,505	0,517	0,090	0,134	0,293	-0,320	0,898	1,700	1,590	-2,813	3,323	-2,851	
Q497L	VUS		22,30	0,561	0,477	0,119	0,200	0,295	0,389	0,898	1,190	1,590	-2,572	2,767	-2,579	
Q497P	VUS		22,30	0,589	0,489	0,096	0,355	0,364	1,191	0,898	0,120	1,590	-2,090	1,573	-1,965	
Q497R	VUS		20,30	0,508	0,513	0,079	0,128	0,240	-1,285	0,898	0,560	1,590	-3,142	1,874	-2,866	

Table S2A : Contributions of Features to the First Two Principal Components

	Contribution to PC1	Contribution to PC2
Bfactor	-0,19	0,55
RSA	-0,29	0,44
DSA	0,04	0,67
LOF	0,40	0,09
REVEL	0,39	0,10
CADD	0,35	0,14
Alphamissense	0,38	0,10
DeMask	0,38	0,09
PrimateAI	0,31	0,05
DDG	0,21	-0,04

The table presents the weights of each feature in the first two principal components (PC1 and PC2) derived from the PCA analysis.
Component weights are shown for PC1 and PC2, with the absolute values used to calculate the total contribution of each feature.
Features are ranked by their total contribution highlighting their relative importance in explaining the variance within the dataset.
Abbreviations: DSA – Delta Solvent Accessibility; LOF – Loss-of-Function score; DDG – Free energy difference; RSA – Relative Solvent Accessibility.

Table S2B : Contributions of Features to the first component of Partial Least Squares regression analysis

	Contribution to PSL1 (Inverted)
Bfactor	-0,29
RSA	-0,32
DSA	0,02
LOF	0,37
REVEL	0,36
CADD	0,33
Alphamissense	0,38
DeMask	0,36
PrimateAI	0,32
DDG	0,27

Values represent the loadings of each variable on the first PLS component (PLS1), multiplied by –1 to align with the inverted direction used in the analysis and plots.
Higher positive values indicate stronger contribution to higher (more damaging) inverted PLS1 scores.
Abbreviations: DSA – Delta Solvent Accessibility; LOF – Loss-of-Function score; DDG – Free energy difference; RSA – Relative Solvent Accessibility.

Table S3A : Results of linear mixed-effects model on the β-Glucocerebrosidase activity according to Gaucher disease classification

	LMM with all patients available<br>Patients (N = 39)<br>Visits (N = 116)			Sensitivity analysis LMM without L444P carriers<br>Patients (N = 36)<br>Visits (N = 107)		
	coefficient ± standard error (SE)	Chisq	p	coefficient ± standard error (SE)	Chisq	p
Intercept	8.23 ± 2.92			8.73 ± 3.2		
Sex (Men)	0.36 ± 0.95	0.14	0.704	0.33 ± 1.03	0.1	0.751
Age at visit	0.02 ± 0.05	0.25	0.620	0.02 ± 0.05	0.09	0.762
GD classification		9.14	**0.010**		5.91	0.052
Mild	-2.39 ± 1.19			-2.39 ± 1.25		
Severe	-3.62 ± 1.40			-3.88 ± 2.27		

Results of linear mixed-effects models analyzing β-Glucocerebrosidase activity in relation to sex, age at visit, and Gaucher disease classification (risk variant, mild, or severe).
The left panel includes all patients with available data (N = 39, 116 visits); the right panel shows a sensitivity analysis excluding L444P carriers (N = 36, 107 visits).
The reference categories are women and risk variant category.
Significant effects are indicated in bold ($p < 0.05$).
Abbreviations : Linear mixed model (LMM), Gaucher disease (GD), Standard error (SE)

Table S3B : Results of linear mixed-effects model on the β-Glucocerebrosidase activity according to principal component classification

	All patients available<br>Patients (N = 43)<br>Visits (N = 127)			Sensitivity analysis without L444P carriers<br>Patients (N = 40)<br>Visits (N = 118)		
	coefficient ± standard error (SE)	Chisq	p	coefficient ± standard error (SE)	Chisq	p
Intercept	6.22 ± 2.80			6.58 ± 2.99		
Sex (Men)	0.01 ± 0.88	0	0.987	-0.06 ± 0.95	0	0.951
Age at visit	0.04 ± 0.05	0.66	0.418	0.03 ± 0.05	0.38	0.537
PC1	-0.93 ± 0.38	6.07	**0.014**	-1 ± 0.57	3.08	0.079
PC2	-0.54 ± 0.54	1.01	0.315	-0.61 ± 0.64	0.89	0.345

Results from linear mixed-effects models evaluating associations between β-Glucocerebrosidase activity and principal components PC1 and PC2, with adjustment for sex and age at visit.
The left panel includes all patients with available data (N = 43, 127 visits); the right panel shows a sensitivity analysis excluding L444P carriers (N = 40, 118 visits).
The reference category of sex is women .
Significant effects are indicated in bold ($p < 0.05$).
Abbreviations : Linear mixed model (LMM), Principal component (PC), Standard error (SE)

Table S4A : Results of linear mixed-effects model on the age at diagnosis according to Gaucher disease classification

	All patients available Patients (N = 591)			Sensitivity analysis without L444P carriers Patients (N = 533)		
	coefficient ± standard error (SE)	Chisq	p	coefficient ± standard error (SE)	Chisq	p
Intercept	55.06 ± 2.16			55.27 ± 2.09		
Sex (Men)	-0.15 ± 0.94	0.03	0.858	-0.51 ± 0.87	0.35	0.553
GD classification		16.73	**<0.001**		16.09	**<0.001**
Mild	1.60 ± 0.94			1.64 ± 0.94		
Severe	-3.57 ± 1.16			-5.02 ± 1.57		

Linear mixed-effects models evaluating the association between age at diagnosis and Gaucher disease classification (risk variant, mild, or severe), adjusting for sex.

The left panel includes all patients with available data (N = 591); the right panel shows a sensitivity analysis excluding L444P carriers (N = 533).

The reference categories are women and risk variant category.

Significant results are indicated in bold ($p < 0.05$).

Abbreviations : Linear mixed-effects model (LMM), Gaucher disease (GD), Standard error (SE)

Table S4B : Results of linear mixed-effects model on the age at diagnosis according to principal component classification

	All patients available Patients (N = 639)			Sensitivity analysis without L444P carriers Patients (N = 581)		
	coefficient ± standard error (SE)	Chisq	p	coefficient ± standard error (SE)	Chisq	p
Intercept	54.55 ± 2.28			54.54 ± 2.23		
Sex (Men)	-0.41 ± 0.81	0.26	0.612	-0.74 ± 0.84	0.76	0.384
PC1	-0.78 ± 0.30	6.65	**0.010**	-0.93 ± 0.39	5.61	**0.018**
PC2	-1.17 ± 0.48	5.87	**0.015**	-1.30 ± 0.53	6.08	**0.014**

Linear mixed-effects models evaluating the association between age at diagnosis and principal component classification, adjusting for sex.

The left panel includes all patients with available data (N = 639); the right panel shows a sensitivity analysis excluding L444P carriers (N = 581).

The reference category of sex is women .

Significant results are indicated in bold ($p < 0.05$).

Abbreviations : Linear mixed-effects model (LMM), Principal component (PC), Standard error (SE)

Table S5A : Results of linear mixed-effects model and sensitivity analysis on the cognitive progression according to Gaucher disease classification

	LMM with all patients available<br>Patients (N = 350)<br>Visits (N = 993)			Sensitivity analysis LMM without L444P carriers<br>Patients (N = 327)<br>Visits (N = 926)			Sensitivity analysis GEE with all patients available<br>Patients (N = 350)<br>Visits (N = 993)			Sensitivity analysis GEE without L444P carriers<br>Patients (N = 327)<br>Visits (N = 926)		
	coefficient ± standard error (SE)	Chisq	p	coefficient ± standard error (SE)	Chisq	p	coefficient ± standard error (SE)	Chisq	p	coefficient ± standard error (SE)	Chisq	p
Intercept	36.48 ± 1.4			36.11 ± 1.39			36.51 ± 1.27			35.92 ± 1.26		
Sex (Men)	-0.72 ± 0.36	10.1	**0.001**	-0.41 ± 0.37	9.5	**0.002**	-0.62 ± 0.34	8.5	**0.004**	-0.59 ± 0.35	10.6	**0.001**
Education level (below 12y)	-0.84 ± 0.42	9.2	**0.002**	-0.76 ± 0.43	7.1	**0.008**	-0.72 ± 0.45	14.9	**<0.001***	-0.66 ± 0.47	12.2	**<0.001**
Age at diagnosis	-0.13 ± 0.02	57.0	**<0.001**	-0.13 ± 0.02	54.2	**<0.001**	-0.15 ± 0.02	35.7	**<0.001***	-0.15 ± 0.02	29.6	**<0.001**
Duration between diagnosis and inclusion	-0.31 ± 0.04	57.5	**<0.001**	-0.30 ± 0.04	55.4	**<0.001**	-0.26 ± 0.06	21.1	**<0.001***	-0.26 ± 0.06	22.1	**<0.001**
GD classification		1.9	0.382		0.3	0.875		0.3	0.843		6.1	**0.048**
Mild	0.25 ± 0.5			0.22 ± 0.39			0.18 ± 0.37			0.16 ± 0.38		
Severe	-0.25 ± 0.55			-0.01 ± 0.70			-0.10 ± 0.52			-0.42 ± 0.51		
Duration since inclusion	0.92 ± 0.28	40.0	**<0.001**	1.07 ± 0.29	32.9	**<0.001**	0.46 ± 0.44	7.7	**0.005**	0.69 ± 0.47	5.5	**0.019**
Sex (Men) *Duration since inclusion	-0.34 ± 0.08	16.5	**<0.001**	-0.39 ± 0.08	22.4	**<0.001**	-0.30 ± 0.12	2.4	0.123	-0.59 ± 0.35	5.8	**0.017**
Education level (below 12y) *Duration since inclusion	-0.38 ± 0.11	11.5	**0.001**	-0.34 ± 0.11	9.3	**0.002**	-0.48 ± 0.19	6.4	**0.011**	-0.37 ± 0.18	4.5	**0.034**
Age at diagnosis *Duration since inclusion	-0.01 ± 0.01	7.8	**0.005**	-0.02 ± 0.01	10.2	**0.001**	-0.01 ± 0.01	0.8	0.363	-0.01 ± 0.01	1.32	0.251
Duration between diagnosis and inclusion *Duration since inclusion	-0.01 ± 0.02	0.3	0.572	-0.02 ± 0.02	0.83	0.362	-0.02 ± 0.02	0.7	0.409	-0.02 ± 0.02	1.12	0.290
GD classification*Duration since inclusion		9.2	**0.002**		5.96	0.051		2.5	0.282		0.92	0.630
Mild	-0.15 ± 0.08			-0.14 ± 0.08			-0.09 ± 0.14			-0.09 ± 0.14		
Severe	-0.44 ± 0.14			-0.49 ± 0.26			-0.46 ± 0.26			-0.45 ± 0.23		

Linear mixed-effects models and generalized estimating equations evaluating cognitive progression in relation to Gaucher disease classification (risk variant, mild, or severe), with interactions involving sex, education, age at diagnosis, and time since inclusion.

The leftmost panel includes all patients with available data (N = 350; 993 visits), followed by three sensitivity analyses: excluding L444P carriers (N = 327; 926 visits), generalized estimating equations with all patients, and generalized estimating equations excluding L444P.

The reference categories are women, more than 12 years of education and risk variant category.

Coefficients of main effects were extracted from complete model and thus they may be misleading due to interactions involvement.

Significant results are in bold ($p < 0.05$).

Abbreviations : Linear mixed-effects model (LMM), Gaucher disease (GD), Generalized estimating equations (GEE), Standard error (SE)

Table S5B : Results of linear mixed-effects model and sensitivity analysis on the cognitive progression according to principal component classification

	LMM with all patients available<br>Patients (N = 364)<br>Visits (N = 1046)			Sensitivity analysis LMM without L444P carriers<br>Patients (N = 341)<br>Visits (N = 979)			Sensitivity analysis GEE with all patients available<br>Patients (N = 364)<br>Visits (N = 1046)			Sensitivity analysis GEE without L444P carriers<br>Patients (N = 341)<br>Visits (N = 979)		
	coefficient ± standard error (SE)	Chisq	p	coefficient ± standard error (SE)	Chisq	p	coefficient ± standard error (SE)	Chisq	p	coefficient ± standard error (SE)	Chisq	p
Intercept	36.14 ± 1.34			35.78 ± 1.34			36.28 ± 1.22			35.80 ± 1.24		
Sex (Men)	-0.74 ± 0.36	11.27	**<0.001**	-0.66 ± 0.36	10.71	**0.001**	-0.67 ± 0.33	10.0	**0.002**	-0.67 ± 0.34	12.8	**<0.001**
Education level (below 12y)	-0.92 ± 0.41	10.34	**0.001**	-0.86 ± 0.42	8.72	**0.003**	-0.72 ± 0.43	13.7	**<0.001**	-0.66 ± 0.46	10.8	**<0.001**
Age at diagnosis	-0.13 ± 0.02	54.88	**<0.001**	-0.13 ± 0.02	50.62	**<0.001**	-0.15 ± 0.02	32.4	**<0.001**	-0.14 ± 0.02	26.7	**<0.001**
Duration between diagnosis and inclusion	-0.29 ± 0.04	53.66	**<0.001**	-0.28 ± 0.04	51.12	**<0.001**	-0.24 ± 0.06	20.0	**<0.001**	-0.24 ± 0.06	20.2	**<0.001**
PC1	-0.07 ± 0.15	2.14	0.143	-0.07 ± 0.19	0.93	0.336	-0.02 ± 0.13	0.0	0.926	-0.07 ± 0.12	1.1	0.287
PC2	-0.47 ± 0.23	2.53	**0.026**	-0.47 ± 0.25	4.09	**0.043**	-0.39 ± 0.21	0.0	0.928	-0.29 ± 0.20	1.0	0.323
Duration since inclusion	0.57 ± 0.27	32.92	**<0.001**	0.71 ± 0.27	26.38	**<0.001**	0.24 ± 0.06	7.1	**0.008**	0.39 ± 0.42	5.0	**0.025**
Sex (Men) *Duration since inclusion	-0.35 ± 0.08	20.21	**<0.001**	-0.41 ± 0.08	26.88	**<0.001**	-0.34 ± 0.12	3.4	0.07	-0.40 ± 0.12	6.8	**0.010**
Education level (below 12y) *Duration since inclusion	-0.34 ± 0.1	10.91	**<0.001**	-0.32 ± 0.1	9.36	**0.002**	-0.39 ± 0.18	5.1	**0.025**	-0.31 ± 0.17	3.4	0.064
Age at diagnosis *Duration since inclusion	-0.01 ± 0	4.72	**0.030**	-0.01 ± 0	7.02	**0.008**	-0.01 ± 0.01	0.4	0.519	-0.01 ± 0.01	0.9	0.357
Duration between diagnosis and inclusion *Duration since inclusion	-0.02 ± 0.02	0.87	0.352	-0.02 ± 0.02	1.67	0.196	-0.02 ± 0.02	0.9	0.340	-0.03 ± 0.02	1.3	0.253
PC1*Duration since inclusion	-0.15 ± 0.04	17.04	**<0.001**	-0.18 ± 0.07	7.86	**0.005**	-0.15 ± 0.07	7.4	**0.007**	-0.15 ± 0.07	6.4	**0.011**
PC2*Duration since inclusion	-0.01 ± 0.05	0.06	0.801	-0.05 ± 0.07	0.48	0.487	-0.02 ± 0.09	0.0	0.887	-0.03 ± 0.09	0.5	0.497

Notes. Coefficients of main effects were extracted from complete LMM and thus they may be misleading due to interactions involvement.

The reference categories are women and more than 12 years of education.

Linear mixed-effects models and generalized estimating equations evaluating cognitive progression in relation to principal component classification (risk variant, mild, or severe), with interactions involving sex, education, age at diagnosis, and time since inclusion.

The leftmost panel includes all patients with available data (N = 364; 1046 visits), followed by three sensitivity analyses: excluding L444P carriers (N = 341; 979 visits), generalized estimating equations with all patients, and generalized estimating equations excluding L444P.

The reference categories are women and more than 12 years of education.

Coefficients of main effects were extracted from complete model and thus they may be misleading due to interactions involvement.

Significant results are in bold ($p < 0.05$).

Abbreviations : Linear mixed-effects model (LMM), Principal component (PC), Generalized estimating equations (GEE), Standard error (SE)

Table S6A : Results of linear mixed-effects model and sensitivity analysis on the motor progression according to Gaucher disease classification

	LMM with all patients available<br>Patients (N = 261)<br>Visits (N = 1108)			Sensitivity analysis LMM without L444P carriers<br>Patients (N = 246)<br>Visits (N = 1042)			Sensitivity analysis GEE with all patients available<br>Patients (N = 261)<br>Visits (N = 1108)			Sensitivity analysis GEE without L444P carriers<br>Patients (N = 246)<br>Visits (N = 1042)		
	coefficient ± standard error (SE)	Chisq	p	coefficient ± standard error (SE)	Chisq	p	coefficient ± standard error (SE)	Chisq	p	coefficient ± standard error (SE)	Chisq	p
Intercept	19.10 ± 5.62			18.15 ± 5.65			15.39 ± 5.50			14.46 ± 5.70		
Sex (Men)	1.33 ± 1.50	4.4	**0.036**	1.12 ± 1.55	3.9	**0.049**	1.53 ± 1.41	7.7	**0.005**	1.28 ± 1.44	6.9	**0.009**
Age at diagnosis	0.06 ± 0.08	5.1	**0.024**	0.09 ± 0.09	7.2	**0.007**	0.11 ± 0.09	0.2	0.638	0.13 ± 0.10	0.6	0.427
Duration between diagnosis and baseline visit	0.69 ± 0.17	21.9	**<0.001**	0.68 ± 0.18	20.3	**<0.001**	0.84 ± 0.24	19.5	**<0.001**	0.83 ± 0.24	18.4	**<0.001**
GD classification		7.6	**0.022**		8.0	**0.019**		9.6	**0.008**		8.8	**0.012**
Mild	-3.71 ± 1.62			-3.74 ± 1.77			-3.17 ± 1.47			-3.30 ± 1.47		
Severe	-5.25 ± 2.52			-2.78 ± 3.55			-5.73 ± 2.66			-5.50 ± 3.48		
Duration since baseline visit	-4.09 ± 1.18	21.9	**<0.001**	-4.68 ± 1.19	28.8	**<0.001**	-3.49 ± 1.76	9.5	**0.002**	-4.09 ± 1.78	7.9	**0.005**
Sex (Men) *Duration since baseline visit	0.99 ± 0.3	10.9	**0.001**	1.07 ± 0.3	12.5	**<0.001**	1.01 ± 0.38	6.2	**0.013**	1.10 ± 0.38	7.1	**0.008**
Age at diagnosis *Duration since baseline visit	0.06 ± 0.08	13.9	**<0.001**	0.08 ± 0.02	18.2	**<0.001**	0.06 ± 0.03	3.6	0.059	0.07 ± 0.03	4.9	**0.027**
Duration between diagnosis and baseline visit *Duration since baseline visit	0.09 ± 0.06	2.2	0.143	0.08 ± 0.06	1.9	0.171	0.09 ± 0.09	1.1	0.289	0.08 ± 0.09	0.9	0.334
GD classification*Duration since diagnosis		6.9	**0.031**		4.3	0.115		1.4	0.508		0.9	0.649
Mild	-0.21 ± 0.32			-0.19 ± 0.31			-0.22 ± 0.40			-0.21 ± 0.39		
Severe	1.3 ± 0.58			1.82 ± 1.04			1.26 ± 0.86			2.06 ± 1.43		

Linear mixed-effects models and generalized estimating equations evaluating cognitive progression in relation to Gaucher disease classification (risk variant, mild, or severe), with interactions involving sex, age at diagnosis, and time since inclusion.

The leftmost panel includes all patients with available data (N = 261; 1108 visits), followed by three sensitivity analyses: excluding L444P carriers (N = 246; 1042 visits), generalized estimating equations with all patients, and generalized estimating equations excluding L444P.

The reference categories are women and risk variant category.

Coefficients of main effects were extracted from complete model and thus they may be misleading due to interactions involvement.

Significant results are in bold ($p < 0.05$).

Abbreviations : Linear mixed-effects model (LMM), Gaucher disease (GD), Generalized estimating equations (GEE), Standard error (SE)

Table S6B : Results of linear mixed-effects model and sensitivity analysis on the motor progression according to principal component classification

	LMM with all patients available<br>Patients (N = 271)<br>Visits (N = 1164)			Sensitivity analysis LMM without L444P carriers<br>Patients (N = 256)<br>Visits (N = 1098)			Sensitivity analysis GEE with all patients available<br>Patients (N = 271)<br>Visits (N = 1164)			Sensitivity analysis GEE without L444P carriers<br>Patients (N = 256)<br>Visits (N = 1098)		
	coefficient ± standard error (SE)	Chisq	p	coefficient ± standard error (SE)	Chisq	p	coefficient ± standard error (SE)	Chisq	p	coefficient ± standard error (SE)	Chisq	p
Intercept	15.47 ± 5.32			14.61 ± 5.41			11.62 ± 5.25			10.76 ± 5.31		
Sex (Men)	1.68 ± 1.46	5.97	**0.015**	1.44 ± 1.48	5.42	**0.020**	1.96 ± 1.38	10.4	**0.001**	1.74 ± 1.40	9.7	**0.002**
Age at diagnosis	0.06 ± 0.08	5.30	**0.021**	0.08 ± 0.08	7.32	**0.007**	0.11 ± 0.08	0.2	0.684	0.13 ± 0.09	0.6	0.458
Duration between diagnosis and inclusion	0.73 ± 0.17	23.56	**<0.001**	0.72 ± 0.17	22.48	**<0.001**	0.88 ± 0.24	20.5	**<0.001**	0.87 ± 0.24	19.2	**<0.001**
PC1	-1.56 ± 0.68	1.79	0.181	-1.40 ± 0.93	0.35	0.554	-1.48 ± 0.71	7.5	**0.006**	-1.34 ± 0.90	7.2	**0.007**
PC2	0.91 ± 0.97	2.50	0.113	1.15 ± 1.16	3.06	0.080	0.99 ± 0.97	1.5	0.225	1.20 ± 1.13	1.5	0.221
Duration since inclusion	-3.44 ± 1.11	39.58	**<0.001**	-3.95 ± 1.10	34.86	**<0.001**	-2.90 ± 1.67	11.5	**<0.001**	-3.32 ± 1.64	9.7	**0.002**
Sex (Men) *Duration since inclusion	0.99 ± 0.28	12.36	**<0.001**	1.08 ± 0.28	14.51	**<0.001**	1.01 ± 0.36	6.5	**0.011**	1.11 ± 0.35	7.4	**0.007**
Age at diagnosis *Duration since inclusion	0.07 ± 0.02	14.01	**<0.001**	0.08 ± 0.02	19.21	**<0.001**	0.06 ± 0.03	3.9	**0.049**	0.07 ± 0.03	5.7	**0.017**
Duration between diagnosis and inclusion *Duration since inclusion	0.06 ± 0.06	1.01	0.316	0.05 ± 0.06	0.86	0.354	0.06 ± 0.09	0.5	0.478	0.05 ± 0.09	0.41	0.522
PC1*Duration since inclusion	0.46 ± 0.15	8.97	**0.003**	0.75 ± 0.27	7.94	**0.005**	0.44 ± 0.23	1.7	0.192	0.78 ± 0.32	1.25	0.264
PC2*Duration since inclusion	0.34 ± 0.20	2.98	0.084	0.63 ± 0.29	4.63	**0.031**	0.31 ± 0.27	0.1	0.725	0.64 ± 0.34	0.5	0.471

Linear mixed-effects models and generalized estimating equations evaluating cognitive progression in relation to principal component classification (risk variant, mild, or severe), with interactions involving sex, education, age at diagnosis, and time since inclusion.

The leftmost panel includes all patients with available data (N = 271; 1164 visits), followed by three sensitivity analyses: excluding L444P carriers (N = 256; 1098 visits), generalized estimating equations with all patients, and generalized estimating equations excluding L444P.

The reference categories are women.

Coefficients of main effects were extracted from complete model and thus they may be misleading due to interactions involvement.

Significant results are in bold ($p < 0.05$).

Abbreviations : Linear mixed-effects model (LMM), Principal component (PC), Generalized estimating equations (GEE), Standard error (SE)

Table S7 : Variant effect predictors and structural scores for *GBA1* missense variant

Score	Link to score
**Variant effect predictors**	
CADD score	dbNSFP
REVEL score	dbNSFP
Primate AI score	dbNSFP
Alphamissense	https://alphamissense.hegelab.org/
Demask	https://demask.princeton.edu/query/
LOF score	https://itanlab.shinyapps.io/goflof/
**Structural scores**	
Free energy difference	https://www.sigma-pred.org/
RSA	https://www.sigma-pred.org/
DSA	https://envision.gs.washington.edu/
B-factor	https://envision.gs.washington.edu/

Abbreviations: RSA: relative solvent accessibility; DSA: delta of solvent accessibility

STROBE statement: Reporting guidelines checklist for cohort, case-control and cross-sectional studies

SECTION	ITEM NUMBER	CHECKLIST ITEM	REPORTED ON PAGE NUMBER:
**TITLE AND ABSTRACT**			
	1a	Indicate the study's design with a commonly used term in the title or the abstract	1
	1b	Provide in the abstract an informative and balanced summary of what was done and what was found	3
**INTRODUCTION**			
Background and objectives	2	Explain the scientific background and rationale for the investigation being reported	4
	3	State specific objectives, including any pre-specified hypotheses	5
**METHODS**			
Study design	4	Present key elements of study design early in the paper	15
Setting	5	Describe the setting, locations, and relevant dates, including periods of recruitment, exposure, follow-up, and data collection	15
Participants	6a	Cohort study—Give the eligibility criteria, and the sources and methods of selection of participants. Describe methods of follow-up<br>Case-control study—Give the eligibility criteria, and the sources and methods of case ascertainment and control selection. Give the rationale for the choice of cases and controls<br>Cross-sectional study—Give the eligibility criteria, and the sources and methods of selection of participants	15
	6b	Cohort study—For matched studies, give matching criteria and number of exposed and unexposed<br>Case-control study—For matched studies, give matching criteria and the number of controls per case<br>Variables	15
Variables	7	Clearly define all outcomes, exposures, predictors, potential confounders, and effect modifiers. Give diagnostic criteria, if applicable	16
Data sources/measurements	8*	For each variable of interest, give sources of data and details of methods of assessment (measurement). Describe comparability of assessment methods if there is more than one group.	16

SECTION	ITEM NUMBER	CHECKLIST ITEM	REPORTED ON PAGE NUMBER:
Bias	9	Describe any efforts to address potential sources of bias.	19
Study size	10	Explain how the study size was arrived at	19
Quantitative variables	11	Explain how quantitative variables were handled in the analyses. If applicable, describe which groupings were chosen and why .	19
Statistical methods	12a	Describe all statistical methods, including those used to control for confounding	19-21
	12b	Describe any methods used to examine subgroups and interactions	19-21
	12c	Explain how missing data were addressed	8
	12d	Cohort study—If applicable, explain how loss to follow-up was addressed<br>Case-control study—If applicable, explain how matching of cases and controls was addressed<br>Cross-sectional study—If applicable, describe analytical methods taking account of sampling strategy	NA
	12e	Describe any sensitivity analyses	21
**RESULTS**			
Participants	13a	Report numbers of individuals at each stage of study—eg numbers potentially eligible, examined for eligibility, confirmed eligible, included in the study, completing follow-up, and analysed	6
	13b	Give reasons for non-participation at each stage	6
	13c	Consider use of a flow diagram	6
Descriptive Data	14a	Give characteristics of study participants (eg demographic, clinical, social) and information on exposures and potential confounders	6
	14b	Indicate number of participants with missing data for each variable of interest	31
	14c	Cohort study—Summarise follow-up time (eg, average and total amount)	8
Outcome Data	15*	Cohort study—Report numbers of outcome events or summary measures over time<br>Case-control study—Report numbers in each exposure category, or summary measures of exposure<br>Cross-sectional study—Report numbers of outcome events or summary measures	8
Main Results	16a	Give unadjusted estimates and, if applicable, confounder-adjusted estimates and their precision (e.g. 95% confidence interval). Make clear which confounders were adjusted for and why they were included	8-9

SECTION	ITEM NUMBER	CHECKLIST ITEM	REPORTED ON PAGE NUMBER:
	16b	Report category boundaries when continuous variables were categorized	6-9
	16c	If relevant, consider translating estimates of relative risk into absolute risk for a meaningful time period	NA
	16d	Report results of any adjustments for multiple comparisons	NA
Other Analyses	17a	Report other analyses done—e.g. analyses of subgroups and interactions, and sensitivity analyses	6-9
	17b	If numerous genetic exposures (genetic variants) were examined, summarize results from all analyses undertaken	NA
	17c	If detailed results are available elsewhere, state how they can be accessed	9
**DISCUSSION**			
Key Results	18	Summarise key results with reference to study objectives	10
Limitations	19	Discuss limitations of the study, taking into account sources of potential bias or imprecision. Discuss both direction and magnitude of any potential bias	12-13
Interpretation	20	Give a cautious overall interpretation of results considering objectives, limitations, multiplicity of analyses, results from similar studies, and other relevant evidence	12-13
Generalisability	21	Discuss the generalisability (external validity) of the study results<br>Other information	12-13
**FUNDING**			
	22	Give the source of funding and the role of the funders for the present study and, if applicable, for the original study on which the present article is based	22

*Give information separately for cases and controls in case-control studies and, if applicable, for exposed and unexposed groups in cohort and cross-sectional studies.
